# Supplementary material for: Flexible changes to the Heliothis virescens ascovirus 3h (HvAV-3h) virion components affect pathogenicity against different host larvae species
Source: Microbiol Spectr. 2023 Nov 9;11(6):e02488-23. doi: 10.1128/spectrum.02488-23 (PMC10714839; doi:10.1128/spectrum.02488-23)

Supplementary Figures

Figure S1

Expression and purification of His-tag fused HvAV-3h coded proteins selected according to Figure 2C (marked as red words). The purified proteins were then sent to Wuhan Institute of Virology, Chinese Academy of Sciences for the preparation of specific rabbit polyclonal antibody.

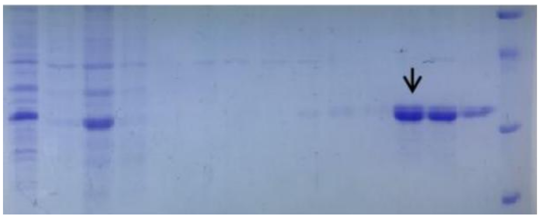

His-3H-21

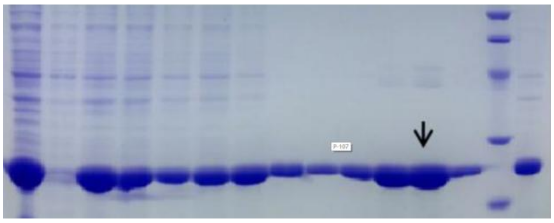

His-3H-107

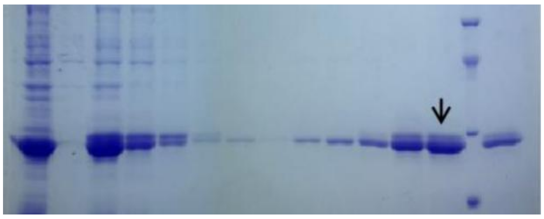

His-3H-117

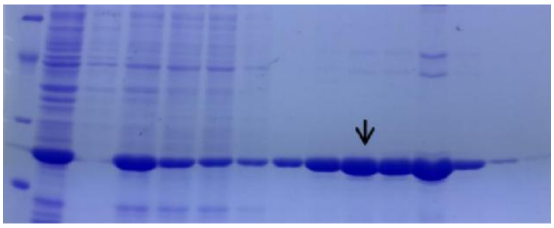

His-3H-122

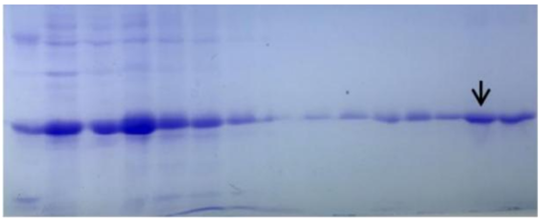

His-3H-135

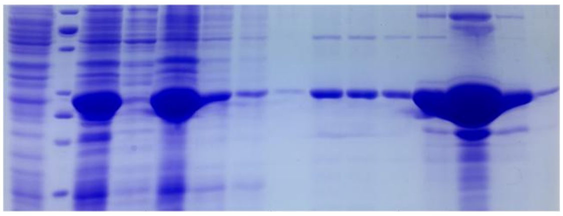

His-3H-135

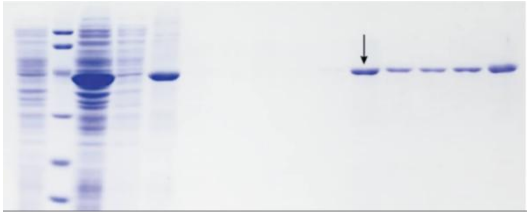

His-3H-38

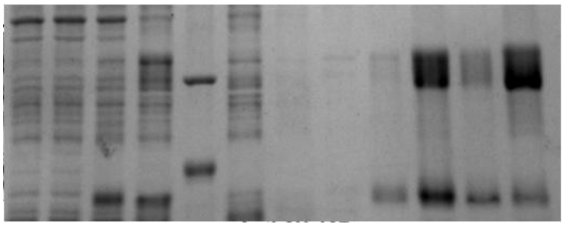

His-3H-171

Figure S2

Immunogold staining of 3H-21, 3H-43, 3H-53, 3H-107, and 3H-117, 3H-135 protein in the thin sections of hemocytes collected from HvAV-3h infected *Mythimna separata* (120 hpi). The right images were enlarged from the red box of the left images.

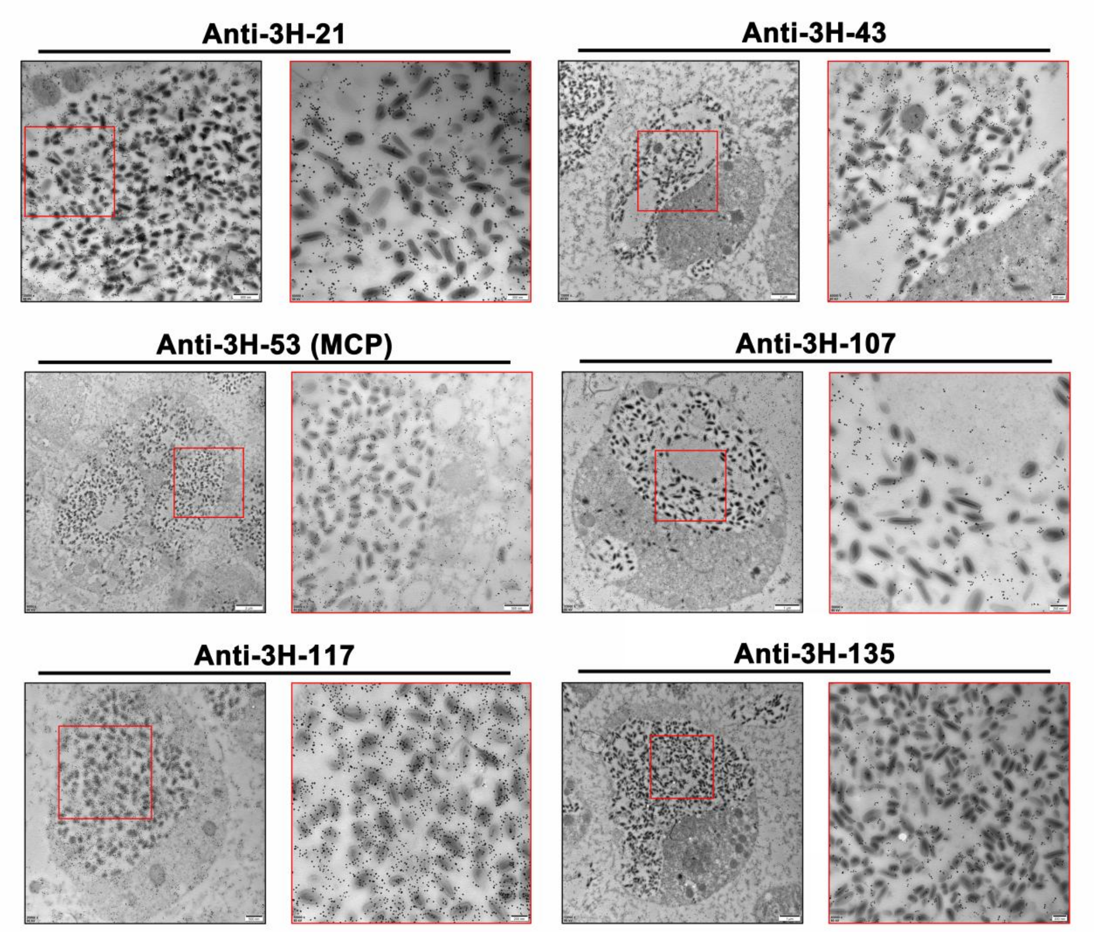

Figure S3

Determination of carboxylesterases (CarEs) activity of 3rd instar *H. armigera*, *M. separata*, *S. exigua*, *S. frugiperda*, and *S. litura* larvae after the inoculation of HvAV-3h. The asterisks indicate the significant differences between the larval detoxification enzyme activity and healthy larval detoxification enzyme activity at each tested time of point based on one-way ANOVA followed by LSD comparisons (α = 0.05).

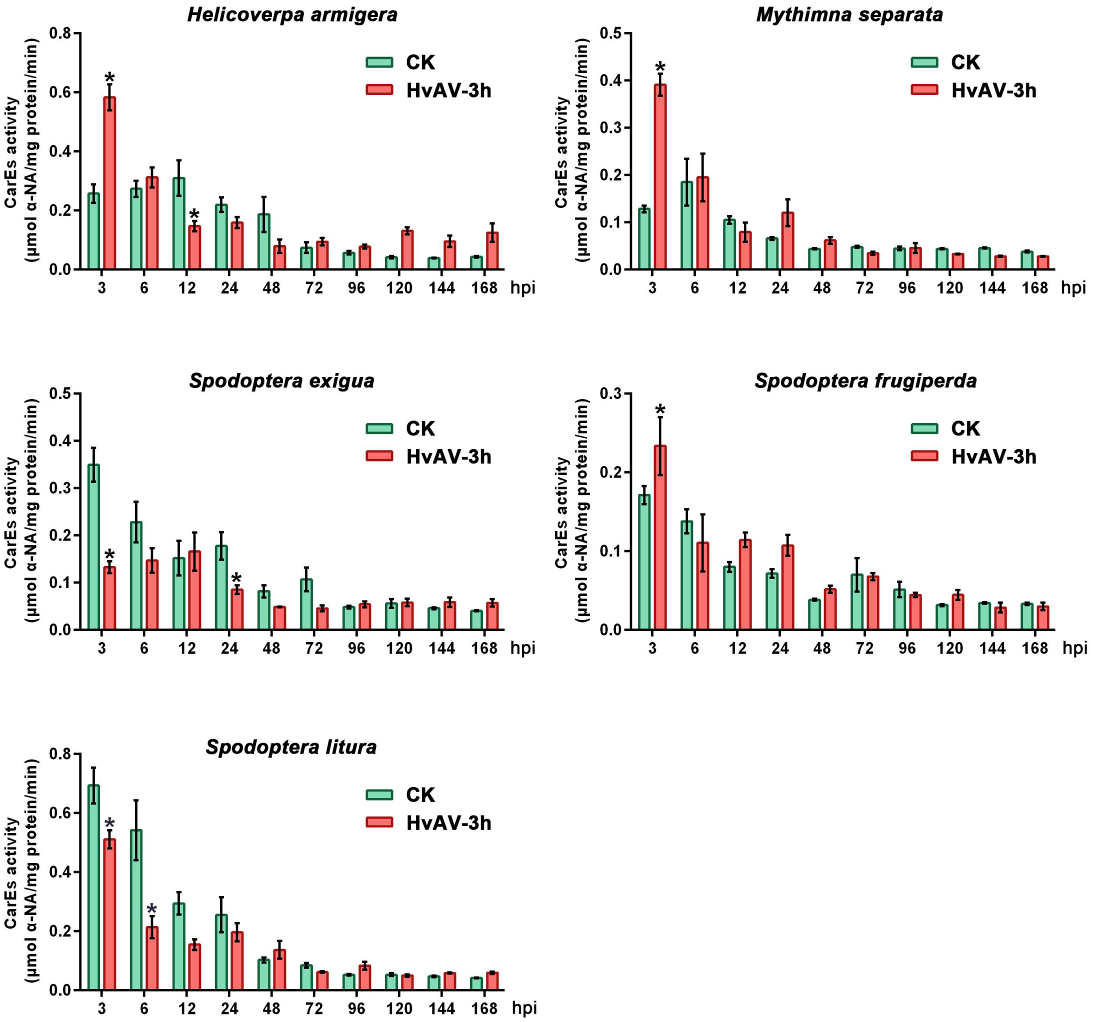

Figure S4

Determination of glutathione S-transferases (GSTs) activity of 3rd instar *H. armigera*, *M. separata*, *S. exigua*, *S. frugiperda*, and *S. litura* larvae after the inoculation of HvAV-3h. The asterisks indicate the significant differences between the larval detoxification enzyme activity and healthy larval detoxification enzyme activity at each tested time of point based on one-way ANOVA followed by LSD comparisons (α = 0.05).

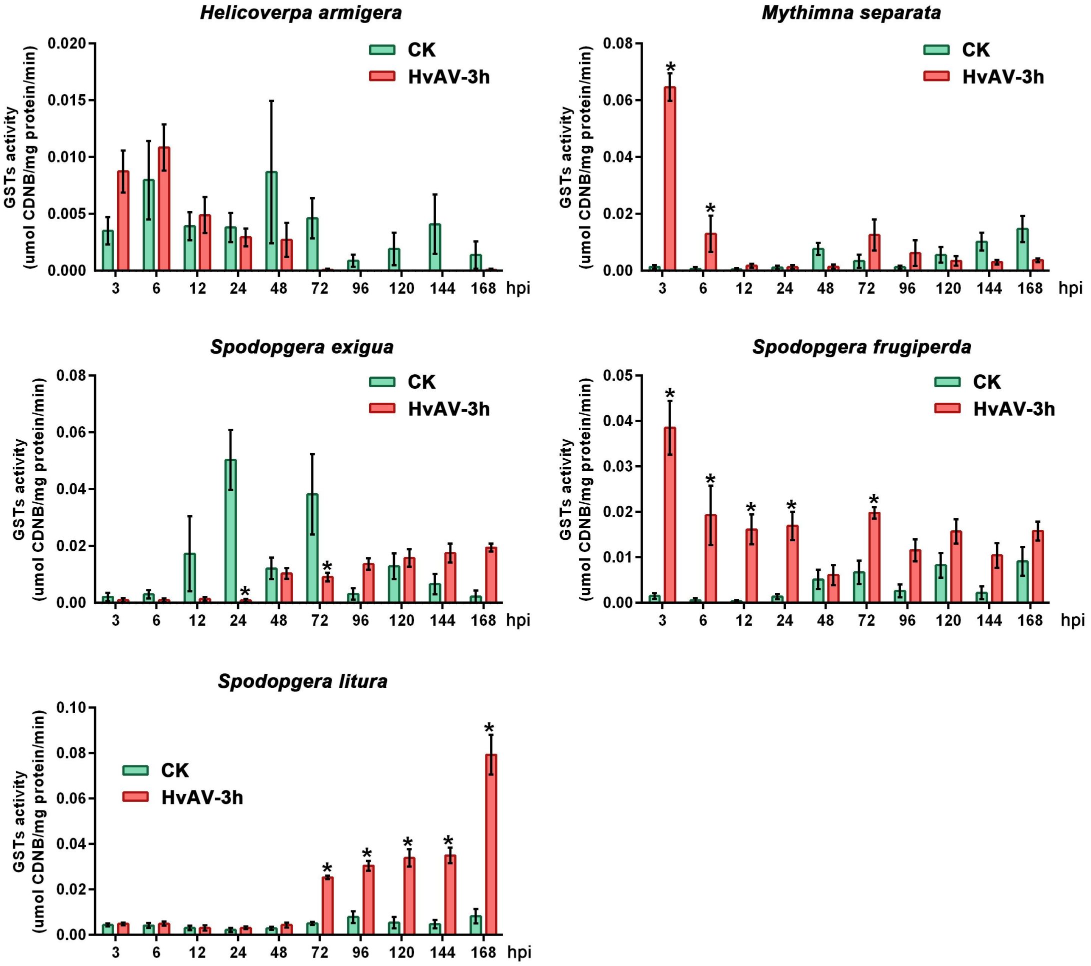

Figure S5

Determination of cytochrome P450 monooxygenases (P450s) activity of 3rd instar *H. armigera*, *M. separata*, *S. exigua*, *S. frugiperda*, and *S. litura* larvae after the inoculation of HvAV-3h. The asterisks indicate the significant differences between the larval detoxification enzyme activity and healthy larval detoxification enzyme activity at each tested time of point based on one-way ANOVA followed by LSD comparisons (α = 0.05).

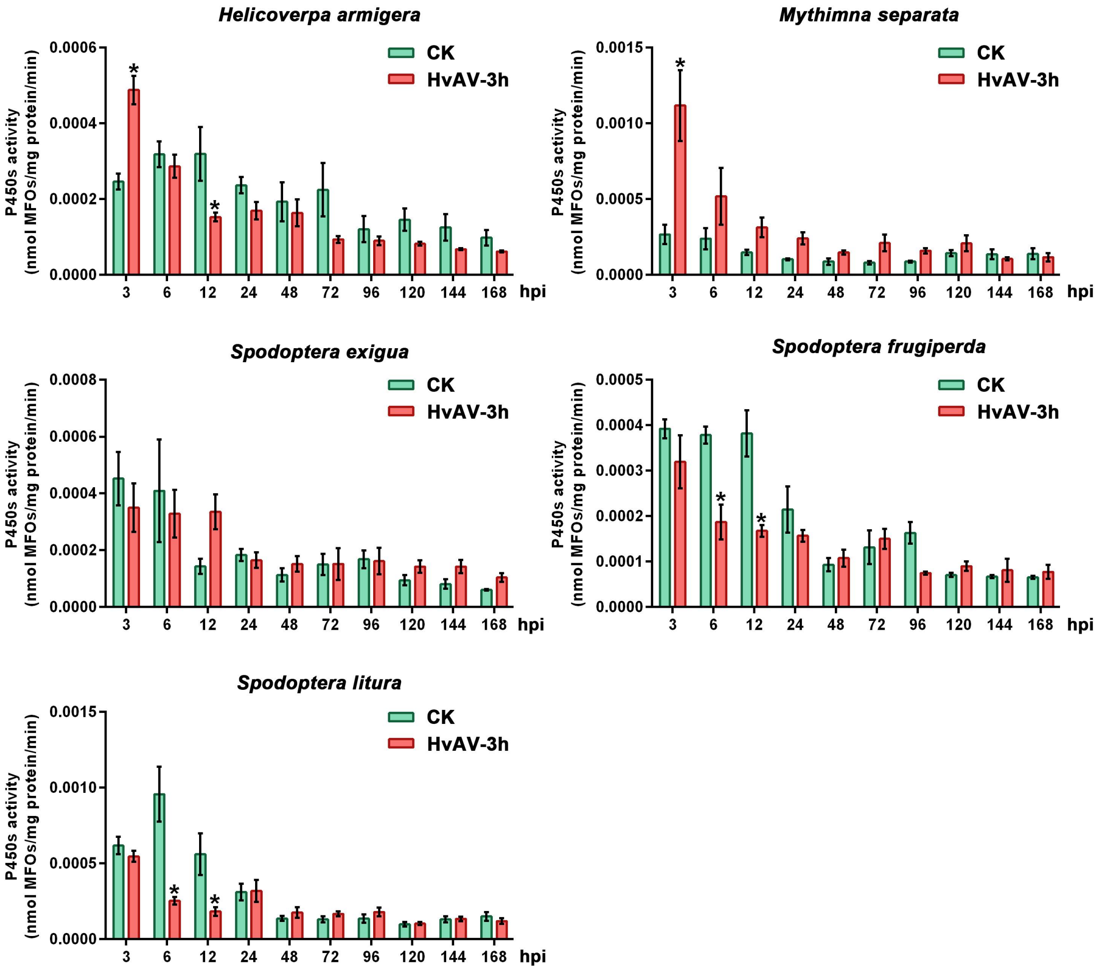

Figure S6

Determination of detoxification enzyme activity the *H. armigera* larvae infection by specific polyclonal antibody (3H-43, 3H-53, 3H-107, or 3H-117) blocked HvAV-3h or the larvae infected by IgG incubated HvAV-3h (used as control). The asterisks indicate the significant differences between the detoxification enzyme activity larvae infection by specific polyclonal antibody blocked HvAV-3h and the larvae infected by IgG incubated HvAV-3h at each tested time of point based on one-way ANOVA followed by LSD comparisons (α = 0.05).

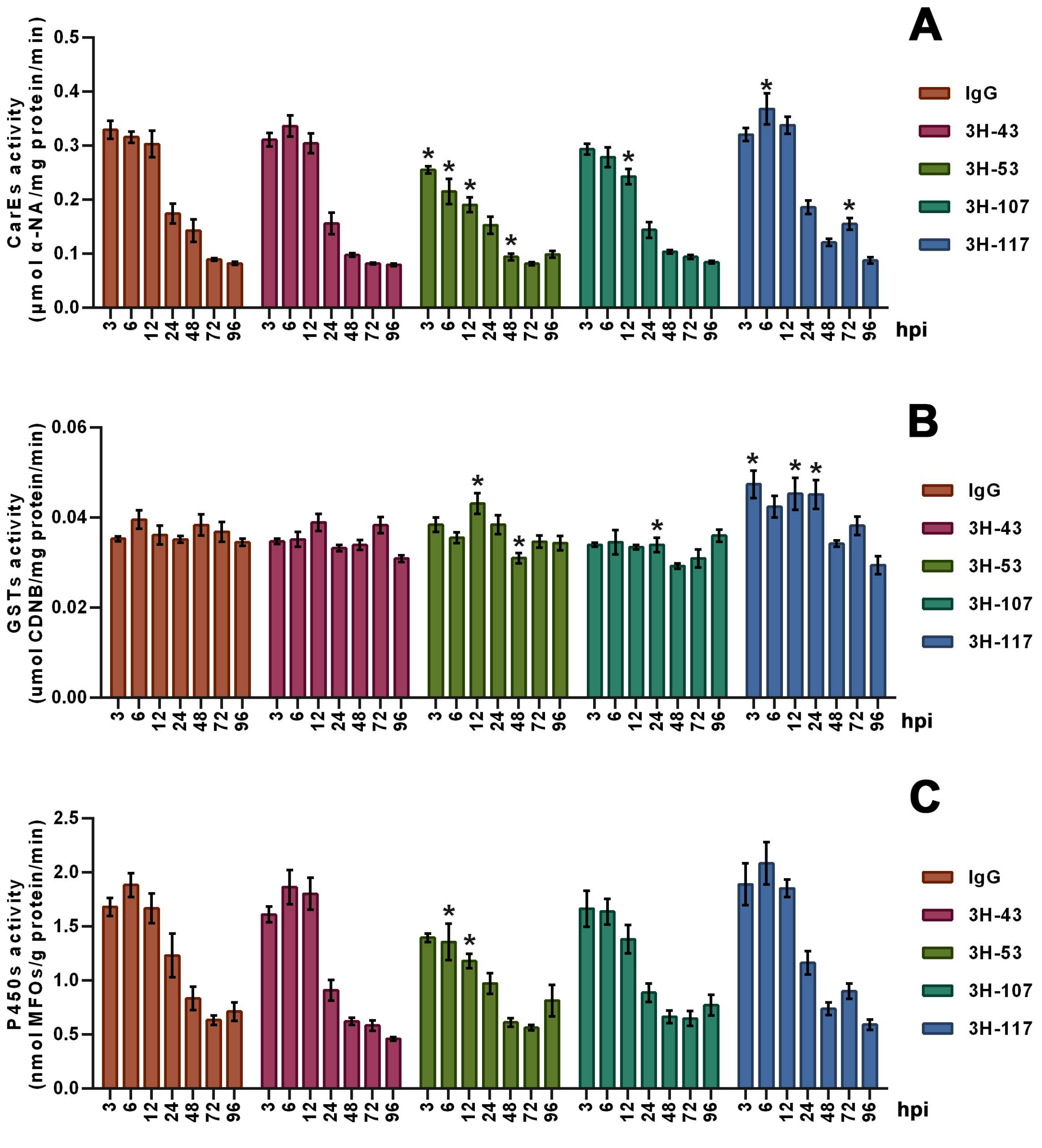

Figure S7

Determination of detoxification enzyme activity the *M. separata* larvae infection by specific polyclonal antibody (3H-43, 3H-53, 3H-107, or 3H-117) blocked HvAV-3h or the larvae infected by IgG incubated HvAV-3h (used as control). The asterisks indicate the significant differences between the detoxification enzyme activity larvae infection by specific polyclonal antibody blocked HvAV-3h and the larvae infected by IgG incubated HvAV-3h at each tested time of point based on one-way ANOVA followed by LSD comparisons ($\alpha = 0.05$).

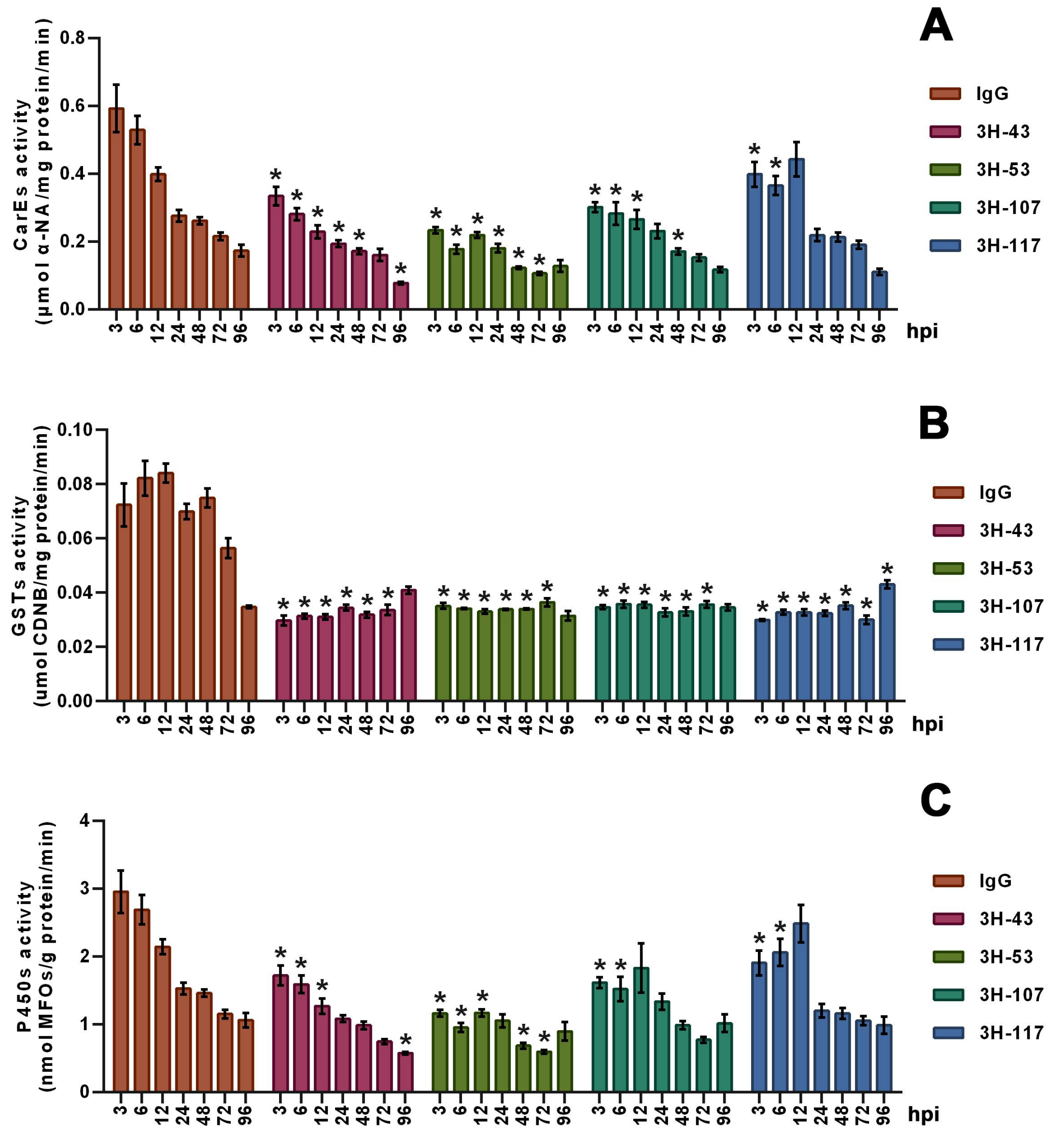

Figure S8

Determination of detoxification enzyme activity the *S. exigua* larvae infection by specific polyclonal antibody (3H-43, 3H-53, 3H-107, or 3H-117) blocked HvAV-3h or the larvae infected by IgG incubated HvAV-3h (used as control). The asterisks indicate the significant differences between the detoxification enzyme activity larvae infection by specific polyclonal antibody blocked HvAV-3h and the larvae infected by IgG incubated HvAV-3h at each tested time of point based on one-way ANOVA followed by LSD comparisons (α = 0.05).

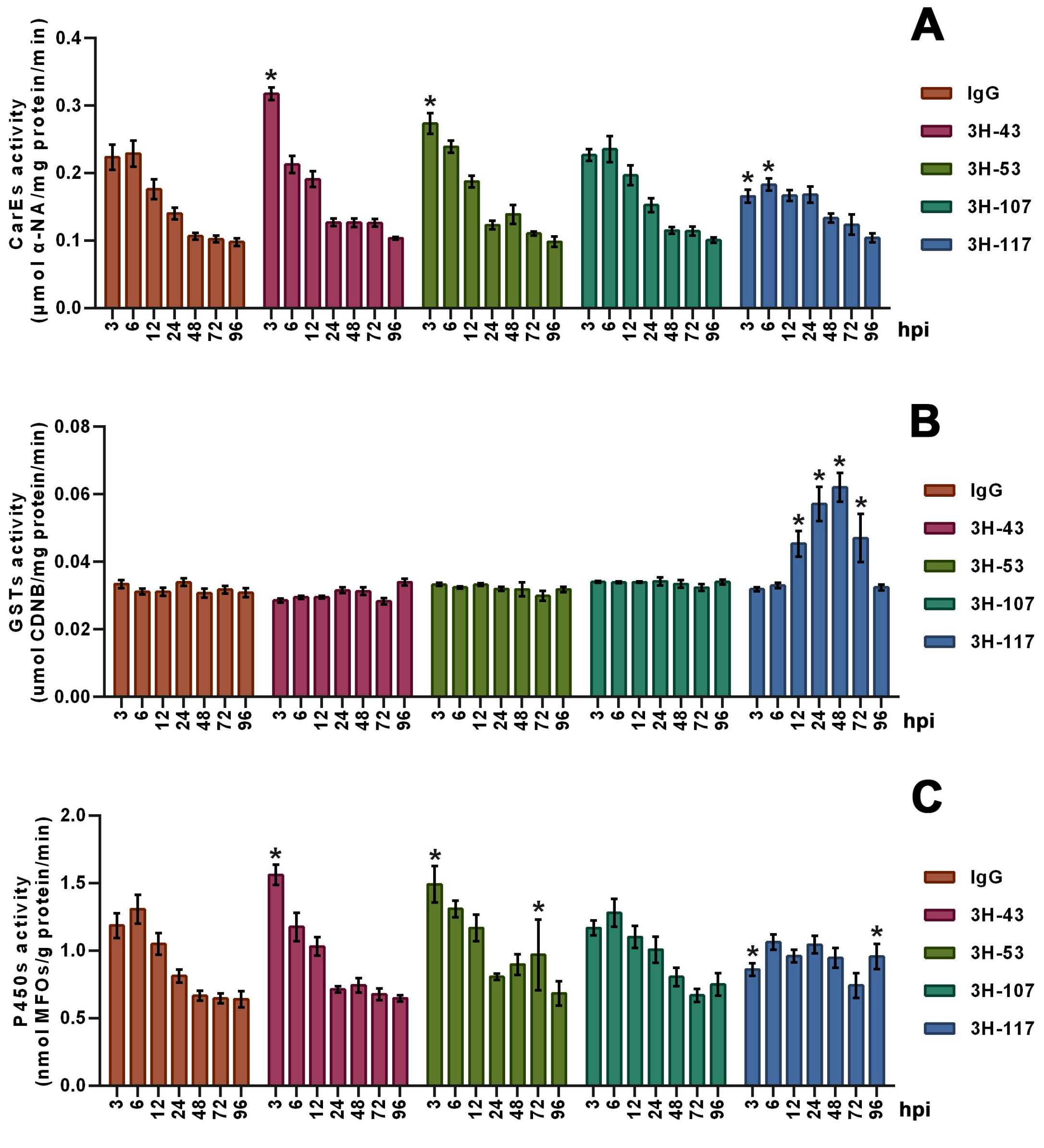

Figure S9

Determination of detoxification enzyme activity the *S. frugiperda* larvae infection by specific polyclonal antibody (3H-43, 3H-53, 3H-107, or 3H-117) blocked HvAV-3h or the larvae infected by IgG incubated HvAV-3h (used as control). The asterisks indicate the significant differences between the detoxification enzyme activity larvae infection by specific polyclonal antibody blocked HvAV-3h and the larvae infected by IgG incubated HvAV-3h at each tested time of point based on one-way ANOVA followed by LSD comparisons (α = 0.05).

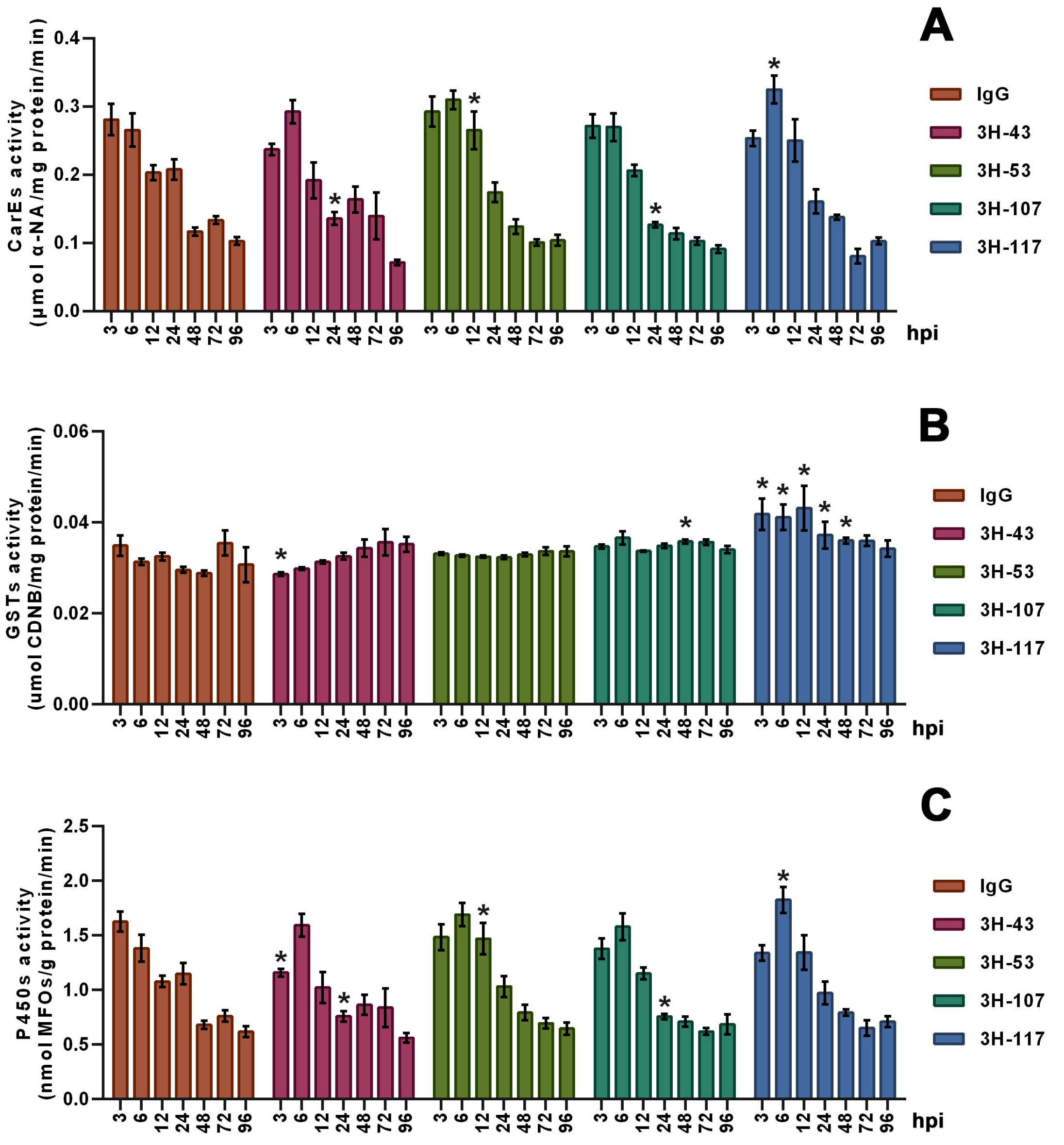

Figure S10

Determination of detoxification enzyme activity the *S. litura* larvae infection by specific polyclonal antibody (3H-43, 3H-53, 3H-107, or 3H-117) blocked HvAV-3h or the larvae infected by IgG incubated HvAV-3h (used as control). The asterisks indicate the significant differences between the detoxification enzyme activity larvae infection by specific polyclonal antibody blocked HvAV-3h and the larvae infected by IgG incubated HvAV-3h at each tested time of point based on one-way ANOVA followed by LSD comparisons (α = 0.05).

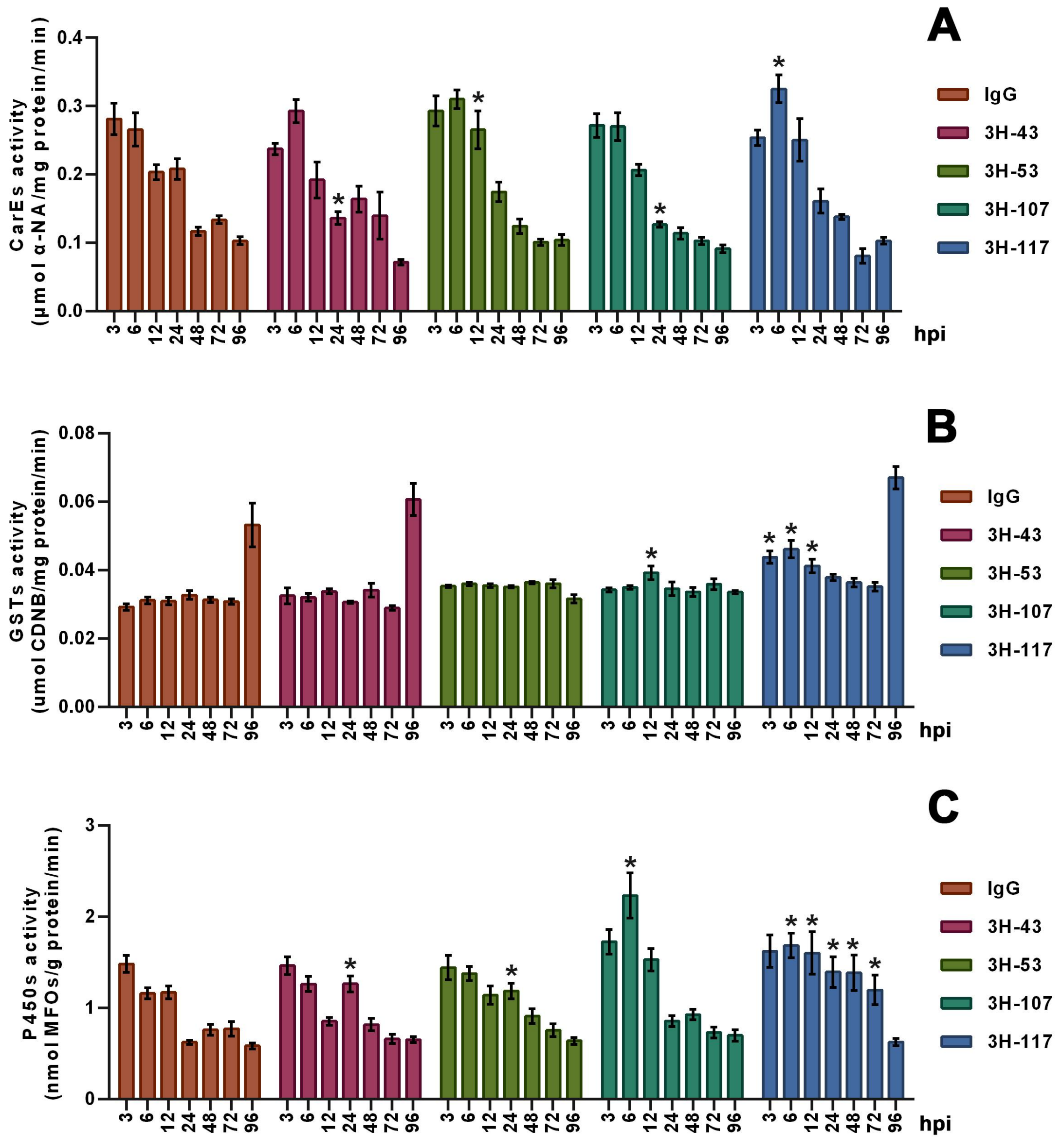

Figure S11

Expression and purification of His-tag fused host larval GST. A. Expression and purification of His-tag fused *S. frugiperda* larval GST (His-Sf-GST). B. Expression and purification of His-tag fused *S. litura* larval GST (His-Sl-GST).

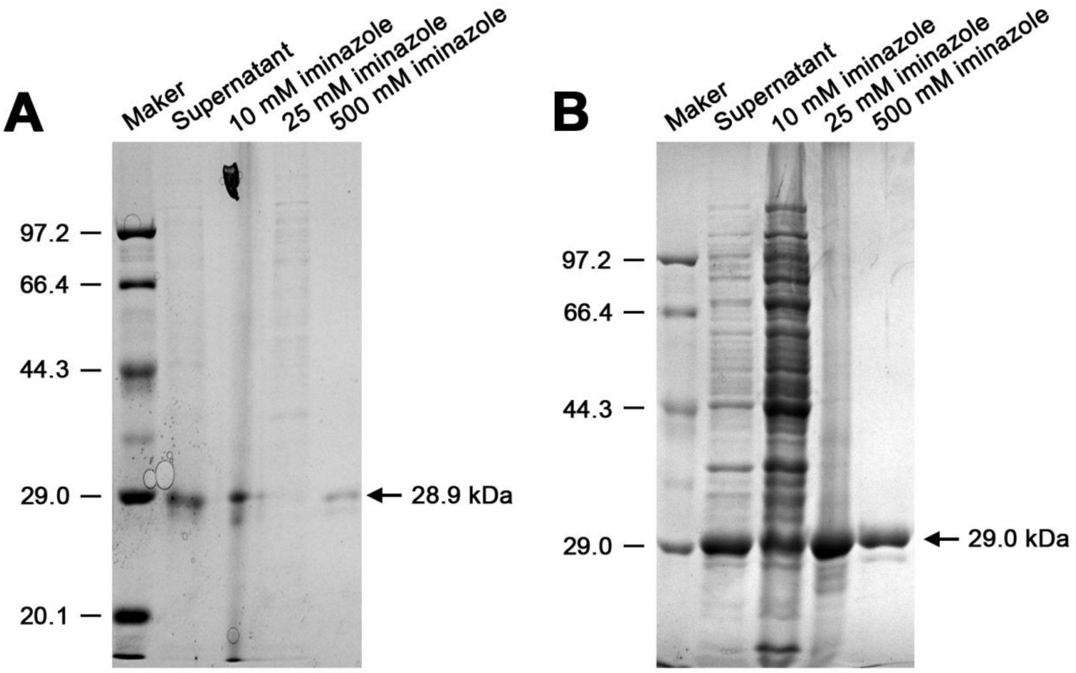

Supplement: Supplemental figures — Fig. S1 to S11. [file spectrum.02488-23-s0001.pdf]
