# Supplementary material for: Flexible changes to the Heliothis virescens ascovirus 3h (HvAV-3h) virion components affect pathogenicity against different host larvae species
Source: Microbiol Spectr. 2023 Nov 9;11(6):e02488-23. doi: 10.1128/spectrum.02488-23 (PMC10714839; doi:10.1128/spectrum.02488-23)
Supplement: Supplemental tables — Tables S1 to S28. [file spectrum.02488-23-s0002.docx]

**Table S1 Formula of artificial diet for maintaining insect larvae (1 L)**

|  | Component group A* | Component group B | Usage |
| --- | --- | --- | --- |
| Diet CSK | Cornmeal (75 g), soybean powder (75 g), yeast (10 g), distilled water (700 ml), agar (20 g) | ketchup (200 g), Sorbic acid (1 g), nipagin (2 g), vitamin C (2 g), cholesterol (0.1 g) | Laboratory maintaining of *Spodoptera exigua* larval populations. |
| Diet RW | Rabbit grain (160 g), wheat germ slice (150 g), yeast (10 g), distilled water (700 ml), agar (30 g) | Sorbic acid (1 g), nipagin (3.8 g),  vitamin C (8 g) | Laboratory maintaining larvae of *Helicoverpa armigera*, *Spodoptera frugiperda*, and *Spodoptera litura* larval populations. |
| Diet WS | Wheat germ slice (80 g), soybean powder (70 g), yeast (10 g), distilled water (1200 ml), agar (30 g) | Sorbic acid (0.9 g), nipagin (1.2 g),  vitamin C (3.5 g) | Laboratory maintaining of *Mythimna separata* larval populations. |

Note: The ingredients of the artificial diets were divided into two groups: group A provided the main nutrients for larval growth, and group B included preservatives and additives to prevent the diets from spoiling. To prepare the artificial diets, the powders in group A were weighed and mixed gently in distilled water at 37 °C, followed by fermentation in an incubator (Sigma-Aldrich, St Louis, MO, USA) at 37 °C for 20 min. The fermented group A was then transferred into the autoclave at 121 °C for 20 min for sterilization and inactivation. Distilled water was added to the sterilized group A to adjust the volume to 1 L. The weighed ingredients contained in group B were then added to the chilled group A (between 50–60 °C) and mixed thoroughly. The diets were transferred to a sterilized container and stored at 4 °C. The different diets were prepared separately.

*Cornmeal purchased from XINXIANG HANGYU CORN PROCESSING Co., LTD.CHN; wheat bran purchased from New hope LIUHE Co., LTD.CHN; Ketchup purchased from Lee Kum Kee (XINHUI) Food Co., LTD.CHN; nipagin, vitamin, cholesterol and sorbic acid purchased from Shanghai Macklin Biochemical Co., LTD.CHN; sucrose purchased from JIANGSU LONGCEHNG FINE CHEMICAL Co., LTD.CHN; soybean powder purchased from EASTOCEAN OLS GRAINS INOUSTRUES (ZHANGJIAGANG) Co., LTD.CHN; Wheat germ slices purchased from XINHUA HENGTAIYUAN FOOD Co., LTD.CHN; Rabbit food purchased from BEIJING KEAO XIELI FEED Co., LTD.CHN; Yeast purchased from Angel Yeast Co., LTD.CHN.

**Table S2 Primers used in this study**

| Primers | Primer sequence (5'→3')^a^ | Product size | Purposes |
| --- | --- | --- | --- |
| 3h-21-F | GGATCCATGTCAATATCCAGTGTATCCG | 687 bp | Amplification of *3h-21* |
| 3h-21-R | CTCGAGTCAGTCGGTTGGTAATCTACAC |  |  |
| 3h-38-F | GAATTCATGCAGTCGACTGATTTATTT | 1080 bp | Amplification of *3h-38* |
| 3h-38-R | CTCGAGTTATGAGTGATTTATTGCATTAT |  |  |
| 3h-43-F | GGATCCATGTTTAACGTAGATCAGG | 702 bp | Amplification of *3h-43* |
| 3h-43-R | CTCGAGCTATGCGCGAATCATAGAAC |  |  |
| 3h-53-F | GGATCCATGACTTCAAACACAGAAAC | 1368 bp | Amplification of *3h-53* |
| 3h-53-R | CTCGAGTTAATTGAAATCGCCTCCG |  |  |
| 3h-107-F | GGATCCATGATGGACCACGTATTTACG | 492 bp | Amplification of *3h-107* |
| 3h-107-R | AAGCTTTTACATATCTCCAAGTGTTCGG |  |  |
| 3h-117-F | GGATCCATGTTTGCGAAACCAGAGC | 570 bp | Amplification of *3h-117* |
| 3h-117-R | AAGCTTCTACGTATATCTATCGTGTAGGT |  |  |
| 3h-122-F | GGATCCATGGATTGCTCACTGGTG | 426 bp | Amplification of *3h-122* |
| 3h-122-R | AAGCTTTTACAACGGTTTAATAGAAGAT |  |  |
| 3h-135-F | GGATCCATGATTGCAAAATCTAATTCC | 378 bp | Amplification of *3h-135* |
| 3h-135-R | AAGCTTTCACTTATCAGAAGTTCTCTCG |  |  |
| 3h-171-F | GGATCCATGTCGTCCAGCTTTCAAT | 945 bp | Amplification of *3h-171* |
| 3h-171-R | CTCGAGTCACTTGACAGCCGTGCGT |  |  |
| q3h-MCP-F | AATCAAATCCGTACGCCTGAAGG | 139 bp | Absolute quantitative PCR detection of HvAV-3h viral DNA copies |
| q3h-MCP-R | TTCGCACCAATGTCAACGGAACG |  |  |
| Sf-gst-F | GGATCCATGCCGAAGTACGTGGTC |  | Amplification of *Sf-gst* |
| Sf-gst-R | AAGCTTGAATTCTGTAGGACCTACGG | 612bp |  |
| Sl-gst-F | GGATCCATGTCTGAGAAGCATTTACAAAC |  | Amplification of *Sl-gst* |
| Sl-gst-R | AAGCTTTTCCTTCTTCTTCCTTGGG | 762bp |  |

^a^Restriction enzyme sites are underlined (*Bam*H I, GGATCC; *Xho* I, CTCGAG; *Eco*R I, GAATTC; *Hind* III, AAGCTT).

**Table S3** Days within larval stage of treated and untreated larvae (days).

| Inset | CK | | | | HvAv-3h | | | | Log-Rank | | |
| --- | --- | --- | --- | --- | --- | --- | --- | --- | --- | --- | --- |
|  | N^a^ | ST_50_ (days)^b^ | Range  (days)^c^ | 95%CI (days)^d^ | N^a^ | ST_50_ (days)^b^ | Range  (days)^c^ | 95%CI  (days)^d^ | *χ^2^* | *d. f.* | *P* |
| *Helicoverpa*  *armigera* | 94 | 8.50 ± 0.12 | 7 ~ 13 | 8.30 ~ 8.76 | 92 | 15.39 ± 0.50 | 7 ~ 28 | 14.42 ~ 16.36 | 148.6 | 1 | <0.0001 |
| *Mythimna*  *separata* | 96 | 11.55 ± 0.15 | 8 ~ 16 | 11.26 ~ 11.85 | 96 | 14.54 ± 0.59 | 5 ~ 27 | 13.39 ~ 15.69 | 28.15 | 1 | <0.0001 |
| *Spodoptera*  *exigua* | 94 | 6.93 ± 0.11 | 5 ~ 10 | 6.71 ~ 7.14 | 66 | 7.89 ± 0.37 | 4 ~ 16 | 7.18 ~ 8.61 | 9.72 | 1 | 0.0018 |
| *Spodoptera*  *frugiperda* | 96 | 9.05 ± 0.13 | 4 ~ 12 | 8.81 ~ 9.30 | 91 | 13.66 ± 0.47 | 8 ~ 26 | 12.75 ~ 14.57 | 97.46 | 1 | <0.0001 |
| *Spodoptera*  *litura* | 96 | 9.34 ± 0.17 | 7 ~ 14 | 9.00 ~ 9.69 | 95 | 11.39 ± 0.25 | 4 ~ 17 | 10.91 ~ 11.87 | 45.07 | 1 | <0.0001 |

Note: ^a^total number of insects used for testing; ^b^median survival time, the asterisk indicates a significant difference was found between the two treatments; ^c^shortest and longest survival time; ^d^95% confidence interval for median survival time.

**Table S4 Days of the instar affected by HvAV-3h.**

| Insect | Stage | CK | | | | HvAV-3h | | | | Log Rank | | |
| --- | --- | --- | --- | --- | --- | --- | --- | --- | --- | --- | --- | --- |
|  |  | N^a^ | ST_50_ (days)^b^ | Range (days)^c^ | CI 95% (days)^d^ | N^a^ | ST_50_ (days)^b^ | Range (days)^c^ | CI 95% (days)^d^ | χ^2^ | *d.f.* | *P* |
| *H. armigera* | 3rd | 95 | 2.4±0.2 a | 1~7 | 2.1~2.8 | 90 | 2.4 ± 0.1 | 1 ~ 8 | 2.3 ~ 2.8 | 6.804 | 1 | 0.0784 |
|  | 4th | 95 | 2.1±0.1 b | 1~6 | 1.9~2.3 | 90 | 5.9 ± 0.5* | 1 ~ 23 | 4.8 ~ 6.9 | 103.2 | 1 | <0.0001 |
|  | 5th | 94 | 2.6±0.1 b | 1~5 | 2.4~2.9 | 68 | 10.3 ± 0.5* | 2 ~ 20 | 9.2 ~ 11.3 | 228.7 | 1 | <0.0001 |
|  | 6th | — |  |  |  | 5 | 4.4 ± 1.8 | 1 ~ 11 | 1.0 ~ 7.9 |  |  |  |
|  | Pre pupa | 93 | 2.9±0.1 | 1~6 | 2.8~3.1 | — |  |  |  |  |  |  |
| *M. separata* | 3rd | 143 | 2.3±0.1 a | 1~5 | 2.2~2.4 | 140 | 2.0 ± 0.1 | 1 ~ 5 | 1.9 ~ 2.2 | 35.54 | 1 | <0.0001 |
|  | 4th | 143 | 2.8±0.1 b | 2~6 | 2.7~2.9 | 137 | 4.5 ± 0.2* | 1 ~ 17 | 4.0 ~ 4.9 | 105.8 | 1 | <0.0001 |
|  | 5th | 142 | 5.5±0.1 b | 2~12 | 5.3~5.8 | 127 | 7.8 ± 0.4* | 1 ~ 21 | 7.0 ~ 8.5 | 98.20 | 1 | <0.0001 |
|  | 6th | — |  |  |  | 45 | 5.4 ± 0.7 | 1 ~ 18 | 4.0 ~ 6.9 | 19.10 | 1 | <0.0001 |
|  | Pre pupa | 115 | 3.8±0.2 | 1~8 | 3.4~4.1 | — |  |  |  |  |  |  |
| 1. *exigua* | 3rd | 96 | 2.7 ± 0.1 | 2 ~ 6 | 1.6 ~ 2.9 | 96 | 2.7 ± 0.1 | 1 ~ 6 | 2.7 ~ 3.0 | 1.666 | 1 | 0.6446 |
|  | 4th | 94 | 3.4 ± 0.1 | 2 ~ 7 | 3.3 ~ 3.6 | 82 | 5.2 ± 0.4* | 1 ~ 15 | 4.4 ~ 6.0 | 32.51 | 1 | <0.0001 |
|  | Pre pupa | 82 | 2.0 ± 0.1 | 1 ~ 4 | 1.9 ~ 2.2 | — |  |  |  |  |  |  |
| *S. frugiperda* | 3rd | 95 | 2.5±0.1 b | 1~4 | 2.4~2.7 | 91 | 3.0 ± 0.1 | 1 ~ 10 | 2.7 ~ 3.3 | 35.77 | 1 | <0.0001 |
|  | 4th | 95 | 2.5±0.1 b | 1~5 | 2.3~2.6 | 90 | 6.5 ± 0.4* | 2 ~ 21 | 5.6 ~ 7.3 | 136.9 | 1 | <0.0001 |
|  | 5th | 93 | 3.0±0.1 b | 1~5 | 2.8~3.1 | 55 | 7.9 ± 0.7* | 1 ~ 22 | 6.5 ~ 9.2 | 161.9 | 1 | <0.0001 |
|  | 6th | — |  |  |  | 4 | 7.8 ± 2.8 | 3 ~ 13 | 2.4 ~ 13.1 |  |  |  |
|  | Pre pupa | 93 | 2.1±0.1 | 1~5 | 1.9~2.4 | — |  |  |  |  |  |  |
| *S. litura* | 3rd | 96 | 2.1±0.0 b | 1~3 | 2.0~2.2 | 96 | 2.5 ± 0.1* | 1~4 | 2.3~2.6 | 50.10 | 1 | <0.0001 |
|  | 4th | 96 | 2.8±0.1 b | 2~4 | 2.7~2.9 | 95 | 5.2 ± 0.3* | 2 ~ 14 | 4.7 ~ 5.7 | 197.8 | 1 | <0.0001 |
|  | 5th | 96 | 3.7±0.1 b | 2~7 | 3.5~3.9 | 75 | 5.7 ± 0.3* | 1 ~ 12 | 5.0 ~ 6.3 | 221.2 | 1 | <0.0001 |
|  | Pre pupa | 88 | 1.9±0.2 | 1~7 | 1.6~2.2 | — |  |  |  |  |  |  |

Note: ^a^total number of insects used for testing; ^b^ median survival time, the asterisk indicates a significant difference was found between the two treatments; ^c^shortest and longest survival time; ^d^95% confidence interval for median survival time.

Table S5 Identified HvAV-3h coded proteins from *Mythimna separata* produced virions (Ms-1)

| Protein ID | Identified peptide sequence | Sequence coverage | Protein Q score | Unique spectra | Function annotation |
| --- | --- | --- | --- | --- | --- |
| 3H-56 | AHMFKPK;AHMFKPKQTLAR;DIHKFNGSK;DMNGIAVSVDAK;EEDGWDVATTEK;IISETVK;IISETVKIMNGAGAAGGAVVPK;IMNGAGAAGGAVVPK;IVKAHMFKPK;LKEEDGWDVATTEK;LQPAPLDIK;LQPAPLDIKGGRPMVR;LTNDEMNR;LTNDEMNRILR;LYPALMSPNTITNVIPDAR;LYPALMSPNTITNVIPDARNGVHSTDR;MELHFEFM;NGVHSTDR;QTLARDMNGIAVSVDAK;SSVMVIGLSEVFMQQTSVTFR;TIIGDLGSLQGPEGGEWLAFFYPAHTVFEK;TIIGDLGSLQGPEGGEWLAFFYPAHTVFEKMR;VGETPDLSSFSQEEISK;VGETPDLSSFSQEEISKLK | 0.8430 | 91.1210 | 131 | NA |
| 3H-55 | AYELANER;AYELANERGPFSVEVYLNPGTSNTYQYVATTR;DSYRLPQSFTR;FNNTDYDDLPWNYTSGK;FNNTDYDDLPWNYTSGKK;GIASLWGYLK;GPFSVEVYLNPGTSNTYQYVATTR;IGQTPKAYELANER;KVVTATGVVSGGER;LPQSFTR;MSLTER;NKFNNTDYDDLPWNYTSGK;SEIAQDIQVTMYNTNGGSNPLNNSNVTR;SNVDSSSYYQQPPQVVYNNGDLYGSR;TGYSGAELGASIDK;TGYSGAELGASIDKGIASLWGYLK;VVTATGVVSGGER;VVTATGVVSGGERYVFLLR;YVFLLR;YYYMSRPIGFGSSGAYDVPLLDTPLLR | 0.8844 | 78.7110 | 117 | NA |
| 3H-57 | AEMQINTQR;AKLDLVTINSVANK;ALLEVTDNVVTDR;ALLEVTDNVVTDRVPVR;AQLDVADLER;AQLQSDLDVLR;ATIDALTANLNSSNSK;EHDVLER;EKAQLQSDLDVLR;ELNTVR;EMWER;EMYAVLEDELK;GELNAMTR;GGMGVLDDIYNR;HENLYLASTTPSR;HIAGVITK;HVGNAQSDGMYTENVALR;IESEAALVESLQR;IQAFVNTEQNILAAGTR;ISELLTQLQESNNQR;ITNEFFDHATLPLLR;IVENMYK;KQMGNVVQEIAQR;LDGSDVTLVVDDENLK;LDLVTINSVANK;LIDSVVK;LLSLDGVR;LLSLDGVRR;LLVSEDK;LNDIAELQSQLTK;LNNELETSITEER;LQLDYMTLQQR;LTNECQAVSSR;MEFEAVNDALSK;MLELQNLMK;NELAATVNSLQMQATK;NIDGAEGFTR;NNVQLQTALQR;NSGVGNAINDR;NVNDTTLAELAQSR;QHAEFLR;QHPSFANKPNNTDVQQSDLTIAEDADTGTSVTK;QLNALNLR;QQLEWILVNSNPAIALQDASVR;QSLLETSAQLNDVR;RLNNELETSITEER;RNNVQLQTALQR;RPSVTDDEDDRIVGTR;RSLSYSLTDDEDDAAQIR;SETNALVVEVADLK;SLSYSLTDDEDDAAQIR;SNANLSPQSIR;SQALYAIK;STMLER;TKEMYAVLEDELK;TQLLTGLR;TYQQEMTQLK;TYQQEMTQLKR;VAYANLEGDMQK;VITGDETVMTYNR;VNRNSGVGNAINDR;VSTDDLTSYSR;YDNYNRNEHELR;YEALMSEQSSK;YESLMTK;YQAAGTPGNGR | 0.4778 | 230.1868 | 95 | chromosome segregation SMC like protein |
| 3H-50 | ACSAPSWTGIPSVNYNNQLMGASMFPNQPLA;ATHYDPIR;ATHYDPIRDGYYMYALPNGR;DGYYMYALPNGR;DGYYMYALPNGRVDR;DYFLSSK;FLKPTFWFSR;LPSDSGVAVTTSPTVVDDLDLR;LPSDSGVAVTTSPTVVDDLDLRIR;NALVALSNSSMNNEQNYTNR;NVYKDPAR;SGSTAAISYPR;YDNFQCNK;YKDDAR;YNNLSWLLSK;YNNLSWLLSKNVYK | 0.8360 | 58.9060 | 86 | NA |
| 3H-53 | ALFFGVR;DSGVALPIGMMVNNR;EIRPCAAFSK;ENVPDTGAGTGFMNMLTDGDFATPPEITK;GQYMYSFALDLK;GSINPSNFNSSISVVIEPSEALR;GTIDSYIYGPDEQVTTYFVR;GTIDSYIYGPDEQVTTYFVREIRPCAAFSK;KLSDLLIK;LSDLLIK;MPVLLSTGNGSNK;NITHSEYLDYYEVGLPK;RGTIDSYIYGPDEQVTTYFVR;SIGVSALSK;SVDIGAKTLVLPIPLYFSR;SYAAGTESSYFPK;TLVLPIPLYFSR;TSCSPHYILMEKPTDIAATEILKPTEAGSTLSYDIEHSSGILK;VPMLPAEHFVFTEPYR;VTVEFVFR;YRVPMLPAEHFVFTEPYR | 0.5824 | 82.0072 | 76 | major capsid protein |
| 3H-107 | ASLKTNTPLEGVPEFVLFHYGNAVK;FFPAETHVEVK;GILSIRR;LQLSDWNDNVTTIK;LQLSDWNDNVTTIKK;MMDHVFTLTPSDVTVR;NYVLNEPPAR;RGILSIR;RIDHVPSAVVK;RYASDSLMYFECDLSIR;SPYANAAR;SYSAYR;TNTPLEGVPEFVLFHYGNAVK;TNTPLEGVPEFVLFHYGNAVKTLDR;YASDSLMYFECDLSIR | 0.7975 | 53.9464 | 42 | thioredoxin-like protein |
| 3H-27 | ADNVWVYTEDLQAIK;ALINEDGHSVVTQR;CLLSITR;DDNSPSNDSVAMK;FNIDNVNYASVAHAIALISVVYHMK;FSVLNSPLSR;FTDEIIK;GVVWSEQSQQLVAAAATTK;HIHSVGDPSYNDEVTR;HITYVVVGHALVSHR;HSNVYSDALESFR;IVTSQLLYK;KADNVWVYTEDLQAIK;KVDEFISFGR;NLCEIGVVSNVTDVDLLR;RDPALYEVQLHTCR;RPDAEECFK;SLVLHFGANPAYTIMNTFASPAESPR;SLVVLTLQHELR;SLVVLTLQHELRK;SPPTLTIDDDSILYPLQYIPR;STEDDRPIQMQR;TGYANLK;TLFGDVVQK;VLTAALMHTDADTVNPTTPSVGYESDAVPR;VSDPQTTVR | 0.4506 | 90.0050 | 37 | NA |
| 3H-110 | ATDINPYETK;FLTNEIK;FLTNEIKADGTAIR;GWVPVNGSDAFEFSCK;HLNPQWATDAPILK;IFEQAGCK;IGFISQILPR;KATDINPYETK;KNVDDDTIELR;LGNVSGIITK;LSFDKK;LTFNGTAK;MAGLPLHAK;NVDDDTIELR;TMYAPLSK;TSNKPLIFECILDEPEK;TYKHLNPQWATDAPILK;TYVTLNR;VVLAPEK;VVLAPEKVYEFISR;VYEFISR | 0.7746 | 70.0426 | 35 | NA |
| 3H-48 | ALLLSQLVSPPLIAK;AMHLLYIEGK;ATQNTVQTLR;CSSTAAVDAAVASSSTEALSQK;GSSISTAVSK;LQSWVTDHEAVQYGTKPTVPYVIPDNLLSQKPTDNTISR;LYGYMANK;NDITAKNITSK;NEIIAKNDITAK;SVASVSSPGNIASSSVGNK;SVASVSSPGNIASSSVGNKR;TAQSIMQNLMTQIK;THIFNYLDSK;TVNVNQDSQTIK;TVNVNQDSQTIKTHIFNYLDSK | 0.3848 | 59.1136 | 34 | NA |
| 3H-137 | AMSNGLGDVILSAK;DTMAMIFK;IFDAFKR;KIFDAFK;NALLTLK;RTGVDLSVNVGQTYK;SEDEEVSAEIVTAK;TGVDLSVNVGQTYK;VSGDVIDKVSTTVDPMMPAQVK;VSTTVDPMMPAQVK;YSVAVAK | 0.5875 | 40.9255 | 31 | hemolysin-like protein |
| 3H-156 | DCYVDHSAGVPTYFVVER;DLFNFVSQTR;DTGHLVAFINGGTR;IVQFPPGSPYTFVATR;QTVLAQSNSGAYPVER;RPAALPPR;RQTVLAQSNSGAYPVER;TSDDVPYEATVNTANMYDGDASGR;VDVQSCMNIFTTTGPSR | 0.3229 | 38.0794 | 29 | NA |
| 3H-84 | ASIQSGYNK;ELMQYIR;LLSPPVDPHVFIDTTAASIDK;NMVINEPQYQQFLR;NSEEDSSPAQADQSESVGSSPPTK;SIDTNEGNDDDSTAIALR;SPHLDMAFR;TGTEPKPEHSDDGKNSEEDSSPAQADQSESVGSSPPTK;TMEFVR;VVAKPQYDDR;VVAKPQYDDRELMQYIR | 0.5714 | 41.2603 | 28 | ABC-type transport system permease |
| 3H-151 | ALQYQTQLEGLLSR;CKPGEVMDDK;CKPGEVMDDKSSTTEER;ETCALTTVTASLATISESLGK;ETCALTTVTASLATISESLGKDEALLK;ITEVGGGVAAIK;ITEVGGGVAAIKSSLTSVVFTPLSEK;LDAIKSDISGK;LVQIDNTVK;SSLTSVVFTPLSEK;SSLTSVVFTPLSEKLDAIK;VAFGLTR;VEKALQYQTQLEGLLSR;VVVDPLDTK;VVVDPLDTKVEK;VVVVPVQDK;VVVVPVQDKITEVGGGVAAIK | 0.6502 | 64.8613 | 27 | NA |
| 3H-13 | AAVWLATTTGDDSGYR;DIISPLILLDAER;DVIHEHVVAHWNDAETEQIIGR;ESPTEYFLR;EVMTVPSIVSALK;EWNATFVVIDEAHDLSVHGVDYPK;GANVAIILGSR;GLVLTPNR;HLNDFEIDR;KVLLLTATPMR;LFDYR;LSTHDITVEQFNR;NIDGALR;NITNDNTIPLDAPR;NNLSDLVPLHNLLMK;SPIDGIIVIHGVGTGK;TLTAILAMINNITAGHDQGMK;VIAEGITLK;VLLLTATPMR;YALPLYPIAESTDESWSYEHLDK;YQSHAYTIADWGSDAKPNVFVYR | 0.2987 | 76.0557 | 26 | DEAD-like helicase |
| 3H-66 | DIIMGHISPNR;DTFAVSPAR;IFLATNTNSHVR;ISTIPEFHTKPK;KVQVDYSPNYDLTVVTVNDHVSTTK;QAIVQNGR;RSLAHEHEEEEVFNDNEK;SIFSSPIPNLGFVAAENGHVR;SLAHEHEEEEVFNDNEK;SLAHEHEEEEVFNDNEKYDDER;SPASDQSYYGESNDGSYK;SPASDQSYYGESNDGSYKR;TADGDYDDDER;VQVDYSPNYDLTVVTVNDHVSTTK;VTVHEVNADLYLCKPR | 0.4514 | 55.8578 | 25 | NA |
| 3H-117 | CIPVVAYHAQDADAPTDTGLK;DDAIIIDDLPDVYITQPHR;DYAAVITAELILR;LDSAESEMAR;LEMFLSSDETR;MFAKPEPAIR;NVNGFVPAVIFSDYEVYK;RLEMFLSSDETR;RPHLDEFLSYLFENFR;SRPTVFLDLDNTLICSLR;TAGEEVGGVK;VGVWTAASK | 0.8254 | 44.6285 | 23 | CDT phosphatase transcription factor |
| 3H-19 | ESTTNIAEDIER;ESTTNIAEDIEREPAYLK;IFSMLDNEIHLTIDSSDHQHVLQIYNR;ILITTYDYSGVQQR;ISPMYSFHSR;KNTNLYGVTR;LLYWLEDNRFDLLTVDGNDAR;MRPTTADVDNAQPTITVDDLK;NEQPQNSSSFDK;QIGNGEDANANVEINCGHR;QTLGLVIK;SETTDASNVSPR;SHVDDNEDLAPSKR;SYADELGDR;TPSPTAVEVNTLAR;TVLDAAR;VLPQHIR;WSYNSLTDGGAFAITGILAR | 0.3762 | 63.2248 | 22 | NA |
| 3H-71 | LGKPIVVIAPVR;LGKPIVVIAPVRR;LSESVAVIEAK;LSESVAVIEAKR;NEVAWLLR;NLFTNIR;RLGKPIVVIAPVR;SDSLDACLTFIDIVLDSHR;SRNEVAWLLR;VQFPHVVHNPELNVIYWR;YCADIFDIVQHAASL | 0.4567 | 38.9152 | 22 | sulfhydry1 oxidase Erv1 like protein |
| 3H-139 | AHIAPALLNHIESNLLK;ATLDEVSNDPDDIDVER;ATLDEVSNDPDDIDVERR;FVFSARPTVDSSLASGGVR;HHISPEMYVTMVK;LAGYLKQSFK;QTTVDKWIDSR;VAAYEIAR | 0.3466 | 30.7075 | 21 | putative S1/P1 nuclease |
| 3H-15 | DGTVVISEDDKHNEPYGIGK;FFNFKEDFTR;IIPNNESADSR;KTIEALISLPR;LKDPPHTDALK;SKYPALVKPYEEYQR;SNVHIKPVNPPSSK;TIEALISLPR;TIVLPTNR;TLIPLQKPIAPVQISEKPVVVK;VGYHTSDLER;VIQTPDPQPVVEGK;VIVNNPR;YYVDDLVK;YYVDDLVKDDALMR | 0.5623 | 56.6282 | 20 | NA |
| 3H-62 | ATLEQVFVR;AYVDLK;AYVDLKDFVVTHENLFAR;DFVVTHENLFAR;INTGSITDSVQCFNK;LDNNFVDGLK;LITDPYQLLQISER;RLFVPGIDLPQPYIGIDTTK;TQTPVNILADIVSLK;VCINVNAMTAGDK;YTTSTVPNETDRR | 0.4315 | 42.5520 | 19 | myristylated membrane protein-like protein |
| 3H-135 | ALNMILK;ALNMILKR;DQVPVLTK;EFPNISCR;KRPIQPTAYNLFYK;RPIQPTAYNLFYK;RPIQPTAYNLFYKDQVPVLTK;SNSNNTMTALDGVHQINSLVTSVFSDSEHINEWLSLSK;WNYHKQYNPK;YLLDNYNYR | 0.7600 | 33.2656 | 18 | yabby-like transcription factor |
| 3H-28 | GIDDGDAIYTITK;IFMLVLLER;ITALYHNVK;LTEWFPNNR;NITLSPQYCSEYLTLK;NNLNYDQLYTALR;NSLSGQSSSMGTGTLSMDQVK;NVAEYQSR;QHLLDER;TAYDQADVIAYIDGDKEK;YNGLMHFISK | 0.3757 | 37.5072 | 18 | NA |
| 3H-43 | DFNVASYESDPLLK;DLYNLQILR;DQSLELLSHTHR;FGLASVNDENSYK;FVEFGTLVRPAGWGSLSELGK;KDFNVASYESDPLLK;LSLGYQTVVQFYR;LSYDSVVDWER;LTIDER;MFNVDQAIEFFENR;SSPQTVGLEWWK;VCHLNAVLDPAFTIDTGNVR;YNSSMDEYRDQSLELLSHTHR | 0.6652 | 53.2924 | 17 | NA |
| 3H-37 | DDNDQLWMLANPFAR;EFFTLNEDNFTELIK;IDALDDFEVWR;ILEYSNAPNAIAK;LHSQVTNLHPLSK;LSELNVASAYDFRPVFVVPTK;LSELNVASAYDFRPVFVVPTKK;MCSDFLISNK;QLEAVLHTK;QTLEEQLPR;REFFTLNEDNFTELIK | 0.4097 | 39.7151 | 16 | bro4 |
| 3H-64 | AVIGSSGSVNNTVNTVHSR;FLGLSGIFSMLADIGNTNK;GGGNSSHIENVIMIPHK;LPDVGGVNDVISSK;NIILSGEHDR;QIVDEDDGR;SDVIAAKPNYK;SIESLLGIPSPSKPQSK;SQWADDYSGR;SVTSDVAALAHSETSR;VMLVEYYALLK;VNQMYR | 0.3724 | 46.6909 | 16 | NA |
| 3H-93 | ALEAAGSVVVPVHVGR;DEAYYSATMR;DNFPAAIVSNR;FKDDYGQYEMFR;HAPSSVIADFENLPIAIQANNHCINER;SAYEWYESR;SINGLLLPGGK;SLEEDYK;SYVVASYVK;YFYDYFVQK;YRDNFPAAIVSNR | 0.3942 | 42.5993 | 16 | gamma_glutamyl hydrolase-like protein |
| 3H-182 | APQQIATVK;CDDGELTLHR;HTGGLIITAPLVHPAVGTTR;RHTGGLIITAPLVHPAVGTTR;SITDMIAAGAPISK;SITLQNTVTK;TQHHNEEVETVDQSILAHVR;VFECPNTEEVPNFR | 0.2481 | 33.8483 | 15 | NA |
| 3H-116 | CLYIDYQGGK;DLPLTLR;EHYTLLNTPSATK;ETFYQVFSQVTSVDHR;IAFVGKPGTGK;IQEDLFKNGR;LWEFADDFPNK;MIDDSLIIPQFHHNLISPR;TCLDGIFLFGTGNHDVR;YIMYTK | 0.3371 | 36.4371 | 14 | ATPase involved in DNA metabolism |
| 3H-150 | CLVDLGR;FHNCPNDLTTQIAR;LAVEYNGR;LQVIFDDNDEKR;MNGYSGTWQPLR;NIDLIDVPYTIPIDEIPNFIDK;QGVNTLAEYGK;QHYEYVPR;SADDALLDPTTSPSPVFAR;VRPDWLINPNTK | 0.5365 | 33.5949 | 14 | Uyr/REP helicase |
| 3H-46 | HSDDIDTTIDALVK;ISKQLTQLR;KLDQEVLIQEMR;QLTQLR;YMEVSSNDTLLYDNVR | 0.1984 | 18.7954 | 14 | NA |
| 3H-152 | ASYMNLLIVEQDR;ELLNPEHSAK;FIYEYNNYTVR;LIGMDGVQLETIR;LVYNASLLLR;RVDDAELER;SEELDILK;SLVIGAEQLER;SQTFYNTQR;TSPTLILHVWR;TVSLFRPPLSLK;VLPLTAFGK | 0.1002 | 39.9777 | 13 | Dynein-like β chain |
| 3H-58 | AVSPLTNKPIDVHGR;HIVVSNGFQPVR;KLIGTSPIR;LGGLSPYR;LTAAQCETFIR;NDQNVPTYYTSSRPFADR;NESINPITGK;SQLINIANNMNIAELR;TAANADTRPDLCATFSR;VAQNTTQSQLLNIVK | 0.2137 | 37.7358 | 13 | cofactor of the virion S/T kinase encoded by thioredoxin |
| 3H-82 | DAAYATDAR;GGHGSVGEVHTSAVVR;GVHCDNVFDTPK;LGYMAAGYESTYESVVR;RTVSAVGSNEELANTDDSSR;TLAMDNR;TLIIQSYSHAIVGDAFIQDR;TVSAVGSNEELANTDDSSR;VCAAFQIKPR;YDGEALTR | 0.3639 | 35.5915 | 13 | NA |
| 3H-88 | ASFDTVPWVDGR;FNDILK;FVSNTQQLVENSLIYGGLR;ITPVTFMTTVER;NCHTNAASALEAR;NIIPTPLLHHFER;QISHVVYADWLTER;SEMSMWLNGR | 0.3808 | 31.0958 | 13 | putative S1/P1 nuclease |
| 3H-109 | ALIVKPMNQFR;EVHVSVTTLMK;IAQYPINTK;ILSLDAVSVPIDQK;QAVSSSGCGGGSTYR;SQLHFETHESK;SVIPTNATTNAYNAVK;VALIMSIDAVIR;VGGAYFVTVR;YLDSLIPTPR | 0.2895 | 34.8586 | 12 | cathepsin B |
| 3H-118 | EDVIHDR;LDSFVGPTGNFHGMNVTMK;LNGQLHTLVIPK;QVGKPPICLGSDDVLR;RYAYTDSDGVYVK;SIVAAFATADLPTALYHSETATYR;YAYTDSDGVYVK | 0.5417 | 28.1669 | 11 | NA |
| 3H-34 | ECDWLLR;ECLDTVK;NNSEVLLHLYK;QLIPMSLYGHR;RPPGTLLWNAYVR;VFFEGCSFTDYVK;YDEESYCPIETDLAHK | 0.2583 | 24.9345 | 11 | NA |
| 3H-77 | MAFVHLFHIDGILECAER;VAALKQWATDELK;VHGWWWR;VQKVHGWWWR | 0.494 | 15.8828 | 11 | NA |
| 3H-26 | ANSIIDNIER;LYALKHWSTANSK;QIVDPNATMK;SGATYAINNSYTIDALR;TSEIVDVVDATR;TSEIVDVVDATRK | 0.4701 | 22.2233 | 10 | NA |
| 3H-63 | FLFLDEANKPLHEIAVQFTCK;GSVSDLEFK;HYTGIIPETMNVAK;KNVGLFIPK;LWNASVDSFCYR;SGDEIDGYIIMKPK;TAFSIVNK;VNAEDDCGVEAK;YLWTFAPLLEQR | 0.3688 | 32.8558 | 10 | NA |
| 3H-76 | DLEGIVMR;EFPLVNPAR;ELYPEHVK;FNEYDATLR;HISGGVEIDLNKK;IAMHGDVSR;LRELYPEHVK;LVLGSQSPYNAYVLNYGSAENQLR;SLWSTDETLPR;YPFTNGYGAYVVEK | 0.2649 | 35.8598 | 10 | NA |
| 3H-168 | HDDELHFAWQYVDYR;LGMAVTHFER;LNSEISSLYDEVAR;NHIQYQIDSISK;RHDDELHFAWQYVDYR | 0.3041 | 20.1138 | 9 | NA |
| 3H-69 | AAILLGASLVLNK;DESHYMLYTK;DYDTGKDESHYMLYTK;LVYKDYDTGK | 0.4231 | 16.9242 | 9 | NA |
| 3H-21 | FKEVEDLVLR;FLSDHPHMTK;IASLLVHDIHSSLDDAVK;IGSIAPIELNPPPR | 0.2281 | 15.4738 | 8 | NA |
| 3H-29 | ISPIFDGSVAILHDLSSGLVAAAISK;NHGTSVSIVGDDHK;TIADPMLDR;VFNDINVVK | 0.5918 | 13.9198 | 8 | NA |
| 3H-3 | DYFGWDK;DYFGWDKLEAAYNAK;IIQIAPVDLIK;KDYFGWDK;TLLKPTVHLNR;YANVNGFEGEFNSQYSVQSYNK;YGTIER | 0.3235 | 24.6827 | 8 | NA |
| 3H-79 | FPFWFER;NEPVSYLAVGNAQFIDR;RNEPVSYLAVGNAQFIDR;SPNCALVFTR;TNPELNDIER;VPVSNTVEFVIVILK | 0.48 | 20.4027 | 7 | NA |
| 3H-104 | ESYDALSVPSHWR;FVSVVGVYALIVHSDDATR;LWGHLMSMLK;YMWNDEDSEVQILQIQR | 0.3782 | 13.7833 | 6 | bro12 |
| 3H-113 | AFMELIR;DVEPSPTMVPK;EWFVQK;LYNTAFTMATSAVLAPATLTAR;RLDIAFSGTDK;SMLIDDLNSGFDVVIPPDRR | 0.2232 | 19.3050 | 6 | lysophospholipid acetyltransferase |
| 3H-157 | DLPDTVPVSLDVR;EQITQWLR;GINDLSDAELAYK;NPPLELWSR;NSTMNLPR;VLIDTEQKR | 0.3468 | 24.3449 | 6 | tyrosyl-DNA phosphodiesterase |
| 3H-81 | DTESVEFGYDENDKLR;HASWLFSYLQK;IITNNLMADMK;LTMYHTALFR;VITDNIVGTLNLLSR | 0.2582 | 17.5605 | 6 | NA |
| 3H-83 | AVTVTKGIAR;ESFGTYMGQR;KPAQELIHEYR;MSPELQEAWR | 0.2697 | 15.8828 | 6 | glycosyltransferase |
| 3H-181 | HIPSADDPGADLFTNWFSINAHDR;WYHLDCFLSK | 0.3063 | 6.4213 | 5 | NA |
| 3H-8 | DGYVQGIAAVYYAPHTSAATDQAFK;LNAQDVAVLGTSNPILR;TLSVYESAFPMR;VYIPVPPITR;YTMYPYVK | 0.1655 | 16.3619 | 5 | NA |
| 3H-128 | IATNTDLK;QTSSSQQRPNDLLYPLSYGAVDETVVLK;YADIPVWEQGVEDEPTSETNDTLNR | 0.2276 | 11.6517 | 4 | NA |
| 3H-134 | DSILQPDTLAK;IITHEEYVNLEADFESYKK;SVTSFAAFTDNDHEPPPK;VATYPPPTSNDESASHER | 0.3014 | 15.8828 | 4 | NA |
| 3H-16 | LGNVLFWNDLLK;LNHGHHLPLDK;QLADGLGFILSK | 0.0534 | 11.6517 | 4 | lipase like protein |
| 3H-171 | LGFQEFVYR;LKDFLTLFDVDELR;MFVVQTNR;VIDTFSK | 0.1210 | 15.3702 | 4 | caspase-like protein |
| 3H-172 | AGLHELTLNAASK;EGQVYVITTSK;FANDFR;SQVQVNALFTNR | 0.1826 | 12.9971 | 4 | bro23 |
| 3H-22 | DSHEIYINAWK;HASNDALNAIVYR;YLVVDSAGLQK | 0.1786 | 11.1392 | 4 | NA |
| 3H-42 | EITLYEHAK;SVGECIGGDPVANFK;VGVCNNLPENWQMR;YGTLSGIPALFR | 0.2137 | 16.9242 | 4 | NA |
| 3H-44 | DNSEASSQNTTVWDR;DNSEASSQNTTVWDRR | 0.1584 | 8.4621 | 4 | NA |
| 3H-70 | CEYVTK;YSGAGKPVSPNDFLMR | 0.2619 | 6.4213 | 4 | NA |
| 3H-72 | EFEYFK;ELQPPYSNYFEESLKPEAFVSNIHYK;QEYEDDDYNFK | 0.2774 | 11.1392 | 4 | phenylalanyl-Trna synthetase α subunit |

Table S6 Identified HvAV-3h coded proteins from *Mythimna separata* produced virions (Ms-2)

| Protein ID | Identified peptide sequence | Sequence coverage | Protein Q score | Unique spectra | Function annotation |
| --- | --- | --- | --- | --- | --- |
| 3H-56 | AHMFKPKQTLAR;DMNGIAVSVDAK;EEDGWDVATTEK;IISETVK;IISETVKIMNGAGAAGGAVVPK;IMNGAGAAGGAVVPK;IVKAHMFKPK;LKEEDGWDVATTEK;LQPAPLDIK;LQPAPLDIKGGRPMVR;LTNDEMNR;LTNDEMNRILR;LYPALMSPNTITNVIPDAR;LYPALMSPNTITNVIPDARNGVHSTDR;MELHFEFM;MTSVVLNFSSPLTGEYVLQMLNALNPTK;NGVHSTDR;SSVMVIGLSEVFMQQTSVTFR;TIIGDLGSLQGPEGGEWLAFFYPAHTVFEK;TIIGDLGSLQGPEGGEWLAFFYPAHTVFEKMR;VGETPDLSSFSQEEISK;VGETPDLSSFSQEEISKLK | 0.9215 | 86.0025 | 139 | NA |
| 3H-55 | AYELANER;AYELANERGPFSVEVYLNPGTSNTYQYVATTR;DSYRLPQSFTR;FNNTDYDDLPWNYTSGK;FNNTDYDDLPWNYTSGKK;GIASLWGYLK;GPFSVEVYLNPGTSNTYQYVATTR;IGQTPKAYELANER;KVVTATGVVSGGER;LPQSFTR;NKFNNTDYDDLPWNYTSGK;SEIAQDIQVTMYNTNGGSNPLNNSNVTR;SNVDSSSYYQQPPQVVYNNGDLYGSR;TGYSGAELGASIDK;TGYSGAELGASIDKGIASLWGYLK;VVTATGVVSGGER;VVTATGVVSGGERYVFLLR;YVFLLR;YYYMSRPIGFGSSGAYDVPLLDTPLLR | 0.8578 | 76.2987 | 115 | NA |
| 3H-57 | AKLDLVTINSVANK;ALLEVTDNVVTDR;ALLEVTDNVVTDRVPVR;AQLDVADLER;DEAIWAIK;ELACNVIPEK;ELNTVR;EMWER;EMYAVLEDELK;GELNAMTR;GGMGVLDDIYNR;HIAGVITK;HVGNAQSDGMYTENVALR;IESEAALVESLQR;IQAFVNTEQNILAAGTR;ISELLTQLQESNNQR;ITNEFFDHATLPLLR;KQMGNVVQEIAQR;LDGSDVTLVVDDENLK;LDLVTINSVANK;LLSLDGVR;LLSLDGVRR;LLVSEDK;LNDIAELQSQLTK;LNNELETSITEER;LQLDYMTLQQR;LTNECQAVSSR;MEFEAVNDALSK;MLELQNLMK;MTAAHLADDR;MVSTDDLTSYSRGELNAMTR;NAEITNLQDR;NELAATVNSLQMQATK;NIDELR;NIDGAEGFTR;NISAIRPR;NNVQLQTALQR;NSGVGNAINDR;NVNDTTLAELAQSR;QHPSFANKPNNTDVQQSDLTIAEDADTGTSVTK;QIDSLR;QMGNVVQEIAQR;QQLEWILVNSNPAIALQDASVR;QSLLETSAQLNDVR;RLLVSEDK;RLNNELETSITEER;RNNVQLQTALQR;RPSVTDDEDDRIVGTR;SETNALVVEVADLK;SLSYSLTDDEDDAAQIR;SNANLSPQSIR;TKEMYAVLEDELK;TQLLTGLR;TYQQEMTQLK;VAYANLEGDMQK;VITGDETVMTYNR;VSTDDLTSYSR;YDNYNRNEHELR;YEALMSEQSSK | 0.4326 | 212.3042 | 94 | chromosome segregation SMC like protein |
| 3H-50 | ACSAPSWTGIPSVNYNNQLMGASMFPNQPLA;ASCGTIPDAR;ATHYDPIRDGYYMYALPNGR;DGYYMYALPNGR;DGYYMYALPNGRVDR;DYFLSSK;FLKPTFWFSR;LPSDSGVAVTTSPTVVDDLDLR;LPSDSGVAVTTSPTVVDDLDLRIR;NALVALSNSSMNNEQNYTNR;YDNFQCNK;YNNLSWLLSK;YNNLSWLLSKNVYK | 0.7778 | 50.1576 | 89 | NA |
| 3H-53 | ALFFGVR;DSGVALPIGMMVNNR;EIRPCAAFSK;ENVPDTGAGTGFMNMLTDGDFATPPEITK;GQYMYSFALDLK;GSINPSNFNSSISVVIEPSEALR;GTIDSYIYGPDEQVTTYFVR;GTIDSYIYGPDEQVTTYFVREIRPCAAFSK;KLSDLLIK;LSDLLIK;MPVLLSTGNGSNK;NITHSEYLDYYEVGLPK;RGTIDSYIYGPDEQVTTYFVR;SIGVSALSK;SIGVSALSKIGIK;SVDIGAKTLVLPIPLYFSR;SYAAGTESSYFPK;TLVLPIPLYFSR;TSCSPHYILMEKPTDIAATEILKPTEAGSTLSYDIEHSSGILK;VPMLPAEHFVFTEPYR;VTVEFVFR;VTVEFVFRK;YRVPMLPAEHFVFTEPYR | 0.5912 | 90.5032 | 73 | major capsid protein |
| 3H-140 | ALSHPDWLTIGSFIK;DVDGVASNGGGSVGTGLGGPLGGTSGVNTSNLR;ELFYHSEAGR;ETNYPIPYFQYNLVLSQR;EVAKELFYHSEAGR;MLYGNSK;NSDKDVTIPK;SGDEMTLSR;STGTTVGYTGTTSNAAMK;STGTTVGYTGTTSNAAMKDVDGVASNGGGSVGTGLGGPLGGTSGVNTSNLR;TATHLLGYK;TATHLLGYKNK;TDVNATNKSTGTTVGYTGTTSNAAMK;TLDRPTDNAIMQR;YTYMYVNK;YTYMYVNKR | 0.7783 | 64.4407 | 63 | NA |
| 3H-48 | ALLLSQLVSPPLIAK;AMHLLYIEGK;AMHLLYIEGKYLR;ATQNTVQTLR;CLGQVVTNVR;CSSTAAVDAAVASSSTEALSQK;LKNWSPK;LQSWVTDHEAVQYGTKPTVPYVIPDNLLSQKPTDNTISR;LYGYMANK;NDITAKNITSK;NEIIAKNDITAK;SVASVSSPGNIASSSVGNK;SVASVSSPGNIASSSVGNKR;TAQSIMQNLMTQIK;THIFNYLDSK;TVNVNQDSQTIK;TVNVNQDSQTIKTHIFNYLDSK;VDQEVYMK | 0.4218 | 71.6689 | 43 | NA |
| 3H-107 | ASLKTNTPLEGVPEFVLFHYGNAVK;FFPAETHVEVK;IDHVPSAVVK;LQLSDWNDNVTTIK;LQLSDWNDNVTTIKK;MMDHVFTLTPSDVTVR;NYVLNEPPAR;RGILSIR;RIDHVPSAVVK;TNTPLEGVPEFVLFHYGNAVK;TNTPLEGVPEFVLFHYGNAVKTLDR;YASDSLMYFECDLSIR | 0.7055 | 44.1711 | 36 | thioredoxin-like protein |
| 3H-27 | ALINEDGHSVVTQR;CLLSITR;DDNSPSNDSVAMK;DSANSLLK;FNIDNVNYASVAHAIALISVVYHMK;FTDEIIK;GVVWSEQSQQLVAAAATTK;HIHSVGDPSYNDEVTR;HITYVVVGHALVSHR;HSNVYSDALESFR;IVTSQLLYK;KVDEFISFGR;NAFTSIIWNVDIFK;NLCEIGVVSNVTDVDLLR;SLVLHFGANPAYTIMNTFASPAESPR;SLVVLTLQHELR;SLVVLTLQHELRK;SPPTLTIDDDSILYPLQYIPR;STEDDRPIQMQR;TGYANLK;TLFGDVVQK;VDEFISFGR;VLTAALMHTDADTVNPTTPSVGYESDAVPR;VSDPQTTVR;YAIDVNILPAISDTIQK | 0.4374 | 87.8825 | 36 | NA |
| 3H-110 | ATDINPYETK;FLTNEIK;FLTNEIKADGTAIR;GWVPVNGSDAFEFSCK;HLNPQWATDAPILK;IGFISQILPR;KATDINPYETK;LGNVSGIITK;LSFDKK;LTFNGTAK;MAGLPLHAK;NVDDDTIELR;TMYAPLSK;TYKHLNPQWATDAPILK;TYVTLNR;VVLAPEK;VVLAPEKVYEFISR;VYEFISR | 0.6573 | 59.1313 | 35 | NA |
| 3H-137 | AMSNGLGDVILSAK;DTMAMIFK;IFDAFKR;KIFDAFK;KVSGDVIDK;NALLTLK;RTGVDLSVNVGQTYK;SEDEEVSAEIVTAK;TGVDLSVNVGQTYK;VSGDVIDK;VSTTVDPMMPAQVK;YSVAVAK | 0.5938 | 43.0913 | 31 | hemolysin-like protein |
| 3H-66 | CIADLR;DIIMGHISPNR;DTFAVSPAR;IFLATNTNSHVR;ISTIPEFHTKPK;KVQVDYSPNYDLTVVTVNDHVSTTK;QAIVQNGR;RSLAHEHEEEEVFNDNEK;SIFSSPIPNLGFVAAENGHVR;SLAHEHEEEEVFNDNEK;SLAHEHEEEEVFNDNEKYDDER;SPASDQSYYGESNDGSYK;SPASDQSYYGESNDGSYKR;TADGDYDDDER;VQVDYSPNYDLTVVTVNDHVSTTK;VTVHEVNADLYLCKPR | 0.4676 | 60.4490 | 30 | NA |
| 3H-135 | ALNMILK;DQVPVLTK;EFPNISCR;KRPIQPTAYNLFYK;QYNPKYLLDNYNYR;RPIQPTAYNLFYK;RPIQPTAYNLFYKDQVPVLTK;SNSNNTMTALDGVHQINSLVTSVFSDSEHINEWLSLSK;YLLDNYNYR | 0.7120 | 33.7854 | 28 | yabby-like transcription factor |
| 3H-156 | DCYVDHSAGVPTYFVVER;DLFNFVSQTR;DTGHLVAFINGGTR;FYDSSTSTCPINYR;IVQFPPGSPYTFVATR;QTVLAQSNSGAYPVER;RPAALPPR;RQTVLAQSNSGAYPVER;TSDDVPYEATVNTANMYDGDASGR;VDVQSCMNIFTTTGPSR | 0.3594 | 37.8829 | 28 | NA |
| 3H-117 | CIPVVAYHAQDADAPTDTGLK;DDAIIIDDLPDVYITQPHR;DYAAVITAELILR;LDSAESEMAR;LEMFLSSDETR;MFAKPEPAIR;NLNALWDLWGADVIIER;NVNGFVPAVIFSDYEVYK;RLEMFLSSDETR;RPHLDEFLSYLFENFR;SRPTVFLDLDNTLICSLR;TAGEEVGGVK;VGVWTAASK | 0.9153 | 49.5300 | 27 | CDT phosphatase transcription factor |
| 3H-84 | ASIQSGYNK;ELMQYIR;LLSPPVDPHVFIDTTAASIDK;NMVINEPQYQQFLR;NSEEDSSPAQADQSESVGSSPPTK;SIDTNEGNDDDSTAIALR;SPHLDMAFR;TGTEPKPEHSDDGKNSEEDSSPAQADQSESVGSSPPTK;VVAKPQYDDR;VVAKPQYDDRELMQYIR | 0.5455 | 39.7548 | 25 | ABC-type transport system permease |
| 3H-151 | ALQYQTQLEGLLSR;ETCALTTVTASLATISESLGK;ETCALTTVTASLATISESLGKDEALLK;ITEVGGGVAAIK;ITEVGGGVAAIKSSLTSVVFTPLSEK;LDAIKSDISGK;LVQIDNTVK;SSLTSVVFTPLSEK;SSLTSVVFTPLSEKLDAIK;VAFGLTR;VEKALQYQTQLEGLLSR;VVVDPLDTK;VVVDPLDTKVEK;VVVVPVQDKITEVGGGVAAIK | 0.5665 | 53.6997 | 24 | Uyr/REP helicase |
| 3H-19 | ESTTNIAEDIER;ESTTNIAEDIEREPAYLK;GDDVRPELSTFVLSYTR;IFSMLDNEIHLTIDSSDHQHVLQIYNR;ISPMYSFHSR;LLYWLEDNR;LLYWLEDNRFDLLTVDGNDAR;MRPTTADVDNAQPTITVDDLK;NEQPQNSSSFDK;NTNLYGVTR;QTLGLVIK;SETTDASNVSPR;SHVDDNEDLAPSK;SSASQSIGDIDK;SYADELGDR;VEEILK;VLPQHIR;WSYNSLTDGGAFAITGILAR | 0.3437 | 62.5408 | 24 | NA |
| 3H-13 | AAVWLATTTGDDSGYR;AVMNSFR;DIGIDTGDPSSVQNR;DIISPLILLDAER;DVIHEHVVAHWNDAETEQIIGR;EWNATFVVIDEAHDLSVHGVDYPK;GANVAIILGSR;GLVLTPNR;LSTHDITVEQFNR;NITNDNTIPLDAPR;NNLSDLVPLHNLLMK;RPMPHQILVANYTTPR;SDRDVALLR;SFIAQLDR;SKLEELGAVITDVTCGVQAK;YALPLYPIAESTDESWSYEHLDK;YAWIAK;YQSHAYTIADWGSDAKPNVFVYR | 0.2786 | 64.8941 | 22 | DEAD-like helicase |
| 3H-139 | AHIAPALLNHIESNLLK;ATLDEVSNDPDDIDVER;FDAKILSLTR;FVFSARPTVDSSLASGGVR;HHISPEMYVTMVK;ILSLTR;LAGYLKQSFK;QTTVDKWIDSR;VAAYEIAR | 0.3791 | 32.5105 | 21 | putative S1/P1 nuclease |
| 3H-37 | AGLLELVIK;EFFTLNEDNFTELIK;IDALDDFEVWR;IGYTCNLNDR;ILEYSNAPNAIAK;LHSQVTNLHPLSK;LSELNVASAYDFRPVFVVPTK;LSELNVASAYDFRPVFVVPTKK;QLEAVLHTK;QTLEEQLPR;REFFTLNEDNFTELIK;YWLVNELFPSLK | 0.4306 | 42.9454 | 20 | bro4 |
| 3H-71 | ANNSFKK;KYCADIFDIVQHAASL;LGKPIVVIAPVR;LGKPIVVIAPVRR;LSESVAVIEAKR;NEVAWLLR;NLFTNIR;RLGKPIVVIAPVR;SDSLDACLTFIDIVLDSHR;SRNEVAWLLR;VQFPHVVHNPELNVIYWR;YCADIFDIVQHAASL | 0.4952 | 42.9286 | 20 | sulfhydry1 oxidase Erv1 like protein |
| 3H-77 | MAFVHLFHIDGILECAER;QWATDELK;VAALKQWATDELK;VHGWWWR;VQKVHGWWWR | 0.4940 | 17.4060 | 19 | NA |
| 3H-62 | ATLEQVFVR;AYVDLK;AYVDLKDFVVTHENLFAR;DFVVTHENLFAR;INTGSITDSVQCFNK;LDNNFVDGLK;LFVPGIDLPQPYIGIDTTK;LITDPYQLLQISER;RLFVPGIDLPQPYIGIDTTK;TQTPVNILADIVSLK;YTTSTVPNETDRR | 0.3870 | 41.2875 | 18 | myristylated membrane protein-like protein |
| 3H-58 | AVSPLTNKPIDVHGR;DCNLSPPPTK;HIVVSNGFQPVR;KLIGTSPIR;LIGTSPIR;NDQNVPTYYTSSRPFADR;SIRPEQAAYR;SQLINIANNMNIAELR;TAANADTRPDLCATFSR;VAQNTTQSQLLNIVK;VLLSPIPDGATPMSR;YMINPNFVPTTTK | 0.2447 | 41.9125 | 17 | cofactor of the virion S/T kinase encoded by thioredoxin |
| 3H-64 | AVIGSSGSVNNTVNTVHSR;ESGNNTNISPLNR;GGGNSSHIENVIMIPHK;LPDVGGVNDVISSK;NIILSGEHDR;QIVDEDDGR;SDVIAAKPNYK;SQWADDYSGR;SVTSDVAALAHSETSR;SVTSDVAALAHSETSRSHK;VMLVEYYALLK | 0.3115 | 39.6132 | 17 | NA |
| 3H-93 | DEAYYSATMR;DNFPAAIVSNR;FKDDYGQYEMFR;HAPSSVIADFENLPIAIQANNHCINER;SINGLLLPGGK;SLEEDYK;SLEEDYKTAVK;SYVVASYVK;YFYDYFVQK;YRDNFPAAIVSNR | 0.3269 | 37.2342 | 17 | gamma_glutamyl hydrolase-like protein |
| 3H-152 | ASYMNLLIVEQDR;ERNHIALFMVR;LIGMDGVQLETIR;LVYNASLLLR;NHIALFMVR;QLSAHWR;SEELDILK;SLVIGAEQLER;SQTFYNTQR;STDTNSATFYPK;TSPTLILHVWR;VLPLTAFGK;VLTDSVDTAR;YGEWLK;YITELLR | 0.1090 | 49.76259 | 16 | NA |
| 3H-82 | GGHGSVGEVHTSAVVR;GVHCDNVFDTPK;LGYMAAGYESTYESVVR;RTVSAVGSNEELANTDDSSR;RVCAAFQIKPR;TLIIQSYSHAIVGDAFIQDR;VCAAFQIKPR;YDGEALTR | 0.3180 | 30.3054 | 15 | NA |
| 3H-116 | EHYTLLNTPSATK;ETFYQVFSQVTSVDHR;IAFVGKPGTGK;IQEDLFK;LWEFADDFPNK;LWEFADDFPNKETFYQVFSQVTSVDHR;MIDDSLIIPQFHHNLISPR;TCLDGIFLFGTGNHDVR;VTTPVVGSSPSESASSPIR | 0.3174 | 35.8682 | 14 | ATPase involved in DNA metabolism |
| 3H-150 | LAVEYNGR;LELDCYNEDLR;LQVIFDDNDEK;LQVIFDDNDEKR;NIDLIDVPYTIPIDEIPNFIDK;QHYEYVPR;SADDALLDPTTSPSPVFAR;VRPDWLINPNTK | 0.3948 | 31.3468 | 14 | NA |
| 3H-43 | DFNVASYESDPLLK;DQSLELLSHTHR;FGLASVNDENSYK;FVEFGTLVRPAGWGSLSELGK;KDFNVASYESDPLLK;LSLGYQTVVQFYR;LSYDSVVDWER;LTIDER;VCHLNAVLDPAFTIDTGNVR;YNSSMDEYRDQSLELLSHTHR | 0.5150 | 35.7045 | 14 | NA |
| 3H-69 | AAILLGASLVLNK;DYDTGKDESHYMLYTK;LVYKDYDTGK | 0.4231 | 10.8406 | 14 | NA |
| 3H-15 | DGTVVISEDDKHNEPYGIGK;FFNFKEDFTR;IIPNNESADSR;ILLENK;KTIEALISLPR;LKDPPHTDALK;SNVHIKPVNPPSSK;TIEALISLPR;VGYHTSDLER;VIQTPDPQPVVEGK;YYVDDLVK | 0.3872 | 36.6273 | 13 | NA |
| 3H-182 | APQQIATVK;CDDGELTLHR;DVEFVAR;EGTMTVANHMGSATR;HTGGLIITAPLVHPAVGTTR;RHTGGLIITAPLVHPAVGTTR;SITDMIAAGAPISK;SITLQNTVTK;SLPDPLDIYVR;TQHHNEEVETVDQSILAHVR | 0.2962 | 34.5550 | 13 | NA |
| 3H-109 | ALIVKPMNQFR;ILSLDAVSVPIDQK;LTFFKK;NSWGEHWGDGGYFK;QAVSSSGCGGGSTYR;SISDDLIR;SQLHFETHESK;VALIMSIDAVIR;YLDSLIPTPR | 0.2457 | 33.6784 | 12 | cathepsin B |
| 3H-63 | EYVFGVR;FLILSASGPLK;GSVSDLEFK;GTCAMTLLQQLK;HYTGIIPETMNVAK;LWNASVDSFCYR;NVGLFIPK;SGDEIDGYIIMKPK;VNAEDDCGVEAK;YLWTFAPLLEQR | 0.3688 | 32.4459 | 12 | NA |
| 3H-4 | DFNEFIVWVPTK;LNNLIR;LSHDYAYSLR;NQPNNYVMVR;NSDFAWK;NVTFYVTIK;SSTVVSTVSSATWVK;VYTNVSDIPSAEK | 0.3475 | 29.1559 | 11 | NA |
| 3H-168 | HDDELHFAWQYVDYR;LGMAVTHFER;LNSEISSLYDEVAR;NHIQYQIDSISK;RHDDELHFAWQYVDYR;YQNTIAQLLR | 0.3626 | 23.2068 | 10 | NA |
| 3H-28 | IFMLVLLER;LDEHVPPVAAFIR;LTEWFPNNR;NITLSPQYCSEYLTLK;TAYDQADVIAYIDGDKEK;YNGLMHFISK | 0.2119 | 25.7274 | 10 | NA |
| 3H-46 | HSDDIDTTIDALVK;ISKQLTQLR;KLDQEVLIQEMR;QLTQLR;YMEVSSNDTLLYDNVR | 0.1984 | 17.6919 | 10 | NA |
| 3H-88 | ASFDTVPWVDGR;FNDILK;FVSNTQQLVENSLIYGGLR;ITPVTFMTTVER;NCHTNAASALEAR;NIIPTPLLHHFER;QISHVVYADWLTER;SEMSMWLNGR | 0.3808 | 30.0732 | 10 | putative S1/P1 nuclease |
| 3H-104 | ADGFHTWFFDSVLSNVISSTTMR;DIMLGIFDR;ESYDALSVPSHWR;FVSVVGVYALIVHSDDATR;LWGHLMSMLK;YMWNDEDSEVQILQIQR | 0.5833 | 24.6860 | 9 | bro12 |
| 3H-26 | ANSIIDNIER;QIVDPNATMK;SGATYAINNSYTIDALR;TSEIVDVVDATR;TSEIVDVVDATRK | 0.3731 | 16.8050 | 9 | NA |
| 3H-3 | DYFGWDKLEAAYNAK;IIQIAPVDLIK;KDYFGWDK;LEAAYNAK;TLLKPTVHLNR;YANVNGFEGEFNSQYSVQSYNK;YGTIER | 0.3235 | 25.8497 | 9 | NA |
| 3H-34 | ECDWLLR;NNSEVLLHLYK;QLIPMSLYGHR;RPPGTLLWNAYVR;VFFEGCSFTDYVK;YDEESYCPIETDLAHK | 0.2351 | 23.6446 | 9 | NA |
| 3H-79 | FPFWFER;NEPVSYLAVGNAQFIDR;RNEPVSYLAVGNAQFIDR;SPNCALVFTR;TNPELNDIER;VPVSNTVEFVIVILK | 0.4800 | 22.6032 | 9 | NA |
| 3H-157 | DLPDTVPVSLDVR;GINDLSDAELAYK;NPPLELWSR;NSTMNLPR;TLLLHYR;VLIDTEQK;VLIDTEQKR | 0.3410 | 24.7760 | 8 | tyrosyl-DNA phosphodiesterase |
| 3H-21 | EVEDLVLR;FKEVEDLVLR;FLSDHPHMTK;IASLLVHDIHSSLDDAVK;IGSIAPIELNPPPR | 0.2281 | 17.3096 | 7 | NA |
| 3H-76 | DLEGIVMR;FNEYDATLR;LVLGSQSPYNAYVLNYGSAENQLR;RFNEYDATLR;VYLMSVEEAK;YPFTNGYGAYVVEK | 0.1634 | 19.2213 | 7 | NA |
| 3H-118 | LNGQLHTLVIPK;RYAYTDSDGVYVK;SIVAAFATADLPTALYHSETATYR;YAYTDSDGVYVK | 0.2917 | 13.5342 | 6 | NA |
| 3H-128 | DVISDQPR;ELAIGIDER;QTSSSQQRPNDLLYPLSYGAVDETVVLK;YADIPVWEQGVEDEPTSETNDTLNR | 0.2612 | 13.9047 | 6 | NA |
| 3H-134 | DSILQPDTLAK;IITHEEYVNLEADFESYKK;ISNILSPK;SVTSFAAFTDNDHEPPPK;VATYPPPTSNDESASHER | 0.3379 | 18.7166 | 6 | NA |
| 3H-44 | DNSEASSQNTTVWDR;DNSEASSQNTTVWDRR | 0.1584 | 8.5758 | 6 | NA |
| 3H-124 | DLEANASLLR;INDPVLVIDSAR;TTGYQQLYAELK;VGENSAWHNFETR;VVDILR | 0.0808 | 13.3245 | 5 | serine/threonine protein kinase |
| 3H-115 | HNNFTQDHEVTLGAPTASR;LITATEEELK;TATATAGLFGATPLIGTIAGQVK;VAGIENLVLQTK | 0.1067 | 16.1102 | 4 | NA |
| 3H-126 | FDFDIK;FVNDFFTSLFPDPEVK;VFWALK;WTLEVINWR | 0.0352 | 11.1536 | 4 | ATPase involved in DNA replication |
| 3H-131 | AKDVAVALEFVNTEQAIR;SKLPAAEEFQR;WLFEEVLPTGK | 0.2051 | 10.7809 | 4 | bro16 |
| 3H-138 | SNVSLPR;STLGVNFIR;TLIELEK;YDGNTLTNSTILGALMGEFR | 0.2792 | 14.0274 | 4 | NA |
| 3H-149 | TIHWVDYTCPGDDTHR;TTIEDVQK;WLDVQLK | 0.2246 | 9.7752 | 4 | NA |
| 3H-16 | LGNVLFWNDLLK;LVAGGLIVIR;QLADGLGFILSK;YVDLLDPFGNSIPVTGR | 0.0779 | 15.0688 | 4 | lipase like protein |
| 3H-181 | HIPSADDPGADLFTNWFSINAHDR;WYHLDCFLSK | 0.3063 | 6.98151 | 4 | NA |
| 3H-22 | DSHEIYINAWK;HASNDALNAIVYR;NHYDDTLYDTSVK | 0.1888 | 11.8223 | 4 | NA |
| 3H-52 | DHSSVYTIDGDR;EHVAGELTAFLSSLR;IVVMER;SPLSAYK | 0.1843 | 13.0217 | 4 | thymidine kinase |
| 3H-72 | EFEYFK;ELQPPYSNYFEESLKPEAFVSNIHYK;QEYEDDDYNFK | 0.2774 | 10.6196 | 4 | phenylalanyl-Trna synthetase α subunit |
| 3H-83 | ESFGTYMGQR;KPAQELIHEYR;MSPELQEAWR | 0.2039 | 11.8223 | 4 | glycosyltransferase |
| 3H-95 | CLYTTVAFR;TKYTYAIANPEHER;YTYAIANPEHER | 0.1811 | 12.8637 | 4 | NA |

Table S7 Identified HvAV-3h coded proteins from *Mythimna separata* produced virions (Ms-3)

| Protein ID | Identified peptide sequence | Sequence coverage | Protein Qscore | Unique spectra | Function annotation |
| --- | --- | --- | --- | --- | --- |
| 3H-56 | AHMFKPKQTLAR;DIHKFNGSK;DMNGIAVSVDAK;EEDGWDVATTEK;EEDGWDVATTEKVYR;IISETVK;IISETVKIMNGAGAAGGAVVPK;IMNGAGAAGGAVVPK;IVKAHMFKPK;LKEEDGWDVATTEK;LQPAPLDIK;LQPAPLDIKGGRPMVR;LTNDEMNR;LYPALMSPNTITNVIPDAR;LYPALMSPNTITNVIPDARNGVHSTDR;MELHFEFM;MTSVVLNFSSPLTGEYVLQMLNALNPTK;NGVHSTDR;SSVMVIGLSEVFMQQTSVTFR;TIIGDLGSLQGPEGGEWLAFFYPAHTVFEK;TIIGDLGSLQGPEGGEWLAFFYPAHTVFEKMR;VGETPDLSSFSQEEISK;VGETPDLSSFSQEEISKLK | 0.9587 | 76.7617 | 123 | NA |
| 3H-55 | AYELANER;AYELANERGPFSVEVYLNPGTSNTYQYVATTR;DSYRLPQSFTR;FNNTDYDDLPWNYTSGK;FNNTDYDDLPWNYTSGKK;GIASLWGYLK;GPFSVEVYLNPGTSNTYQYVATTR;IGQTPKAYELANER;KVVTATGVVSGGER;LPQSFTR;LPQSFTRDPLFR;NKFNNTDYDDLPWNYTSGK;SEIAQDIQVTMYNTNGGSNPLNNSNVTR;SNVDSSSYYQQPPQVVYNNGDLYGSR;TGYSGAELGASIDK;TGYSGAELGASIDKGIASLWGYLK;VVTATGVVSGGER;VVTATGVVSGGERYVFLLR;YVFLLR;YYYMSRPIGFGSSGAYDVPLLDTPLLR | 0.8800 | 70.5022 | 119 | NA |
| 3H-50 | ACSAPSWTGIPSVNYNNQLMGASMFPNQPLA;ASCGTIPDAR;ATHYDPIR;ATHYDPIRDGYYMYALPNGR;DGYYMYALPNGR;DGYYMYALPNGRVDR;DYFLSSKYK;FLKPTFWFSR;LPSDSGVAVTTSPTVVDDLDLR;LPSDSGVAVTTSPTVVDDLDLRIR;NALVALSNSSMNNEQNYTNR;NVYKDPAR;YDNFQCNK;YNNLSWLLSK;YNNLSWLLSKNVYK | 0.8095 | 49.0855 | 85 | NA |
| 3H-57 | AKLDLVTINSVANK;ALLEVTDNVVTDR;ALLEVTDNVVTDRVPVR;AQLDVADLER;AQLQSDLDVLR;DEAIWAIK;EHDVLER;EKAQLQSDLDVLR;EKQHAEFLR;EMYAVLEDELK;GELNAMTR;GGMGVLDDIYNR;HVGNAQSDGMYTENVALR;IESEAALVESLQR;IQAFVNTEQNILAAGTR;ISELLTQLQESNNQR;ITNEFFDHATLPLLR;KQMGNVVQEIAQR;KTNDNLEIK;LDGSDVTLVVDDENLK;LDLVTINSVANK;LLKEHDVLER;LLSLDGVR;LLSLDGVRR;LNDIAELQSQLTK;LNNELETSITEER;LQLDYMTLQQR;MEFEAVNDALSK;MLELQNLMK;NAEITNLQDR;NELAATVNSLQMQATK;NIDGAEGFTR;NISAIRPR;NNVQLQTALQR;NSGVGNAINDR;NVNDTTLAELAQSR;QHAEFLR;QHPSFANKPNNTDVQQSDLTIAEDADTGTSVTK;QLNALNLR;QQLEWILVNSNPAIALQDASVR;RLNNELETSITEER;RNNVQLQTALQR;RPIRPMADNFAEGYVYERPMGGGAPR;RPSVTDDEDDRIVGTR;SETNALVVEVADLK;SEYELSASR;SLSYSLTDDEDDAAQIR;SVTGPIR;TQLLTGLR;TYQQEMTQLK;VAYANLEGDMQK;VITGDETVMTYNR;VSTDDLTSYSR;YEALMSEQSSK;YESLMTK | 0.4241 | 168.78250 | 84 | chromosome segregation SMC like protein |
| 3H-140 | ALSHPDWLTIGSFIK;DVDGVASNGGGSVGTGLGGPLGGTSGVNTSNLR;ELFYHSEAGR;ENFVNLVELWCK;ETNYPIPYFQYNLVLSQR;EVAKELFYHSEAGR;GSVSKSGDEMTLSR;MKTLDRPTDNAIMQR;NSDKDVTIPK;SGDEMTLSR;STGTTVGYTGTTSNAAMK;TATHLLGYK;TATHLLGYKNK;TDVNATNKSTGTTVGYTGTTSNAAMK;TLDRPTDNAIMQR;YTYMYVNK;YTYMYVNKR | 0.8349 | 56.5217 | 63 | NA |
| 3H-53 | DSGVALPIGMMVNNR;EIRPCAAFSK;ENVPDTGAGTGFMNMLTDGDFATPPEITK;GQYMYSFALDLK;GSINPSNFNSSISVVIEPSEALR;GTIDSYIYGPDEQVTTYFVR;KLSDLLIK;MPVLLSTGNGSNK;NITHSEYLDYYEVGLPK;RGTIDSYIYGPDEQVTTYFVR;SIGVSALSKIGIK;SYAAGTESSYFPK;TLVLPIPLYFSR;TSCSPHYILMEKPTDIAATEILKPTEAGSTLSYDIEHSSGILK;VPMLPAEHFVFTEPYR;VTVEFVFR;VTVEFVFRK;YRVPMLPAEHFVFTEPYR | 0.5604 | 60.5071 | 61 | major capsid protein |
| 3H-27 | ADNVWVYTEDLQAIK;ALINEDGHSVVTQR;AMIESILK;CLLSITR;DDNSPSNDSVAMK;FNIDNVNYASVAHAIALISVVYHMK;FTDEIIK;FTDEIIKK;GVVWSEQSQQLVAAAATTK;HIHSVGDPSYNDEVTR;HSNVYSDALESFR;IVTSQLLYK;KADNVWVYTEDLQAIK;KVDEFISFGR;RPDAEECFK;SLVLHFGANPAYTIMNTFASPAESPR;SLVVLTLQHELR;SLVVLTLQHELRK;SPPTLTIDDDSILYPLQYIPR;STEDDRPIQMQR;TDYELR;TLFGDVVQK;VLTAALMHTDADTVNPTTPSVGYESDAVPR;YAIDVNILPAISDTIQK;YTDFDQLWSK;YVPINR | 0.4177 | 82.3497 | 37 | NA |
| 3H-48 | ALLLSQLVSPPLIAK;AMHLLYIEGK;ATQNTVQTLR;CLGQVVTNVR;GKLYGYMANK;GSSISTAVSK;LKNWSPK;LQSWVTDHEAVQYGTKPTVPYVIPDNLLSQKPTDNTISR;NDITAKNITSK;NEIIAKNDITAK;SVASVSSPGNIASSSVGNK;SVASVSSPGNIASSSVGNKR;TAQSIMQNLMTQIK;THIFNYLDSK;TVNVNQDSQTIK;TVNVNQDSQTIKTHIFNYLDSK;VDQEVYMK | 0.3951 | 51.8687 | 35 | NA |
| 3H-107 | ASLKTNTPLEGVPEFVLFHYGNAVK;FFPAETHVEVK;GILSIRR;IDHVPSAVVK;LQLSDWNDNVTTIK;LQLSDWNDNVTTIKK;MMDHVFTLTPSDVTVR;NYVLNEPPAR;RIDHVPSAVVK;SYSAYR;TNTPLEGVPEFVLFHYGNAVK;TNTPLEGVPEFVLFHYGNAVKTLDR;YASDSLMYFECDLSIR | 0.7362 | 38.0394 | 32 | thioredoxin-like protein |
| 3H-66 | DIIMGHISPNR;IFLATNTNSHVR;ISTIPEFHTKPK;KVQVDYSPNYDLTVVTVNDHVSTTK;QAIVQNGR;RSLAHEHEEEEVFNDNEK;SIFSSPIPNLGFVAAENGHVR;SLAHEHEEEEVFNDNEK;SLAHEHEEEEVFNDNEKYDDER;SPASDQSYYGESNDGSYK;TVISGDTYIK;VQVDYSPNYDLTVVTVNDHVSTTK;VTVHEVNADLYLCKPR | 0.4216 | 41.9147 | 30 | NA |
| 3H-110 | ADGTAIRTYK;ATDINPYETK;FLTNEIK;FLTNEIKADGTAIR;HLNPQWATDAPILK;IGFISQILPR;KATDINPYETK;LGNVSGIITK;LTFNGTAK;MAGLPLHAK;NVDDDTIELR;TSNKPLIFECILDEPEK;TYVTLNR;VVLAPEK;VYEFISR | 0.5962 | 43.7885 | 29 | NA |
| 3H-117 | CIPVVAYHAQDADAPTDTGLK;DDAIIIDDLPDVYITQPHR;DYAAVITAELILR;LDSAESEMAR;LEMFLSSDETR;MFAKPEPAIR;NLNALWDLWGADVIIER;NVNGFVPAVIFSDYEVYK;RLEMFLSSDETR;RPHLDEFLSYLFENFR;SRPTVFLDLDNTLICSLR;TAGEEVGGVK;VGVWTAASK | 0.9153 | 42.1832 | 28 | CDT phosphatase transcription factor |
| 3H-137 | ACMTSSVK;AMSNGLGDVILSAK;DTMAMIFK;KIFDAFK;NALLTLK;NNCVNVCTTIK;RTGVDLSVNVGQTYK;SEDEEVSAEIVTAK;TGVDLSVNVGQTYK;VSGDVIDK;VSTTVDPMMPAQVK;YSVAVAK | 0.7063 | 37.3446 | 26 | hemolysin-like protein |
| 3H-151 | ALQYQTQLEGLLSR;CKPGEVMDDK;CKPGEVMDDKSSTTEER;ETCALTTVTASLATISESLGK;ETCALTTVTASLATISESLGKDEALLK;ITEVGGGVAAIK;LDAIKSDISGK;LVQIDNTVK;RVVVDPLDTK;SSLTSVVFTPLSEK;SSLTSVVFTPLSEKLDAIK;VAFGLTR;VVVDPLDTKVEK;VVVVPVQDK;VVVVPVQDKITEVGGGVAAIK | 0.6552 | 48.1619 | 24 | Uyr/REP helicase |
| 3H-156 | DCYVDHSAGVPTYFVVER;DTGHLVAFINGGTR;FYDSSTSTCPINYR;IVQFPPGSPYTFVATR;QTVLAQSNSGAYPVER;RPAALPPR;RQTVLAQSNSGAYPVER;TSDDVPYEATVNTANMYDGDASGR;VDVQSCMNIFTTTGPSR | 0.3333 | 31.2629 | 23 | NA |
| 3H-84 | ASIQSGYNK;ELMQYIR;LLSPPVDPHVFIDTTAASIDK;NMVINEPQYQQFLR;NSEEDSSPAQADQSESVGSSPPTK;SIDTNEGNDDDSTAIALR;SPHLDMAFR;TGTEPKPEHSDDGKNSEEDSSPAQADQSESVGSSPPTK;TMEFVR;VVAKPQYDDRELMQYIR | 0.5714 | 33.5242 | 23 | ABC-type transport system permease |
| 3H-135 | ALNMILK;ALNMILKR;DQVPVLTK;EFPNISCR;KRPIQPTAYNLFYK;QYNPKYLLDNYNYR;RPIQPTAYNLFYK;RPIQPTAYNLFYKDQVPVLTK;WNYHKQYNPK;YLLDNYNYR | 0.4560 | 28.2440 | 22 | yabby-like transcription factor |
| 3H-37 | AGLLELVIK;DDNDQLWMLANPFAR;EFFTLNEDNFTELIK;FINKAGLLELVIK;FQFNSQELEVIGVK;IDALDDFEVWR;IGYTCNLNDR;ILEYSNAPNAIAK;LHSQVTNLHPLSK;LSELNVASAYDFRPVFVVPTK;LSELNVASAYDFRPVFVVPTKK;MCSDFLISNK;QLEAVLHTK;QTLEEQLPR;REFFTLNEDNFTELIK;YWLVNELFPSLK | 0.5799 | 45.4600 | 22 | bro4 |
| 3H-139 | AHIAPALLNHIESNLLK;ATLDEVSNDPDDIDVER;FDAKILSLTR;FVFSARPTVDSSLASGGVR;HHISPEMYVTMVK;ILSLTR;QTTVDKWIDSR;VAAYEIAR;VCAAVAR | 0.3682 | 29.8717 | 21 | putative S1/P1 nuclease |
| 3H-13 | DIISPLILLDAER;DVIHEHVVAHWNDAETEQIIGR;EWNATFVVIDEAHDLSVHGVDYPK;GANVAIILGSR;LSTHDITVEQFNR;LTTYAEFNETQLLYSDDDDRDR;NIDGALR;NITNDNTIPLDAPR;QRPTFVYPFVLDNVSLYGLDAYR;SPIDGIIVIHGVGTGK;SSIEAQLNNDMLAK;TVDILMYATSEAK;YAWIAK;YQSHAYTIADWGSDAKPNVFVYR | 0.2341 | 43.1896 | 18 | DEAD-like helicase |
| 3H-15 | DGTVVISEDDKHNEPYGIGK;FFNFKEDFTR;IIPNNESADSR;KTIEALISLPR;LKDPPHTDALK;SKYPALVKPYEEYQR;SNVHIKPVNPPSSK;TIEALISLPR;TLIPLQKPIAPVQISEKPVVVK;VIQTPDPQPVVEGK;YYVDDLVK | 0.4579 | 31.2388 | 16 | NA |
| 3H-182 | APQQIATVK;CDDGELTLHR;HTGGLIITAPLVHPAVGTTR;RHTGGLIITAPLVHPAVGTTR;SITDMIAAGAPISK;SITLQNTVTK;SLPDPLDIYVR;SSIIHNIQK;TQHHNEEVETVDQSILAHVR;VFECPNTEEVPNFR | 0.2987 | 32.4249 | 16 | NA |
| 3H-43 | DFNVASYESDPLLK;DLYNLQILR;DQSLELLSHTHR;FGLASVNDENSYK;FVEFGTLVRPAGWGSLSELGK;KDFNVASYESDPLLK;LSLGYQTVVQFYR;LSYDSVVDWER;MFNVDQAIEFFENR;RFGLASVNDENSYK;SSPQTVGLEWWK;TLSSMIR;VCHLNAVLDPAFTIDTGNVR;YNSSMDEYRDQSLELLSHTHR | 0.6738 | 44.7754 | 16 | NA |
| 3H-62 | ATLEQVFVR;AYVDLKDFVVTHENLFAR;DFVVTHENLFAR;KTQTPVNILADIVSLK;LDNNFVDGLK;LFVPGIDLPQPYIGIDTTK;LITDPYQLLQISER;RLFVPGIDLPQPYIGIDTTK;TQTPVNILADIVSLK;YTTSTVPNETDRR | 0.3390 | 32.3927 | 16 | myristylated membrane protein-like protein |
| 3H-64 | AVIGSSGSVNNTVNTVHSR;ESGNNTNISPLNR;FLGLSGIFSMLADIGNTNK;GGGNSSHIENVIMIPHK;LPDVGGVNDVISSK;NIILSGEHDR;SDVIAAKPNYK;SKESGNNTNISPLNR;SVTSDVAALAHSETSR;VMLVEYYALLK | 0.3091 | 32.2875 | 15 | NA |
| 3H-116 | EHYTLLNTPSATK;ETFYQVFSQVTSVDHR;IAFVGKPGTGK;IQEDLFK;LWEFADDFPNK;LWEFADDFPNKETFYQVFSQVTSVDHR;MIDDSLIIPQFHHNLISPR;VTTPVVGSSPSESASSPIR;YIMYTK | 0.2865 | 29.2506 | 14 | ATPase involved in DNA metabolism |
| 3H-19 | ATVGALAGDVVK;ESTTNIAEDIEREPAYLK;GDDVRPELSTFVLSYTR;IFSMLDNEIHLTIDSSDHQHVLQIYNR;ILITTYDYSGVQQR;IMDVIVSDLGTFNGPIPNDADHYMFK;QTLGLVIK;SETTDASNVSPR;SHVDDNEDLAPSK;SHVDDNEDLAPSKR;TPSPTAVEVNTLAR;TVLDAAR | 0.2616 | 39.5451 | 14 | NA |
| 3H-77 | CVENDKPATK;MAFVHLFHIDGILECAER;QWATDELK;VAALKQWATDELK;VHGWWWR;VQKVHGWWWR | 0.6145 | 18.8157 | 14 | NA |
| 3H-88 | ASFDTVPWVDGR;FNDILK;FVSNTQQLVENSLIYGGLR;ITPVTFMTTVER;NCHTNAASALEAR;NIIPTPLLHHFER;QISHVVYADWLTER | 0.3423 | 21.5719 | 14 | putative S1/P1 nuclease |
| 3H-93 | DEAYYSATMR;DNFPAAIVSNR;FKDDYGQYEMFR;HAPSSVIADFENLPIAIQANNHCINER;SAYEWYESR;SINGLLLPGGK;SLEEDYKTAVK;SYVVASYVK;YRDNFPAAIVSNR | 0.3269 | 27.6587 | 14 | gamma_glutamyl hydrolase-like protein |
| 3H-150 | FHNCPNDLTTQIAR;LAVEYNGR;LELDCYNEDLR;LQVIFDDNDEKR;MNGYSGTWQPLR;NIDLIDVPYTIPIDEIPNFIDK;QGVNTLAEYGK;QHYEYVPR;SADDALLDPTTSPSPVFAR;VRPDWLINPNTK | 0.5536 | 30.9890 | 13 | NA |
| 3H-46 | HSDDIDTTIDALVK;ISKQLTQLR;KLDQEVLIQEMR;LDQEVLIQEMR;QCLSLIDEYAE;QLTQLR;YMEVSSNDTLLYDNVR | 0.2412 | 23.0202 | 13 | NA |
| 3H-71 | KYCADIFDIVQHAASL;LGKPIVVIAPVR;LGKPIVVIAPVRR;LSESVAVIEAK;LSESVAVIEAKR;NEVAWLLR;NLFTNIR;VQFPHVVHNPELNVIYWR;YCADIFDIVQHAASL | 0.3558 | 29.7792 | 13 | sulfhydry1 oxidase Erv1 like protein |
| 3H-82 | ATITEVGNR;GGHGSVGEVHTSAVVR;GVHCDNVFDTPK;LGYMAAGYESTYESVVR;RTVSAVGSNEELANTDDSSR;TLIIQSYSHAIVGDAFIQDR;TVSAVGSNEELANTDDSSR;YDGEALTR | 0.3119 | 27.2775 | 13 | NA |
| 3H-109 | ALIVKPMNQFR;ILSLDAVSVPIDQK;KSISDDLIR;QAVSSSGCGGGSTYR;SQLHFETHESK;SVIPTNATTNAYNAVK;THILNR;VALIMSIDAVIR;YLDSLIPTPR | 0.2530 | 27.6391 | 12 | cathepsin B |
| 3H-63 | EYVFGVR;FLFLDEANKPLHEIAVQFTCK;FLILSASGPLK;GSVSDLEFK;GTCAMTLLQQLK;HYTGIIPETMNVAK;KNVGLFIPK;LWNASVDSFCYR;NVGLFIPK;TAFSIVNK;YLWTFAPLLEQR | 0.3821 | 29.7240 | 12 | NA |
| 3H-76 | DLEGIVMR;EFPLVNPAR;ELYPEHVK;FNEYDATLR;IAMHGDVSR;LVLGSQSPYNAYVLNYGSAENQLR;MQIHFVNSEYSALSK;RFNEYDATLR;SDLNALLR;SLWSTDETLPR;VYLMSVEEAK;YPFTNGYGAYVVEK | 0.3119 | 34.6977 | 12 | NA |
| 3H-69 | AAILLGASLVLNK;DYDTGKDESHYMLYTK | 0.3718 | 7.1280 | 11 | NA |
| 3H-28 | GIDDGDAIYTITK;IFMLVLLER;LDEHVPPVAAFIR;LFFDLR;LTEWFPNNR;NITLSPQYCSEYLTLK;NNLNYDQLYTALR;TAYDQADVIAYIDGDKEK;YNGLMHFISK | 0.3023 | 27.3044 | 10 | NA |
| 3H-104 | ADGFHTWFFDSVLSNVISSTTMR;DIMLGIFDR;ESYDALSVPSHWR;FVSVVGVYALIVHSDDATR;LWGHLMSMLK;YMWNDEDSEVQILQIQR | 0.5833 | 19.8571 | 9 | bro12 |
| 3H-21 | EVEDLVLR;FKEVEDLVLR;FLSDHPHMTK;IASLLVHDIHSSLDDAVK;IGSIAPIELNPPPR;NVETFVR;YNNDSIR | 0.2895 | 22.1719 | 9 | NA |
| 3H-58 | AVSPLTNKPIDVHGR;HIVVSNGFQPVR;LIGTSPIR;LYNDLTK;NDQNVPTYYTSSRPFADR;SQLINIANNMNIAELR;TAANADTRPDLCATFSR;VAQNTTQSQLLNIVK | 0.1762 | 26.0975 | 9 | cofactor of the virion S/T kinase encoded by thioredoxin |
| 3H-152 | LVYNASLLLR;RVDDAELER;STDTNSATFYPK;TSPTLILHVWR;TVSLFRPPLSLK;VLPLTAFGK;YGEWLK;YITELLR | 0.0605 | 21.6505 | 8 | NA |
| 3H-34 | ECDWLLR;NNSEVLLHLYK;QLIPMSLYGHR;RPPGTLLWNAYVR;YDEESYCPIETDLAHK | 0.1921 | 14.7310 | 8 | NA |
| 3H-79 | FPFWFER;RNEPVSYLAVGNAQFIDR;SPNCALVFTR;TNPELNDIER;VPVSNTVEFVIVILK | 0.4800 | 16.6207 | 8 | NA |
| 3H-168 | LGMAVTHFER;NHIQYQIDSISK;RHDDELHFAWQYVDYR;YQNTIAQLLR | 0.2807 | 11.8808 | 7 | NA |
| 3H-26 | ANSIIDNIER;QIVDPNATMK;SGATYAINNSYTIDALR;TSEIVDVVDATR;TSEIVDVVDATRK | 0.3731 | 17.1062 | 7 | NA |
| 3H-29 | ISPIFDGSVAILHDLSSGLVAAAISK;NHGTSVSIVGDDHK;NSSVVMVK;VFNDINVVK | 0.5816 | 12.4420 | 7 | NA |
| 3H-3 | DYFGWDKLEAAYNAK;IIQIAPVDLIK;KDYFGWDK;TLLKPTVHLNR;YANVNGFEGEFNSQYSVQSYNK | 0.2941 | 14.8585 | 7 | NA |
| 3H-42 | DLGSNNFWDK;EITLYEHAK;ELYDGFTSDVR;SVGECIGGDPVANFK;VTSVEIK;YGTLSGIPALFR | 0.2735 | 16.5604 | 7 | NA |
| 3H-113 | AAGFIVIEQDINTDNVTLVHEALR;AFMELIR;ALVVDPPQSVVR;DSSTTDEALK;EWFVQK;RLDIAFSGTDK | 0.2029 | 17.8288 | 6 | lysophospholipid acetyltransferase |
| 3H-118 | LNGQLHTLVIPK;RYAYTDSDGVYVK;SIVAAFATADLPTALYHSETATYR;YAYTDSDGVYVK | 0.2917 | 11.8808 | 6 | NA |
| 3H-171 | ILIVINQR;IYSVIGWLCK;LGFQEFVYR;LKDFLTLFDVDELR;MFVVQTNR | 0.1561 | 14.9480 | 6 | caspase-like protein |
| 3H-4 | DFNEFIVWVPTK;LNNLIR;LSHDYAYSLR;NQPNNYVMVR;SSTVVSTVSSATWVK;VYTNVSDIPSAEK | 0.2797 | 16.4036 | 6 | NA |
| 3H-72 | EFEYFK;ELQPPYSNYFEESLKPEAFVSNIHYK;QEYEDDDYNFK;VTTSSILR | 0.3290 | 11.8809 | 6 | phenylalanyl-Trna synthetase α subunit |
| 3H-8 | DGYVQGIAAVYYAPHTSAATDQAFK;LNAQDVAVLGTSNPILR;NMYFSNR;QLQNMYIVVDEFLK;VYIPVPPITR | 0.1678 | 13.7135 | 6 | NA |
| 3H-134 | DSILQPDTLAK;IITHEEYVNLEADFESYKK;ISNILSPK;SVTSFAAFTDNDHEPPPK;VATYPPPTSNDESASHER | 0.3379 | 15.2828 | 5 | NA |
| 3H-149 | GFDNFTNINENFYK;NVSSLDMINITTEEQR;TIHWVDYTCPGDDTHR;TTIEDVQK | 0.3913 | 14.2560 | 5 | NA |
| 3H-157 | DLPDTVPVSLDVR;GINDLSDAELAYK;NPPLELWSR;VLIDTEQKR | 0.2543 | 12.1732 | 5 | tyrosyl-DNA phosphodiesterase |
| 3H-181 | HIPSADDPGADLFTNWFSINAHDR;WYHLDCFLSK | 0.3063 | 6.73220 | 5 | NA |
| 3H-81 | HLAVDGLR;LFSTSTNR;LTMYHTALFR;VTMFAPVYAYR | 0.1516 | 11.1863 | 5 | NA |
| 3H-124 | INDPVLVIDSAR;TTGYQQLYAELK;VGENSAWHNFETR;VVDILR | 0.0655 | 11.5742 | 4 | serine/threonine protein kinase |
| 3H-128 | QTSSSQQRPNDLLYPLSYGAVDETVVLK;VVGVTYK;YADIPVWEQGVEDEPTSETNDTLNR | 0.2239 | 10.8169 | 4 | NA |
| 3H-133 | DTTFSQFYER;FIEEFGDFLEELGHSR;TLVISGGGFK | 0.1169 | 8.9368 | 4 | patatin-like phospholipase |
| 3H-16 | LGNVLFWNDLLK;NQGVNDVLHAIDWTIENAR;QLADGLGFILSK | 0.0656 | 10.1713 | 4 | lipase like protein |
| 3H-170 | ILTTANDVIEHHFENNPLNLTTTPTLSAEDNGK;SLQLELVEVK | 0.4674 | 6.93490 | 4 | NA |
| 3H-22 | DSHEIYINAWK;HASNDALNAIVYR;NHYDDTLYDTSVK | 0.1888 | 9.7755 | 4 | NA |
| 3H-44 | DNSEASSQNTTVWDR;DNSEASSQNTTVWDRR | 0.1584 | 8.1694 | 4 | NA |
| 3H-51 | DKVSTIVSDVK;EMLEDVKDK;QFTGDVFPRPITVDGING | 0.1739 | 8.7341 | 4 | NA |
| 3H-59 | ALDELMDFK;HVSAEIELK;KDNEISQLHEELDYATETINDLNDQLR | 0.2616 | 7.8341 | 4 | putative nucleic |
| 3H-83 | ESFGTYMGQR;KPAQELIHEYR;MSPELQEAWR | 0.2039 | 9.1299 | 4 | glycosyltransferase |

Table S8 Identified HvAV-3h coded proteins from *Spodoptera exigua* produced virions (Se-1)

| Protein ID | Identified peptide sequence | Sequence coverage | Protein Q score | Unique spectra | Function annotation |
| --- | --- | --- | --- | --- | --- |
| 3H-57 | AEMQINTQR;AKLDLVTINSVANK;ALEMKEMWER;ALLEVTDNVVTDR;ALLEVTDNVVTDRVPVR;AQLDVADLER;AQLQSDLDVLR;ATIDALTANLNSSNSK;CQAELQQR;DEAIWAIK;DQLLKLWR;DSSFPIVDADNTEQLVVR;EKAQLQSDLDVLR;EKQHAEFLR;ELACNVIPEK;ELNTVR;EMWER;EMYAVLEDELK;FQMHITNDFLDLVQPEGIATLASYLSVSESQISDR;GELNAMTR;GGMGVLDDIYNR;HANDVR;HENLYLASTTPSR;HIAGVITK;HVGNAQSDGMYTENVALR;IESEAALVESLQR;IQAFVNTEQNILAAGTR;ISELLTQLQESNNQR;ISNSVKNDVESK;ITNEFFDHATLPLLR;IVENMYK;IVENMYKR;KQMGNVVQEIAQR;KTNDNLEIK;LDGSDVTLVVDDENLK;LDLVTINSVANK;LIDSVVK;LIDSVVKITR;LLISSETLGR;LLKEHDVLER;LLSLDGVR;LLSLDGVRR;LLVSEDK;LNDIAELQSQLTK;LNNELETSITEER;LQLDYMTLQQR;LSNASVGVQLDR;LTNECQAVSSR;MEFEAVNDALSK;MLELQNLMK;MTAAHLADDR;MTAAHLADDRLSNASVGVQLDR;NELAATVNSLQMQATK;NIDGAEGFTR;NIDGAEGFTRDEAIWAIK;NISAIRPR;NNVQLQTALQR;NSGVGNAINDR;NSGVGNAINDRLNK;NVNDTTLAELAQSR;QHAEFLR;QHPSFANKPNNTDVQQSDLTIAEDADTGTSVTK;QLNALNLR;QLNALNLRR;QMGNVVQEIAQR;QQLEWILVNSNPAIALQDASVR;QSLLETSAQLNDVR;RLLVSEDK;RLNNELETSITEER;RNNVQLQTALQR;RPIRPMADNFAEGYVYERPMGGGAPR;RPSVTDDEDDRIVGTR;RSLSYSLTDDEDDAAQIR;SDVVVLR;SETNALVVEVADLK;SETNALVVEVADLKSQLNR;SEYELSASR;SEYELSASRR;SLSYSLTDDEDDAAQIR;SNANLSPQSIR;SQALYAIK;SQALYAIKR;SVTGPIR;SYIIELESR;TKEMYAVLEDELK;TNDNLEIK;TQLLTGLR;TYQQEMTQLK;TYQQEMTQLKR;VAYANLEGDMQK;VITGDETVMTYNR;VNRNSGVGNAINDR;VSTDDLTSYSR;YDNYNRNEHELR;YEALMSEQSSK;YESLMTK;YQAAGTPGNGR | 0.6415 | 369.7496 | 199 | chromosome segregation SMC like protein |
| 3H-53 | ALFFGVR;DSGVALPIGMMVNNR;GQYMYSFALDLK;GSINPSNFNSSISVVIEPSEALR;GTIDSYIYGPDEQVTTYFVR;KIDNGGDFN;KLSDLLIK;LSDLLIK;MPVLLSTGNGSNK;NITHSEYLDYYEVGLPK;NITHSEYLDYYEVGLPKSNNGVR;RGTIDSYIYGPDEQVTTYFVR;SDVNKR;SIGVSALSK;SIGVSALSKIGIK;SVDIGAKTLVLPIPLYFSR;SYAAGTESSYFPK;SYAAGTESSYFPKLAHK;TLVLPIPLYFSR;TSCSPHYILMEKPTDIAATEILKPTEAGSTLSYDIEHSSGILK;VPMLPAEHFVFTEPYR;VTVEFVFR;VTVEFVFRK;YRVPMLPAEHFVFTEPYR | 0.5582 | 95.8556 | 126 | major capsid protein |
| 3H-140 | ALSHPDWLTIGSFIK;ALSHPDWLTIGSFIKETNYPIPYFQYNLVLSQR;DVDGVASNGGGSVGTGLGGPLGGTSGVNTSNLR;ELFYHSEAGR;ENFVNLVELWCK;ETNYPIPYFQYNLVLSQR;EVAKELFYHSEAGR;GSVSK;GSVSKSGDEMTLSR;MLYGNSKTDVNATNK;NSDKDVTIPK;SGDEMTLSR;STGTTVGYTGTTSNAAMK;STGTTVGYTGTTSNAAMKDVDGVASNGGGSVGTGLGGPLGGTSGVNTSNLR;TATHLLGYK;TATHLLGYKNK;TDVNATNK;TDVNATNKSTGTTVGYTGTTSNAAMK;TLDRPTDNAIMQR;YFFKENFVNLVELWCK;YTYMYVNK;YTYMYVNKR | 0.8774 | 88.8930 | 109 | NA |
| 3H-55 | AYELANER;AYELANERGPFSVEVYLNPGTSNTYQYVATTR;DPLFRNSV;DSYRLPQSFTR;FNNTDYDDLPWNYTSGK;FNNTDYDDLPWNYTSGKK;GIASLWGYLK;GPFSVEVYLNPGTSNTYQYVATTR;IGQTPKAYELANER;KVVTATGVVSGGER;LPQSFTR;NKFNNTDYDDLPWNYTSGK;SEIAQDIQVTMYNTNGGSNPLNNSNVTR;SNVDSSSYYQQPPQVVYNNGDLYGSR;TGYSGAELGASIDK;TGYSGAELGASIDKGIASLWGYLK;VVTATGVVSGGER;VVTATGVVSGGERYVFLLR;YVFLLR;YYYMSRPIGFGSSGAYDVPLLDTPLLR | 0.8933 | 81.0125 | 92 | NA |
| 3H-56 | DMNGIAVSVDAK;EEDGWDVATTEK;IISETVK;IMNGAGAAGGAVVPK;IMNGAGAAGGAVVPKLYPALMSPNTITNVIPDAR;IVKAHMFKPK;LKEEDGWDVATTEK;LQPAPLDIK;LQPAPLDIKGGRPMVR;LYPALMSPNTITNVIPDAR;LYPALMSPNTITNVIPDARNGVHSTDR;MELHFEFM;MTSVVLNFSSPLTGEYVLQMLNALNPTK;NGVHSTDR;SSVMVIGLSEVFMQQTSVTFR;TIIGDLGSLQGPEGGEWLAFFYPAHTVFEK;VGETPDLSSFSQEEISK | 0.8471 | 64.9942 | 80 | NA |
| 3H-50 | ASCGTIPDAR;ATHYDPIR;ATHYDPIRDGYYMYALPNGR;DGYYMYALPNGR;DYFLSSK;DYFLSSKYK;FLKPTFWFSR;LPSDSGVAVTTSPTVVDDLDLR;NALVALSNSSMNNEQNYTNR;YDNFQCNK;YKDDAR;YNNLSWLLSK | 0.5979 | 42.0638 | 69 | NA |
| 3H-152 | AIYYVSQGK;ASYMNLLIVEQDR;DMVSPSNADGPR;DNTSYFDPLR;DVAVANSDK;EHITFNPGR;EKVVQGATAVR;ELLNPEHSAK;FIYEYNNYTVR;FNLLHDTINSLFEAGVVDR;FSVTVESTRR;FVEHCIIDAYTSNR;FYTEHLHTDELLNINVR;GAVQVGPNAAIEAVMR;GIYSPGMTYDIAHVYPAPGVGYTISVLSNNAPTVDVPR;GTLMSELFTVDER;HDDTNTVIVQYSVDK;HPPTADTALSAMSR;IYDAPNTNVLYGIANDITSHMIQYK;LVYNASLLLR;MSAYETSEIR;MSAYETSEIRK;MVWFPEFVSPVSDDAPR;QLSAHWR;RFNLLHDTINSLFEAGVVDR;RLVYNASLLLR;RVDDAELER;SLSDAVQMVK;SLVIGAEQLER;SQTFYNTQR;SQTIDGVGR;STDTNSATFYPK;SVDLVPDGK;SVDLVPDGKEHITFNPGR;SWVDRDPESNTK;TSPTLILHVWR;TVSLFRPPLSLK;VDDAELER;VLLHLTAYTILK;VLLHLTAYTILKK;VLPLTAFGK;VVQGATAVR;YETDFTSDESPFSIVTSIDK;YGEWLK;YITELLR | 0.3827 | 165.3618 | 65 | NA |
| 3H-58 | AVSPLTNKPIDVHGR;DCNLSPPPTK;EGNLKWLPR;HIVVSNGFQPVR;KLIGTSPIR;LIGTSPIR;LTAAQCETFIR;LTAAQCETFIRNNK;LYNDLTK;MCAGTPNTR;MVYSMCHGVPYR;NDQNVPTYYTSSRPFADR;NESINPITGK;NESINPITGKK;RASPQR;RDCNLSPPPTK;SIRPEQAAYR;SPSPRPYTATSVTR;SQLINIANNMNIAELR;SQLINIANNMNIAELRHIVVSNGFQPVR;SVSPYSGRPIK;TAANADTRPDLCATFSR;VAQNTTQSQLLNIVK;VAQNTTQSQLLNIVKFQIR;VLLSPIPDGATPMSR;YMINPNFVPTTTK;YMINPNFVPTTTKK | 0.4046 | 99.0695 | 61 | cofactor of the virion S/T kinase encoded by thioredoxin |
| 3H-151 | ADEVMDKLVQIDNTVK;ALQYQTQLEGLLSR;ETCALTTVTASLATISESLGK;ETCALTTVTASLATISESLGKDEALLK;ITEVGGGVAAIK;ITEVGGGVAAIKSSLTSVVFTPLSEK;LDAIKSDISGK;LVQIDNTVK;LVQIDNTVKETCALTTVTASLATISESLGK;RVVVDPLDTK;SDISGK;SSLTSVVFTPLSEK;SSLTSVVFTPLSEKLDAIK;SSTTEER;VAFGLTR;VEKALQYQTQLEGLLSR;VVVDPLDTK;VVVDPLDTKVEK;VVVVPVQDK;VVVVPVQDKITEVGGGVAAIK | 0.6404 | 71.5199 | 57 | Uyr/REP helicase |
| 3H-48 | ALLLSQLVSPPLIAK;AMHLLYIEGK;ATQNTVQTLR;CLGQVVTNVR;CSSTAAVDAAVASSSTEALSQK;DINLLQVGLSAADTACISNVTLQAFTK;GSSISTAVSK;LKNWSPK;LQSWVTDHEAVQYGTKPTVPYVIPDNLLSQKPTDNTISR;LSDDRFSINVYTIHDPTKPVAAVSLVGGEAFQSDDIGVTFYNPENYYPPADR;LYGYMANK;NDITAKNITSK;NEIIAKNDITAK;SVASVSSPGNIASSSVGNK;SVASVSSPGNIASSSVGNKR;TANKCLGQVVTNVR;TAQSIMQNLMTQIK;THIFNYLDSK;TVNVNQDSQTIK;TVNVNQDSQTIKTHIFNYLDSK;VDQEVYMK | 0.6070 | 81.4480 | 51 | NA |
| 3H-13 | AAVWLATTTGDDSGYR;ALSSVALTCR;AVMNSFR;CVDEFR;DIGIDTGDPSSVQNRK;DIISPLILLDAER;DPYDTLNK;DVIHEHVVAHWNDAETEQIIGR;ESPTEYFLR;EVMTVPSIVSALK;EWNATFVVIDEAHDLSVHGVDYPK;FAGLVSYLPSIVDVNEVDIVNVGER;GANVAIILGSR;HLNDFEIDR;HTEGLYPMTR;KFAGLVSYLPSIVDVNEVDIVNVGER;LSTHDITVEQFNR;LTTYAEFNETQLLYSDDDDRDR;NIDGALR;NITNDNTIPLDAPR;NTVIVVHDMSDVMSATYHYAGAGER;QRPTFVYPFVLDNVSLYGLDAYR;QRPTFVYPFVLDNVSLYGLDAYRDTLAK;RPMPHQILVANYTTPR;SFIAQLDR;SKLEELGAVITDVTCGVQAK;SPIDGIIVIHGVGTGK;SVINAAER;TFFFNTITAFANTVSR;TVDILMYATSEAK;VIAEGITLK;YALPLYPIAESTDESWSYEHLDK;YAWIAK;YQSHAYTIADWGSDAKPNVFVYR | 0.4947 | 131.6084 | 50 | DEAD-like helicase |
| 3H-27 | ADNVWVYTEDLQAIK;ALINEDGHSVVTQR;AMIESILK;DPALYEVQLHTCR;FNIDNVNYASVAHAIALISVVYHMK;FSVLNSPLSR;FTDEIIK;FTDEIIKK;GVVWSEQSQQLVAAAATTK;HIHSVGDPSYNDEVTR;HITYVVVGHALVSHR;HSNVYSDALESFR;IVTSQLLYK;KVDEFISFGR;LYSTANELLADVLYGDGVDLGSTVSETTNR;NAFTSIIWNVDIFK;NLCEIGVVSNVTDVDLLR;RDPALYEVQLHTCR;RPDAEECFK;RYAIDVNILPAISDTIQK;SLVLHFGANPAYTIMNTFASPAESPR;SLVVLTLQHELR;SLVVLTLQHELRK;SPPTLTIDDDSILYPLQYIPR;STEDDRPIQMQR;TGYANLK;TLFGDVVQK;VDEFISFGR;VLTAALMHTDADTVNPTTPSVGYESDAVPR;VSDPQTTVR;YTDFDQLWSK;YVDMSQYR | 0.5402 | 124.3023 | 47 | NA |
| 3H-110 | ATDINPYETK;FLTNEIK;FLTNEIKADGTAIR;GWVPVNGSDAFEFSCK;HLNPQWATDAPILK;IFEQAGCK;IGFISQILPR;KATDINPYETK;KNVDDDTIELR;LGNVSGIITK;LSFDKK;LTFNGTAK;NVDDDTIELR;TMYAPLSK;TSNKPLIFECILDEPEK;TYKHLNPQWATDAPILK;VVLAPEK;VYEFISR | 0.6995 | 63.6874 | 37 | NA |
| 3H-126 | ALNTHDAGAYK;APVTATPVPVKPLYTAR;DTDITTADNSGVVAK;DTLTLR;ELAGVVGVVCNAVR;ELPIWYDKFEEHVSEYLER;FDFDIK;FHIIPFDSYFTTELDGFLPHGVDR;FTYDIENNDLLSYEK;FVNDFFTSLFPDPEVK;GPSVVTVAK;HLEGFASLLLK;IRDPVATER;ISGSDVSQGLR;IYIDNER;LAESGMGAVPHGDEDANGER;LAVTDEIAGSDDINVGQAK;MSIINSK;NSAPQSGVITQIR;RVTYASIFYDAK;SAEPTTVSR;STAAAITETVAGILK;SVLISNK;TDIDSTVIADK;TDIDSTVIADKHLEGFASLLLK;VFWALK;VFWALKDTVQK;VMYQFNGNIWKPVR;VTYASIFYDAK;WTLEVINWR | 0.3203 | 113.6689 | 36 | ATPase involved in DNA replication |
| 3H-19 | AELEGVADNIK;ATVGALAGDVVKR;DIYIENKAR;EKLVNVTR;ESTTNIAEDIER;ESTTNIAEDIEREPAYLK;GDDVRPELSTFVLSYTR;IFSMLDNEIHLTIDSSDHQHVLQIYNR;ILITTYDYSGVQQR;ISPMYSFHSR;KNTNLYGVTR;LLYWLEDNRFDLLTVDGNDAR;MRPTTADVDNAQPTITVDDLKDVSK;NEQPQNSSSFDK;QIGNGEDANANVEINCGHR;QTLGLVIK;RNEQPQNSSSFDK;RTVLDAAR;SETTDASNVSPR;STVTIEIVDDNHFEHNVECVLDTK;SYADELGDR;VFDVPPVIHGEIFYGPFDVTGLHSYAGPPSLSIIVSNHPSNGSQSK;VLPQHIR;WSYNSLTDGGAFAITGILAR | 0.5402 | 91.5970 | 36 | NA |
| 3H-66 | DIIMGHISPNR;IFLATNTNSHVR;IINDKLK;ISTIPEFHTKPK;KVQVDYSPNYDLTVVTVNDHVSTTK;SIFSSPIPNLGFVAAENGHVR;SLAHEHEEEEVFNDNEK;SLAHEHEEEEVFNDNEKYDDER;SPASDQSYYGESNDGSYK;SPASDQSYYGESNDGSYKR;TADGDYDDDER;TVISGDTYIK;VQVDYSPNYDLTVVTVNDHVSTTK;VTVHEVNADLYLCKPR;YTDIEYK;YTSNNDK;YTSNNDKTVISGDTYIK | 0.4865 | 69.0943 | 34 | NA |
| 3H-136 | DRPIPNQDDWER;EFAVWNLCR;ELPLFPYTIGVMYR;GAAVAVSNINILSR;GVDGGDTFK;HNGLSDLSNILVR;ITITLLAIYNQYK;LAAEMFTDSLILK;LADVDFLVNYR;LAKPQSWFANGHILR;LEEPTIFTVHTR;MDDDANNLGDAYFVPHVK;NISYDWGSFLEAVVSR;NSAVGSQQIR;QPCGLISTTTTSSSTSR;QSMTYDEQSTFK;RLADVDFLVNYR;RLEEPTIFTVHTR;VFGSLAFNTTTVATILNYDR;WNLRPR;YIIHMDGHVAAFR;YSAATSEAYSMR | 0.2650 | 82.7589 | 33 | lipopolysaccharide modifying enzyme |
| 3H-107 | ASLKTNTPLEGVPEFVLFHYGNAVK;FFPAETHVEVK;IDHVPSAVVK;LQLSDWNDNVTTIK;LQLSDWNDNVTTIKK;MMDHVFTLTPSDVTVR;NYVLNEPPAR;RGILSIR;RIDHVPSAVVK;TNTPLEGVPEFVLFHYGNAVK;YASDSLMYFECDLSIR | 0.6810 | 41.7428 | 29 | thioredoxin-like protein |
| 3H-115 | ADDCDISDIISR;ALVIECGIK;DAILKR;DICTPILEK;HNNFTQDHEVTLGAPTASR;KQPSPVTTPSHSTDVR;KSVAASSR;KTPQLQEDLASYTK;LITATEEELK;LITATEEELKALVIECGIK;LVYLEESDEEMRTEDNQS;QCFNTHGAFTMVGTSLK;QGNSSTMVAYGQHHQQQVAVR;TATATAGLFGATPLIGTIAGQVK;TPQLQEDLASYTK;TTTNPLASR;VAGIENLVLQTK;VCSIPTNEDNPDK;VCSIPTNEDNPDKTK;VMFDDEVPSK;YTANVTENESNSTIQSLDEFAK | 0.4167 | 77.4717 | 29 | NA |
| 3H-23 | AVGHVVTIPMDR;DHMIFVK;HKNGVPSNDDLSHRPPYK;IMTVLTSLR;INYAATVNNNAHR;LLHLVR;LVEADVNESTVHEYEHSIK;LVEADVNESTVHEYEHSIKR;RYVFNVDSVK;SPLNYSHENIER;TCFGLVVYK;VIEEIFK;VLPWSTTAAR;VPISYVHNYIMSR;VVVNSDETVYHAR;YDFMSEYLNVK;YGTFAVDSAR;YVFNVDSVK | 0.2326 | 64.6444 | 28 | NA |
| 3H-117 | CIPVVAYHAQDADAPTDTGLK;DDAIIIDDLPDVYITQPHR;DLHDRYT;DYAAVITAELILR;LDSAESEMAR;LEMFLSSDETR;MFAKPEPAIR;NLNALWDLWGADVIIER;NVNGFVPAVIFSDYEVYK;RLEMFLSSDETR;RPHLDEFLSYLFENFR;TAGEEVGGVK;VGVWTAASK | 0.8571 | 49.8937 | 27 | CDT phosphatase transcription factor |
| 3H-64 | AVIGSSGSVNNTVNTVHSR;EMTPSVR;ESGNNTNISPLNR;EVDNYAR;FLGLSGIFSMLADIGNTNK;GGGNSSHIENVIMIPHK;IRSDVIAAKPNYK;LPDVGGVNDVISSK;NIILSGEHDR;QIVDEDDGR;QIVDEDDGRSIESLLGIPSPSKPQSK;QVYGDSLCLEK;SDVIAAKPNYK;SIESLLGIPSPSKPQSK;SKESGNNTNISPLNR;SQWADDYSGR;SVTSDVAALAHSETSR | 0.4309 | 61.9551 | 26 | NA |
| 3H-69 | AAILLGASLVLNK;DESHYMLYTK;DYDTGKDESHYMLYTK;LVYKDYDTGK | 0.4231 | 16.0188 | 24 | NA |
| 3H-116 | CLYIDYQGGK;EHYTLLNTPSATK;ETFYQVFSQVTSVDHR;FGCLEYR;IAFVGKPGTGK;IECSPLPEPGR;IQEDLFKNGR;KIEAQCAYDQINT;LWEFADDFPNK;LWEFADDFPNKETFYQVFSQVTSVDHR;MIDDSLIIPQFHHNLISPR;MLYMVGVQYPK;TCLDGIFLFGTGNHDVR;VTTPVVGSSPSESASSPIR;YIMYTK | 0.4888 | 57.7328 | 23 | ATPase involved in DNA metabolism |
| 3H-135 | ALNMILK;ALNMILKR;DQVPVLTK;KRPIQPTAYNLFYK;QYNPKYLLDNYNYR;RPIQPTAYNLFYK;RPIQPTAYNLFYKDQVPVLTK;SNSNNTMTALDGVHQINSLVTSVFSDSEHINEWLSLSK;YLLDNYNYR | 0.6560 | 34.4082 | 23 | yabby-like transcription factor |
| 3H-62 | ATLEQVFVR;AYVDLKDFVVTHENLFAR;DFVVTHENLFAR;LDNNFVDGLK;LFVPGIDLPQPYIGIDTTK;LFVPGIDLPQPYIGIDTTKR;RLFVPGIDLPQPYIGIDTTK;TQTPVNILADIVSLK;VCINVNAMTAGDK;YTTSTVPNETDR;YTTSTVPNETDRR | 0.3356 | 43.7158 | 22 | myristylated membrane protein-like protein |
| 3H-109 | ALIVKPMNQFR;CVDAVPFK;IDYKETQR;ILSLDAVSVPIDQK;LTFFKK;NSWGEHWGDGGYFK;QAVSSSGCGGGSTYR;SISDDLIR;SQLHFETHESK;SVIPTNATTNAYNAVK;THILNR;VALIMSIDAVIR;VGGAYFVTVR;YLDSLIPTPR | 0.3625 | 49.7616 | 21 | cathepsin B |
| 3H-24 | DFDKLLNYEYFEYIGDSVVNR;EGVLEITNSK;FIIDYIR;HIMNMYLIR;IAEIVVK;IDYDYLVSPQLK;LAQECIEVLTNR;LLNYEYFEYIGDSVVNR;NSVDRPAFK;QFVYGVIGDYAGIINPTYENLNSAR;QLMNAFEINHNIDLNER;SANTTSVNAVNYR;THGFEGSGGSNDSLVNVSR;VNADSVYTDETAANVHR;YGNVDSQYYK | 0.2582 | 58.9843 | 21 | RNaseIII |
| 3H-125 | DDLYNLGIRPDR;ENTTPRPVAIDVR;HSTLLTELAILEER;KLEDLYR;LDSLNVK;LELTTSLIHDTVTVLK;LELTTSLIHDTVTVLKR;LVLENFR;QNILSNIDSTTK;RIFGFK;TTLMQITSTDEHVSEQLLTELTQQLASLETDHSESK;TTSVTLEHAGIR;YDKLDIEFASITSALIEER | 0.1831 | 45.9846 | 20 | ATPase involved in DNA repair |
| 3H-63 | EYVFGVRK;FLFLDEANKPLHEIAVQFTCK;FLILSASGPLK;GSVSDLEFK;GTCAMTLLQQLK;HDKTAFSIVNK;HYTGIIPETMNVAK;LWNASVDSFCYR;NVGLFIPK;SGDEIDGYIIMKPK;SNASAAACCFVGYEFEKDDTTK;YLWTFAPLLEQR | 0.5116 | 46.35468 | 20 | NA |
| 3H-71 | LGKPIVVIAPVR;LGKPIVVIAPVRR;LSESVAVIEAK;LSESVAVIEAKR;NEVAWLLR;NLFTNIR;RLGKPIVVIAPVR;RNLFTNIR;SRNEVAWLLR;VQFPHVVHNPELNVIYWR;YCADIFDIVQHAASL | 0.3654 | 39.4889 | 20 | sulfhydry1 oxidase Erv1 like protein |
| 3H-15 | FFNFKEDFTR;IIPNNESADSR;KTIEALISLPR;LKDPPHTDALK;SNVHIKPVNPPSSK;TIEALISLPR;TIVLPTNR;TLIPLQKPIAPVQISEKPVVVK;VGYHTSDLER;VIQTPDPQPVVEGK;VIVNNPR;YYVDDLVK | 0.4242 | 46.5857 | 18 | NA |
| 3H-182 | APQQIATVK;CDDGELTLHR;DVEFVAR;EGTMTVANHMGSATR;HTGGLIITAPLVHPAVGTTR;RHTGGLIITAPLVHPAVGTTR;SITDMIAAGAPISK;SITLQNTVTK;SLPDPLDIYVR;SSIIHNIQK;TQHHNEEVETVDQSILAHVR;VFECPNTEEVPNFR | 0.3544 | 42.5184 | 18 | NA |
| 3H-28 | GIDDGDAIYTITK;IFMLVLLER;ITALYHNVK;LDEHVPPVAAFIR;LFFDLR;LTEWFPNNR;NNLNYDQLYTALR;NVAEYQSR;QHLLDER;TAYDQADVIAYIDGDKEK;YNGLMHFISK | 0.3249 | 39.2978 | 18 | NA |
| 3H-43 | DLYNLQILR;DQSLELLSHTHR;FVEFGTLVRPAGWGSLSELGK;KDFNVASYESDPLLK;LSLGYQTVVQFYR;LSYDSVVDWER;MFNVDQAIEFFENR;SSPQTVGLEWWK;TLSSMIR;VCHLNAVLDPAFTIDTGNVR;YNSSMDEYRDQSLELLSHTHR | 0.6137 | 46.5188 | 18 | NA |
| 3H-84 | LLSPPVDPHVFIDTTAASIDK;NMVINEPQYQQFLR;NSEEDSSPAQADQSESVGSSPPTK;SIDTNEGNDDDSTAIALR;SPHLDMAFR;TGTEPKPEHSDDGKNSEEDSSPAQADQSESVGSSPPTK;TMEFVR;VVAKPQYDDR | 0.5022 | 31.7419 | 18 | ABC-type transport system permease |
| 3H-118 | EDVIHDR;HLGLKVNGDLR;LDSFVGPTGNFHGMNVTMK;LNGQLHTLVIPK;QVGKPPICLGSDDVLR;RYAYTDSDGVYVK;SIVAAFATADLPTALYHSETATYR;YAYTDSDGVYVK | 0.6071 | 31.6093 | 17 | NA |
| 3H-150 | CLVDLGR;FHNCPNDLTTQIAR;FRVPFAK;LAVEYNGR;LELDCYNEDLR;LQVIFDDNDEK;LQVIFDDNDEKR;NIDLIDVPYTIPIDEIPNFIDK;QHYEYVPR;SADDALLDPTTSPSPVFAR;VRPDWLINPNTK | 0.5150 | 39.5026 | 17 | NA |
| 3H-44 | DNSEASSQNTTVWDR;DNSEASSQNTTVWDRR;TANTSTNTSAAATSGK;TANTSTNTSAAATSGKDNSEASSQNTTVWDR | 0.3168 | 18.1016 | 17 | NA |
| 3H-3 | DYFGWDK;DYFGWDKLEAAYNAK;IIQIAPVDLIK;KDYFGWDK;TLLKPTVHLNR;YANVNGFEGEFNSQYSVQSYNK;YGTIER | 0.3235 | 26.2136 | 16 | NA |
| 3H-46 | HSDDIDTTIDALVK;ISKQLTQLR;KLDQEVLIQEMR;LDQEVLIQEMR;QLTQLR;YMEVSSNDTLLYDNVR | 0.1984 | 24.6879 | 15 | NA |
| 3H-61 | ADVVNATLGTK;AVDLWIR;FVRPLLYNR;GLLGGSLDHAHK;LALALVAFASVR;LYLVSDVFK;NLDYEFSVR;QPTVLVR;SHGVLLNGTPIGYVSDVQR;TLTVTELVLPYSAK;TMVLYPSTAR;TSIATAIDSVRR;TVGVVESISMR;VEETIHHATNHTIGDK | 0.1368 | 48.6104 | 15 | RNA polymerase subunit |
| 3H-113 | ALVVDPPQSVVR;DSSTTDEALK;DVEPSPTMVPK;EWFVQK;FTSVLPPR;LMYDPAILNR;LYNTAFTMATSAVLAPATLTAR;MHAIIPSMVTALTALVSVR;RLDIAFSGTDK;SLGMTGGIR;SMLIDDLNSGFDVVIPPDRR;TTLGGSTDSVMR | 0.4348 | 43.1012 | 14 | lysophospholipid acetyltransferase |
| 3H-37 | AGLLELVIK;IGYTCNLNDR;LHSQVTNLHPLSK;LSELNVASAYDFRPVFVVPTK;LSELNVASAYDFRPVFVVPTKK;QLEAVLHTK;QTLEEQLPR;REFFTLNEDNFTELIK;YWLVNELFPSLK | 0.3472 | 37.6044 | 14 | bro4 |
| 3H-128 | ELAIGIDER;HICNDIVNVFNEK;IATNTDLK;QTSSSQQRPNDLLYPLSYGAVDETVVLK;VVGVTYK;YADIPVWEQGVEDEPTSETNDTLNR;YADIPVWEQGVEDEPTSETNDTLNRDVISDQPR | 0.3657 | 27.8929 | 13 | NA |
| 3H-67 | ALDREGNENALR;FLEALNVHTPQWK;IFEELFK;IFEELFKK;ITISDLNLRR;LIVAHQELR;LLFVGDPHFK;LLFVGDPHFKR;SILQAGGPLLR | 0.1855 | 31.8080 | 13 | DNA repair exonuclease |
| 3H-137 | AMSNGLGDVILSAK;DTMAMIFK;IFDAFKR;KIFDAFK;KVSGDVIDK;NALLTLK;SEDEEVSAEIVTAK;TVRSEDEEVSAEIVTAK;VSTTVDPMMPAQVK;YSVAVAK | 0.5250 | 34.6604 | 12 | hemolysin-like protein |
| 3H-171 | DFLTLFDVDELR;DFLTLFDVDELRDKPK;IASHQLACLNYTLPVR;ILIVINQR;IYSVIGWLCK;LGFQEFVYR;LKDFLTLFDVDELR;LMHYGDAPNDVTPSK;NMTTADLQDIVTLLTK;VIDTFSK | 0.3153 | 36.4329 | 12 | caspase-like protein |
| 3H-77 | MAFVHLFHIDGILECAER;QWATDELKCVENDKPATK;VAALKQWATDELK;VHGWWWR;VQKVHGWWWR | 0.6145 | 18.1176 | 12 | NA |
| 3H-9 | AFSVTATDFLHVGPAIK;IPEFLEK;IPHPLNSNVFIRPK;ISDLLTLLEK;LNEVGFPR;LNNIAGSDDR;LNTILATAK;VLYDVPDEDAKR;VTEHNYEWASSIR | 0.1164 | 33.2419 | 12 | RNA polymerase subunit |
| 3H-156 | DLFNFVSQTR;DTGHLVAFINGGTR;FYDSSTSTCPINYR;IVQFPPGSPYTFVATR;QTVLAQSNSGAYPVER;RQTVLAQSNSGAYPVER | 0.1849 | 21.7121 | 11 | NA |
| 3H-59 | ALDELMDFK;ALDELMDFKK;HVSAEIELK;HVSAEIELKTELDHIK;KDNEISQLHEELDYATETINDLNDQLR;LQSELTMVR;TAAQQLASYR;TELDHIK | 0.4186 | 29.2328 | 11 | putative nucleic |
| 3H-80 | ATIQLLSMILTR;DNGWVEHEATK;GGTTYVTLYPADPNWTFR;KIEDIINLPK;LLDFIRPR;LSLYGLYR;NAGLFQEVER;QNVPSIVEEQR;SLAFTHNVYDVFR;YDGIEVNVPR | 0.2472 | 38.1353 | 11 | RNA polymerase subunit |
| 3H-124 | DLEANASLLR;FSAIKPFTK;GYLLAAIK;IDINRDDDDYLVTDINPFEVNTENAR;INDPVLVIDSAR;LSRPPGIDGDPNK;NSHGDSAYDSSYGSSEAQLSQAAHHMYPNAR;TLLEYNSLYR;TTGYQQLYAELK;VGENSAWHNFETR | 0.2195 | 35.6959 | 10 | serine/threonine protein kinase |
| 3H-139 | AHIAPALLNHIESNLLK;ATLDEVSNDPDDIDVER;ATLDEVSNDPDDIDVERR;FVFSARPTVDSSLASGGVR;QTTVDKWIDSR;VAAYEIAR;VCAAVAR | 0.2888 | 26.9806 | 10 | putative S1/P1 nuclease |
| 3H-21 | ATENELDR;FKEVEDLVLR;IASLLVHDIHSSLDDAVK;IGSIAPIELNPPPR;NVETFVR | 0.2500 | 18.4138 | 10 | NA |
| 3H-82 | ATITEVGNR;DAAYATDAR;DAWTSNTEEASLR;GGHGSVGEVHTSAVVR;LGYMAAGYESTYESVVR;RTVSAVGSNEELANTDDSSR;TLIIQSYSHAIVGDAFIQDR;TVSAVGSNEELANTDDSSR;VCAAFQIKPR | 0.3486 | 35.7064 | 10 | NA |
| 3H-163 | ATSNSTSAGNNITNK;ETAELANR;FGDKEVETYTVDFNGEK;ITLHEDLTR;ITLHEDLTREDLLSAIESTVSSR;KIDDLTLALK;LSVDEKDIVFK;QVAIIVNK | 0.2527 | 24.2245 | 8 | bro22 |
| 3H-76 | EFPLVNPAR;ELYPEHVK;FNEYDATLR;LRELYPEHVK;LVLGSQSPYNAYVLNYGSAENQLR;SLWSTDETLPR;VYLMSVEEAK | 0.1807 | 23.4768 | 8 | NA |
| 3H-142 | EVPTTVSEDSKELSTLHR;FAVPSGK;KTPAISVTR;NSWITVPTGGVLDDLQSVITDNYNSPGLR;QMVDIMTELK | 0.3782 | 18.8421 | 7 | NA |
| 3H-16 | LGNVLFWNDLLK;LVAGGLIVIR;NQGVNDVLHAIDWTIENAR;QLADGLGFILSK;TANEQPVLFTNTLNEYHNFVYGSTVFTHAR;YLSFVEPK | 0.1389 | 23.1716 | 7 | lipase like protein |
| 3H-26 | ANSIIDNIER;QIVDPNATMK;SGATYAINNSYTIDALR;TSEIVDVVDATR;TSEIVDVVDATRK | 0.3731 | 20.1159 | 7 | NA |
| 3H-4 | LSHDYAYSLR;MDDAPKDFNEFIVWVPTK;NQPNNYVMVR;NSDFAWK;NVTFYVTIK;SSTVVSTVSSATWVK;VYTNVSDIPSAEK | 0.3475 | 24.7869 | 7 | NA |
| 3H-7 | DTVELFK;FFADTYATYR;FQTQVTQLFR;NLVYTLR;SDPTGAFIEYPFGSLHTITAAVYR;SIIVLGADTLNLTNK | 0.1776 | 19.7477 | 7 | NA |
| 3H-108 | AEHLQQFTR;KLNVDFSVK;LNGVLDVK;QNELHTGETVLHLQSR;VGSTEMLIEVAR | 0.2523 | 17.5014 | 6 | NA |
| 3H-129 | LREYFENHGVR;LVALTTSVR;SAYDGIITTFK;SFLLHR;VVVTATQAVAAQLVSGK;YSISSDFVK | 0.1240 | 20.1122 | 6 | helicase |
| 3H-134 | IITHEEYVNLEADFESYKK;ISNILSPK;SVTSFAAFTDNDHEPPPK;VATYPPPTSNDESASHER | 0.2877 | 15.0900 | 6 | NA |
| 3H-157 | DLPDTVPVSLDVR;EQITQWLR;GINDLSDAELAYK;NPPLELWSR;TLLLHYR;VLIDTEQKR | 0.3410 | 22.3261 | 6 | tyrosyl-DNA phosphodiesterase |
| 3H-181 | HIPSADDPGADLFTNWFSINAHDR;HIPSADDPGADLFTNWFSINAHDRQEIR;WYHLDCFLSK | 0.3423 | 11.3437 | 6 | NA |
| 3H-29 | ISPIFDGSVAILHDLSSGLVAAAISK;NHGTSVSIVGDDHK;VFNDINVVK | 0.5000 | 13.5762 | 6 | NA |
| 3H-83 | AVTVTKGIAR;ESFGTYMGQR;KPAQELIHEYR;MSPELQEAWR | 0.2697 | 14.3167 | 6 | glycosyltransferase |
| 3H-90 | DLHRLELPEETFIK;LELPEETFIK;TAEAWYK;TGASTGYSSDSPSR;YDMLELLK | 0.1581 | 17.0098 | 6 | *iap*-like protein-5 |
| 3H-22 | DSHEIYINAWK;HASNDALNAIVYR;KVTVNTDYFVLFEDK;NHYDDTLYDTSVK | 0.2653 | 15.7864 | 5 | NA |
| 3H-72 | EFEYFK;ELQPPYSNYFEESLKPEAFVSNIHYK;LSSLLTSIVSSHSGMIHDKR;QEYEDDDYNFK;VTTSSILR | 0.4581 | 15.2553 | 5 | phenylalanyl-Trna synthetase α subunit |
| 3H-78 | FWYGFLSNK;GLSTFIAK;LEQYIDKK;TYDWIR;VLYSISLPR | 0.1212 | 14.7834 | 5 | nuclease |
| 3H-79 | FPFWFER;RNEPVSYLAVGNAQFIDR;SGLNYIITFDSSATEK;VPVSNTVEFVIVILK | 0.4480 | 15.3581 | 5 | NA |
| 3H-88 | ASFDTVPWVDGR;FVSNTQQLVENSLIYGGLR;NIIPTPLLHHFER;QISHVVYADWLTER | 0.2231 | 16.6785 | 5 | putative S1/P1 nuclease |
| 3H-92 | DKIIEYIER;KLILVGLQR;LILVGLQR;LYRPDSETVKK;NVIPITPVLFNR | 0.0624 | 15.9945 | 5 | NA |
| 3H-149 | GFDNFTNINENFYK;NVSSLDMINITTEEQR;TTIEDVQK;WLDVQLK | 0.3261 | 14.5491 | 4 | NA |
| 3H-42 | EITLYEHAK;ELYDGFTSDVR;VTSVEIK;YGTLSGIPALFR | 0.1667 | 10.4453 | 4 | NA |
| 3H-54 | FNNFITLGTTR;GLAHSFENLR;HSGESLTNYLLR | 0.1352 | 11.0651 | 4 | NA |
| 3H-70 | CEYVTK;VLSEMAVCTKR;YSGAGKPVSPNDFLMR | 0.3929 | 9.0473 | 4 | NA |
| 3H-81 | HASWLFSYLQK;LFSTSTNR;LTMYHTALFR | 0.1189 | 7.9907 | 4 | NA |

Table S9 Identified HvAV-3h coded proteins from *Spodoptera exigua* produced virions (Se-2)

| Protein ID | Identified peptide sequence | Sequence coverage | Protein Q score | Unique spectra | Function annotation |
| --- | --- | --- | --- | --- | --- |
| 3H-57 | AKLDLVTINSVANK;ALLEVTDNVVTDR;ALLEVTDNVVTDRVPVR;AQLDVADLER;AQLDVADLERR;AQLQSDLDVLR;ATIDALTANLNSSNSK;CQAELQQR;DEAIWAIK;DQLLKLWR;DSSFPIVDADNTEQLVVR;EHDVLER;EKAQLQSDLDVLR;EKQHAEFLR;ELACNVIPEKCGDITQMNR;EMYAVLEDELK;GELNAMTR;GGMGVLDDIYNR;HENLYLASTTPSR;HIAGVITK;HVGNAQSDGMYTENVALR;IESEAALVESLQR;IQAFVNTEQNILAAGTR;ISELLTQLQESNNQR;ITNEFFDHATLPLLR;KQMGNVVQEIAQR;KTNDNLEIK;LDGSDVTLVVDDENLK;LDLVTINSVANK;LDLVTINSVANKVNR;LIDSVVK;LIDSVVKITR;LLISSETLGR;LLKEHDVLER;LLSLDGVR;LLSLDGVRR;LLVSEDK;LNDIAELQSQLTK;LNKELNTVR;LNNELETSITEER;LQDEIK;LQLDYMTLQQR;LSNASVGVQLDR;LTNECQAVSSR;MEFEAVNDALSK;MLELQNLMK;MTAAHLADDR;MTAAHLADDRLSNASVGVQLDR;NAEITNLQDR;NELAATVNSLQMQATK;NIDGAEGFTR;NISAIRPR;NNVQLQTALQR;NSGVGNAINDR;NVNDTTLAELAQSR;QHAEFLR;QHPSFANKPNNTDVQQSDLTIAEDADTGTSVTK;QIDSLRR;QLNALNLR;QMGNVVQEIAQR;QQLEWILVNSNPAIALQDASVR;QSLLETSAQLNDVR;RLNNELETSITEER;RNNVQLQTALQR;RPIRPMADNFAEGYVYERPMGGGAPR;RPSVTDDEDDRIVGTR;SDVVVLR;SETNALVVEVADLK;SETNALVVEVADLKSQLNR;SEYELSASR;SLSYSLTDDEDDAAQIR;SNANLSPQSIR;SRSDVVVLR;SVTGPIR;SYIIELESR;TKEMYAVLEDELK;TNDNLEIK;TQLLTGLR;TYQQEMTQLK;TYQQEMTQLKR;VITGDETVMTYNR;VSTDDLTSYSR;YDNYNRNEHELR;YEALMSEQSSK | 0.5787 | 323.6209 | 172 | chromosome segregation SMC like protein |
| 3H-53 | ALFFGVR;DSGVALPIGMMVNNR;EIRPCAAFSK;GQYMYSFALDLK;GSINPSNFNSSISVVIEPSEALR;GTIDSYIYGPDEQVTTYFVR;KIDNGGDFN;KLSDLLIK;LSDLLIK;MPVLLSTGNGSNK;NITHSEYLDYYEVGLPK;NITHSEYLDYYEVGLPKSNNGVR;RGTIDSYIYGPDEQVTTYFVR;SDVNKR;SIGVSALSKIGIK;SVDIGAKTLVLPIPLYFSR;SYAAGTESSYFPK;TLVLPIPLYFSR;TSCSPHYILMEKPTDIAATEILKPTEAGSTLSYDIEHSSGILK;VPMLPAEHFVFTEPYR;VTVEFVFR;VTVEFVFRK;YRVPMLPAEHFVFTEPYR | 0.5714 | 94.5770 | 103 | major capsid protein |
| 3H-140 | ALSHPDWLTIGSFIK;ALSHPDWLTIGSFIKETNYPIPYFQYNLVLSQR;DVDGVASNGGGSVGTGLGGPLGGTSGVNTSNLR;ELFYHSEAGR;ENFVNLVELWCK;ETNYPIPYFQYNLVLSQR;EVAKELFYHSEAGR;NSDKDVTIPK;SGDEMTLSR;STGTTVGYTGTTSNAAMK;STGTTVGYTGTTSNAAMKDVDGVASNGGGSVGTGLGGPLGGTSGVNTSNLR;TATHLLGYK;TATHLLGYKNK;TDVNATNKSTGTTVGYTGTTSNAAMK;TLDRPTDNAIMQR;YTYMYVNK;YTYMYVNKR | 0.8019 | 70.6541 | 65 | NA |
| 3H-55 | AYELANER;AYELANERGPFSVEVYLNPGTSNTYQYVATTR;DSYRLPQSFTR;FNNTDYDDLPWNYTSGK;FNNTDYDDLPWNYTSGKK;GIASLWGYLK;GPFSVEVYLNPGTSNTYQYVATTR;IGQTPKAYELANER;KVVTATGVVSGGER;LPQSFTR;LPQSFTRDPLFR;NKFNNTDYDDLPWNYTSGK;SEIAQDIQVTMYNTNGGSNPLNNSNVTR;SNVDSSSYYQQPPQVVYNNGDLYGSR;TGYSGAELGASIDK;TGYSGAELGASIDKGIASLWGYLK;VVTATGVVSGGER;YVFLLR;YYYMSRPIGFGSSGAYDVPLLDTPLLR | 0.8800 | 79.6302 | 61 | NA |
| 3H-50 | ASCGTIPDAR;ATHYDPIR;ATHYDPIRDGYYMYALPNGR;DGYYMYALPNGR;DYFLSSK;FLKPTFWFSR;LPSDSGVAVTTSPTVVDDLDLR;LPSDSGVAVTTSPTVVDDLDLRIR;NALVALSNSSMNNEQNYTNR;YDNFQCNK;YNNLSWLLSK | 0.5767 | 42.3594 | 56 | NA |
| 3H-56 | DMNGIAVSVDAK;EEDGWDVATTEK;IISETVK;IMNGAGAAGGAVVPK;LKEEDGWDVATTEK;LQPAPLDIK;LQPAPLDIKGGRPMVR;LYPALMSPNTITNVIPDAR;LYPALMSPNTITNVIPDARNGVHSTDR;MELHFEFM;SSVMVIGLSEVFMQQTSVTFR;TIIGDLGSLQGPEGGEWLAFFYPAHTVFEK;VGETPDLSSFSQEEISK | 0.6901 | 48.9324 | 54 | NA |
| 3H-151 | ALQYQTQLEGLLSR;ETCALTTVTASLATISESLGK;ETCALTTVTASLATISESLGKDEALLK;ITEVGGGVAAIK;ITEVGGGVAAIKSSLTSVVFTPLSEK;LDAIKSDISGK;LVQIDNTVK;LVQIDNTVKETCALTTVTASLATISESLGK;RVVVDPLDTK;SSLTSVVFTPLSEK;SSLTSVVFTPLSEKLDAIK;VAFGLTR;VEKALQYQTQLEGLLSR;VVVDPLDTK;VVVDPLDTKVEK;VVVVPVQDK;VVVVPVQDKITEVGGGVAAIK | 0.5714 | 69.2617 | 46 | Uyr/REP helicase |
| 3H-27 | ALINEDGHSVVTQR;DPALYEVQLHTCR;DSANSLLK;FNIDNVNYASVAHAIALISVVYHMK;FTDEIIKK;GVVWSEQSQQLVAAAATTK;HIHSVGDPSYNDEVTR;HITYVVVGHALVSHR;HSNVYSDALESFR;HSNVYSDALESFRR;IVTSQLLYK;KADNVWVYTEDLQAIK;KVDEFISFGR;LYSTANELLADVLYGDGVDLGSTVSETTNR;NAFTSIIWNVDIFK;NLCEIGVVSNVTDVDLLR;RDPALYEVQLHTCR;RPDAEECFK;RYAIDVNILPAISDTIQK;SLVLHFGANPAYTIMNTFASPAESPR;SLVVLTLQHELR;SLVVLTLQHELRK;SPPTLTIDDDSILYPLQYIPR;TGYANLK;TLFGDVVQK;VDEFISFGR;VLTAALMHTDADTVNPTTPSVGYESDAVPR;YTDFDQLWSK;YVDMSQYR;YVPINR | 0.5086 | 123.1987 | 45 | NA |
| 3H-48 | ALLLSQLVSPPLIAK;AMHLLYIEGK;AMHLLYIEGKYLR;ATQNTVQTLR;CLGQVVTNVR;CSSTAAVDAAVASSSTEALSQK;GSSISTAVSK;LQSWVTDHEAVQYGTKPTVPYVIPDNLLSQKPTDNTISR;LSDDRFSINVYTIHDPTKPVAAVSLVGGEAFQSDDIGVTFYNPENYYPPADR;LYGYMANK;NDITAKNITSK;NEIIAKNDITAK;SVASVSSPGNIASSSVGNKR;TAQSIMQNLMTQIK;THIFNYLDSK;TVNVNQDSQTIK;TVNVNQDSQTIKTHIFNYLDSK | 0.5185 | 71.4670 | 41 | NA |
| 3H-152 | AIYYVSQGK;ASYMNLLIVEQDR;DNTSYFDPLR;EHITFNPGR;EKVVQGATAVR;FIYEYNNYTVR;FVEHCIIDAYTSNR;FYTEHLHTDELLNINVR;GTLMSELFTVDER;HDDTNTVIVQYSVDK;HDDTNTVIVQYSVDKHYLSR;HPPTADTALSAMSR;LIGMDGVQLETIR;LVYNASLLLR;MVWFPEFVSPVSDDAPR;RLVYNASLLLR;SEELDILK;SLVIGAEQLER;SQTFYNTQR;SVDLVPDGK;SWVDRDPESNTK;TSPTLILHVWR;TVSLFRPPLSLK;VDDAELER;VLLHLTAYTILK;VLPLTAFGK;VVQGATAVR;YETDFTSDESPFSIVTSIDK;YGEWLK;YITELLR | 0.2514 | 116.0016 | 39 | NA |
| 3H-58 | DCNLSPPPTK;HIVVSNGFQPVR;LGGLSPYR;LIGTSPIR;LYNDLTK;MVYSMCHGVPYR;NDQNVPTYYTSSRPFADR;NESINPITGK;NESINPITGKK;SIRPEQAAYR;SPSPRPYTATSVTR;SQLINIANNMNIAELR;SVSPYSGRPIK;SVSPYSGRPIKR;TAANADTRPDLCATFSR;VAQNTTQSQLLNIVK;VAQNTTQSQLLNIVKFQIR;VLLSPIPDGATPMSR;YMINPNFVPTTTK;YMINPNFVPTTTKK | 0.3312 | 72.0304 | 38 | cofactor of the virion S/T kinase encoded by thioredoxin |
| 3H-13 | AAVWLATTTGDDSGYR;ALSSVALTCR;DIGIDTGDPSSVQNR;DIISPLILLDAER;DVIHEHVVAHWNDAETEQIIGR;ESPTEYFLR;EWNATFVVIDEAHDLSVHGVDYPK;GANVAIILGSR;HLNDFEIDR;HTEGLYPMTR;KFAGLVSYLPSIVDVNEVDIVNVGER;LSTHDITVEQFNR;LTTYAEFNETQLLYSDDDDRDR;NIDGALR;NITNDNTIPLDAPR;QRPTFVYPFVLDNVSLYGLDAYR;SFIAQLDR;SFLCLTR;SKLEELGAVITDVTCGVQAK;SPIDGIIVIHGVGTGK;TFFFNTITAFANTVSR;TSDVVLHK;VDSLLKMFNIDPNSTYSLELLFVIMNTIQK;VIAEGITLK;YALPLYPIAESTDESWSYEHLDK;YQSHAYTIADWGSDAKPNVFVYR | 0.4280 | 98.2803 | 33 | DEAD-like helicase |
| 3H-19 | AELEGVADNIK;ATVGALAGDVVKR;DIYIENK;EKLVNVTR;ESTTNIAEDIER;ESTTNIAEDIEREPAYLK;GDDVRPELSTFVLSYTR;IFSMLDNEIHLTIDSSDHQHVLQIYNR;ISPMYSFHSR;KNTNLYGVTR;LLYWLEDNRFDLLTVDGNDAR;NEQPQNSSSFDK;NTNLYGVTR;QTLGLVIK;SHVDDNEDLAPSKR;SYADELGDR;TPSPTAVEVNTLAR;VFDVPPVIHGEIFYGPFDVTGLHSYAGPPSLSIIVSNHPSNGSQSK;VLPQHIR;WSYNSLTDGGAFAITGILAR | 0.4211 | 74.6263 | 26 | NA |
| 3H-135 | ALNMILK;DQVPVLTK;EFPNISCR;KRPIQPTAYNLFYK;RPIQPTAYNLFYK;RPIQPTAYNLFYKDQVPVLTK;SNSNNTMTALDGVHQINSLVTSVFSDSEHINEWLSLSK;YLLDNYNYR | 0.6720 | 32.3292 | 25 | yabby-like transcription factor |
| 3H-110 | ATDINPYETK;FLTNEIK;FLTNEIKADGTAIR;GWVPVNGSDAFEFSCK;HLNPQWATDAPILK;IGFISQILPR;KATDINPYETK;KNVDDDTIELR;LGNVSGIITK;LSFDKK;LTFNGTAK;MAGLPLHAK;NVDDDTIELR;TSNKPLIFECILDEPEK;TYKHLNPQWATDAPILK;VYEFISR | 0.6338 | 56.2896 | 24 | NA |
| 3H-66 | DIIMGHISPNR;DTFAVSPAR;IFLATNTNSHVR;IINDKLK;ISTIPEFHTKPK;KVQVDYSPNYDLTVVTVNDHVSTTK;SALVNCYAK;SIFSSPIPNLGFVAAENGHVR;SLAHEHEEEEVFNDNEK;SLAHEHEEEEVFNDNEKYDDER;SPASDQSYYGESNDGSYKR;TADGDYDDDER;TVISGDTYIK;VQVDYSPNYDLTVVTVNDHVSTTK;VTVHEVNADLYLCKPR;YTDIEYK;YTSNNDKTVISGDTYIK | 0.5351 | 64.0919 | 23 | NA |
| 3H-126 | ALNTHDAGAYK;APVTATPVPVKPLYTAR;DTDITTADNSGVVAK;DTLTLR;ELAGVVGVVCNAVR;ELPIWYDKFEEHVSEYLER;EYFLLSVAQIFR;FDFDIK;GPSVVTVAK;HLEGFASLLLK;IRDPVATER;ISGSDVSQGLR;IYIDNER;LAVTDEIAGSDDINVGQAK;NSAPQSGVITQIR;RVTYASIFYDAK;TDIDSTVIADK;VFWALK;WTLEVINWR | 0.2063 | 69.9913 | 22 | ATPase involved in DNA replication |
| 3H-117 | CIPVVAYHAQDADAPTDTGLK;DDAIIIDDLPDVYITQPHR;DYAAVITAELILR;NLNALWDLWGADVIIER;NVNGFVPAVIFSDYEVYK;RLEMFLSSDETR;RPHLDEFLSYLFENFR;SRPTVFLDLDNTLICSLR;TAGEEVGGVK;VGVWTAASK | 0.8095 | 42.2090 | 21 | CDT phosphatase transcription factor |
| 3H-109 | ALIVKPMNQFR;IAQYPINTK;IDYKETQR;ILSLDAVSVPIDQK;KSISDDLIR;LTFFKK;NSWGEHWGDGGYFK;QAVSSSGCGGGSTYR;SISDDLIR;SQLHFETHESK;SVIPTNATTNAYNAVK;VALIMSIDAVIR;VGGAYFVTVR;YLDSLIPTPR | 0.3504 | 54.3367 | 20 | cathepsin B |
| 3H-62 | ATLEQVFVR;AYVDLKDFVVTHENLFAR;DFVVTHENLFAR;INTGSITDSVQCFNK;KTQTPVNILADIVSLK;LDNNFVDGLK;LFVPGIDLPQPYIGIDTTK;LFVPGIDLPQPYIGIDTTKR;LITDPYQLLQISER;RLFVPGIDLPQPYIGIDTTK;TQTPVNILADIVSLK;YTTSTVPNETDRR | 0.3938 | 50.6927 | 20 | myristylated membrane protein-like protein |
| 3H-71 | LGKPIVVIAPVR;LGKPIVVIAPVRR;LSESVAVIEAK;LSESVAVIEAKR;NEVAWLLR;NLFTNIR;RLGKPIVVIAPVR;RNLFTNIR;SDSLDACLTFIDIVLDSHR;SRNEVAWLLR;VQFPHVVHNPELNVIYWR | 0.3846 | 39.0685 | 20 | sulfhydry1 oxidase Erv1 like protein |
| 3H-115 | DICTPILEK;HNNFTQDHEVTLGAPTASR;KQPSPVTTPSHSTDVR;KTPQLQEDLASYTK;LESVENVINAFNLR;LVYLEESDEEMRTEDNQS;QGNSSTMVAYGQHHQQQVAVR;RADDCDISDIISR;TATATAGLFGATPLIGTIAGQVK;TPQLQEDLASYTK;TTTNPLASR;VAGIENLVLQTK;VCSIPTNEDNPDK;VMFDDEVPSK;YTANVTENESNSTIQSLDEFAK | 0.3550 | 60.5228 | 18 | NA |
| 3H-116 | CLYIDYQGGK;DLPLTLR;EHYTLLNTPSATK;ETFYQVFSQVTSVDHR;IAFVGKPGTGK;IECSPLPEPGR;IQEDLFKNGR;KIEAQCAYDQINT;LWEFADDFPNK;LWEFADDFPNKETFYQVFSQVTSVDHR;MIDDSLIIPQFHHNLISPR;TCLDGIFLFGTGNHDVR;VTTPVVGSSPSESASSPIR;YIMYTK | 0.4579 | 51.4713 | 18 | ATPase involved in DNA metabolism |
| 3H-136 | DRPIPNQDDWER;EFAVWNLCR;ELPLFPYTIGVMYR;GAAVAVSNINILSR;GVDGGDTFK;HNGLSDLSNILVR;LAAEMFTDSLILK;LAKPQSWFANGHILR;LEEPTIFTVHTR;QSMTYDEQSTFK;TFEEFR;VFGSLAFNTTTVATILNYDR;WNLRPR;YSAATSEAYSMR | 0.1695 | 48.7982 | 17 | lipopolysaccharide modifying enzyme |
| 3H-64 | AVIGSSGSVNNTVNTVHSR;ESGNNTNISPLNR;FLGLSGIFSMLADIGNTNK;ISSPTAVK;QIVDEDDGR;SDVIAAKPNYK;SIESLLGIPSPSKPQSK;SKESGNNTNISPLNR;SQWADDYSGR;SVTSDVAALAHSETSR;VMLVEYYALLK | 0.3162 | 41.1812 | 17 | NA |
| 3H-24 | DRLQIASK;EGVLEITNSK;FIIDYIR;IAEIVVK;IDYDYLVSPQLK;IYSGWTR;LAQECIEVLTNR;NSVDRPAFK;QFVYGVIGDYAGIINPTYENLNSAR;SANTTSVNAVNYR;THGFEGSGGSNDSLVNVSR;VNADSVYTDETAANVHR;YGNVDSQYYK | 0.2143 | 45.0881 | 15 | RNaseIII |
| 3H-37 | AGLLELVIK;EFFTLNEDNFTELIK;FQFNSQELEVIGVK;IGYTCNLNDR;ILEYSNAPNAIAK;LHSQVTNLHPLSK;LSELNVASAYDFRPVFVVPTK;LSELNVASAYDFRPVFVVPTKK;QLEAVLHTK;QTLEEQLPR;YWLVNELFPSLK | 0.4375 | 44.9033 | 15 | bro4 |
| 3H-46 | HSDDIDTTIDALVK;ISKQLTQLR;KLDQEVLIQEMR;QLTQLR;YMEVSSNDTLLYDNVR | 0.1984 | 19.0018 | 15 | NA |
| 3H-107 | FFPAETHVEVK;IDHVPSAVVK;LQLSDWNDNVTTIK;LQLSDWNDNVTTIKK;NYVLNEPPAR;RIDHVPSAVVK;SPYANAAR;TNTPLEGVPEFVLFHYGNAVK | 0.4663 | 27.8656 | 14 | thioredoxin-like protein |
| 3H-125 | DDLYNLGIRPDR;ENTTPRPVAIDVR;HSTLLTELAILEER;KLEDLYR;LDSLNVK;LELTTSLIHDTVTVLK;LSDPLRDVISK;LVLENFR;QNILSNIDSTTK;TTSVTLEHAGIR | 0.1254 | 37.8786 | 14 | ATPase involved in DNA repair |
| 3H-15 | FFNFKEDFTR;KTIEALISLPR;LKDPPHTDALK;TIEALISLPR;TIVLPTNR;TLIPLQKPIAPVQISEKPVVVK;VIQTPDPQPVVEGK;VIVNNPR;YYVDDLVK | 0.3064 | 30.3997 | 14 | NA |
| 3H-43 | DFNVASYESDPLLK;DLYNLQILR;DQSLELLSHTHR;FGLASVNDENSYK;FVEFGTLVRPAGWGSLSELGK;KDFNVASYESDPLLK;LSLGYQTVVQFYR;LSYDSVVDWER;MFNVDQAIEFFENR;VCHLNAVLDPAFTIDTGNVR | 0.5494 | 42.7635 | 14 | NA |
| 3H-63 | DNKYLWTFAPLLEQR;EYVFGVR;FLFLDEANKPLHEIAVQFTCK;FLILSASGPLK;GSVSDLEFK;HYTGIIPETMNVAK;LWNASVDSFCYR;NVGLFIPK;SGDEIDGYIIMKPK;TAFSIVNK;VNAEDDCGVEAK;YLWTFAPLLEQR | 0.4352 | 41.0723 | 14 | NA |
| 3H-1 | ELAAHITSK;ELDWHGVITVR;LPFAGWIETGR;LSSVDLQR;QIINPLTK;QLNYSGAYVK;TFAWDIEAK;TILQSIDIAQR;TSNPDDLPIEEISYFK;VPQHSHHDILYNTK;YFKPTSTTR | 0.1068 | 39.1687 | 13 | DNA polymerase |
| 3H-69 | AAILLGASLVLNK;DYDTGKDESHYMLYTK;LVYKDYDTGK | 0.4231 | 11.9772 | 13 | NA |
| 3H-118 | EDVIHDR;LNGQLHTLVIPK;RYAYTDSDGVYVK;SIVAAFATADLPTALYHSETATYR;YAYTDSDGVYVK | 0.3333 | 19.5564 | 12 | NA |
| 3H-128 | DVISDQPR;ELAIGIDER;IATNTDLK;KQTSSSQQRPNDLLYPLSYGAVDETVVLK;LIAAVGGIVIALGVLLKR;QTSSSQQRPNDLLYPLSYGAVDETVVLK;YADIPVWEQGVEDEPTSETNDTLNR | 0.3619 | 25.3055 | 12 | NA |
| 3H-182 | APQQIATVK;CDDGELTLHR;DVEFVAR;HTGGLIITAPLVHPAVGTTR;RHTGGLIITAPLVHPAVGTTR;SITDMIAAGAPISK;SLPDPLDIYVR;SSIIHNIQK;VFECPNTEEVPNFR | 0.2405 | 32.4080 | 12 | NA |
| 3H-23 | IMTVLTSLR;INYAATVNNNAHR;LLHLVR;LVEADVNESTVHEYEHSIK;LVEADVNESTVHEYEHSIKR;SPLNYSHENIER;VVVNSDETVYHAR;YGTFAVDSAR;YVFNVDSVK | 0.1189 | 35.3771 | 12 | NA |
| 3H-3 | DYFGWDK;DYFGWDKLEAAYNAK;IIQIAPVDLIK;KDYFGWDK;TLLKPTVHLNR;YANVNGFEGEFNSQYSVQSYNK;YGTIER | 0.3235 | 25.8068 | 12 | NA |
| 3H-137 | AMSNGLGDVILSAK;DTMAMIFK;IFDAFK;IFDAFKR;KIFDAFK;NALLTLK;SEDEEVSAEIVTAK;TGVDLSVNVGQTYK;VSTTVDPMMPAQVK;YSVAVAK | 0.5375 | 32.4176 | 11 | hemolysin-like protein |
| 3H-28 | GIDDGDAIYTITK;IFMLVLLER;ITALYHNVK;LDEHVPPVAAFIR;LFFDLR;LTEWFPNNR;NVAEYQSR;TAYDQADVIAYIDGDKEK | 0.2401 | 28.7447 | 11 | NA |
| 3H-84 | ELMQYIR;LLSPPVDPHVFIDTTAASIDK;NSEEDSSPAQADQSESVGSSPPTK;SIDTNEGNDDDSTAIALR;SPHLDMAFR;TGTEPKPEHSDDGKNSEEDSSPAQADQSESVGSSPPTK;TMEFVR | 0.4286 | 27.2076 | 11 | ABC-type transport system permease |
| 3H-139 | AHIAPALLNHIESNLLK;ATLDEVSNDPDDIDVER;ATLDEVSNDPDDIDVERR;FVFSARPTVDSSLASGGVR;NKFDAK;QTTVDKWIDSR;VAAYEIAR | 0.2852 | 25.5147 | 10 | putative S1/P1 nuclease |
| 3H-44 | DNSEASSQNTTVWDR;DNSEASSQNTTVWDRR;TANTSTNTSAAATSGK;TANTSTNTSAAATSGKDNSEASSQNTTVWDR | 0.3168 | 16.3753 | 10 | NA |
| 3H-82 | DAAYATDAR;DAWTSNTEEASLR;GGHGSVGEVHTSAVVR;LGYMAAGYESTYESVVR;RTVSAVGSNEELANTDDSSR;RVCAAFQIKPR;TVSAVGSNEELANTDDSSR;VCAAFQIKPR;YDGEALTR | 0.2875 | 33.9590 | 10 | NA |
| 3H-9 | AFSVTATDFLHVGPAIK;IPEFLEK;IPHPLNSNVFIRPK;ISDLLTLLEK;LNEVGFPR;LNTILATAK;VLYDVPDEDAKR;VTEHNYEWASSIR | 0.1048 | 29.7477 | 10 | RNA polymerase subunit |
| 3H-150 | LAVEYNGR;LQVIFDDNDEK;LQVIFDDNDEKR;NIDLIDVPYTIPIDEIPNFIDK;QGVNTLAEYGK;QHYEYVPR;SADDALLDPTTSPSPVFAR;VRPDWLINPNTK | 0.3948 | 35.1844 | 9 | NA |
| 3H-67 | FLEALNVHTPQWK;IFEELFKK;ITISDLNLR;LIVAHQELR;LLFVGDPHFK;LLFVGDPHFKR;SILQAGGPLLR;SLPTGVGFAFK | 0.1805 | 28.0462 | 9 | DNA repair exonuclease |
| 3H-80 | ATIQLLSMILTR;GGTTYVTLYPADPNWTFR;KIEDIINLPK;LLDFIRPR;LSLYGLYR;NAGLFQEVER;QNVPSIVEEQR;SLAFTHNVYDVFR | 0.2004 | 31.7091 | 9 | RNA polymerase subunit |
| 3H-157 | DLPDTVPVSLDVR;EQITQWLR;GINDLSDAELAYK;NPPLELWSR;TLLLHYR;VLIDTEQKR | 0.3410 | 22.0472 | 8 | tyrosyl-DNA phosphodiesterase |
| 3H-79 | FPFWFER;NEPVSYLAVGNAQFIDR;RNEPVSYLAVGNAQFIDR;SGLNYIITFDSSATEK;SPNCALVFTR;VPVSNTVEFVIVILK | 0.5280 | 25.1714 | 8 | NA |
| 3H-124 | GYLLAAIK;IDINRDDDDYLVTDINPFEVNTENAR;INDPVLVIDSAR;LSRPPGIDGDPNK;TLLEYNSLYR;TTGYQQLYAELK | 0.1235 | 22.9557 | 7 | serine/threonine protein kinase |
| 3H-129 | GVDDSIWTDSQK;LVALTTSVR;NVVDVQR;SAYDGIITTFK;SFLLHR;VVVTATQAVAAQLVSGK;VVVVQPQSYR | 0.1417 | 21.3422 | 7 | helicase |
| 3H-156 | DLFNFVSQTR;DTGHLVAFINGGTR;FYDSSTSTCPINYR;IVQFPPGSPYTFVATR;QTVLAQSNSGAYPVER | 0.1823 | 18.3644 | 7 | NA |
| 3H-171 | DFLTLFDVDELR;ILIVINQR;IYSVIGWLCK;LKDFLTLFDVDELR;LMHYGDAPNDVTPSK | 0.1497 | 21.9902 | 7 | caspase-like protein |
| 3H-61 | ADVVNATLGTK;AIEDNVR;AVDLWIR;FIGSLIK;FVRPLLYNR;TLTVTELVLPYSAK;TSIATAIDSVR | 0.0571 | 24.1633 | 7 | RNA polymerase subunit |
| 3H-21 | ATENELDR;FKEVEDLVLR;IASLLVHDIHSSLDDAVK;IGSIAPIELNPPPR | 0.2193 | 14.0603 | 6 | NA |
| 3H-26 | ANSIIDNIER;QIVDPNATMK;SGATYAINNSYTIDALR;TSEIVDVVDATRK | 0.3731 | 16.3753 | 6 | NA |
| 3H-76 | EFPLVNPAR;FNEYDATLR;ILFEYAPVNVYYSTIESR;LVLGSQSPYNAYVLNYGSAENQLR;SLWSTDETLPR | 0.1757 | 19.5564 | 6 | NA |
| 3H-77 | MAFVHLFHIDGILECAER;QWATDELK;VHGWWWR | 0.3976 | 13.1941 | 6 | NA |
| 3H-22 | HASNDALNAIVYR;KVTVNTDYFVLFEDK;NHYDDTLYDTSVK | 0.2092 | 13.1941 | 5 | NA |
| 3H-59 | ALDELMDFKK;HVSAEIELK;KDNEISQLHEELDYATETINDLNDQLR;TAAQQLASYR;TELDHIK | 0.3663 | 17.3262 | 5 | putative nucleic |
| 3H-142 | ELSTLHR;EVPTTVSEDSKELSTLHR;FAVPSGK;KTPAISVTR | 0.1762 | 12.9001 | 4 | NA |
| 3H-163 | AGVFELINASDMPGAK;IDDLTLALK;ITLHEDLTR;KIDDLTLALK;LSVDEKDIVFK;QVAIIVNK | 0.1484 | 11.4227 | 4 | bro22 |
| 3H-181 | HIPSADDPGADLFTNWFSINAHDR;WYHLDCFLSK | 0.3063 | 7.5792 | 4 | NA |
| 3H-83 | ESFGTYMGQR;KPAQELIHEYR;MSPELQEAWR | 0.2039 | 13.1941 | 4 | glycosyltransferase |
| 3H-90 | DLHRLELPEETFIK;TGASTGYSSDSPSR;YDMLELLK | 0.1324 | 11.2030 | 4 | *iap*-like protein-5 |
| 3H-92 | DKIIEYIER;KLILVGLQR;LILVGLQR;NVIPITPVLFNR | 0.0457 | 11.7112 | 4 | NA |

Table S10 Identified HvAV-3h coded proteins from *Spodoptera exigua* produced virions (Se-3)

| Protein ID | Identified peptide sequence | Sequence coverage | Protein Q score | Unique spectra | Function annotation |
| --- | --- | --- | --- | --- | --- |
| 3H-57 | AEMQINTQR;AKLDLVTINSVANK;ALEMKEMWER;ALLEVTDNVVTDR;ALLEVTDNVVTDRVPVR;AQLDVADLER;AQLDVADLERR;AQLQSDLDVLR;ATIDALTANLNSSNSK;CQAELQQR;DEAIWAIK;DQLLKLWR;DSSFPIVDADNTEQLVVR;EHDVLER;EKAQLQSDLDVLR;EKQHAEFLR;ELACNVIPEKCGDITQMNR;EMYAVLEDELK;GGMGVLDDIYNR;HIAGVITK;HVGNAQSDGMYTENVALR;IESEAALVESLQR;IQAFVNTEQNILAAGTR;ISELLTQLQESNNQR;ITNEFFDHATLPLLR;IVENMYK;KQMGNVVQEIAQR;KTNDNLEIK;LDGSDVTLVVDDENLK;LDLVTINSVANK;LIDSVVK;LIDSVVKITR;LLISSETLGR;LLKEHDVLER;LLSLDGVR;LLSLDGVRR;LNDIAELQSQLTK;LNNELETSITEER;LQLDYMTLQQR;LTNECQAVSSR;MEFEAVNDALSK;MLELQNLMK;MTAAHLADDR;MTAAHLADDRLSNASVGVQLDR;NAEITNLQDR;NEIGQLR;NELAATVNSLQMQATK;NIDGAEGFTR;NIDGAEGFTRDEAIWAIK;NISAIRPR;NNVQLQTALQR;NSGVGNAINDR;NVNDTTLAELAQSR;QHAEFLR;QHPSFANKPNNTDVQQSDLTIAEDADTGTSVTK;QLNALNLR;QLNALNLRR;QMGNVVQEIAQR;QMKHENLYLASTTPSR;QQLEWILVNSNPAIALQDASVR;QSLLETSAQLNDVR;RLNNELETSITEER;RNNVQLQTALQR;RPIRPMADNFAEGYVYERPMGGGAPR;RPSVTDDEDDRIVGTR;RSLSYSLTDDEDDAAQIR;SDVVVLR;SETNALVVEVADLK;SETNALVVEVADLKSQLNR;SEYELSASR;SEYELSASRR;SLSYSLTDDEDDAAQIR;SNANLSPQSIR;SNANLSPQSIRNTDQQR;SQALYAIKR;SRSDVVVLR;SVTGPIR;SVTGPIRR;SYIIELESR;TKEMYAVLEDELK;TNDNLEIK;TQLLTGLR;TYQQEMTQLK;TYQQEMTQLKR;VAYANLEGDMQK;VITGDETVMTYNR;VNRNSGVGNAINDR;VSTDDLTSYSR;YDNYNRNEHELR;YEALMSEQSSK;YESLMTK;YQAAGTPGNGR | 0.6133 | 366.5109 | 180 | chromosome segregation SMC like protein |
| 3H-53 | ALFFGVR;DSGVALPIGMMVNNR;GQYMYSFALDLK;GSINPSNFNSSISVVIEPSEALR;GTIDSYIYGPDEQVTTYFVR;KIDNGGDFN;KLSDLLIK;LSDLLIK;MPVLLSTGNGSNK;NITHSEYLDYYEVGLPK;NITHSEYLDYYEVGLPKSNNGVR;RGTIDSYIYGPDEQVTTYFVR;SDVNKR;SIGVSALSK;SIGVSALSKIGIK;SVDIGAKTLVLPIPLYFSR;SYAAGTESSYFPK;TLVLPIPLYFSR;TSCSPHYILMEKPTDIAATEILKPTEAGSTLSYDIEHSSGILK;VPMLPAEHFVFTEPYR;VTVEFVFR;VTVEFVFRK;YRVPMLPAEHFVFTEPYR | 0.5495 | 99.6925 | 104 | major capsid protein |
| 3H-140 | ALSHPDWLTIGSFIK;DVDGVASNGGGSVGTGLGGPLGGTSGVNTSNLR;ELFYHSEAGR;ETNYPIPYFQYNLVLSQR;EVAKELFYHSEAGR;MLYGNSKTDVNATNK;NSDKDVTIPK;SGDEMTLSR;STGTTVGYTGTTSNAAMK;STGTTVGYTGTTSNAAMKDVDGVASNGGGSVGTGLGGPLGGTSGVNTSNLR;TATHLLGYK;TDVNATNKSTGTTVGYTGTTSNAAMK;TLDRPTDNAIMQR;YFFKENFVNLVELWCK;YTYMYVNK;YTYMYVNKR | 0.8443 | 72.6929 | 81 | NA |
| 3H-55 | AYELANER;AYELANERGPFSVEVYLNPGTSNTYQYVATTR;DSYRLPQSFTR;FNNTDYDDLPWNYTSGK;FNNTDYDDLPWNYTSGKK;GIASLWGYLK;GPFSVEVYLNPGTSNTYQYVATTR;IGQTPKAYELANER;KVVTATGVVSGGER;LPQSFTR;LPQSFTRDPLFR;NKFNNTDYDDLPWNYTSGK;SEIAQDIQVTMYNTNGGSNPLNNSNVTR;SNVDSSSYYQQPPQVVYNNGDLYGSR;TGYSGAELGASIDK;TGYSGAELGASIDKGIASLWGYLK;VVTATGVVSGGER;YVFLLR;YYYMSRPIGFGSSGAYDVPLLDTPLLR | 0.8800 | 78.4088 | 72 | NA |
| 3H-152 | ASYMNLLIVEQDR;DMVSPSNADGPR;DNTSYFDPLR;DVAVANSDK;EKVVQGATAVR;ELLNPEHSAK;FNLLHDTINSLFEAGVVDR;FSVTVESTR;FSVTVESTRR;FVEHCIIDAYTSNR;FYTEHLHTDELLNINVR;GTLMSELFTVDER;HDDTNTVIVQYSVDK;HDDTNTVIVQYSVDKHYLSR;HPPTADTALSAMSR;LIGMDGVQLETIR;LVYNASLLLR;QLSAHWR;RFNLLHDTINSLFEAGVVDR;RLVYNASLLLR;RVDDAELER;SEELDILK;SLSDAVQMVK;SLVIGAEQLER;STDTNSATFYPK;SVDLVPDGK;SVDLVPDGKEHITFNPGR;TSPTLILHVWR;TVSLFRPPLSLK;VDDAELER;VLLHLTAYTILK;VLLHLTAYTILKK;VLPLTAFGK;VLTDSVDTAR;VVQGATAVR;YETDFTSDESPFSIVTSIDK;YGEWLK;YITELLR | 0.2864 | 158.0269 | 52 | NA |
| 3H-56 | DMNGIAVSVDAK;EEDGWDVATTEK;IISETVK;IMNGAGAAGGAVVPK;LKEEDGWDVATTEK;LQPAPLDIK;LYPALMSPNTITNVIPDAR;MELHFEFM;SSVMVIGLSEVFMQQTSVTFR;TIIGDLGSLQGPEGGEWLAFFYPAHTVFEK;VGETPDLSSFSQEEISK;VGETPDLSSFSQEEISKLK | 0.6281 | 50.7576 | 50 | NA |
| 3H-151 | ALQYQTQLEGLLSR;ETCALTTVTASLATISESLGK;ETCALTTVTASLATISESLGKDEALLK;ITEVGGGVAAIK;ITEVGGGVAAIKSSLTSVVFTPLSEK;LDAIKSDISGK;LVQIDNTVK;LVQIDNTVKETCALTTVTASLATISESLGK;RVVVDPLDTK;SSLTSVVFTPLSEK;SSLTSVVFTPLSEKLDAIK;SSTTEERVAFGLTR;VAFGLTR;VEKALQYQTQLEGLLSR;VVVDPLDTK;VVVDPLDTKVEK;VVVVPVQDK;VVVVPVQDKITEVGGGVAAIK | 0.6059 | 76.2351 | 49 | Uyr/REP helicase |
| 3H-58 | AVSPLTNKPIDVHGR;DCNLSPPPTK;HIVVSNGFQPVR;KLIGTSPIR;LIGTSPIR;LTAAQCETFIR;LTAAQCETFIRNNK;LYNDLTK;MVYSMCHGVPYR;NDQNVPTYYTSSRPFADR;NESINPITGK;NESINPITGKK;RASPQR;SIRPEQAAYR;SPSPRPYTATSVTR;SQLINIANNMNIAELR;SVSPYSGRPIK;SVSPYSGRPIKR;TAANADTRPDLCATFSR;VAQNTTQSQLLNIVK;VAQNTTQSQLLNIVKFQIR;VLLSPIPDGATPMSR;YMINPNFVPTTTK;YMINPNFVPTTTKK | 0.3752 | 97.5405 | 49 | cofactor of the virion S/T kinase encoded by thioredoxin |
| 3H-13 | AAVWLATTTGDDSGYR;CVDEFR;DIGIDTGDPSSVQNR;DIISPLILLDAER;DPYDTLNK;DVIHEHVVAHWNDAETEQIIGR;ESPTEYFLR;EVMTVPSIVSALK;EWNATFVVIDEAHDLSVHGVDYPK;GANVAIILGSR;GLVLTPNR;HLNDFEIDR;KFAGLVSYLPSIVDVNEVDIVNVGER;KVLLLTATPMR;LSTHDITVEQFNR;LTTYAEFNETQLLYSDDDDRDR;NITNDNTIPLDAPR;QRPTFVYPFVLDNVSLYGLDAYR;QRPTFVYPFVLDNVSLYGLDAYRDTLAK;RPMPHQILVANYTTPR;SDRDVALLR;SFIAQLDR;SFLCLTR;SKLEELGAVITDVTCGVQAK;SPIDGIIVIHGVGTGK;TFFFNTITAFANTVSR;TSDVVLHK;TVDILMYATSEAK;VIAEGITLK;YALPLYPIAESTDESWSYEHLDK;YAWIAK;YQSHAYTIADWGSDAKPNVFVYR | 0.4682 | 127.0701 | 41 | DEAD-like helicase |
| 3H-27 | ADNVWVYTEDLQAIK;ALINEDGHSVVTQR;AMIESILK;CLLSITR;DPALYEVQLHTCR;FNIDNVNYASVAHAIALISVVYHMK;FSVLNSPLSR;FTDEIIK;FTDEIIKK;GVVWSEQSQQLVAAAATTK;HIHSVGDPSYNDEVTR;HITYVVVGHALVSHR;HSNVYSDALESFR;IVTSQLLYK;KVDEFISFGR;LYSTANELLADVLYGDGVDLGSTVSETTNR;NLCEIGVVSNVTDVDLLR;RDPALYEVQLHTCR;RPDAEECFK;RYAIDVNILPAISDTIQK;SLVLHFGANPAYTIMNTFASPAESPR;SLVVLTLQHELR;SLVVLTLQHELRK;SPPTLTIDDDSILYPLQYIPR;STEDDRPIQMQR;TGYANLK;VLTAALMHTDADTVNPTTPSVGYESDAVPR;YAIDVNILPAISDTIQK;YTDFDQLWSK;YVDMSQYR;YVPINR | 0.5152 | 128.2289 | 41 | NA |
| 3H-48 | ALLLSQLVSPPLIAK;AMHLLYIEGK;ATQNTVQTLR;CLGQVVTNVR;CSSTAAVDAAVASSSTEALSQK;GSSISTAVSK;LKNWSPK;LQSWVTDHEAVQYGTKPTVPYVIPDNLLSQKPTDNTISR;LYGYMANK;NDITAKNITSK;NEIIAKNDITAK;SVASVSSPGNIASSSVGNK;SVASVSSPGNIASSSVGNKR;TAQSIMQNLMTQIK;THIFNYLDSK;TVNVNQDSQTIK | 0.4198 | 65.0937 | 40 | NA |
| 3H-19 | AELEGVADNIK;ATVGALAGDVVK;ATVGALAGDVVKR;ESTTNIAEDIER;ESTTNIAEDIEREPAYLK;GDDVRPELSTFVLSYTR;ILITTYDYSGVQQR;ISPMYSFHSR;KNTNLYGVTR;LLYWLEDNRFDLLTVDGNDAR;MRPTTADVDNAQPTITVDDLK;MRPTTADVDNAQPTITVDDLKDVSK;NEQPQNSSSFDK;NTNLYGVTR;QIGNGEDANANVEINCGHR;QTLGLVIK;RNEQPQNSSSFDK;RTVLDAAR;SHVDDNEDLAPSK;SYADELGDR;TPSPTAVEVNTLAR;VFDVPPVIHGEIFYGPFDVTGLHSYAGPPSLSIIVSNHPSNGSQSK;VLPQHIR;WSYNSLTDGGAFAITGILAR;YRAELEGVADNIK | 0.4613 | 105.5206 | 34 | NA |
| 3H-110 | ATDINPYETK;FLTNEIK;FLTNEIKADGTAIR;GWVPVNGSDAFEFSCK;HLNPQWATDAPILK;IGFISQILPR;KATDINPYETK;KNVDDDTIELR;LGNVSGIITK;LSFDKK;LTFNGTAK;MAGLPLHAK;NVDDDTIELR;TMYAPLSK;TSNKPLIFECILDEPEK;TYKHLNPQWATDAPILK;VVLAPEK;VYEFISR | 0.7042 | 71.6975 | 33 | NA |
| 3H-66 | DIIMGHISPNR;DTFAVSPAR;IFLATNTNSHVR;ISTIPEFHTKPK;KVQVDYSPNYDLTVVTVNDHVSTTK;QAIVQNGR;SALVNCYAK;SIFSSPIPNLGFVAAENGHVR;SLAHEHEEEEVFNDNEK;SLAHEHEEEEVFNDNEKYDDER;SPASDQSYYGESNDGSYKR;TADGDYDDDER;TVISGDTYIK;VQVDYSPNYDLTVVTVNDHVSTTK;VTVHEVNADLYLCKPR;YTSNNDKTVISGDTYIK | 0.5189 | 66.3903 | 29 | NA |
| 3H-117 | CIPVVAYHAQDADAPTDTGLK;DDAIIIDDLPDVYITQPHR;DYAAVITAELILR;LDSAESEMAR;LEMFLSSDETR;MFAKPEPAIR;NLNALWDLWGADVIIER;NVNGFVPAVIFSDYEVYK;RLEMFLSSDETR;RPHLDEFLSYLFENFR;SRPTVFLDLDNTLICSLR;TAGEEVGGVK;VGVWTAASK | 0.9153 | 57.5978 | 25 | CDT phosphatase transcription factor |
| 3H-136 | AIDTFMYGYSPVTIEK;DRPIPNQDDWER;EFAVWNLCR;ELPLFPYTIGVMYR;FAYDANFR;GAAVAVSNINILSR;HNGLSDLSNILVR;ITITLLAIYNQYK;LAAEMFTDSLILK;LADVDFLVNYR;LAKPQSWFANGHILR;NSAVGSQQIR;QPCGLISTTTTSSSTSR;QSMTYDEQSTFK;RLADVDFLVNYR;TFEEFR;VFGSLAFNTTTVATILNYDR;WNLRPR;YSAATSEAYSMR | 0.2254 | 76.2736 | 25 | lipopolysaccharide modifying enzyme |
| 3H-62 | ATLEQVFVR;AYVDLK;AYVDLKDFVVTHENLFAR;DFVVTHENLFAR;KTQTPVNILADIVSLK;LDNNFVDGLK;LFVPGIDLPQPYIGIDTTK;LFVPGIDLPQPYIGIDTTKR;LITDPYQLLQISER;RLFVPGIDLPQPYIGIDTTK;TQTPVNILADIVSLK;VCINVNAMTAGDK;YTTSTVPNETDR;YTTSTVPNETDRR | 0.3870 | 59.6832 | 25 | myristylated membrane protein-like protein |
| 3H-115 | ADDCDISDIISR;DAILKR;DICTPILEK;HNNFTQDHEVTLGAPTASR;KQPSPVTTPSHSTDVR;KTPQLQEDLASYTK;LITATEEELK;LVYLEESDEEMRTEDNQS;QGNSSTMVAYGQHHQQQVAVR;TATATAGLFGATPLIGTIAGQVK;TPQLQEDLASYTK;TTTNPLASR;VAGIENLVLQTK;VCSIPTNEDNPDK;VCSIPTNEDNPDKTK;VMFDDEVPSK;YTANVTENESNSTIQSLDEFAK | 0.3600 | 72.4126 | 23 | NA |
| 3H-126 | ALNTHDAGAYK;APVTATPVPVKPLYTAR;DTDITTADNSGVVAK;ELPIWYDKFEEHVSEYLER;FVNDFFTSLFPDPEVK;GPSVVTVAK;HLEGFASLLLK;IRDPVATER;ISGSDVSQGLR;LAVTDEIAGSDDINVGQAK;NSAPQSGVITQIR;RVTYASIFYDAK;SAEPTTVSR;SVLISNK;TDIDSTVIADK;VFWALK;VTYASIFYDAK;WTLEVINWR | 0.1939 | 70.0565 | 23 | ATPase involved in DNA replication |
| 3H-23 | AVGHVVTIPMDR;FAITGSR;HKNGVPSNDDLSHRPPYK;IMTVLTSLR;INYAATVNNNAHR;LLHLVR;LVEADVNESTVHEYEHSIK;LVEADVNESTVHEYEHSIKR;RYVFNVDSVK;SPLNYSHENIER;VIEEIFK;VLPWSTTAAR;VPISYVHNYIMSR;VVVNSDETVYHAR;YDFMSEYLNVK;YGTFAVDSAR | 0.2209 | 61.8651 | 23 | NA |
| 3H-107 | ASLKTNTPLEGVPEFVLFHYGNAVK;FFPAETHVEVK;GILSIRR;IDHVPSAVVK;LQLSDWNDNVTTIK;LQLSDWNDNVTTIKK;NYVLNEPPAR;SPYANAAR;SYSAYR;TNTPLEGVPEFVLFHYGNAVK | 0.5644 | 39.2707 | 22 | thioredoxin-like protein |
| 3H-64 | AVIGSSGSVNNTVNTVHSR;ESGNNTNISPLNR;FLGLSGIFSMLADIGNTNK;GTPENPIVIR;IRSDVIAAKPNYK;LPDVGGVNDVISSK;NIILSGEHDR;QIVDEDDGR;QVYGDSLCLEK;SDVIAAKPNYK;SIESLLGIPSPSKPQSK;SKESGNNTNISPLNR;SQWADDYSGR;SVTSDVAALAHSETSR;TNPPGTVGR | 0.4028 | 64.1463 | 22 | NA |
| 3H-71 | KYCADIFDIVQHAASL;LGKPIVVIAPVR;LGKPIVVIAPVRR;LSESVAVIEAK;LSESVAVIEAKR;NEVAWLLR;NLFTNIR;RNLFTNIR;SDSLDACLTFIDIVLDSHR;SRNEVAWLLR;VQFPHVVHNPELNVIYWR;YCADIFDIVQHAASL | 0.4567 | 48.0059 | 21 | sulfhydry1 oxidase Erv1 like protein |
| 3H-69 | AAILLGASLVLNK;AAILLGASLVLNKVMVS;DESHYMLYTK;DYDTGKDESHYMLYTK;LVYKDYDTGK | 0.4744 | 21.2513 | 19 | NA |
| 3H-109 | ALIVKPMNQFR;IDYKETQR;LTFFKK;NSWGEHWGDGGYFK;QAVSSSGCGGGSTYR;SQLHFETHESK;SVIPTNATTNAYNAVK;VALIMSIDAVIR;VGGAYFVTVR;YLDSLIPTPR | 0.2749 | 41.8820 | 17 | cathepsin B |
| 3H-116 | CLYIDYQGGK;EHYTLLNTPSATK;ETFYQVFSQVTSVDHR;FGCLEYR;IAFVGKPGTGK;IECSPLPEPGR;KIEAQCAYDQINT;LWEFADDFPNK;LWEFADDFPNKETFYQVFSQVTSVDHR;MIDDSLIIPQFHHNLISPR;TCLDGIFLFGTGNHDVR;VTTPVVGSSPSESASSPIR;YIMYTK | 0.4298 | 53.6569 | 17 | ATPase involved in DNA metabolism |
| 3H-150 | AKLELDCYNEDLR;CLVDLGR;FHNCPNDLTTQIAR;LAVEYNGR;LQVIFDDNDEK;LQVIFDDNDEKR;NIDLIDVPYTIPIDEIPNFIDK;QGVNTLAEYGK;QHYEYVPR;SADDALLDPTTSPSPVFAR;VRPDWLINPNTK | 0.5408 | 47.3553 | 17 | NA |
| 3H-182 | APQQIATVK;CDDGELTLHR;DVEFVAR;HTGGLIITAPLVHPAVGTTR;RHTGGLIITAPLVHPAVGTTR;SITDMIAAGAPISK;SITLQNTVTK;SLPDPLDIYVR;TQHHNEEVETVDQSILAHVR;VFECPNTEEVPNFR | 0.2937 | 43.8534 | 17 | NA |
| 3H-1 | DLNELHSR;ELAAHITSK;IEFLNIR;KTILQSIDIAQR;LPFAGWIETGR;LSSVDLQR;LSVSLVDR;QIINPLTK;QLNYSGAYVK;SIFEDLAK;TFAWDIEAK;TILQSIDIAQR;TSNPDDLPIEEISYFK;VPQHSHHDILYNTK;VTANSVYGSTGAVNGK | 0.1326 | 61.6147 | 16 | DNA polymerase |
| 3H-128 | DVISDQPR;ELAIGIDER;HICNDIVNVFNEK;IATNTDLK;KQTSSSQQRPNDLLYPLSYGAVDETVVLK;LIAAVGGIVIALGVLLKR;QTSSSQQRPNDLLYPLSYGAVDETVVLK;VVGVTYK;YADIPVWEQGVEDEPTSETNDTLNR;YADIPVWEQGVEDEPTSETNDTLNRDVISDQPR | 0.4366 | 39.5137 | 16 | NA |
| 3H-43 | AVVFSDSR;DLYNLQILR;DQSLELLSHTHR;FVEFGTLVRPAGWGSLSELGK;KDFNVASYESDPLLK;LSLGYQTVVQFYR;LSYDSVVDWER;LTIDER;MFNVDQAIEFFENR;SSPQTVGLEWWK;VCHLNAVLDPAFTIDTGNVR;YNSSMDEYRDQSLELLSHTHR | 0.6438 | 47.2136 | 16 | NA |
| 3H-46 | HSDDIDTTIDALVK;ISKQLTQLR;KLDQEVLIQEMR;QLTQLR;QLTQLREK;YMEVSSNDTLLYDNVR | 0.2062 | 23.3907 | 16 | NA |
| 3H-135 | ALNMILK;DQVPVLTK;KRPIQPTAYNLFYK;RPIQPTAYNLFYKDQVPVLTK;SNSNNTMTALDGVHQINSLVTSVFSDSEHINEWLSLSK;YLLDNYNYR | 0.6080 | 25.7946 | 15 | yabby-like transcription factor |
| 3H-37 | AGLLELVIK;EFFTLNEDNFTELIK;IDALDDFEVWR;IGYTCNLNDR;ILEYSNAPNAIAK;LHSQVTNLHPLSK;LSELNVASAYDFRPVFVVPTK;LSELNVASAYDFRPVFVVPTKK;QLEAVLHTK;QTLEEQLPR;REFFTLNEDNFTELIK;YWLVNELFPSLK | 0.4306 | 49.9764 | 15 | bro4 |
| 3H-63 | DNKYLWTFAPLLEQR;EYVFGVR;EYVFGVRK;FLFLDEANKPLHEIAVQFTCK;FLILSASGPLK;HYTGIIPETMNVAK;NVGLFIPK;NVGLFIPKK;SGDEIDGYIIMKPK;TAFSIVNK | 0.3322 | 36.6445 | 15 | NA |
| 3H-125 | DDLYNLGIRPDR;ENTTPRPVAIDVR;HSTLLTELAILEER;KLEDLYR;LELTTSLIHDTVTVLK;LVLENFR;QLPDVPRPSR;QNILSNIDSTTK;TTSVTLEHAGIR | 0.1164 | 34.5312 | 14 | ATPase involved in DNA repair |
| 3H-84 | LLSPPVDPHVFIDTTAASIDK;NMVINEPQYQQFLR;NSEEDSSPAQADQSESVGSSPPTK;SPHLDMAFR;TGTEPKPEHSDDGKNSEEDSSPAQADQSESVGSSPPTK;VVAKPQYDDR | 0.3983 | 27.2598 | 14 | ABC-type transport system permease |
| 3H-24 | EGVLEITNSK;EIYGDR;IAEIVVK;IDYDYLVSPQLK;KNSVDRPAFK;LAQECIEVLTNR;QFVYGVIGDYAGIINPTYENLNSAR;SANTTSVNAVNYR;THGFEGSGGSNDSLVNVSR;VNADSVYTDETAANVHR;YGNVDSQYYK | 0.1937 | 45.9731 | 13 | RNaseIII |
| 3H-28 | GIDDGDAIYTITK;IFMLVLLER;ITALYHNVK;LFFDLR;LTEWFPNNR;NITLSPQYCSEYLTLK;NNLNYDQLYTALR;NVAEYQSR;TAYDQADVIAYIDGDKEK | 0.2853 | 34.1539 | 13 | NA |
| 3H-137 | AMSNGLGDVILSAK;DTMAMIFK;IFDAFK;IFDAFKR;KIFDAFK;NALLTLK;SEDEEVSAEIVTAK;TGVDLSVNVGQTYK;TVRSEDEEVSAEIVTAK;VSTTVDPMMPAQVK;YSVAVAK | 0.5563 | 43.4915 | 12 | hemolysin-like protein |
| 3H-67 | ALDREGNENALR;FLEALNVHTPQWK;GVSLRPDIVSVSGDVWNDDYPQLVSGHIHTR;IFEELFKK;ITISDLNLR;LIVAHQELR;LLFVGDPHFK;LLFVGDPHFKR;SILQAGGPLLR;SLPTGVGFAFK | 0.2882 | 41.2965 | 12 | DNA repair exonuclease |
| 3H-118 | LNGQLHTLVIPK;RYAYTDSDGVYVK;SIVAAFATADLPTALYHSETATYR;YAYTDSDGVYVK | 0.2917 | 18.1732 | 11 | NA |
| 3H-3 | DYFGWDK;DYFGWDKLEAAYNAK;IIQIAPVDLIK;KDYFGWDK;TLLKPTVHLNR;YANVNGFEGEFNSQYSVQSYNK | 0.2941 | 25.2291 | 11 | NA |
| 3H-44 | DNSEASSQNTTVWDR;DNSEASSQNTTVWDRR;TANTSTNTSAAATSGKDNSEASSQNTTVWDR | 0.3168 | 13.6299 | 11 | NA |
| 3H-61 | ADVVNATLGTK;AIEDNVR;FVRPLLYNR;LEPIVK;NLDYEFSVR;QFLIDVMNK;SHGVLLNGTPIGYVSDVQR;TLTVTELVLPYSAK;TSIATAIDSVR;VEETIHHATNHTIGDK | 0.0961 | 37.0721 | 11 | RNA polymerase subunit |
| 3H-9 | AFSVTATDFLHVGPAIK;GDYFNVGQTK;IPEFLEK;IPHPLNSNVFIRPK;ISDLLTLLEK;LNTILATAK;SVIGPDPTLK;VLYDVPDEDAKR;VTEHNYEWASSIR | 0.1187 | 36.7998 | 11 | RNA polymerase subunit |
| 3H-124 | GYLLAAIK;IDINRDDDDYLVTDINPFEVNTENAR;INDPVLVIDSAR;LSRPPGIDGDPNK;RGYLLAAIK;TLLEYNSLYR;TTGYQQLYAELK;VGENSAWHNFETR;VVDILR | 0.1540 | 33.0503 | 10 | serine/threonine protein kinase |
| 3H-139 | AHIAPALLNHIESNLLK;ATLDEVSNDPDDIDVERR;FVFSARPTVDSSLASGGVR;QTTVDKWIDSR;VAAYEIAR;VCAAVAR | 0.2888 | 23.6199 | 10 | putative S1/P1 nuclease |
| 3H-80 | ATIQLLSMILTR;DNGWVEHEATK;IDEDTVAAAITSALSVR;KIEDIINLPK;LLDFIRPR;LSLYGLYR;QNVPSIVEEQR;SLAFTHNVYDVFR;YDGIEVNVPR;YRIDEDTVAAAITSALSVR | 0.2272 | 42.0341 | 10 | RNA polymerase subunit |
| 3H-82 | DAWTSNTEEASLR;GGHGSVGEVHTSAVVR;LGYMAAGYESTYESVVR;RTVSAVGSNEELANTDDSSR;TVSAVGSNEELANTDDSSR;YDGEALTR | 0.2263 | 25.6802 | 10 | NA |
| 3H-171 | DFLTLFDVDELRDKPK;IASHQLACLNYTLPVR;ILIVINQR;LGFQEFVYR;LMHYGDAPNDVTPSK;NMTTADLQDIVTLLTK;VIDTFSK | 0.2771 | 29.0208 | 9 | caspase-like protein |
| 3H-21 | FKEVEDLVLR;IASLLVHDIHSSLDDAVK;IGSIAPIELNPPPR;NVETFVR;YNNDSIR | 0.2456 | 18.3970 | 9 | NA |
| 3H-7 | ALYELASELAVEDQEK;DTVELFK;FFADTYATYR;FQTQVTQLFR;LSGVDDIR;NLVYTLR;SIIVLGADTLNLTNK | 0.1776 | 27.9859 | 9 | NA |
| 3H-77 | MAFVHLFHIDGILECAER;QWATDELK;VAALKQWATDELK;VHGWWWR | 0.4578 | 17.1318 | 9 | NA |
| 3H-113 | AAGFIVIEQDINTDNVTLVHEALR;AFMELIR;ALVVDPPQSVVR;FTSVLPPR;MHAIIPSMVTALTALVSVR;RLDIAFSGTDK;SMLIDDLNSGFDVVIPPDRR;TTLGGSTDSVMR | 0.3275 | 30.3125 | 8 | lysophospholipid acetyltransferase |
| 3H-16 | ASTFGVGFK;LGNVLFWNDLLK;LVAGGLIVIR;NQGVNDVLHAIDWTIENAR;QLADGLGFILSK;YLSFVEPK | 0.1069 | 23.3074 | 8 | lipase like protein |
| 3H-79 | FPFWFER;NEPVSYLAVGNAQFIDR;RNEPVSYLAVGNAQFIDR;SGLNYIITFDSSATEK;SPNCALVFTR;VPVSNTVEFVIVILK | 0.5280 | 27.2598 | 8 | NA |
| 3H-129 | LREYFENHGVR;LVALTTSVR;NVVDVQR;SAYDGIITTFK;SFLLHR;VVVTATQAVAAQLVSGK;YSISSDFVK | 0.1378 | 26.2230 | 7 | helicase |
| 3H-156 | DLFNFVSQTR;DTGHLVAFINGGTR;FYDSSTSTCPINYR;IVQFPPGSPYTFVATR;QTVLAQSNSGAYPVER;RQTVLAQSNSGAYPVER | 0.1849 | 26.2184 | 7 | NA |
| 3H-4 | LSHDYAYSLR;NSDFAWK;NVTFYVTIK;SSTVVSTVSSATWVK;VYTNVSDIPSAEK | 0.2288 | 19.3687 | 7 | NA |
| 3H-142 | ELSTLHR;EVPTTVSEDSK;EVPTTVSEDSKELSTLHR;FAVPSGK;KTPAISVTR | 0.1762 | 20.9851 | 6 | NA |
| 3H-163 | ATSNSTSAGNNITNK;ITLHEDLTR;ITLHEDLTREDLLSAIESTVSSR;KIDDLTLALK;LSVDEKDIVFK;QVAIIVNK | 0.1841 | 18.1732 | 6 | bro22 |
| 3H-26 | ANSIIDNIER;QIVDPNATMK;SGATYAINNSYTIDALR;TSEIVDVVDATR;TSEIVDVVDATRK | 0.3731 | 21.2513 | 6 | NA |
| 3H-157 | DLPDTVPVSLDVR;GINDLSDAELAYK;NPPLELWSR;VLIDTEQKR | 0.2543 | 16.7080 | 5 | tyrosyl-DNA phosphodiesterase |
| 3H-59 | ALDELMDFK;ALDELMDFKK;HVSAEIELK;KDNEISQLHEELDYATETINDLNDQLR;TAAQQLASYR | 0.3256 | 19.5199 | 5 | putative nucleic |
| 3H-8 | DGYVQGIAAVYYAPHTSAATDQAFK;LNAQDVAVLGTSNPILR;VYIPVPPITR | 0.1195 | 12.1647 | 5 | NA |
| 3H-90 | DLHRLELPEETFIK;LELPEETFIK;TAEAWYK;TGASTGYSSDSPSR;YDMLELLK | 0.1581 | 20.4447 | 5 | *iap*-like protein-5 |
| 3H-133 | DTTFSQFYER;FIEEFGDFLEELGHSR;TLVISGGGFK | 0.1169 | 11.8365 | 4 | patatin-like phospholipase |
| 3H-134 | IITHEEYVNLEADFESYKK;ISNILSPK;VATYPPPTSNDESASHER | 0.2055 | 11.4922 | 4 | NA |
| 3H-181 | HIPSADDPGADLFTNWFSINAHDR;WYHLDCFLSK | 0.3063 | 7.0559 | 4 | NA |
| 3H-76 | FNEYDATLR;ILFEYAPVNVYYSTIESR;LVLGSQSPYNAYVLNYGSAENQLR;SLWSTDETLPR | 0.1535 | 18.1732 | 4 | NA |
| 3H-78 | AIYVAEGMAPER;FWYGFLSNK;GLSTFIAK;LEQYIDKK | 0.1121 | 12.5685 | 4 | nuclease |
| 3H-92 | DKIIEYIER;KLILVGLQR;LILVGLQR;NVIPITPVLFNR | 0.0457 | 13.4663 | 4 | NA |
| 3H-93 | SAYEWYESR;SYVVASYVK;YFYDYFVQK | 0.0865 | 11.8985 | 4 | gamma_glutamyl hydrolase-like protein |

Table S11 Identified HvAV-3h coded proteins from *Spodoptera frugiperda* produced virions (Sf-1)

| Protein ID | Identified peptide sequence | Sequence coverage | Protein Q score | Unique spectra | Function annotation |
| --- | --- | --- | --- | --- | --- |
| 3H-57 | AEMQINTQR;AKLDLVTINSVANK;ALLEVTDNVVTDR;ALLEVTDNVVTDRVPVR;AQLDVADLER;AQLDVADLERR;AQLQSDLDVLR;ATIDALTANLNSSNSK;CGDITQMNR;CQAELQQR;DEAIWAIK;DQLLKLWR;DSSFPIVDADNTEQLVVR;DVNLAKSETNALVVEVADLK;EHDVLER;EKAQLQSDLDVLR;FNRQQLEWILVNSNPAIALQDASVR;GELNAMTR;GGMGVLDDIYNR;GRISELLTQLQESNNQR;HENLYLASTTPSRNDEMAK;HIAGVITK;HIAGVITKNIDGAEGFTR;HVGNAQSDGMYTENVALR;IESEAALVESLQR;IQAFVNTEQNILAAGTR;ISELLTQLQESNNQR;ISNSVKNDVESK;ITNEFFDHATLPLLR;KQMGNVVQEIAQR;LDGSDVTLVVDDENLK;LDLVTINSVANK;LDLVTINSVANKVNR;LIDSVVK;LIDSVVKITR;LLISSETLGR;LLKEHDVLER;LLSLDGVR;LLSLDGVRR;LLVSEDK;LNDIAELQSQLTK;LNKELNTVR;LNNELETSITEER;LQLDYMTLQQR;LSNASVGVQLDR;LTNECQAVSSR;MEFEAVNDALSK;MLELQNLMK;MTAAHLADDR;MTAAHLADDRLSNASVGVQLDR;NAEITNLQDR;NELAATVNSLQMQATK;NIDGAEGFTRDEAIWAIK;NISAIRPR;NNVQLQTALQR;NSGVGNAINDR;NVNDTTLAELAQSR;QHAEFLR;QHPSFANKPNNTDVQQSDLTIAEDADTGTSVTK;QLNALNLR;QMGNVVQEIAQR;QQLEWILVNSNPAIALQDASVR;QSLLETSAQLNDVR;RLLVSEDK;RLNNELETSITEER;RNNVQLQTALQR;RSLSYSLTDDEDDAAQIR;SDVVVLR;SETNALVVEVADLK;SETNALVVEVADLKSQLNR;SEYELSASRR;SLSYSLTDDEDDAAQIR;SNANLSPQSIR;SQALYAIK;SRSDVVVLR;TKEMYAVLEDELK;TNDNLEIK;TYQQEMTQLK;TYQQEMTQLKR;VAYANLEGDMQK;VITGDETVMTYNR;YEALMSEQSSK | 0.5413 | 350.8651 | 217 | chromosome segregation SMC like protein |
| 3H-53 | ALFFGVR;DSGVALPIGMMVNNR;EIRPCAAFSK;ENVPDTGAGTGFMNMLTDGDFATPPEITK;FEVVADFAVVTDFEIDR;GQYMYSFALDLK;GQYMYSFALDLKTVDPK;GSINPSNFNSSISVVIEPSEALR;GTIDSYIYGPDEQVTTYFVR;KIDNGGDFN;KLSDLLIK;LSDLLIK;MPVLLSTGNGSNK;NITHSEYLDYYEVGLPK;NRGQYMYSFALDLK;RGTIDSYIYGPDEQVTTYFVR;SIGVSALSK;SIGVSALSKIGIK;SVDIGAKTLVLPIPLYFSR;SYAAGTESSYFPK;SYAAGTESSYFPKLAHK;TLVLPIPLYFSR;TSCSPHYILMEKPTDIAATEILKPTEAGSTLSYDIEHSSGILK;VPMLPAEHFVFTEPYR;VTVEFVFR;VTVEFVFRK;YRVPMLPAEHFVFTEPYR | 0.6725 | 117.5065 | 111 | major capsid protein |
| 3H-152 | ASYMNLLIVEQDR;CPGNLEAMLAVQR;CTYTWYLSK;DMVSPSNADGPR;DNTSYFDPLR;EHITFNPGR;EKVVQGATAVR;ERNHIALFMVR;FIYEYNNYTVR;FNLLHDTINSLFEAGVVDR;FSVTVESTR;FVEHCIIDAYTSNR;FYTEHLHTDELLNINVR;GAVQVGPNAAIEAVMR;GIYSPGMTYDIAHVYPAPGVGYTISVLSNNAPTVDVPR;HDDTNTVIVQYSVDKHYLSR;HPPTADTALSAMSR;IYDAPNTNVLYGIANDITSHMIQYK;KGTLMSELFTVDER;LIGMDGVQLETIR;LVYNASLLLR;MSAYETSEIR;MSAYETSEIRK;MVWFPEFVSPVSDDAPR;RELLNPEHSAK;RFNLLHDTINSLFEAGVVDR;RLVYNASLLLR;RVDDAELER;SGVHSAVDMYMYIR;SIINVNNYAR;SLVIGAEQLER;SQTFYNTQR;STDTNSATFYPK;SWVDRDPESNTK;TSPTLILHVWR;TVSLFRPPLSLK;TYQALLPQPMR;VDDAELER;VLLHLTAYTILK;VLPLTAFGK;VLTDSVDTAR;VLTDSVDTARR;YETDFTSDESPFSIVTSIDK;YGEWLK;YITELLR | 0.4169 | 188.2303 | 64 | NA |
| 3H-27 | ALINEDGHSVVTQR;ARDDNSPSNDSVAMK;CLLSITR;DDNSPSNDSVAMK;DDNSPSNDSVAMKK;DPALYEVQLHTCR;DRSLVVLTLQHELR;DSANSLLK;FNIDNVNYASVAHAIALISVVYHMK;FTDEIIK;FTDEIIKK;GVVWSEQSQQLVAAAATTK;HIHSVGDPSYNDEVTR;HITYVVVGHALVSHR;HSNVYSDALESFR;HSNVYSDALESFRR;IVTSQLLYK;KADNVWVYTEDLQAIK;KVDEFISFGR;LYSTANELLADVLYGDGVDLGSTVSETTNR;NAFTSIIWNVDIFK;NLCEIGVVSNVTDVDLLR;RDPALYEVQLHTCR;RYAIDVNILPAISDTIQK;RYDMDYYNGNMYSDLYATGNSFTLK;SLVLHFGANPAYTIMNTFASPAESPR;SLVVLTLQHELR;SLVVLTLQHELRK;SPPTLTIDDDSILYPLQYIPR;STEDDRPIQMQR;TDYELR;TGYANLK;VLTAALMHTDADTVNPTTPSVGYESDAVPR;VNPWMFAELAMLMR;VSDPQTTVR;YAIDVNILPAISDTIQK;YTDFDQLWSK;YVDMSQYR | 0.5968 | 155.6577 | 64 | NA |
| 3H-50 | ASCGTIPDAR;ATHYDPIR;ATHYDPIRDGYYMYALPNGR;DGYYMYALPNGR;DGYYMYALPNGRVDR;DYFLSSK;DYFLSSKYK;FLKPTFWFSR;LPSDSGVAVTTSPTVVDDLDLR;NALVALSNSSMNNEQNYTNR;NVYKDPAR;YDNFQCNK;YNNLSWLLSK;YNNLSWLLSKNVYK | 0.6349 | 58.1824 | 58 | NA |
| 3H-13 | AAVWLATTTGDDSGYR;AVMNSFR;CVDEFR;DIISPLILLDAER;DPYDTLNK;DVIHEHVVAHWNDAETEQIIGR;EVMTVPSIVSALK;EWNATFVVIDEAHDLSVHGVDYPK;FAGLVSYLPSIVDVNEVDIVNVGER;FVFPDGTYGAEGYATWMDSK;GANVAIILGSR;HLNDFEIDR;KVLLLTATPMR;LSTHDITVEQFNR;LTTYAEFNETQLLYSDDDDRDR;NITNDNTIPLDAPR;NNLSDLVPLHNLLMK;NTVIVVHDMSDVMSATYHYAGAGER;QRPTFVYPFVLDNVSLYGLDAYR;QRPTFVYPFVLDNVSLYGLDAYRDTLAK;RPMPHQILVANYTTPR;SFIAQLDR;SKLEELGAVITDVTCGVQAK;SPIDGIIVIHGVGTGK;SSIEAQLNNDMLAK;SVINAAER;TFFFNTITAFANTVSR;TLTAILAMINNITAGHDQGMK;TVDILMYATSEAK;YALPLYPIAESTDESWSYEHLDK;YAWIAK;YQSHAYTIADWGSDAKPNVFVYR | 0.5148 | 135.5450 | 53 | DEAD-like helicase |
| 3H-56 | DIHKFNGSK;DMNGIAVSVDAK;EEDGWDVATTEK;IMNGAGAAGGAVVPK;LKEEDGWDVATTEK;LQPAPLDIK;LQPAPLDIKGGRPMVR;LTNDEMNR;LYPALMSPNTITNVIPDAR;LYPALMSPNTITNVIPDARNGVHSTDR;MELHFEFM;QTLARDMNGIAVSVDAK;SSVMVIGLSEVFMQQTSVTFR;TIIGDLGSLQGPEGGEWLAFFYPAHTVFEK;TIIGDLGSLQGPEGGEWLAFFYPAHTVFEKMR;VGETPDLSSFSQEEISK | 0.7603 | 68.2896 | 52 | NA |
| 3H-55 | AYELANER;AYELANERGPFSVEVYLNPGTSNTYQYVATTR;DSYRLPQSFTR;FNNTDYDDLPWNYTSGK;FNNTDYDDLPWNYTSGKK;GIASLWGYLK;GPFSVEVYLNPGTSNTYQYVATTR;IGQTPKAYELANER;KVVTATGVVSGGER;LPQSFTR;NKFNNTDYDDLPWNYTSGK;SEIAQDIQVTMYNTNGGSNPLNNSNVTR;SNVDSSSYYQQPPQVVYNNGDLYGSR;TGYSGAELGASIDK;TGYSGAELGASIDKGIASLWGYLK;VVTATGVVSGGER;YYYMSRPIGFGSSGAYDVPLLDTPLLR | 0.8311 | 75.6786 | 47 | NA |
| 3H-58 | AVSPLTNKPIDVHGR;DCNLSPPPTK;HIVVSNGFQPVR;KLIGTSPIR;LIGTSPIR;LIGTSPIRK;LTAAQCETFIRNNK;LYNDLTK;MCAGTPNTR;MVYSMCHGVPYR;NDQNVPTYYTSSRPFADR;NESINPITGKK;QSMTAMR;SIRPEQAAYR;SQLINIANNMNIAELR;SVSPYSGRPIK;TAANADTRPDLCATFSR;VAQNTTQSQLLNIVK;VAQNTTQSQLLNIVKFQIR;VLLSPIPDGATPMSR;YMINPNFVPTTTK;YMINPNFVPTTTKK | 0.3687 | 86.8817 | 43 | cofactor of the virion S/T kinase encoded by thioredoxin |
| 3H-140 | ALSHPDWLTIGSFIK;DVDGVASNGGGSVGTGLGGPLGGTSGVNTSNLR;ELFYHSEAGR;ETNYPIPYFQYNLVLSQR;MLYGNSK;SGDEMTLSR;STGTTVGYTGTTSNAAMK;TATHLLGYK;TATHLLGYKNK;TLDRPTDNAIMQR;YTYMYVNK;YTYMYVNKR | 0.6745 | 53.1402 | 39 | NA |
| 3H-48 | ALLLSQLVSPPLIAK;AMHLLYIEGK;ATQNTVQTLR;CLGQVVTNVR;CSSTAAVDAAVASSSTEALSQK;FSINVYTIHDPTKPVAAVSLVGGEAFQSDDIGVTFYNPENYYPPADR;GKLYGYMANK;LQSWVTDHEAVQYGTKPTVPYVIPDNLLSQKPTDNTISR;LSDDRFSINVYTIHDPTKPVAAVSLVGGEAFQSDDIGVTFYNPENYYPPADR;NEIIAKNDITAK;SVASVSSPGNIASSSVGNK;SVASVSSPGNIASSSVGNKR;TAQSIMQNLMTQIK;THIFNYLDSK;TVNVNQDSQTIK | 0.4856 | 69.7958 | 39 | NA |
| 3H-151 | ALQYQTQLEGLLSR;CKPGEVMDDK;ETCALTTVTASLATISESLGK;ETCALTTVTASLATISESLGKDEALLK;ITEVGGGVAAIKSSLTSVVFTPLSEK;LDAIKSDISGK;LVQIDNTVK;RVVVDPLDTK;SSLTSVVFTPLSEK;SSLTSVVFTPLSEKLDAIK;SSTTEER;VAFGLTR;VEKALQYQTQLEGLLSR;VVVDPLDTK;VVVVPVQDK;VVVVPVQDKITEVGGGVAAIK | 0.6552 | 67.0249 | 33 | Uyr/REP helicase |
| 3H-19 | ATTLANDR;ATVGALAGDVVKR;DIYIENK;ESTTNIAEDIER;ESTTNIAEDIEREPAYLK;GDDVRPELSTFVLSYTR;IFSMLDNEIHLTIDSSDHQHVLQIYNR;ILITTYDYSGVQQR;ISPMYSFHSR;KNTNLYGVTR;LLYWLEDNRFDLLTVDGNDAR;MRPTTADVDNAQPTITVDDLKDVSK;QIGNGEDANANVEINCGHR;QTLGLVIK;SETTDASNVSPR;SHVDDNEDLAPSK;SHVDDNEDLAPSKR;STVTIEIVDDNHFEHNVECVLDTK;SYADELGDR;TPSPTAVEVNTLAR;VFDVPPVIHGEIFYGPFDVTGLHSYAGPPSLSIIVSNHPSNGSQSK;WSYNSLTDGGAFAITGILAR;YMSNYVPHDDGNVELDDLQLIQPLEEVSR | 0.5650 | 96.9103 | 33 | NA |
| 3H-110 | ATDINPYETK;FLTNEIK;FLTNEIKADGTAIR;GWVPVNGSDAFEFSCK;HLNPQWATDAPILK;IGFISQILPR;KATDINPYETK;LGNVSGIITK;LTFNGTAK;MAGLPLHAK;NVDDDTIELR;TMYAPLSK;TSNKPLIFECILDEPEK;TYKHLNPQWATDAPILK;TYVTLNR;VVLAPEK;VYEFISR | 0.7089 | 63.7795 | 26 | NA |
| 3H-117 | CIPVVAYHAQDADAPTDTGLK;DDAIIIDDLPDVYITQPHR;DYAAVITAELILR;LDSAESEMAR;LEMFLSSDETR;NLNALWDLWGADVIIER;NVNGFVPAVIFSDYEVYK;RLEMFLSSDETR;RPHLDEFLSYLFENFR;SRPTVFLDLDNTLICSLR;VGVWTAASK | 0.8095 | 52.2483 | 24 | CDT phosphatase transcription factor |
| 3H-43 | AVVFSDSR;DQSLELLSHTHR;FGLASVNDENSYK;FVEFGTLVRPAGWGSLSELGK;IGTSAFTDDLHTVVDQGSGECMDTYIAR;KDFNVASYESDPLLK;LSLGYQTVVQFYR;LSYDSVVDWER;LTIDER;MFNVDQAIEFFENR;SSPQTVGLEWWK;VCHLNAVLDPAFTIDTGNVR;YNSSMDEYR;YNSSMDEYRDQSLELLSHTHR | 0.7811 | 58.8380 | 21 | NA |
| 3H-107 | ASLKTNTPLEGVPEFVLFHYGNAVK;FFPAETHVEVK;IDHVPSAVVK;LQLSDWNDNVTTIK;LQLSDWNDNVTTIKK;NYVLNEPPAR;RIDHVPSAVVK;SPYANAAR;TNTPLEGVPEFVLFHYGNAVK;YASDSLMYFECDLSIR | 0.5890 | 44.7413 | 20 | thioredoxin-like protein |
| 3H-62 | ATLEQVFVR;AYVDLKDFVVTHENLFAR;DFVVTHENLFAR;INTGSITDSVQCFNK;KTQTPVNILADIVSLK;LDNNFVDGLK;LFVPGIDLPQPYIGIDTTKR;LITDPYQLLQISER;RLFVPGIDLPQPYIGIDTTK;TQTPVNILADIVSLK | 0.3527 | 47.4984 | 20 | myristylated membrane protein-like protein |
| 3H-69 | AAILLGASLVLNK;DESHYMLYTK;DYDTGKDESHYMLYTK | 0.3718 | 14.2495 | 19 | NA |
| 3H-71 | KYCADIFDIVQHAASL;LGKPIVVIAPVR;LGKPIVVIAPVRR;LSESVAVIEAK;LSESVAVIEAKR;MMVDVK;NEVAWLLR;NLFTNIR;NVHLVLPCASCAHEAYTYSSSHR;RNLFTNIR;SDSLDACLTFIDIVLDSHR;SRNEVAWLLR;VQFPHVVHNPELNVIYWR | 0.5962 | 53.3167 | 19 | sulfhydry1 oxidase Erv1 like protein |
| 3H-109 | ALIVKPMNQFR;IAQYPINTK;IDYKETQR;NSWGEHWGDGGYFK;QAVSSSGCGGGSTYR;SISDDLIR;SQLHFETHESK;SVIPTNATTNAYNAVK;VALIMSIDAVIR;VGGAYFVTVR;YLDSLIPTPR | 0.3017 | 47.2873 | 16 | cathepsin B |
| 3H-126 | ALNTHDAGAYK;APVTATPVPVKPLYTAR;ELAGVVGVVCNAVR;ELPIWYDKFEEHVSEYLER;EYFLLSVAQIFR;ISGSDVSQGLR;LAVTDEIAGSDDINVGQAK;NDEALIR;NSAPQSGVITQIR;RVTYASIFYDAK;STAAAITETVAGILK;TDIDSTVIADK;VTYASIFYDAK;WTLEVINWR | 0.1616 | 61.2793 | 15 | ATPase involved in DNA replication |
| 3H-37 | AGLLELVIK;EFFTLNEDNFTELIK;IDALDDFEVWR;ILEYSNAPNAIAK;LHSQVTNLHPLSK;LSELNVASAYDFRPVFVVPTK;QLEAVLHTK;QTLEEQLPR;REFFTLNEDNFTELIK;YAAEFR;YWLVNELFPSLK | 0.4132 | 40.7423 | 14 | bro4 |
| 3H-66 | DIIMGHISPNR;DTFAVSPAR;IFLATNTNSHVR;KVQVDYSPNYDLTVVTVNDHVSTTK;TADGDYDDDER;VQVDYSPNYDLTVVTVNDHVSTTK;VTVHEVNADLYLCKPR;YTDIEYK | 0.2459 | 37.9987 | 13 | NA |
| 3H-77 | MAFVHLFHIDGILECAER;QWATDELK;VAALKQWATDELK;VHGWWWR;VQKVHGWWWR | 0.4940 | 21.1386 | 13 | NA |
| 3H-84 | LLSPPVDPHVFIDTTAASIDK;NSEEDSSPAQADQSESVGSSPPTK;SIDTNEGNDDDSTAIALR;SPHLDMAFR;TGTEPKPEHSDDGKNSEEDSSPAQADQSESVGSSPPTK;VVAKPQYDDR | 0.4156 | 27.4577 | 13 | ABC-type transport system permease |
| 3H-135 | ALNMILK;DQVPVLTK;KRPIQPTAYNLFYK;RPIQPTAYNLFYKDQVPVLTK;SNSNNTMTALDGVHQINSLVTSVFSDSEHINEWLSLSK;YLLDNYNYR | 0.6080 | 23.4889 | 12 | yabby-like transcription factor |
| 3H-3 | DYFGWDKLEAAYNAK;FNHGTIEHPNCPGDVLPDDCSK;IIQIAPVDLIK;TLLKPTVHLNR;YANVNGFEGEFNSQYSVQSYNK | 0.3971 | 22.4439 | 12 | NA |
| 3H-44 | DNSEASSQNTTVWDR;DNSEASSQNTTVWDRR;TANTSTNTSAAATSGK;TANTSTNTSAAATSGKDNSEASSQNTTVWDR | 0.3168 | 18.9994 | 12 | NA |
| 3H-28 | GIDDGDAIYTITK;IFMLVLLER;LDEHVPPVAAFIR;LTEWFPNNR;NITLSPQYCSEYLTLK;NNLNYDQLYTALR;TAYDQADVIAYIDGDKEK | 0.2571 | 31.6873 | 11 | NA |
| 3H-116 | ETFYQVFSQVTSVDHR;FGCLEYR;IAFVGKPGTGK;LWEFADDFPNK;LWEFADDFPNKETFYQVFSQVTSVDHR;TCLDGIFLFGTGNHDVR | 0.1742 | 27.0472 | 10 | ATPase involved in DNA metabolism |
| 3H-118 | LDSFVGPTGNFHGMNVTMK;LNGQLHTLVIPK;RYAYTDSDGVYVK;SIVAAFATADLPTALYHSETATYR;YAYTDSDGVYVK | 0.4048 | 21.1119 | 10 | NA |
| 3H-137 | AMSNGLGDVILSAK;DTMAMIFK;IFDAFKR;KIFDAFK;KVSGDVIDK;TGVDLSVNVGQTYK;VSTTVDPMMPAQVK | 0.4188 | 26.7878 | 10 | hemolysin-like protein |
| 3H-150 | LAVEYNGR;LQVIFDDNDEKR;NIDLIDVPYTIPIDEIPNFIDK;QHYEYVPR;SADDALLDPTTSPSPVFAR;VRPDWLINPNTK | 0.3476 | 28.4991 | 10 | NA |
| 3H-139 | AHIAPALLNHIESNLLK;ATLDEVSNDPDDIDVER;ATLDEVSNDPDDIDVERR;FVFSARPTVDSSLASGGVR;HHISPEMYVTMVK;QTTVDKWIDSR | 0.2816 | 26.7690 | 9 | putative S1/P1 nuclease |
| 3H-63 | FLILSASGPLK;GSVSDLEFK;GTCAMTLLQQLK;HYTGIIPETMNVAK;NVGLFIPK;SGDEIDGYIIMKPK;YLWTFAPLLEQR | 0.2658 | 33.2489 | 9 | NA |
| 3H-171 | DFLTLFDVDELR;DFLTLFDVDELRDKPK;IASHQLACLNYTLPVR;ILIVINQR;LGFQEFVYR;LKDFLTLFDVDELR;LMHYGDAPNDVTPSK;MFVVQTNR | 0.2357 | 37.9987 | 8 | caspase-like protein |
| 3H-4 | LSHDYAYSLR;MDDAPKDFNEFIVWVPTK;NQPNNYVMVR;NSDFAWK;NVTFYVTIK;SSTVVSTVSSATWVK;VYTNVSDIPSAEK | 0.3475 | 26.8153 | 8 | NA |
| 3H-46 | HSDDIDTTIDALVK;KLDQEVLIQEMR;LDQEVLIQEMR;YMEVSSNDTLLYDNVR | 0.1634 | 18.9994 | 8 | NA |
| 3H-59 | ALDELMDFK;ALDELMDFKK;DNEISQLHEELDYATETINDLNDQLR;HVSAEIELK;KDNEISQLHEELDYATETINDLNDQLR;TAAQQLASYR | 0.3256 | 27.45765 | 8 | putative nucleic |
| 3H-79 | FPFWFER;NEPVSYLAVGNAQFIDR;RNEPVSYLAVGNAQFIDR;SGLNYIITFDSSATEK;SPNCALVFTR;VPVSNTVEFVIVILK | 0.5280 | 23.7563 | 8 | NA |
| 3H-136 | EFAVWNLCR;KPVAVFR;NISYDWGSFLEAVVSR;TFEEFR;VFGSLAFNTTTVATILNYDR;WNLRPR | 0.0650 | 18.6338 | 7 | lipopolysaccharide modifying enzyme |
| 3H-182 | APQQIATVK;DVEFVAR;HTGGLIITAPLVHPAVGTTR;SLPDPLDIYVR;TQHHNEEVETVDQSILAHVR;VFECPNTEEVPNFR | 0.2051 | 23.2135 | 7 | NA |
| 3H-26 | ANSIIDNIER;QIVDPNATMK;SGATYAINNSYTIDALR;TSEIVDVVDATR;TSEIVDVVDATRK | 0.3731 | 21.8734 | 7 | NA |
| 3H-72 | DQPMAGAVVETTGDWR;ELQPPYSNYFEESLKPEAFVSNIHYK;LSSLLTSIVSSHSGMIHDK;LSSLLTSIVSSHSGMIHDKR | 0.4000 | 18.9994 | 7 | phenylalanyl-tRNA synthetase α subunit |
| 3H-83 | AVTVTKGIAR;ESFGTYMGQR;KPAQELIHEYR;MSPELQEAWR | 0.2697 | 15.4288 | 7 | glycosyltransferase |
| 3H-88 | FVSNTQQLVENSLIYGGLR;ITPVTFMTTVER;LAAHMYMTYLR;NIIPTPLLHHFER;QISHVVYADWLTER | 0.2654 | 22.2974 | 7 | putative S1/P1 nuclease |
| 3H-113 | AAGFIVIEQDINTDNVTLVHEALR;AFMELIR;LYNTAFTMATSAVLAPATLTAR;RLDIAFSGTDK;TTLGGSTDSVMR | 0.2203 | 21.9188 | 5 | lysophospholipid acetyltransferase |
| 3H-115 | LESVENVINAFNLR;LITATEEELK;TPQLQEDLASYTK;VAGIENLVLQTK;VCSIPTNEDNPDK | 0.1033 | 21.6711 | 5 | NA |
| 3H-15 | DGTVVISEDDKHNEPYGIGK;FFNFKEDFTR;TIEALISLPR;VIQTPDPQPVVEGK;YYVDDLVK | 0.2088 | 21.4025 | 5 | NA |
| 3H-21 | EVEDLVLR;IASLLVHDIHSSLDDAVK;IGSIAPIELNPPPR | 0.1754 | 11.7657 | 5 | NA |
| 3H-24 | FIIDYIR;SANTTSVNAVNYR;VNADSVYTDETAANVHR;YGNVDSQYYK | 0.0646 | 16.4322 | 5 | RNaseIII |
| 3H-82 | GGHGSVGEVHTSAVVR;LGYMAAGYESTYESVVR;TVSAVGSNEELANTDDSSR;VCAAFQIKPR | 0.1896 | 16.9790 | 5 | NA |
| 3H-124 | INDPVLVIDSAR;LSRPPGIDGDPNK;TLLEYNSLYR;TTGYQQLYAELK | 0.0716 | 14.6310 | 4 | serine/threonine protein kinase |
| 3H-128 | ELAIGIDER;QTSSSQQRPNDLLYPLSYGAVDETVVLK;YADIPVWEQGVEDEPTSETNDTLNR | 0.2313 | 14.2495 | 4 | NA |
| 3H-157 | DLPDTVPVSLDVR;GINDLSDAELAYK;NPPLELWSR;VLIDTEQKR | 0.2543 | 17.4378 | 4 | tyrosyl-DNA phosphodiesterase |
| 3H-16 | QLADGLGFILSK;YLSFVEPK;YVDLLDPFGNSIPVTGR | 0.0565 | 13.2081 | 4 | lipase like protein |
| 3H-23 | GMENLAEATLEALAK;SPLNYSHENIER;VVVNSDETVYHAR;YVFNVDSVK | 0.0633 | 13.6524 | 4 | NA |
| 3H-76 | DLEGIVMR;EFPLVNPAR;ILFEYAPVNVYYSTIESR;SLWSTDETLPR | 0.1139 | 14.6554 | 4 | NA |
| 3H-8 | LNAQDVAVLGTSNPILR;QLQNMYIVVDEFLK;VIPTTATYQICDDDDRFLFQR;VYIPVPPITR | 0.1425 | 14.6304 | 4 | NA |
| 3H-90 | DLHRLELPEETFIK;TAEAWYK;TGASTGYSSDSPSR;YDMLELLK | 0.1581 | 17.5476 | 4 | *iap*-like protein-5 |

Table S12 Identified HvAV-3h coded proteins from *Spodoptera frugiperda* produced virions (Sf-2)

| Protein ID | Identified peptide sequence | Sequence coverage | Protein Qscore | Unique spectra | Function annotation |
| --- | --- | --- | --- | --- | --- |
| 3H-57 | AKLDLVTINSVANK;ALLEVTDNVVTDR;ALLEVTDNVVTDRVPVR;AQLDVADLER;AQLDVADLERR;AQLQSDLDVLR;ATIDALTANLNSSNSK;CQAELQQR;DEAIWAIK;DQLLKLWR;DSSFPIVDADNTEQLVVR;EHDVLER;EKAQLQSDLDVLR;EKQHAEFLR;ELACNVIPEKCGDITQMNR;EMYAVLEDELK;FQMHITNDFLDLVQPEGIATLASYLSVSESQISDR;GGMGVLDDIYNR;HANDVR;HIAGVITK;HVGNAQSDGMYTENVALR;IESEAALVESLQR;IQAFVNTEQNILAAGTR;ISELLTQLQESNNQR;ISNSVKNDVESK;ITNEFFDHATLPLLR;KQMGNVVQEIAQR;LDGSDVTLVVDDENLK;LDLVTINSVANK;LIDSVVK;LIDSVVKITR;LLISSETLGR;LLKEHDVLER;LLSLDGVR;LLSLDGVRR;LLVSEDK;LNKELNTVR;LNNELETSITEER;LQLDYMTLQQR;LSNASVGVQLDR;LTNECQAVSSR;MEFEAVNDALSK;MLELQNLMK;MTAAHLADDR;MTAAHLADDRLSNASVGVQLDR;NAEITNLQDR;NELAATVNSLQMQATK;NIDGAEGFTR;NIDGAEGFTRDEAIWAIK;NISAIRPR;NNVQLQTALQR;NSGVGNAINDR;NVNDTTLAELAQSR;QHAEFLR;QHPSFANKPNNTDVQQSDLTIAEDADTGTSVTK;QLNALNLR;QMGNVVQEIAQR;QQLEWILVNSNPAIALQDASVR;QSLLETSAQLNDVR;RLNNELETSITEER;RNNVQLQTALQR;RPSVTDDEDDRIVGTR;RSLSYSLTDDEDDAAQIR;SETNALVVEVADLK;SETNALVVEVADLKSQLNR;SEYELSASR;SLSYSLTDDEDDAAQIR;SNANLSPQSIR;SQALYAIK;SYIIELESR;TKEMYAVLEDELK;TMGACTAVGDVNECAR;TNDNLEIK;TQLLTGLR;TYQQEMTQLK;TYQQEMTQLKR;VAYANLEGDMQK;VITGDETVMTYNR;VSTDDLTSYSR;YEALMSEQSSK;YESLMTK | 0.5737 | 333.2961 | 179 | chromosome segregation SMC like protein |
| 3H-53 | ALFFGVR;DSGVALPIGMMVNNR;ENVPDTGAGTGFMNMLTDGDFATPPEITK;GQYMYSFALDLK;GSINPSNFNSSISVVIEPSEALR;GTIDSYIYGPDEQVTTYFVR;KIDNGGDFN;KLSDLLIK;LSDLLIK;MPVLLSTGNGSNK;NITHSEYLDYYEVGLPK;RGTIDSYIYGPDEQVTTYFVR;SIGVSALSK;SIGVSALSKIGIK;SVDIGAKTLVLPIPLYFSR;SYAAGTESSYFPK;TLVLPIPLYFSR;TSCSPHYILMEKPTDIAATEILKPTEAGSTLSYDIEHSSGILK;TVDPKGSINPSNFNSSISVVIEPSEALR;VPMLPAEHFVFTEPYR;VTVEFVFR;YRVPMLPAEHFVFTEPYR | 0.6000 | 92.1578 | 71 | major capsid protein |
| 3H-152 | ASYMNLLIVEQDR;AVHCLQSSTADPR;DMVSPSNADGPR;DNTSYFDPLR;EHITFNPGR;EISFDMFDALYGNGDDADVR;EKVVQGATAVR;ERNHIALFMVR;FIYEYNNYTVR;FNLLHDTINSLFEAGVVDR;FVEHCIIDAYTSNR;FYTEHLHTDELLNINVR;GAVQVGPNAAIEAVMR;GTLMSELFTVDER;HDDTNTVIVQYSVDK;HDDTNTVIVQYSVDKHYLSR;HPPTADTALSAMSR;KGTLMSELFTVDER;LIGMDGVQLETIR;MSAYETSEIR;MSAYETSEIRK;MVWFPEFVSPVSDDAPR;QLSAHWR;RELLNPEHSAK;RFNLLHDTINSLFEAGVVDR;RVDDAELER;SIINVNNYAR;SLSDAVQMVK;SQTFYNTQR;SQTIDGVGR;STDTNSATFYPK;TSPTLILHVWR;TVSLFRPPLSLK;TYQALLPQPMR;VDDAELER;VLLHLTAYTILK;VLPLTAFGK;VLTDSVDTAR;VVQGATAVR;YGEWLK | 0.3294 | 165.0281 | 60 | NA |
| 3H-50 | ASCGTIPDAR;ATHYDPIR;ATHYDPIRDGYYMYALPNGR;DGYYMYALPNGR;DGYYMYALPNGRVDR;DYFLSSK;DYFLSSKYK;FLKPTFWFSR;LPSDSGVAVTTSPTVVDDLDLR;NALVALSNSSMNNEQNYTNR;YNNLSWLLSK | 0.5503 | 45.3160 | 54 | NA |
| 3H-56 | DMNGIAVSVDAK;EEDGWDVATTEK;IMNGAGAAGGAVVPK;LKEEDGWDVATTEK;LQPAPLDIK;LTNDEMNR;LYPALMSPNTITNVIPDAR;LYPALMSPNTITNVIPDARNGVHSTDR;MELHFEFM;SSVMVIGLSEVFMQQTSVTFR;TIIGDLGSLQGPEGGEWLAFFYPAHTVFEK;TIIGDLGSLQGPEGGEWLAFFYPAHTVFEKMR;VGETPDLSSFSQEEISK | 0.6736 | 54.7052 | 45 | NA |
| 3H-27 | ALINEDGHSVVTQR;AMIESILK;FSVLNSPLSR;FTDEIIKK;GVVWSEQSQQLVAAAATTK;HIHSVGDPSYNDEVTR;HSNVYSDALESFR;KVDEFISFGR;LYSTANELLADVLYGDGVDLGSTVSETTNR;NAFTSIIWNVDIFK;NLCEIGVVSNVTDVDLLR;RDPALYEVQLHTCR;RPDAEECFK;RYAIDVNILPAISDTIQK;SLVLHFGANPAYTIMNTFASPAESPR;SLVVLTLQHELR;SLVVLTLQHELRK;SPPTLTIDDDSILYPLQYIPR;STEDDRPIQMQR;VDEFISFGR;VLTAALMHTDADTVNPTTPSVGYESDAVPR;YAIDVNILPAISDTIQK;YVDMSQYR | 0.4097 | 92.5976 | 37 | NA |
| 3H-55 | AYELANER;AYELANERGPFSVEVYLNPGTSNTYQYVATTR;FNNTDYDDLPWNYTSGK;FNNTDYDDLPWNYTSGKK;GIASLWGYLK;GPFSVEVYLNPGTSNTYQYVATTR;IGQTPKAYELANER;KVVTATGVVSGGER;LPQSFTR;NKFNNTDYDDLPWNYTSGK;SEIAQDIQVTMYNTNGGSNPLNNSNVTR;SNVDSSSYYQQPPQVVYNNGDLYGSR;TGYSGAELGASIDK;TGYSGAELGASIDKGIASLWGYLK;VVTATGVVSGGER | 0.6933 | 63.2588 | 32 | NA |
| 3H-140 | ALSHPDWLTIGSFIK;DVDGVASNGGGSVGTGLGGPLGGTSGVNTSNLR;ELFYHSEAGR;ETNYPIPYFQYNLVLSQR;EVAKELFYHSEAGR;QLCNAIKALK;SGDEMTLSR;STGTTVGYTGTTSNAAMK;TATHLLGYK;TLDRPTDNAIMQR;YTYMYVNK;YTYMYVNKR | 0.6981 | 51.4074 | 31 | NA |
| 3H-13 | AAVWLATTTGDDSGYR;AVMNSFR;CVDEFR;DIISPLILLDAER;DVIHEHVVAHWNDAETEQIIGR;ESPTEYFLR;EWNATFVVIDEAHDLSVHGVDYPK;FAGLVSYLPSIVDVNEVDIVNVGER;FVFPDGTYGAEGYATWMDSK;HLNDFEIDR;LSTHDITVEQFNR;LTTYAEFNETQLLYSDDDDRDR;NNLSDLVPLHNLLMK;NTVIVVHDMSDVMSATYHYAGAGER;QRPTFVYPFVLDNVSLYGLDAYR;RPMPHQILVANYTTPR;SFIAQLDR;SKLEELGAVITDVTCGVQAK;SPIDGIIVIHGVGTGK;SSIEAQLNNDMLAK;TFFFNTITAFANTVSR;TVDILMYATSEAK;VIAEGITLK;YAWIAK;YQSHAYTIADWGSDAKPNVFVYR | 0.4131 | 103.0921 | 30 | DEAD-like helicase |
| 3H-58 | AVSPLTNKPIDVHGR;DCNLSPPPTK;HIVVSNGFQPVR;LGGLSPYR;LTAAQCETFIR;MVYSMCHGVPYR;NDQNVPTYYTSSRPFADR;NESINPITGK;QSMTAMR;SIRPEQAAYR;SVSPYSGRPIK;SVSPYSGRPIKR;TAANADTRPDLCATFSR;VAQNTTQSQLLNIVK;VLLSPIPDGATPMSR;YMINPNFVPTTTK;YMINPNFVPTTTKK | 0.3034 | 71.3234 | 30 | cofactor of the virion S/T kinase encoded by thioredoxin |
| 3H-48 | ALLLSQLVSPPLIAK;AMHLLYIEGK;AMHLLYIEGKYLR;ATQNTVQTLR;CLGQVVTNVR;CSSTAAVDAAVASSSTEALSQK;LQSWVTDHEAVQYGTKPTVPYVIPDNLLSQKPTDNTISR;SVASVSSPGNIASSSVGNK;SVASVSSPGNIASSSVGNKR;TAQSIMQNLMTQIK;THIFNYLDSK;TVNVNQDSQTIK | 0.3395 | 50.2295 | 25 | NA |
| 3H-151 | ALQYQTQLEGLLSR;CKPGEVMDDK;ETCALTTVTASLATISESLGK;ETCALTTVTASLATISESLGKDEALLK;LVQIDNTVK;RVVVDPLDTK;SSLTSVVFTPLSEK;SSLTSVVFTPLSEKLDAIK;VAFGLTR;VVVDPLDTK;VVVDPLDTKVEK;VVVVPVQDK;VVVVPVQDKITEVGGGVAAIK | 0.5911 | 51.5008 | 24 | Uyr/REP helicase |
| 3H-107 | FFPAETHVEVK;FYSPSCGACEHVK;IDHVPSAVVK;LQLSDWNDNVTTIK;LQLSDWNDNVTTIKK;NYVLNEPPAR;RIDHVPSAVVK;TNTPLEGVPEFVLFHYGNAVK;YASDSLMYFECDLSIR | 0.5951 | 37.7815 | 21 | thioredoxin-like protein |
| 3H-110 | ADGTAIR;ATDINPYETK;FLTNEIK;GWVPVNGSDAFEFSCK;HLNPQWATDAPILK;IGFISQILPR;KATDINPYETK;KNVDDDTIELR;LGNVSGIITK;LSFDKK;LTFNGTAK;TSNKPLIFECILDEPEK;TYKHLNPQWATDAPILK;VVLAPEK;VVLAPEKVYEFISR;VYEFISR | 0.6244 | 62.4228 | 21 | NA |
| 3H-117 | CIPVVAYHAQDADAPTDTGLK;DDAIIIDDLPDVYITQPHR;DYAAVITAELILR;LDSAESEMAR;LEMFLSSDETR;NLNALWDLWGADVIIER;NVNGFVPAVIFSDYEVYK;RLEMFLSSDETR;RPHLDEFLSYLFENFR;SRPTVFLDLDNTLICSLR;TAGEEVGGVK | 0.8148 | 49.9845 | 17 | CDT phosphatase transcription factor |
| 3H-62 | AYVDLKDFVVTHENLFAR;DFVVTHENLFAR;KTQTPVNILADIVSLK;LDNNFVDGLK;LFVPGIDLPQPYIGIDTTKR;LITDPYQLLQISER;RLFVPGIDLPQPYIGIDTTK;TQTPVNILADIVSLK;YTTSTVPNETDRR | 0.3116 | 37.3139 | 16 | myristylated membrane protein-like protein |
| 3H-19 | ATVGALAGDVVKR;ESTTNIAEDIEREPAYLK;FDLLTVDGNDAR;IFSMLDNEIHLTIDSSDHQHVLQIYNR;LLYWLEDNR;LLYWLEDNRFDLLTVDGNDAR;QTLGLVIK;SETTDASNVSPR;SHVDDNEDLAPSK;SYADELGDR;YMSNYVPHDDGNVELDDLQLIQPLEEVSR;YRAELEGVADNIK | 0.2523 | 48.2709 | 15 | NA |
| 3H-71 | KYCADIFDIVQHAASL;LGKPIVVIAPVR;LSESVAVIEAK;LSESVAVIEAKR;NEVAWLLR;RNLFTNIR;SRNEVAWLLR;VQFPHVVHNPELNVIYWR;YCADIFDIVQHAASL | 0.3606 | 34.0347 | 14 | sulfhydry1 oxidase Erv1 like protein |
| 3H-43 | AVVFSDSR;DLYNLQILR;DQSLELLSHTHR;FGLASVNDENSYK;FVEFGTLVRPAGWGSLSELGK;LSLGYQTVVQFYR;MFNVDQAIEFFENR;RFGLASVNDENSYK;VCHLNAVLDPAFTIDTGNVR;YNSSMDEYR;YNSSMDEYRDQSLELLSHTHR | 0.5150 | 45.8864 | 13 | NA |
| 3H-109 | ALIVKPMNQFR;EVHVSVTTLMK;IAQYPINTK;IDYKETQR;ILSLDAVSVPIDQK;LTFFKK;QAVSSSGCGGGSTYR;SQLHFETHESK;SVIPTNATTNAYNAVK;VALIMSIDAVIR;YLDSLIPTPR | 0.2993 | 44.9038 | 12 | cathepsin B |
| 3H-135 | ALNMILK;KRPIQPTAYNLFYK;RPIQPTAYNLFYK;RPIQPTAYNLFYKDQVPVLTK;SNSNNTMTALDGVHQINSLVTSVFSDSEHINEWLSLSK | 0.5360 | 18.9967 | 11 | yabby-like transcription factor |
| 3H-137 | AMSNGLGDVILSAK;DTMAMIFK;IFDAFKR;KVSGDVIDK;NALLTLK;SEDEEVSAEIVTAK;TGVDLSVNVGQTYK;VSTTVDPMMPAQVK;YSVAVAK | 0.5875 | 31.2888 | 11 | hemolysin-like protein |
| 3H-37 | EFFTLNEDNFTELIK;IDALDDFEVWR;IGYTCNLNDR;LSELNVASAYDFRPVFVVPTK;LSELNVASAYDFRPVFVVPTKK;QLEAVLHTK;QTLEEQLPR;YAAEFR;YWLVNELFPSLK | 0.3264 | 38.6135 | 11 | bro4 |
| 3H-84 | ASIQSGYNK;LLSPPVDPHVFIDTTAASIDK;NMVINEPQYQQFLR;NSEEDSSPAQADQSESVGSSPPTK;SIDTNEGNDDDSTAIALR;SPHLDMAFR;TGTEPKPEHSDDGKNSEEDSSPAQADQSESVGSSPPTK;VVAKPQYDDR | 0.5152 | 32.7670 | 11 | ABC-type transport system permease |
| 3H-118 | LDSFVGPTGNFHGMNVTMK;LNGQLHTLVIPK;RYAYTDSDGVYVK;SIVAAFATADLPTALYHSETATYR;YAYTDSDGVYVK | 0.4048 | 21.0795 | 10 | NA |
| 3H-69 | AAILLGASLVLNK;DYDTGKDESHYMLYTK;LVYKDYDTGK | 0.4231 | 12.5907 | 10 | NA |
| 3H-77 | MAFVHLFHIDGILECAER;VAALKQWATDELK;VHGWWWR | 0.4578 | 13.6321 | 10 | NA |
| 3H-139 | AHIAPALLNHIESNLLK;ATLDEVSNDPDDIDVER;ATLDEVSNDPDDIDVERR;FVFSARPTVDSSLASGGVR;HHISPEMYVTMVK;QTTVDKWIDSR | 0.2816 | 25.9648 | 9 | putative S1/P1 nuclease |
| 3H-3 | DYFGWDKLEAAYNAK;IIQIAPVDLIK;LEAAYNAK;TLLKPTVHLNR;YANVNGFEGEFNSQYSVQSYNK | 0.2892 | 22.7202 | 9 | NA |
| 3H-63 | EYVFGVR;GTCAMTLLQQLK;HYTGIIPETMNVAK;KNVGLFIPK;LWNASVDSFCYR;NVGLFIPK;SGDEIDGYIIMKPK;TAFSIVNK | 0.2525 | 27.2989 | 9 | NA |
| 3H-66 | DIIMGHISPNR;DTFAVSPAR;IFLATNTNSHVR;KVQVDYSPNYDLTVVTVNDHVSTTK;TADGDYDDDER;VQVDYSPNYDLTVVTVNDHVSTTK;VTVHEVNADLYLCKPR;YTDIEYK | 0.2459 | 30.4244 | 8 | NA |
| 3H-28 | GIDDGDAIYTITK;IFMLVLLER;ITALYHNVK;LTEWFPNNR;QHLLDER;TAYDQADVIAYIDGDKEK | 0.1836 | 22.2370 | 7 | NA |
| 3H-88 | ASFDTVPWVDGR;FVSNTQQLVENSLIYGGLR;ITPVTFMTTVER;NCHTNAASALEAR;NIIPTPLLHHFER | 0.2654 | 22.7202 | 7 | putative S1/P1 nuclease |
| 3H-113 | AAGFIVIEQDINTDNVTLVHEALR;AFMELIR;LLTGTQGK;LMYDPAILNR;MHAIIPSMVTALTALVSVR;RLDIAFSGTDK | 0.2290 | 21.6118 | 6 | lysophospholipid acetyltransferase |
| 3H-150 | LAVEYNGR;LQVIFDDNDEKR;NIDLIDVPYTIPIDEIPNFIDK;QHYEYVPR;SADDALLDPTTSPSPVFAR;VRPDWLINPNTK | 0.3476 | 21.8667 | 6 | NA |
| 3H-46 | HSDDIDTTIDALVK;KLDQEVLIQEMR;YMEVSSNDTLLYDNVR | 0.1634 | 13.6321 | 6 | NA |
| 3H-72 | ELQPPYSNYFEESLKPEAFVSNIHYK;LSSLLTSIVSSHSGMIHDK;LSSLLTSIVSSHSGMIHDKR;QEYEDDDYNFK;VTTSSILR | 0.4194 | 21.6788 | 6 | phenylalanyl-tRNA synthetase α subunit |
| 3H-126 | ALNTHDAGAYK;APVTATPVPVKPLYTAR;DTLTLR;STAAAITETVAGILK;WTLEVINWR | 0.0551 | 20.4373 | 5 | ATPase involved in DNA replication |
| 3H-157 | EQITQWLR;GINDLSDAELAYK;NPPLELWSR;NSTMNLPR;VLIDTEQKR | 0.2717 | 18.1392 | 5 | tyrosyl-DNA phosphodiesterase |
| 3H-171 | IASHQLACLNYTLPVR;LGFQEFVYR;LKDFLTLFDVDELR;LMHYGDAPNDVTPSK;MFVVQTNR | 0.1975 | 21.6788 | 5 | caspase-like protein |
| 3H-26 | ANSIIDNIER;SGATYAINNSYTIDALR;TSEIVDVVDATR;TSEIVDVVDATRK | 0.2985 | 18.1762 | 5 | NA |
| 3H-4 | DFNEFIVWVPTK;NSDFAWK;NVTFYVTIK;VYTNVSDIPSAEK | 0.1737 | 15.3694 | 5 | NA |
| 3H-44 | DNSEASSQNTTVWDR;DNSEASSQNTTVWDRR;TANTSTNTSAAATSGKDNSEASSQNTTVWDR | 0.3168 | 13.6321 | 5 | NA |
| 3H-76 | DLEGIVMR;EFPLVNPAR;FNEYDATLR;IAMHGDVSR;ILFEYAPVNVYYSTIESR | 0.1312 | 17.0930 | 5 | NA |
| 3H-116 | ETFYQVFSQVTSVDHR;FGCLEYR;LWEFADDFPNK;YIMYTK | 0.1124 | 14.8903 | 4 | ATPase involved in DNA metabolism |
| 3H-128 | DVISDQPR;QTSSSQQRPNDLLYPLSYGAVDETVVLK;YADIPVWEQGVEDEPTSETNDTLNR | 0.2276 | 10.4308 | 4 | NA |
| 3H-15 | FFNFKEDFTR;KTIEALISLPR;TIEALISLPR | 0.0707 | 12.5907 | 4 | NA |
| 3H-156 | DLFNFVSQTR;DTGHLVAFINGGTR;QTVLAQSNSGAYPVER | 0.1042 | 11.0331 | 4 | NA |
| 3H-182 | EGTMTVANHMGSATR;SLPDPLDIYVR;TQHHNEEVETVDQSILAHVR | 0.1165 | 10.3463 | 4 | NA |
| 3H-23 | FAITGSR;INYAATVNNNAHR;SPLNYSHENIER;YGTFAVDSAR | 0.0543 | 14.8965 | 4 | NA |
| 3H-81 | LFSTSTNR;VITDNIVGTLNLLSR;VTMFAPVYAYR | 0.1393 | 12.0716 | 4 | NA |

Table S13 Identified HvAV-3h coded proteins from *Spodoptera frugiperda* produced virions (Sf-3)

| Protein ID | Identified peptide sequence | Sequence coverage | Protein Q score | Unique spectra | Function annotation |
| --- | --- | --- | --- | --- | --- |
| 3H-57 | AEMQINTQR;AKLDLVTINSVANK;ALLEVTDNVVTDRVPVR;AQLDVADLER;AQLQSDLDVLR;ATIDALTANLNSSNSK;CQAELQQR;DEAIWAIK;DSSFPIVDADNTEQLVVR;EHDVLER;EKAQLQSDLDVLR;EKQHAEFLR;ELACNVIPEKCGDITQMNR;EMYAVLEDELK;FQMHITNDFLDLVQPEGIATLASYLSVSESQISDR;GGMGVLDDIYNR;HENLYLASTTPSR;HIAGVITK;HVGNAQSDGMYTENVALR;IESEAALVESLQR;IQAFVNTEQNILAAGTR;ISELLTQLQESNNQR;ISNSVKNDVESK;ITNEFFDHATLPLLR;KQMGNVVQEIAQR;LDGSDVTLVVDDENLK;LIDSVVK;LIDSVVKITR;LLISSETLGR;LLKEHDVLER;LLSLDGVR;LLSLDGVRR;LNDIAELQSQLTK;LNKELNTVR;LNNELETSITEER;LQLDYMTLQQR;MEFEAVNDALSK;MLELQNLMK;MTAAHLADDR;NDVESKK;NELAATVNSLQMQATK;NIDGAEGFTR;NNVQLQTALQR;NSGVGNAINDR;NVNDTTLAELAQSR;QHPSFANKPNNTDVQQSDLTIAEDADTGTSVTK;QIDSLR;QLNALNLR;QMGNVVQEIAQR;QQLEWILVNSNPAIALQDASVR;QSLLETSAQLNDVR;RLNNELETSITEER;RNNVQLQTALQR;RPSVTDDEDDRIVGTR;SETNALVVEVADLK;SLSYSLTDDEDDAAQIR;SNANLSPQSIR;SQALYAIK;SRSDVVVLR;SYIIELESR;TNDNLEIK;TQLLTGLR;TYQQEMTQLK;TYQQEMTQLKR;VAYANLEGDMQK;VITGDETVMTYNR;VSTDDLTSYSR;YESLMTK;YQAAGTPGNGR | 0.5413 | 287.4801 | 152 | chromosome segregation SMC like protein |
| 3H-53 | ALFFGVR;DSGVALPIGMMVNNR;EIRPCAAFSK;ENVPDTGAGTGFMNMLTDGDFATPPEITK;FEVVADFAVVTDFEIDR;GQYMYSFALDLK;GSINPSNFNSSISVVIEPSEALR;GTIDSYIYGPDEQVTTYFVR;KIDNGGDFN;KLSDLLIK;LSDLLIK;MPVLLSTGNGSNK;NITHSEYLDYYEVGLPK;NRGQYMYSFALDLK;RGTIDSYIYGPDEQVTTYFVR;SDVNKR;SIGVSALSK;SIGVSALSKIGIK;SVDIGAKTLVLPIPLYFSR;SYAAGTESSYFPK;SYAAGTESSYFPKLAHK;TLVLPIPLYFSR;TSCSPHYILMEKPTDIAATEILKPTEAGSTLSYDIEHSSGILK;TVDPKGSINPSNFNSSISVVIEPSEALR;VPMLPAEHFVFTEPYR;VTVEFVFR;VTVEFVFRK;YRVPMLPAEHFVFTEPYR | 0.6835 | 110.4096 | 86 | major capsid protein |
| 3H-27 | ALINEDGHSVVTQR;ARDDNSPSNDSVAMK;DPALYEVQLHTCR;FNIDNVNYASVAHAIALISVVYHMK;FSVLNSPLSR;FTDEIIK;FTDEIIKK;GVVWSEQSQQLVAAAATTK;HIHSVGDPSYNDEVTR;HITYVVVGHALVSHR;HSNVYSDALESFR;IVTSQLLYK;KADNVWVYTEDLQAIK;LYSTANELLADVLYGDGVDLGSTVSETTNR;NAFTSIIWNVDIFK;NLCEIGVVSNVTDVDLLR;RDPALYEVQLHTCR;RYAIDVNILPAISDTIQK;SLVLHFGANPAYTIMNTFASPAESPR;SLVVLTLQHELR;SLVVLTLQHELRK;SPPTLTIDDDSILYPLQYIPR;STEDDRPIQMQR;TGYANLK;TLFGDVVQK;VDEFISFGR;VLTAALMHTDADTVNPTTPSVGYESDAVPR;VNPWMFAELAMLMR;YAIDVNILPAISDTIQK;YKHFDIR;YTDFDQLWSK | 0.5428 | 131.7952 | 51 | NA |
| 3H-50 | ASCGTIPDAR;ATHYDPIR;ATHYDPIRDGYYMYALPNGR;DGYYMYALPNGR;DGYYMYALPNGRVDR;DYFLSSK;DYFLSSKYK;FLKPTFWFSR;LPSDSGVAVTTSPTVVDDLDLR;NALVALSNSSMNNEQNYTNR;YNNLSWLLSK;YNNLSWLLSKNVYK | 0.5714 | 47.9372 | 50 | NA |
| 3H-152 | ASYMNLLIVEQDR;AVHCLQSSTADPR;CPGNLEAMLAVQR;DMVSPSNADGPR;EHITFNPGR;EKVVQGATAVR;ELLNPEHSAK;ERNHIALFMVR;FIYEYNNYTVR;FNLLHDTINSLFEAGVVDR;FVEHCIIDAYTSNR;GTLMSELFTVDER;HDDTNTVIVQYSVDK;HPPTADTALSAMSR;IYDAPNTNVLYGIANDITSHMIQYK;KGTLMSELFTVDER;LIGMDGVQLETIR;MSAYETSEIR;RELLNPEHSAK;RGIYSPGMTYDIAHVYPAPGVGYTISVLSNNAPTVDVPR;RLVYNASLLLR;RVDDAELER;SEELDILK;SLVIGAEQLER;SQTFYNTQR;SQTIDGVGR;SWVDRDPESNTK;TSPTLILHVWR;VLLHLTAYTILK;VLLHLTAYTILKK;VVQGATAVR;YETDFTSDESPFSIVTSIDK | 0.3015 | 130.5320 | 41 | NA |
| 3H-140 | ALSHPDWLTIGSFIK;DVDGVASNGGGSVGTGLGGPLGGTSGVNTSNLR;ELFYHSEAGR;ENFVNLVELWCK;ETNYPIPYFQYNLVLSQR;GSVSKSGDEMTLSR;NSDKDVTIPK;SGDEMTLSR;STGTTVGYTGTTSNAAMK;TATHLLGYK;TATHLLGYKNK;TLDRPTDNAIMQR;YTYMYVNKR | 0.7689 | 53.6172 | 35 | NA |
| 3H-13 | AAVWLATTTGDDSGYR;CVDEFR;DIISPLILLDAER;DPYDTLNK;DVIHEHVVAHWNDAETEQIIGR;ESPTEYFLR;EVMTVPSIVSALK;EWNATFVVIDEAHDLSVHGVDYPK;FAGLVSYLPSIVDVNEVDIVNVGER;HLNDFEIDR;KVLLLTATPMR;LSTHDITVEQFNR;NELNCYYR;NNLSDLVPLHNLLMK;NTVIVVHDMSDVMSATYHYAGAGER;QRPTFVYPFVLDNVSLYGLDAYR;RPMPHQILVANYTTPR;SFIAQLDR;SKLEELGAVITDVTCGVQAK;TFFFNTITAFANTVSR;TVDILMYATSEAK;VLLLTATPMR;YQSHAYTIADWGSDAKPNVFVYR | 0.3559 | 90.6628 | 33 | DEAD-like helicase |
| 3H-56 | DIHKFNGSK;DMNGIAVSVDAK;EEDGWDVATTEK;IMNGAGAAGGAVVPK;LKEEDGWDVATTEK;LQPAPLDIK;LTNDEMNR;LYPALMSPNTITNVIPDAR;MELHFEFM;SSVMVIGLSEVFMQQTSVTFR;TIIGDLGSLQGPEGGEWLAFFYPAHTVFEK;VGETPDLSSFSQEEISK | 0.6694 | 48.4886 | 33 | NA |
| 3H-55 | AYELANER;AYELANERGPFSVEVYLNPGTSNTYQYVATTR;FNNTDYDDLPWNYTSGK;FNNTDYDDLPWNYTSGKK;GPFSVEVYLNPGTSNTYQYVATTR;KVVTATGVVSGGER;LPQSFTR;NKFNNTDYDDLPWNYTSGK;SEIAQDIQVTMYNTNGGSNPLNNSNVTR;SNVDSSSYYQQPPQVVYNNGDLYGSR;TGYSGAELGASIDK;TGYSGAELGASIDKGIASLWGYLK;VVTATGVVSGGER;YVFLLR | 0.6933 | 55.6394 | 29 | NA |
| 3H-58 | AVSPLTNKPIDVHGR;HIVVSNGFQPVR;LIGTSPIR;LIGTSPIRK;MCAGTPNTR;NDQNVPTYYTSSRPFADR;NESINPITGKK;SIRPEQAAYR;SQLINIANNMNIAELR;SVSPYSGRPIK;TAANADTRPDLCATFSR;VAQNTTQSQLLNIVK;VAQNTTQSQLLNIVKFQIR;VLLSPIPDGATPMSR | 0.2643 | 55.0234 | 24 | cofactor of the virion S/T kinase encoded by thioredoxin |
| 3H-48 | ALLLSQLVSPPLIAK;AMHLLYIEGK;CLGQVVTNVR;CSSTAAVDAAVASSSTEALSQK;DINLLQVGLSAADTACISNVTLQAFTK;GSSISTAVSK;LQSWVTDHEAVQYGTKPTVPYVIPDNLLSQKPTDNTISR;LYGYMANK;SVASVSSPGNIASSSVGNK;SVASVSSPGNIASSSVGNKR;TAQSIMQNLMTQIK;THIFNYLDSK;TVNVNQDSQTIK | 0.4053 | 54.0362 | 22 | NA |
| 3H-151 | ALQYQTQLEGLLSR;CKPGEVMDDKSSTTEER;ETCALTTVTASLATISESLGK;ETCALTTVTASLATISESLGKDEALLK;ITEVGGGVAAIKSSLTSVVFTPLSEK;RVVVDPLDTK;SSLTSVVFTPLSEK;SSLTSVVFTPLSEKLDAIK;VVVDPLDTK;VVVVPVQDK;VVVVPVQDKITEVGGGVAAIK | 0.5320 | 49.6720 | 20 | Uyr/REP helicase |
| 3H-117 | CIPVVAYHAQDADAPTDTGLK;DDAIIIDDLPDVYITQPHR;DYAAVITAELILR;LEMFLSSDETR;MFAKPEPAIR;NLNALWDLWGADVIIER;NVNGFVPAVIFSDYEVYK;RLEMFLSSDETR;RPHLDEFLSYLFENFR;SRPTVFLDLDNTLICSLR | 0.7619 | 43.4405 | 19 | CDT phosphatase transcription factor |
| 3H-62 | AYVDLKDFVVTHENLFAR;DFVVTHENLFAR;KTQTPVNILADIVSLK;LDNNFVDGLK;LFVPGIDLPQPYIGIDTTK;LFVPGIDLPQPYIGIDTTKR;LITDPYQLLQISER;RLFVPGIDLPQPYIGIDTTK;TQTPVNILADIVSLK;YTTSTVPNETDR | 0.3116 | 44.1149 | 19 | myristylated membrane protein-like protein |
| 3H-19 | ATVGALAGDVVKR;DIYIENK;ESTTNIAEDIEREPAYLK;GDDVRPELSTFVLSYTR;IFSMLDNEIHLTIDSSDHQHVLQIYNR;ISPMYSFHSR;LLYWLEDNR;LLYWLEDNRFDLLTVDGNDAR;QTLGLVIK;RNEQPQNSSSFDK;SHVDDNEDLAPSKR;TPSPTAVEVNTLAR;WSYNSLTDGGAFAITGILAR | 0.2817 | 52.3957 | 17 | NA |
| 3H-43 | DFNVASYESDPLLK;DLYNLQILR;DQSLELLSHTHR;FGLASVNDENSYK;FVEFGTLVRPAGWGSLSELGK;KDFNVASYESDPLLK;LSLGYQTVVQFYR;LSYDSVVDWER;MFNVDQAIEFFENR;RFGLASVNDENSYK;VCHLNAVLDPAFTIDTGNVR;YNSSMDEYRDQSLELLSHTHR | 0.5923 | 51.7893 | 16 | NA |
| 3H-110 | ATDINPYETK;GWVPVNGSDAFEFSCK;HLNPQWATDAPILK;IGFISQILPR;KATDINPYETK;LGNVSGIITK;LTFNGTAK;NVDDDTIELR;TMYAPLSK;VVLAPEK;VVLAPEKVYEFISR;VYEFISR | 0.4742 | 44.5052 | 15 | NA |
| 3H-84 | ASIQSGYNK;LLSPPVDPHVFIDTTAASIDK;NMVINEPQYQQFLR;NSEEDSSPAQADQSESVGSSPPTK;SIDTNEGNDDDSTAIALR;SPHLDMAFR;TGTEPKPEHSDDGKNSEEDSSPAQADQSESVGSSPPTK | 0.4719 | 31.6094 | 13 | ABC-type transport system permease |
| 3H-3 | DYFGWDKLEAAYNAK;FNHGTIEHPNCPGDVLPDDCSK;IIQIAPVDLIK;LEAAYNAK;TLLKPTVHLNR;YANVNGFEGEFNSQYSVQSYNK;YGTIER | 0.4265 | 26.1909 | 12 | NA |
| 3H-37 | AGLLELVIK;EFFTLNEDNFTELIK;IGYTCNLNDR;ILEYSNAPNAIAK;LHSQVTNLHPLSK;LSELNVASAYDFRPVFVVPTK;LSELNVASAYDFRPVFVVPTKK;QLEAVLHTK;QTLEEQLPR;YWLVNELFPSLK | 0.3889 | 36.8806 | 11 | bro4 |
| 3H-66 | DIIMGHISPNR;DTFAVSPAR;IFLATNTNSHVR;KVQVDYSPNYDLTVVTVNDHVSTTK;TVISGDTYIK;VQVDYSPNYDLTVVTVNDHVSTTK;VTVHEVNADLYLCKPR;YTDIEYK | 0.2432 | 28.6732 | 11 | NA |
| 3H-107 | FFPAETHVEVK;IDHVPSAVVK;LQLSDWNDNVTTIK;LQLSDWNDNVTTIKK;NYVLNEPPAR;RIDHVPSAVVK;TNTPLEGVPEFVLFHYGNAVK | 0.4172 | 28.4758 | 10 | thioredoxin-like protein |
| 3H-137 | AMSNGLGDVILSAK;IFDAFKR;KIFDAFK;KVSGDVIDK;SEDEEVSAEIVTAK;TGVDLSVNVGQTYK;VSTTVDPMMPAQVK;YSVAVAK | 0.5000 | 27.9168 | 10 | hemolysin-like protein |
| 3H-63 | EYVFGVR;GSVSDLEFK;GTCAMTLLQQLK;HYTGIIPETMNVAK;KNVGLFIPK;LWNASVDSFCYR;SGDEIDGYIIMKPK;TAFSIVNK | 0.2824 | 28.8242 | 10 | NA |
| 3H-135 | ALNMILK;KRPIQPTAYNLFYK;RPIQPTAYNLFYK;SNSNNTMTALDGVHQINSLVTSVFSDSEHINEWLSLSK | 0.4720 | 17.0211 | 9 | yabby-like transcription factor |
| 3H-150 | LQVIFDDNDEKR;NIDLIDVPYTIPIDEIPNFIDK;QHYEYVPR;SADDALLDPTTSPSPVFAR;VRPDWLINPNTK | 0.3133 | 22.5782 | 9 | NA |
| 3H-171 | DFLTLFDVDELR;IASHQLACLNYTLPVR;LGFQEFVYR;LKDFLTLFDVDELR;LMHYGDAPNDVTPSK;MFVVQTNR;VIDTFSK | 0.2197 | 29.5172 | 9 | caspase-like protein |
| 3H-71 | LGKPIVVIAPVR;LGKPIVVIAPVRR;NLFTNIR;RLGKPIVVIAPVR;VQFPHVVHNPELNVIYWR | 0.1875 | 20.9282 | 9 | sulfhydry1 oxidase Erv1 like protein |
| 3H-109 | CVDAVPFK;EVHVSVTTLMK;IAQYPINTK;IDYKETQR;NSWGEHWGDGGYFK;QAVSSSGCGGGSTYR;VGGAYFVTVR;YLDSLIPTPR | 0.2068 | 32.3383 | 8 | cathepsin B |
| 3H-139 | AHIAPALLNHIESNLLK;ATLDEVSNDPDDIDVER;ATLDEVSNDPDDIDVERR;FVFSARPTVDSSLASGGVR | 0.1949 | 18.0625 | 8 | putative S1/P1 nuclease |
| 3H-69 | AAILLGASLVLNK;DYDTGKDESHYMLYTK;LVYKDYDTGK | 0.4231 | 11.7746 | 8 | NA |
| 3H-77 | MAFVHLFHIDGILECAER;QWATDELK;VAALKQWATDELK;VHGWWWR;VQKVHGWWWR | 0.4940 | 20.4954 | 8 | NA |
| 3H-115 | KTPQLQEDLASYTK;LESVENVINAFNLR;LITATEEELK;TPQLQEDLASYTK;VAGIENLVLQTK;VCSIPTNEDNPDK | 0.1050 | 23.5285 | 7 | NA |
| 3H-126 | ALNTHDAGAYK;APVTATPVPVKPLYTAR;ELAGVVGVVCNAVR;EYFLLSVAQIFR;NDEALIR;STAAAITETVAGILK | 0.0722 | 22.0761 | 7 | ATPase involved in DNA replication |
| 3H-182 | APQQIATVK;EGTMTVANHMGSATR;HTGGLIITAPLVHPAVGTTR;SLPDPLDIYVR;SSIIHNIQK;TQHHNEEVETVDQSILAHVR | 0.2127 | 23.7886 | 7 | NA |
| 3H-46 | HSDDIDTTIDALVK;KLDQEVLIQEMR;YMEVSSNDTLLYDNVR | 0.1634 | 13.5469 | 7 | NA |
| 3H-118 | EDVIHDR;LDSFVGPTGNFHGMNVTMK;LNGQLHTLVIPK;QVGKPPICLGSDDVLR;SIVAAFATADLPTALYHSETATYR | 0.4643 | 21.2908 | 6 | NA |
| 3H-79 | NEPVSYLAVGNAQFIDR;RNEPVSYLAVGNAQFIDR;SPNCALVFTR;VPVSNTVEFVIVILK | 0.3440 | 16.7751 | 6 | NA |
| 3H-88 | ASFDTVPWVDGR;FVSNTQQLVENSLIYGGLR;ITPVTFMTTVER;NCHTNAASALEAR;QISHVVYADWLTER | 0.2692 | 22.5782 | 6 | putative S1/P1 nuclease |
| 3H-28 | GIDDGDAIYTITK;ITALYHNVK;LTEWFPNNR;TAYDQADVIAYIDGDKEK | 0.1384 | 18.0625 | 5 | NA |
| 3H-44 | DNSEASSQNTTVWDR;DNSEASSQNTTVWDRR | 0.1584 | 9.0312 | 5 | NA |
| 3H-116 | DLPLTLR;ETFYQVFSQVTSVDHR;LWEFADDFPNKETFYQVFSQVTSVDHR | 0.0955 | 8.1122 | 4 | ATPase involved in DNA metabolism |
| 3H-128 | DVISDQPR;HICNDIVNVFNEK;YADIPVWEQGVEDEPTSETNDTLNR | 0.1716 | 12.0915 | 4 | NA |
| 3H-157 | EQITQWLR;GINDLSDAELAYK;NPPLELWSR;VLIDTEQKR | 0.2254 | 18.0625 | 4 | tyrosyl-DNA phosphodiesterase |
| 3H-4 | DFNEFIVWVPTK;LSHDYAYSLR;NSDFAWK;SSTVVSTVSSATWVK | 0.1864 | 15.0593 | 4 | NA |
| 3H-72 | ELQPPYSNYFEESLKPEAFVSNIHYK;LSSLLTSIVSSHSGMIHDK;QEYEDDDYNFK;VTTSSILR | 0.4129 | 18.0625 | 4 | phenylalanyl-tRNA synthetase α subunit |
| 3H-8 | LNAQDVAVLGTSNPILR;QLQNMYIVVDEFLK;TLSVYESAFPMR;VYIPVPPITR | 0.1218 | 15.9703 | 4 | NA |

Table S14 Identified HvAV-3h coded proteins from *Spodoptera litura* produced virions (Sl-1)

| Protein ID | Identified peptide sequence | Sequence coverage | Unique Peptide | Function annotation |
| --- | --- | --- | --- | --- |
| 3H-57 | QHPSFANKPNNTDVQQSDLTIAEDADTGTSVTK;MTAAHLADDR;FNRQQLEWILVNSNPAIALQDASVR;QLNALNLR;TNDNLEIK;RNNVQLQTALQR;RLLVSEDK;QHPSFANKPNNTDVQQSDLTIAEDADTGTSVTK;LQLDYMTLQQR;NSGVGNAINDRLNK;QSLLETSAQLNDVR;EKAQLQSDLDVLR;DEAIWAIK;VNRNSGVGNAINDR;AKLDLVTINSVANK;SEYELSASRR;LSNASVGVQLDR;MEFEAVNDALSK;QHPSFANKPNNTDVQQSDLTIAEDADTGTSVTK;RPIRPMADNFAEGYVYERPMGGGAPR;ELNTVR;QQLEWILVNSNPAIALQDASVR;NAEITNLQDR;KTNDNLEIK;AQLDVADLER;SNANLSPQSIR;NVNDTTLAELAQSR;NNVQLQTALQR;TNDNLEIK;ITNEFFDHATLPLLR;SRSDVVVLR;KTNDNLEIK;NSGVGNAINDR;TKEMYAVLEDELK;TNDNLEIK;QLNALNLR;QHPSFANKPNNTDVQQSDLTIAEDADTGTSVTKLLISSETLGR;TYQQEMTQLKR;QIDSLR;QSLLETSAQLNDVR;MLELQNLMK;LNNELETSITEER;YESLMTK;YNLIKTNVQQQR;IQAFVNTEQNILAAGTR;QMGNVVQEIAQR;LLSLDGVR;SETNALVVEVADLK;SETNALVVEVADLKSQLNR;ATIDALTANLNSSNSK;NSGVGNAINDR;LNDIAELQSQLTK;GGMGVLDDIYNR;NELAATVNSLQMQATK;QHPSFANKPNNTDVQQSDLTIAEDADTGTSVTK;LTNECQAVSSR;AQTDLEK;QLNALNLR;DSSFPIVDADNTEQLVVR;LLISSETLGR;ISNSVKNDVESK;KQMGNVVQEIAQR;LNNELETSITEER;VITGDETVMTYNR;NSGVGNAINDR;AQLQSDLDVLR;LNKELNTVR;LQLDYMTLQQR;QMGNVVQEIAQR;TYQQEMTQLK;MEFEAVNDALSK;QQLEWILVNSNPAIALQDASVR;ITNEFFDHATLPLLR;LTNECQAVSSR;QQLEWILVNSNPAIALQDASVR;VITGDETVMTYNR;ISNSVKNDVESK;RLNNELETSITEER;QMKHENLYLASTTPSR;QHPSFANKPNNTDVQQSDLTIAEDADTGTSVTK;SDVVVLR;NIDGAEGFTR;NIDELR;CQAELQQR;SETNALVVEVADLK;NNVQLQTALQR;EHDVLER;ISELLTQLQESNNQR;ELACNVIPEKCGDITQMNR;AEMQINTQR;LQLDYMTLQQR;QLAQNER;MLELQNLMK;AEMQINTQR;EKQHAEFLR;HIAGVITKNIDGAEGFTR;IQAFVNTEQNILAAGTR;LLKEHDVLER;QQLEWILVNSNPAIALQDASVR;QHPSFANKPNNTDVQQSDLTIAEDADTGTSVTK;YEALMSEQSSK;RPSVTDDEDDRIVGTR;TQLLTGLR;QMGNVVQEIAQR;MTAAHLADDRLSNASVGVQLDR;GRISELLTQLQESNNQR;TYQQEMTQLK;NELAATVNSLQMQATK;SEYELSASR;SLSYSLTDDEDDAAQIR;MTAAHLADDRLSNASVGVQLDR;ALLEVTDNVVTDRVPVR;YDNYNRNEHELR;MEFEAVNDALSK;QSLLETSAQLNDVR;HVGNAQSDGMYTENVALR;ATIDALTANLNSSNSK;IVENMYKR;QHPSFANKPNNTDVQQSDLTIAEDADTGTSVTK;RNNVQLQTALQR;GELNAMTR;EMYAVLEDELK;ISELLTQLQESNNQR;QLNALNLR;QHPSFANKPNNTDVQQSDLTIAEDADTGTSVTK;MLELQNLMK;ALEMKEMWER;ISELLTQLQESNNQR;NAEITNLQDR;HVGNAQSDGMYTENVALRNEIGQLR;IDHDRR;SQALYAIK;QLNALNLR;QHPSFANKPNNTDVQQSDLTIAEDADTGTSVTK;ALLEVTDNVVTDR;ATIDALTANLNSSNSK;QQLEWILVNSNPAIALQDASVR;LDLVTINSVANK;SLAELRNVNDTTLAELAQSR;VAYANLEGDMQK;NELAATVNSLQMQATK;QMGNVVQEIAQR;MEFEAVNDALSK;MLELQNLMK;SNANLSPQSIR;KQMGNVVQEIAQR;LDLVTINSVANKVNR;NVNDTTLAELAQSR;LIDSVVKITR;EMYAVLEDELK;NIDGAEGFTR;IESEAALVESLQR;YEALMSEQSSK;LIDSVVK;QHAEFLR;LLVSEDK;LDGSDVTLVVDDENLK;HIAGVITK;RLNNELETSITEER;QMGNVVQEIAQR;QHPSFANKPNNTDVQQSDLTIAEDADTGTSVTK;NSGVGNAINDR;DVNLAKSETNALVVEVADLK;TKEMYAVLEDELK;STMLER;AKLDLVTINSVANK;NSGVGNAINDR;LDLVTINSVANK;NISAIRPR;LDLVTINSVANK;VAYANLEGDMQK;HVGNAQSDGMYTENVALR;MLELQNLMKVER;TQLLTGLRLYR;IESEAALVESLQR;LLSLDGVRR;RLNNELETSITEER | 1.7107 | 177 | chromosome segregation SMC like protein |
| 3H-152 | RFNLLHDTINSLFEAGVVDR;YETDFTSDESPFSIVTSIDK;ERNHIALFMVR;GTLMSELFTVDER;FIYEYNNYTVR;EISFDMFDALYGNGDDADVR;VDDAELER;ASYMNLLIVEQDR;HPPTADTALSAMSR;NAAADACDNDSLAPEVR;SLSDAVQMVK;FVEHCIIDAYTSNR;CPGNLEAMLAVQR;FIYEYNNYTVR;MSAYETSEIRK;RELLNPEHSAK;MSAYETSEIR;YGEWLK;LLQYHFDTNVFLYVR;HDDTNTVIVQYSVDKHYLSR;FSVTVESTRR;SQTFYNTQR;TVSLFRPPLSLK;IYDAPNTNVLYGIANDITSHMIQYK;EKVVQGATAVR;SGVHSAVDMYMYIR;RVDDAELER;MSAYETSEIRK;STDTNSATFYPK;ASYMNLLIVEQDR;GIYSPGMTYDIAHVYPAPGVGYTISVLSNNAPTVDVPR;EISFDMFDALYGNGDDADVR;FSVTVESTR;DVAVANSDKTAPR;YITELLR;DMVSPSNADGPR;HPPTADTALSAMSR;TSPTLILHVWR;GAVQVGPNAAIEAVMR;QLSAHWR;TYQALLPQPMR;RGIYSPGMTYDIAHVYPAPGVGYTISVLSNNAPTVDVPR;ATCIFIDETHLMR;AVHCLQSSTADPR;SLVIGAEQLER;NHIALFMVR;SWVDRDPESNTK;AIYYVSQGK;FIYEYNNYTVR;VLTDSVDTAR;MVWFPEFVSPVSDDAPR;EHITFNPGR;TYQALLPQPMR;SEELDILK;VVQGATAVR;KGTLMSELFTVDER;LIGMDGVQLETIR;VLLHLTAYTILK;FTISSPR;RLVYNASLLLR;VLLHLTAYTILKK;DVAVANSDK;LIGMDGVQLETIR;FYTEHLHTDELLNINVR;GTLMSELFTVDER;RGIYSPGMTYDIAHVYPAPGVGYTISVLSNNAPTVDVPR;SLSDAVQMVK;LVYNASLLLR;STDTNSATFYPK;MSAYETSEIR;SQTIDGVGR;ELLNPEHSAK;VLPLTAFGK;LVYNASLLLR;DNTSYFDPLR;SVDLVPDGK;VLTDSVDTARR;DMVSPSNADGPR;FNLLHDTINSLFEAGVVDR;HDDTNTVIVQYSVDK;SVDLVPDGKEHITFNPGR;LVYNASLLLRR | 0.8584 | 82 | NA |
| 3H-53 | ENVPDTGAGTGFMNMLTDGDFATPPEITK;GSINPSNFNSSISVVIEPSEALR;GSINPSNFNSSISVVIEPSEALR;EIRPCAAFSK;DSGVALPIGMMVNNR;DSGVALPIGMMVNNR;GQYMYSFALDLK;NITHSEYLDYYEVGLPK;IDNGGDFN;GTIDSYIYGPDEQVTTYFVR;TSCSPHYILMEKPTDIAATEILKPTEAGSTLSYDIEHSSGILK;VPMLPAEHFVFTEPYR;VTVEFVFR;YRVPMLPAEHFVFTEPYR;SDVNKR;NRGQYMYSFALDLK;TVDPKGSINPSNFNSSISVVIEPSEALR;ALFFGVR;MPVLLSTGNGSNK;GQYMYSFALDLK;SYAAGTESSYFPKLAHK;SIGVSALSKIGIK;ALFFGVRNITHSEYLDYYEVGLPK;KIDNGGDFN;KIDNGGDFN;ENVPDTGAGTGFMNMLTDGDFATPPEITK;LSDLLIK;RGTIDSYIYGPDEQVTTYFVR;GSINPSNFNSSISVVIEPSEALR;MPVLLSTGNGSNK;SYAAGTESSYFPK;NITHSEYLDYYEVGLPKSNNGVR;KLSDLLIK;MPVLLSTGNGSNK;GTIDSYIYGPDEQVTTYFVR;GSINPSNFNSSISVVIEPSEALR;ENVPDTGAGTGFMNMLTDGDFATPPEITK;GSINPSNFNSSISVVIEPSEALR;DSGVALPIGMMVNNR;NITHSEYLDYYEVGLPK;MPVLLSTGNGSNK;SDVNKR;TLVLPIPLYFSR;VTVEFVFRK;VPMLPAEHFVFTEPYR;NITHSEYLDYYEVGLPKSNNGVR;YRVPMLPAEHFVFTEPYR;TSCSPHYILMEKPTDIAATEILKPTEAGSTLSYDIEHSSGILK;SIGVSALSK;ENVPDTGAGTGFMNMLTDGDFATPPEITK | 1.9033 | 50 | major capsid protein |
| 3H-27 | ALINEDGHSVVTQR;YAIDVNILPAISDTIQK;SPPTLTIDDDSILYPLQYIPR;HIHSVGDPSYNDEVTR;KADNVWVYTEDLQAIK;SLVVLTLQHELRK;HSNVYSDALESFRR;SLVLHFGANPAYTIMNTFASPAESPR;SLVVLTLQHELR;RSLVLHFGANPAYTIMNTFASPAESPR;FNIDNVNYASVAHAIALISVVYHMK;HIHSVGDPSYNDEVTR;ADNVWVYTEDLQAIK;ALHGDSR;IVTSQLLYK;RPDAEECFK;DVLAKTGYANLK;FTDEIIK;ARDDNSPSNDSVAMK;GVVWSEQSQQLVAAAATTK;TLFGDVVQK;RYAIDVNILPAISDTIQK;YTDFDQLWSK;VDEFISFGR;TGYANLK;SLVLHFGANPAYTIMNTFASPAESPR;FSVLNSPLSR;DDNSPSNDSVAMK;RYAIDVNILPAISDTIQK;DSANSLLK;FTDEIIKK;HITYVVVGHALVSHR;VLTAALMHTDADTVNPTTPSVGYESDAVPR;CLLSITR;AMIESILK;YKHFDIR;HSNVYSDALESFR;FSVLNSPLSR;KVDEFISFGR;YVDMSQYR;NLCEIGVVSNVTDVDLLR;STEDDRPIQMQR;FNIDNVNYASVAHAIALISVVYHMK;RDPALYEVQLHTCR;ALINEDGHSVVTQR | 0.8406 | 45 | NA |
| 3H-50 | NALVALSNSSMNNEQNYTNR;ATHYDPIRDGYYMYALPNGR;NALVALSNSSMNNEQNYTNR;ATHYDPIRDGYYMYALPNGR;NVYKDPAR;ATHYDPIRDGYYMYALPNGR;NALVALSNSSMNNEQNYTNR;DGYYMYALPNGR;NALVALSNSSMNNEQNYTNR;DYFLSSKYK;NALVALSNSSMNNEQNYTNR;NALVALSNSSMNNEQNYTNR;NALVALSNSSMNNEQNYTNR;YDNFQCNK;YNNLSWLLSKNVYK;FLKPTFWFSR;ASCGTIPDAR;DGYYMYALPNGR;DYFLSSK;DGYYMYALPNGRVDR;DGYYMYALPNGR;ATHYDPIRDGYYMYALPNGR;LPSDSGVAVTTSPTVVDDLDLR;ATHYDPIR;YNNLSWLLSK;YNNLSWLLSK;YNNLSWLLSK;YNNLSWLLSK;DGYYMYALPNGR;NVYKDPAR;DGYYMYALPNGRVDR;ESYCKK | 2.3704 | 32 | NA |
| 3H-56 | LYPALMSPNTITNVIPDAR;LTNDEMNR;EEDGWDVATTEK;IISETVK;MELHFEFM;LYPALMSPNTITNVIPDAR;DMNGIAVSVDAK;MELHFEFM;VGETPDLSSFSQEEISK;IMNGAGAAGGAVVPK;DMNGIAVSVDAK;SSVMVIGLSEVFMQQTSVTFR;TIIGDLGSLQGPEGGEWLAFFYPAHTVFEK;LYPALMSPNTITNVIPDAR;LKEEDGWDVATTEK;MELHFEFM;IVKAHMFKPK;LQPAPLDIKGGRPMVR;TIIGDLGSLQGPEGGEWLAFFYPAHTVFEKMR;IMNGAGAAGGAVVPK;SSVMVIGLSEVFMQQTSVTFR;LTNDEMNR;SSVMVIGLSEVFMQQTSVTFR;LQPAPLDIKGGRPMVR;NGVHSTDR;SSVMVIGLSEVFMQQTSVTFR;IMNGAGAAGGAVVPK;DMNGIAVSVDAK;DMNGIAVSVDAK;LQPAPLDIK | 1.8388 | 30 | NA |
| 3H-55 | VVTATGVVSGGERYVFLLR;GPFSVEVYLNPGTSNTYQYVATTR;TGYSGAELGASIDK;SEIAQDIQVTMYNTNGGSNPLNNSNVTR;NKFNNTDYDDLPWNYTSGK;AYELANERGPFSVEVYLNPGTSNTYQYVATTR;LPQSFTR;NKFNNTDYDDLPWNYTSGK;FNNTDYDDLPWNYTSGK;SNVDSSSYYQQPPQVVYNNGDLYGSR;SNVDSSSYYQQPPQVVYNNGDLYGSR;FNNTDYDDLPWNYTSGK;IGQTPKAYELANER;SNVDSSSYYQQPPQVVYNNGDLYGSR;SEIAQDIQVTMYNTNGGSNPLNNSNVTR;YYYMSRPIGFGSSGAYDVPLLDTPLLR;NKFNNTDYDDLPWNYTSGK;AYELANER;AYELANERGPFSVEVYLNPGTSNTYQYVATTR;TGYSGAELGASIDKGIASLWGYLK;FNNTDYDDLPWNYTSGKK;GIASLWGYLK;YVFLLR;SEIAQDIQVTMYNTNGGSNPLNNSNVTR;VVTATGVVSGGER;AYELANER;SEIAQDIQVTMYNTNGGSNPLNNSNVTR;FNNTDYDDLPWNYTSGKK;KVVTATGVVSGGER;FNNTDYDDLPWNYTSGK | 2.6044 | 30 | NA |
| 3H-58 | VAQNTTQSQLLNIVK;VAQNTTQSQLLNIVK;LYNDLTK;TAANADTRPDLCATFSR;VAQNTTQSQLLNIVKFQIR;LIGTSPIR;YMINPNFVPTTTK;LIGTSPIRK;HIVVSNGFQPVR;NESINPITGKK;LGGLSPYR;DCNLSPPPTK;VLLSPIPDGATPMSR;LTAAQCETFIR;VLLSPIPDGATPMSR;YMINPNFVPTTTK;NDQNVPTYYTSSRPFADR;MVYSMCHGVPYR;VAQNTTQSQLLNIVK;AVSPLTNKPIDVHGR;LYNDLTK;SQLINIANNMNIAELR;MVYSMCHGVPYR;HIVVSNGFQPVR;QSMTAMR;NDQNVPTYYTSSRPFADR;KLIGTSPIR;SIRPEQAAYR | 0.5693 | 28 | cofactor of the virion S/T kinase encoded by thioredoxin |
| 3H-19 | KNTNLYGVTR;QIGNGEDANANVEINCGHR;ATVGALAGDVVKR;WSYNSLTDGGAFAITGILAR;VLPQHIR;ESTTNIAEDIEREPAYLK;QIGNGEDANANVEINCGHR;QTLGLVIK;QTLGLVIK;SETTDASNVSPR;IFSMLDNEIHLTIDSSDHQHVLQIYNR;STVTIEIVDDNHFEHNVECVLDTK;DIYIENK;NTNLYGVTR;GDDVRPELSTFVLSYTR;AELEGVADNIK;LLYWLEDNRFDLLTVDGNDAR;QIGNGEDANANVEINCGHR;SHVDDNEDLAPSK;STVTIEIVDDNHFEHNVECVLDTK;DDDEIR;SYADELGDR;VFDVPPVIHGEIFYGPFDVTGLHSYAGPPSLSIIVSNHPSNGSQSK;ISPMYSFHSR;NEQPQNSSSFDK;ISPMYSFHSR;TPSPTAVEVNTLAR;LLYWLEDNR | 0.6533 | 28 | NA |
| 3H-48 | TAQSIMQNLMTQIK;TAQSIMQNLMTQIK;LQSWVTDHEAVQYGTKPTVPYVIPDNLLSQKPTDNTISR;VDQEVYMK;SVASVSSPGNIASSSVGNKR;AMHLLYIEGK;TAQSIMQNLMTQIK;ATQNTVQTLR;ATQNTVQTLR;LYGYMANK;THIFNYLDSK;ALLLSQLVSPPLIAK;LQSWVTDHEAVQYGTKPTVPYVIPDNLLSQKPTDNTISR;ALLLSQLVSPPLIAK;SVASVSSPGNIASSSVGNKR;VDQEVYMK;GSSISTAVSK;THIFNYLDSK;NKAMHLLYIEGK;AMHLLYIEGK;LKNWSPK;SVASVSSPGNIASSSVGNK;CLGQVVTNVR;TVNVNQDSQTIK;CSSTAAVDAAVASSSTEALSQK;TVNVNQDSQTIK | 0.7778 | 25 | NA |
| 3H-43 | AVVFSDSR;RFGLASVNDENSYK;MFNVDQAIEFFENR;VCHLNAVLDPAFTIDTGNVR;FGLASVNDENSYK;TLSSMIR;YNSSMDEYR;FVEFGTLVRPAGWGSLSELGK;DQSLELLSHTHR;DFNVASYESDPLLK;YNSSMDEYRDQSLELLSHTHR;SSPQTVGLEWWKR;FGLASVNDENSYK;YNSSMDEYRDQSLELLSHTHR;YNSSMDEYR;FGLASVNDENSYK;RLSLGYQTVVQFYR;MFNVDQAIEFFENR;KDFNVASYESDPLLK;DLYNLQILR;SSPQTVGLEWWK;LSLGYQTVVQFYR;LSYDSVVDWER;LSLGYQTVVQFYR;IGTSAFTDDLHTVVDQGSGECMDTYIAR | 1.5064 | 25 | NA |
| 3H-140 | YTYMYVNKR;TLDRPTDNAIMQR;ALSHPDWLTIGSFIK;TLDRPTDNAIMQR;SGDEMTLSR;YTYMYVNKR;STGTTVGYTGTTSNAAMK;YTYMYVNK;YTYMYVNK;TATHLLGYK;YTYMYVNKR;TATHLLGYKNK;ETNYPIPYFQYNLVLSQR;STGTTVGYTGTTSNAAMK;ELFYHSEAGR;TLDRPTDNAIMQR;DVDGVASNGGGSVGTGLGGPLGGTSGVNTSNLR;DVDGVASNGGGSVGTGLGGPLGGTSGVNTSNLR;KQLCNAIK;EVAKELFYHSEAGR;QLCNAIK;SGDEMTLSR;STGTTVGYTGTTSNAAMK;NSDKDVTIPK | 1.5189 | 24 | NA |
| 3H-110 | VYEFISR;LTFNGTAK;GWVPVNGSDAFEFSCK;KNVDDDTIELR;NVDDDTIELR;FLTNEIKADGTAIR;ATDINPYETK;NVDDDTIELR;ATDINPYETK;TSNKPLIFECILDEPEK;LTFNGTAK;VVLAPEK;LGNVSGIITK;GWVPVNGSDAFEFSCK;LGNVSGIITK;VVLAPEKVYEFISR;IGFISQILPR;LSFDKK;TMYAPLSK;TMYAPLSK;KATDINPYETK;TYKHLNPQWATDAPILK;FLTNEIK;HLNPQWATDAPILK | 1.2160 | 24 | NA |
| 3H-117 | NLNALWDLWGADVIIER;DDAIIIDDLPDVYITQPHR;LDSAESEMAR;NVNGFVPAVIFSDYEVYK;DDAIIIDDLPDVYITQPHR;RPHLDEFLSYLFENFR;RLEMFLSSDETR;LEMFLSSDETR;DYAAVITAELILR;VGVWTAASKDYAAVITAELILR;NVNGFVPAVIFSDYEVYK;RLEMFLSSDETR;TAGEEVGGVK;CIPVVAYHAQDADAPTDTGLK;MFAKPEPAIR;LEMFLSSDETR;NVNGFVPAVIFSDYEVYK;LDSAESEMAR;SRPTVFLDLDNTLICSLR;VGVWTAASK | 1.5608 | 20 | CDT phosphatase transcription factor |
| 3H-62 | ATLEQVFVR;VCINVNAMTAGDK;YTTSTVPNETDRR;INTGSITDSVQCFNK;KTQTPVNILADIVSLK;YTTSTVPNETDRR;LDNNFVDGLK;SKAYVDLK;LDNNFVDGLK;TQTPVNILADIVSLK;AYVDLKDFVVTHENLFAR;RLFVPGIDLPQPYIGIDTTK;YTTSTVPNETDR;LFVPGIDLPQPYIGIDTTKR;DFVVTHENLFAR;LITDPYQLLQISER;LFVPGIDLPQPYIGIDTTK | 0.8116 | 17 | myristylated membrane protein-like protein |
| 3H-151 | SSLTSVVFTPLSEKLDAIK;SSLTSVVFTPLSEK;LVQIDNTVK;VVVDPLDTKVEK;ETCALTTVTASLATISESLGK;VEKALQYQTQLEGLLSR;VVVVPVQDKITEVGGGVAAIK;VVVDPLDTK;LVQIDNTVK;VVVVPVQDK;RVVVDPLDTK;ETCALTTVTASLATISESLGKDEALLK;LDAIKSDISGK;VAFGLTR;SSTTEER;ALQYQTQLEGLLSR;ITEVGGGVAAIK | 1.1232 | 17 | Uyr/REP helicase |
| 3H-109 | NSWGEHWGDGGYFK;LTFFKK;VGGAYFVTVR;EVHVSVTTLMK;ALIVKPMNQFR;VALIMSIDAVIR;ALIVKPMNQFR;YLDSLIPTPR;SISDDLIR;EVHVSVTTLMK;QAVSSSGCGGGSTYR;VALIMSIDAVIR;ILSLDAVSVPIDQK;CVDAVPFK;NSWGEHWGDGGYFK;IAQYPINTK;SQLHFETHESK | 0.4550 | 17 | cathepsin B |
| 3H-107 | FYSPSCGACEHVK;LQLSDWNDNVTTIK;ASLKTNTPLEGVPEFVLFHYGNAVK;GSEELVR;NYVLNEPPAR;LQLSDWNDNVTTIK;RIDHVPSAVVK;RGILSIR;YASDSLMYFECDLSIR;NYVLNEPPAR;LQLSDWNDNVTTIKK;FFPAETHVEVK;IDHVPSAVVK;TNTPLEGVPEFVLFHYGNAVK;TNTPLEGVPEFVLFHYGNAVK | 1.2577 | 15 | thioredoxin-like protein |
| 3H-137 | VSTTVDPMMPAQVK;AMSNGLGDVILSAK;AMSNGLGDVILSAK;TGVDLSVNVGQTYK;YSVAVAK;VSGDVIDK;IFDAFKR;NALLTLK;SEDEEVSAEIVTAK;AMSNGLGDVILSAK;DTMAMIFK;KIFDAFK;KVSGDVIDK | 0.8563 | 13 | hemolysin-like protein |
| 3H-126 | VFWALK;EYFLLSVAQIFR;IRDPVATER;FVNDFFTSLFPDPEVK;WTLEVINWR;APVTATPVPVKPLYTAR;IYIDNER;HLEGFASLLLK;ISGSDVSQGLR;SVLISNK;ALNTHDAGAYK;TDIDSTVIADK;FDFDIK | 0.1264 | 13 | ATPase involved in DNA replication |
| 3H-71 | LSESVAVIEAK;RLGKPIVVIAPVR;LGKPIVVIAPVRR;RNLFTNIR;SRNEVAWLLR;NLFTNIR;NLFTNIR;NEVAWLLR;YCADIFDIVQHAASL;LGKPIVVIAPVR;VQFPHVVHNPELNVIYWR;LSESVAVIEAKR | 0.6442 | 12 | sulfhydry1 oxidase Erv1 like protein |
| 3H-28 | NNLNYDQLYTALR;LTEWFPNNR;NVAEYQSR;GIDDGDAIYTITK;ITALYHNVK;NITLSPQYCSEYLTLK;QHLLDER;LDEHVPPVAAFIR;NNLNYDQLYTALR;IFMLVLLER;TAYDQADVIAYIDGDKEK;LFFDLR | 0.3785 | 12 | NA |
| 3H-66 | SALVNCYAK;IFLATNTNSHVR;TADGDYDDDER;VQVDYSPNYDLTVVTVNDHVSTTK;IINDKLK;IINDKLK;YTDIEYK;VTVHEVNADLYLCKPR;DTFAVSPAR;DIIMGHISPNR;TVISGDTYIK | 0.3324 | 11 | NA |
| 3H-59 | FACAVMR;LQSELTMVR;LQSELTMVR;TAAQQLASYR;ALDELMDFKK;ALDELMDFKK;HVSAEIELK;KDNEISQLHEELDYATETINDLNDQLR;TELDHIK;ALDELMDFK | 0.6221 | 10 | putative nucleic acid-binding protein |
| 3H-135 | ALNMILK;ALNMILKR;RPIQPTAYNLFYK;RPIQPTAYNLFYKDQVPVLTK;ALNMILK;ALNMILKR;KRPIQPTAYNLFYK;YLLDNYNYR;YLLDNYNYR;DQVPVLTK | 0.8320 | 10 | yabby-like transcription factor |
| 3H-84 | NMVINEPQYQQFLR;LLSPPVDPHVFIDTTAASIDK;NSEEDSSPAQADQSESVGSSPPTK;VVAKPQYDDR;TGTEPKPEHSDDGKNSEEDSSPAQADQSESVGSSPPTK;SPHLDMAFR;SPHLDMAFR;ASIQSGYNK | 0.5801 | 8 | ABC-type transport system permease |
| 3H-63 | FLILSASGPLK;YLWTFAPLLEQR;GSVSDLEFK;EYVFGVR;TAFSIVNK;LWNASVDSFCYR;SGDEIDGYIIMKPK;KNVGLFIPK | 0.2724 | 8 | NA |
| 3H-15 | DGTVVISEDDKHNEPYGIGK;YYVDDLVK;LKDPPHTDALK;SVADVCNPGGVYLGGR;FFNFKEDFTR;TIEALISLPR;VIVNNPR;KTIEALISLPR | 0.3131 | 8 | NA |
| 3H-79 | TNPELNDIER;NEPVSYLAVGNAQFIDR;FPFWFER;SGLNYIITFDSSATEK;RNEPVSYLAVGNAQFIDR;VPVSNTVEFVIVILK;SPNCALVFTR | 0.7440 | 7 |  |
| 3H-157 | NSTMNLPR;EQITQWLR;NPPLELWSR;VLIDTEQKR;GINDLSDAELAYK;TLLLHYR;DLPDTVPVSLDVR | 0.3873 | 7 | tyrosyl-DNA phosphodiesterase |
| 3H-83 | ESFGTYMGQR;ESFGTYMGQR;KPAQELIHEYR;MSPELQEAWR;AVTVTKGIAR;MSPELQEAWR | 0.4013 | 6 | glycosyltransferase |
| 3H-76 | ELYPEHVK;SLWSTDETLPR;SDLNALLR;EFPLVNPAR;FNEYDATLR;VYLMSVEEAK | 0.1361 | 6 | NA |
| 3H-72 | EKYESCIR;LSSLLTSIVSSHSGMIHDK;DQPMAGAVVETTGDWR;LSSLLTSIVSSHSGMIHDK;ELQPPYSNYFEESLKPEAFVSNIHYK;EFEYFK | 0.6065 | 6 | phenylalanyl-tRNA synthetase α subunit |
| 3H-69 | AAILLGASLVLNK;DESHYMLYTK;DYDTGKDESHYMLYTK;LVYKDYDTGK;DYDTGKDESHYMLYTK;DESHYMLYTK | 0.9615 | 6 | NA |
| 3H-26 | TSEIVDVVDATRK;TSEIVDVVDATR;QIVDPNATMK;SGATYAINNSYTIDALR;SGATYAINNSYTIDALR;ANSIIDNIER | 0.5896 | 6 | NA |
| 3H-182 | APQQIATVK;SLPDPLDIYVR;SITLQNTVTK;DVEFVAR;HTGGLIITAPLVHPAVGTTR;CDDGELTLHR | 0.1696 | 6 | NA |
| 3H-150 | QHYEYVPR;LAVEYNGR;VRPDWLINPNTK;LQVIFDDNDEKR;SADDALLDPTTSPSPVFAR;QHYEYVPR | 0.2876 | 6 | NA |
| 3H-46 | QLTQLR;KLDQEVLIQEMR;KLDQEVLIQEMR;YMEVSSNDTLLYDNVR;HSDDIDTTIDALVK | 0.2335 | 5 | NA |
| 3H-37 | QLEAVLHTK;LSELNVASAYDFRPVFVVPTK;AGLLELVIK;YWLVNELFPSLK;ILEYSNAPNAIAK | 0.2222 | 5 | bro4 |
| 3H-24 | YGNVDSQYYK;IAEIVVK;EGVLEITNSK;SANTTSVNAVNYR;IDYDYLVSPQLK | 0.0714 | 5 | NA |
| 3H-171 | LGFQEFVYR;LKDFLTLFDVDELR;DFLTLFDVDELR;MFVVQTNR;ILIVINQR | 0.1624 | 5 | caspase-like protein |
| 3H-82 | YDGEALTR;TVSAVGSNEELANTDDSSR;GGHGSVGEVHTSAVVR;ATITEVGNR | 0.1590 | 4 | NA |
| 3H-77 | VAALKQWATDELK;VHGWWWR;MAFVHLFHIDGILECAER;QWATDELK | 0.5542 | 4 | NA |
| 3H-139 | ATLDEVSNDPDDIDVERR;VAAYEIAR;ATLDEVSNDPDDIDVER;AHIAPALLNHIESNLLK | 0.2166 | 4 | putative S1/P1 nuclease |
| 3H-116 | ETFYQVFSQVTSVDHR;IQEDLFK;LWEFADDFPNK;IAFVGKPGTGK | 0.1292 | 4 | ATPase involved in DNA metabolism |
| 3H-81 | VITDNIVGTLNLLSR;HASWLFSYLQK;VTMFAPVYAYR | 0.1516 | 3 | NA |
| 3H-70 | YSGAGKPVSPNDFLMR;CEYVTKSTVCNESNDMLTLTESNPIDR;YSGAGKPVSPNDFLMR | 0.7024 | 3 | NA |
| 3H-29 | VFNDINVVK;ISPIFDGSVAILHDLSSGLVAAAISK;NSSVVMVK | 0.4388 | 3 | NA |
| 3H-21 | IASLLVHDIHSSLDDAVK;IGSIAPIELNPPPR;FKEVEDLVLR | 0.1842 | 3 | NA |
| 3H-128 | QTSSSQQRPNDLLYPLSYGAVDETVVLK;QIQYR;VVGVTYK | 0.1493 | 3 |  |
| 3H-118 | SIVAAFATADLPTALYHSETATYR;LNGQLHTLVIPK;RYAYTDSDGVYVK | 0.2917 | 3 | NA |
| 3H-113 | RLDIAFSGTDK;AAGFIVIEQDINTDNVTLVHEALR;EWFVQK | 0.1188 | 3 | lysophospholipid acetyltransferase |
| 3H-93 | SYVVASYVK;SAYEWYESR | 0.0577 | 2 | gamma_glutamyl hydrolase-like protein |
| 3H-90 | EFVDVHSSDTEADEDSHSSDSPSR;LELPEETFIK | 0.1250 | 2 | *iap*-like protein-5 |
| 3H-51 | VSTIVSDVK;VSELATSAK | 0.0870 | 2 | NA |
| 3H-44 | DNSEASSQNTTVWDR;DNSEASSQNTTVWDRR | 0.3069 | 2 | NA |
| 3H-3 | YGTIERIIQIAPVDLIK;TLLKPTVHLNR | 0.1373 | 2 | NA |
| 3H-168 | NHIQYQIDSISK;YQNTIAQLLR | 0.1287 | 2 | NA |
| 3H-166 | SLLGVLK;LFLISVVEHEK | 0.0994 | 2 | NA |
| 3H-146 | LATLGWYYYILK;SPYDTTQYFVK | 0.0852 | 2 | fatty acid elongase |
| 3H-138 | TLIELEK;VVLDRPQNVADVER | 0.1364 | 2 | NA |
| 3H-136 | GAAVAVSNINILSR;VFGSLAFNTTTVATILNYDR | 0.0345 | 2 | lipopolysaccharide modifying enzyme |
| 3H-124 | VGENSAWHNFETR;GYLLAAIK | 0.0320 | 2 | serine/threonine protein kinase |
| 3H-115 | LITATEEELK;VAGIENLVLQTK | 0.0367 | 2 | NA |
| 3H-1 | IEFLNIR;DLNELHSR | 0.0138 | 2 | DNA polymerase |
| 3H-88 | QISHVVYADWLTER | 0.0538 | 1 | putative S1/P1 nuclease |
| 3H-86 | IPETATSSCPSAAPK | 0.1389 | 1 | NA |
| 3H-80 | LSLYGLYR | 0.0178 | 1 | RNA polymerase subunit |
| 3H-67 | ITISDLNLR | 0.0226 | 1 | DNA repair exonuclease |
| 3H-64 | ESGNNTNISPLNR | 0.0304 | 1 | NA |
| 3H-61 | ASLDRGMFQATQR | 0.0113 | 1 | RNA polymerase subunit |
| 3H-54 | HSGESLTNYLLR | 0.0492 | 1 | NA |
| 3H-23 | YGTFAVDSAR | 0.0129 | 1 | NA |
| 3H-181 | HIPSADDPGADLFTNWFSINAHDR | 0.2162 | 1 | NA |
| 3H-174 | NDVSFGLYFPSEK | 0.0448 | 1 | NA |
| 3H-172 | EGQVYVITTSK | 0.0478 | 1 | bro23 |
| 3H-156 | DLFNFVSQTR | 0.0260 | 1 | NA |
| 3H-149 | WLDVQLK | 0.0507 | 1 | NA |
| 3H-131 | WLFEEVLPTGK | 0.0564 | 1 | bro16 |
| 3H-121 | VNYVYTNR | 0.0428 | 1 | NA |
| 3H-104 | ESYDALSVPSHWR | 0.0833 | 1 | bro12 |

Table S15 Identified HvAV-3h coded proteins from *Spodoptera litura* produced virions (Sl-2)

| Protein ID | Identified peptide sequence | Sequence coverage | Protein Q score | Unique spectra | Function annotation | |
| --- | --- | --- | --- | --- | --- | --- |
| 3H-57 | AEMQINTQR;AKLDLVTINSVANK;ALEMKEMWER;ALLEVTDNVVTDR;ALLEVTDNVVTDRVPVR;AQLDVADLER;AQLQSDLDVLR;ATIDALTANLNSSNSK;CGDITQMNR;CQAELQQR;DEAIWAIK;DEAIWAIKQHPSFANKPNNTDVQQSDLTIAEDADTGTSVTK;DSSFPIVDADNTEQLVVR;DVNLAKSETNALVVEVADLK;EHDVLER;EKAQLQSDLDVLR;EKQHAEFLR;ELACNVIPEKCGDITQMNR;ELNTVR;EMYAVLEDELK;FNRQQLEWILVNSNPAIALQDASVR;GELNAMTR;GGMGVLDDIYNR;GRISELLTQLQESNNQR;HIAGVITK;HIAGVITKNIDGAEGFTR;HVGNAQSDGMYTENVALR;IESEAALVESLQR;IQAFVNTEQNILAAGTR;ISELLTQLQESNNQR;ISNSVKNDVESK;ITNEFFDHATLPLLR;KQMGNVVQEIAQR;LDGSDVTLVVDDENLK;LDLVTINSVANK;LIDSVVK;LIDSVVKITR;LLISSETLGR;LLKEHDVLER;LLSLDGVR;LLSLDGVRR;LLVSEDK;LNDIAELQSQLTK;LNKELNTVR;LNNELETSITEER;LQDEIK;LQLDYMTLQQR;LSNASVGVQLDR;LTNECQAVSSR;MEFEAVNDALSK;MLELQNLMK;MTAAHLADDR;MTAAHLADDRLSNASVGVQLDR;NAEITNLQDR;NEIGQLRQSLLETSAQLNDVR;NELAATVNSLQMQATK;NIDELR;NIDGAEGFTR;NIDGAEGFTRDEAIWAIK;NISAIRPR;NNVQLQTALQR;NSGVGNAINDR;NSGVGNAINDRLNK;NVNDTTLAELAQSR;QHAEFLR;QHPSFANKPNNTDVQQSDLTIAEDADTGTSVTK;QHPSFANKPNNTDVQQSDLTIAEDADTGTSVTKLLISSETLGR;QIDSLR;QLAQNER;QLNALNLR;QMGNVVQEIAQR;QMKHENLYLASTTPSR;QQLEWILVNSNPAIALQDASVR;QSLLETSAQLNDVR;RLNNELETSITEER;RNNVQLQTALQR;RPIRPMADNFAEGYVYERPMGGGAPR;RPSVTDDEDDRIVGTR;RSLSYSLTDDEDDAAQIR;SDVVVLR;SETNALVVEVADLK;SETNALVVEVADLKSQLNR;SEYELSASR;SEYELSASRR;SLSYSLTDDEDDAAQIR;SNANLSPQSIR;SQALYAIK;SRSDVVVLR;SYIIELESR;TKEMYAVLEDELK;TNDNLEIK;TQLLTGLR;TQLLTGLRLYR;TYQQEMTQLK;TYQQEMTQLKR;VAYANLEGDMQK;VITGDETVMTYNR;VNRNSGVGNAINDR;VSTDDLTSYSR;YDNYNRNEHELR;YEALMSEQSSK;YESLMTK;YNLIKTNVQQQR;YQAAGTPGNGR | 0.6514 | 355.1825 | 335 | chromosome segregation SMC like protein | |
| 3H-53 | ALFFGVR;ALFFGVRNITHSEYLDYYEVGLPK;DSGVALPIGMMVNNR;EIRPCAAFSK;ENVPDTGAGTGFMNMLTDGDFATPPEITK;GQYMYSFALDLK;GSINPSNFNSSISVVIEPSEALR;GTIDSYIYGPDEQVTTYFVR;KIDNGGDFN;KLSDLLIK;LSDLLIK;MPVLLSTGNGSNK;NITHSEYLDYYEVGLPK;NITHSEYLDYYEVGLPKSNNGVR;NRGQYMYSFALDLK;RGTIDSYIYGPDEQVTTYFVR;SIGVSALSK;SIGVSALSKIGIK;SVDIGAKTLVLPIPLYFSR;SYAAGTESSYFPK;SYAAGTESSYFPKLAHK;TLVLPIPLYFSR;TSCSPHYILMEKPTDIAATEILKPTEAGSTLSYDIEHSSGILK;VPMLPAEHFVFTEPYR;VTVEFVFR;VTVEFVFRK;YRVPMLPAEHFVFTEPYR | 0.6374 | 97.8308 | 179 | major capsid protein | |
| 3H-50 | ASCGTIPDAR;ATHYDPIR;ATHYDPIRDGYYMYALPNGR;DGYYMYALPNGR;DGYYMYALPNGRVDR;DYFLSSK;FLKPTFWFSR;LPSDSGVAVTTSPTVVDDLDLR;NALVALSNSSMNNEQNYTNR;NVYKDPAR;YDNFQCNK;YKDDAR;YNNLSWLLSK;YNNLSWLLSKNVYK | 0.6561 | 45.9221 | 118 | NA | |
| 3H-152 | AIYYVSQGK;ASYMNLLIVEQDR;ATCIFIDETHLMR;AVHCLQSSTADPR;CPGNLEAMLAVQR;DGDHVDVR;DMVSPSNADGPR;DNTSYFDPLR;DVAVANSDK;DVAVANSDKTAPR;EHITFNPGR;EKVVQGATAVR;ELLNPEHSAK;ERNHIALFMVR;FIYEYNNYTVR;FNLLHDTINSLFEAGVVDR;FSVTVESTR;FVEHCIIDAYTSNR;FYTEHLHTDELLNINVR;GAVQVGPNAAIEAVMR;GIYSPGMTYDIAHVYPAPGVGYTISVLSNNAPTVDVPR;GLPDVYLYCNHDDAPYPGLAANR;GTLMSELFTVDER;HDDTNTVIVQYSVDK;HDDTNTVIVQYSVDKHYLSR;HPPTADTALSAMSR;IYDAPNTNVLYGIANDITSHMIQYK;KGTLMSELFTVDER;LIGMDGVQLETIR;LVYNASLLLR;LVYNASLLLRR;MSAYETSEIR;MSAYETSEIRK;MVWFPEFVSPVSDDAPR;NHIALFMVR;QLSAHWR;RELLNPEHSAK;RFNLLHDTINSLFEAGVVDR;RLVYNASLLLR;RVDDAELER;SEELDILK;SGVHSAVDMYMYIR;SIINVNNYAR;SLSDAVQMVK;SLVIGAEQLER;SQTFYNTQR;SQTIDGVGR;STDTNSATFYPK;SVDLVPDGK;SVDLVPDGKEHITFNPGR;TSPTLILHVWR;TVSLFRPPLSLK;TYQALLPQPMR;VDDAELER;VLLHLTAYTILK;VLLHLTAYTILKK;VLPLTAFGK;VLTDSVDTAR;VLTDSVDTARR;VVQGATAVR;YETDFTSDESPFSIVTSIDK;YGEWLK;YITELLR | 0.4980 | 203.7285 | 108 | NA | |
| 3H-13 | AAVWLATTTGDDSGYR;ALSSVALTCR;AVMNSFR;CVDEFR;CVTWSGEK;DIISPLILLDAER;DPYDTLNK;DVIHEHVVAHWNDAETEQIIGR;ENCAEYVCAR;ESPTEYFLR;EVMTVPSIVSALK;EWNATFVVIDEAHDLSVHGVDYPK;GANVAIILGSR;GLVLTPNR;HGQLDVDK;HGQLDVDKYIDCNGQYISISK;HLNDFEIDR;INGFLKLLTSR;KFAGLVSYLPSIVDVNEVDIVNVGER;KVLLLTATPMR;LEELGAVITDVTCGVQAK;LSTHDITVEQFNR;LTTYAEFNETQLLYSDDDDRDR;MIQYYGTSTNGDNDIR;NELNCYYR;NIDGALR;NITNDNTIPLDAPR;NNLSDLVPLHNLLMK;NQNVVVK;NTVIVVHDMSDVMSATYHYAGAGER;QRPTFVYPFVLDNVSLYGLDAYR;QRPTFVYPFVLDNVSLYGLDAYRDTLAK;RPMPHQILVANYTTPR;SDRDVALLR;SFIAQLDR;SFLCLTR;SKLEELGAVITDVTCGVQAK;SPIDGIIVIHGVGTGK;SSIEAQLNNDMLAK;SVINAAER;TFFFNTITAFANTVSR;TLTAILAMINNITAGHDQGMK;TSDVVLHK;TVDILMYATSEAK;VIAEGITLK;VLLLTATPMR;VTKSFIAQLDR;YALPLYPIAESTDESWSYEHLDK;YAWIAK;YIDCNGQYISISK;YQSHAYTIADWGSDAKPNVFVYR;YYGVIHPSTDDFCIK | 0.6706 | 171.7592 | 90 | DEAD-like helicase | |
| 3H-27 | ADNVWVYTEDLQAIK;ALINEDGHSVVTQR;AMIESILK;ARDDNSPSNDSVAMK;CLLSITR;DDNSPSNDSVAMK;DPALYEVQLHTCR;DSANSLLK;DVLAKTGYANLK;FNIDNVNYASVAHAIALISVVYHMK;FSVLNSPLSR;FTDEIIKK;GVVWSEQSQQLVAAAATTK;HIHSVGDPSYNDEVTR;HITYVVVGHALVSHR;HLTEYCR;HSNVYSDALESFR;HSNVYSDALESFRR;IVTSQLLYK;KADNVWVYTEDLQAIK;KCLLSITR;KSCGSSDDMNMQHVYDQVK;KVDEFISFGR;LYSTANELLADVLYGDGVDLGSTVSETTNR;NLCEIGVVSNVTDVDLLR;RDPALYEVQLHTCR;RPDAEECFK;RYAIDVNILPAISDTIQK;SLVLHFGANPAYTIMNTFASPAESPR;SLVVLTLQHELR;SLVVLTLQHELRK;SPPTLTIDDDSILYPLQYIPR;STEDDRPIQMQR;TDYELR;TGYANLK;TLFGDVVQK;VLTAALMHTDADTVNPTTPSVGYESDAVPR;VNPWMFAELAMLMR;VSDPQTTVR;YAIDVNILPAISDTIQK;YKHFDIR;YTDFDQLWSK;YVDMSQYR;YVPINR | 0.6469 | 144.3426 | 82 | NA | |
| 3H-55 | AYELANER;AYELANERGPFSVEVYLNPGTSNTYQYVATTR;DSYRLPQSFTR;FNNTDYDDLPWNYTSGK;FNNTDYDDLPWNYTSGKK;GIASLWGYLK;GPFSVEVYLNPGTSNTYQYVATTR;IGQTPKAYELANER;KVVTATGVVSGGER;LPQSFTR;NKFNNTDYDDLPWNYTSGK;SEIAQDIQVTMYNTNGGSNPLNNSNVTR;SNVDSSSYYQQPPQVVYNNGDLYGSR;TGYSGAELGASIDK;VVTATGVVSGGER;VVTATGVVSGGERYVFLLR;YVFLLR | 0.7378 | 62.7261 | 82 | NA | |
| 3H-58 | AVSPLTNKPIDVHGR;DCNLSPPPTK;HIVVSNGFQPVR;KLIGTSPIR;KNPFTGR;LGGLSPYR;LIGTSPIR;LIGTSPIRK;LTAAQCETFIR;LYNDLTK;MVYSMCHGVPYR;NDQNVPTYYTSSRPFADR;NESINPITGK;NESINPITGKK;RDCNLSPPPTK;SIRPEQAAYR;SVSPYSGRPIK;TAANADTRPDLCATFSR;VAQNTTQSQLLNIVK;VAQNTTQSQLLNIVKFQIR;VLLSPIPDGATPMSR;YMINPNFVPTTTK;YMINPNFVPTTTKK | 0.3377 | 71.1737 | 56 | cofactor of the virion S/T kinase encoded by thioredoxin |  |
| 3H-48 | ALLLSQLVSPPLIAK;AMHLLYIEGK;AMHLLYIEGKYLR;ATQNTVQTLR;CLGQVVTNVR;CSSTAAVDAAVASSSTEALSQK;GSSISTAVSK;LKNWSPK;LQSWVTDHEAVQYGTKPTVPYVIPDNLLSQKPTDNTISR;LYGYMANK;NDITAKNITSK;NEIIAKNDITAK;NKAMHLLYIEGK;SVASVSSPGNIASSSVGNK;SVASVSSPGNIASSSVGNKR;TAQSIMQNLMTQIK;THIFNYLDSK;TVNVNQDSQTIK;TVNVNQDSQTIKTHIFNYLDSK;VDQEVYMK | 0.4465 | 66.1662 | 54 | NA | |
| 3H-110 | ADGTAIR;ATDINPYETK;FLTNEIK;FLTNEIKADGTAIR;GWVPVNGSDAFEFSCK;HLNPQWATDAPILK;IGFISQILPR;KATDINPYETK;KNVDDDTIELR;LGNVSGIITK;LTFNGTAK;MAGLPLHAK;NVDDDTIELR;TMYAPLSK;TSNKPLIFECILDEPEK;TYKHLNPQWATDAPILK;TYVTLNR;VVLAPEK;VVLAPEKVYEFISR;VYEFISR | 0.7136 | 62.9170 | 53 | NA | |
| 3H-56 | DMNGIAVSVDAK;EEDGWDVATTEK;IISETVK;IMNGAGAAGGAVVPK;LKEEDGWDVATTEK;LQPAPLDIK;LQPAPLDIKGGRPMVR;LYPALMSPNTITNVIPDAR;LYPALMSPNTITNVIPDARNGVHSTDR;MELHFEFM;NGVHSTDR;SSVMVIGLSEVFMQQTSVTFR;TIIGDLGSLQGPEGGEWLAFFYPAHTVFEK;VGETPDLSSFSQEEISK | 0.6901 | 49.8890 | 50 | NA | |
| 3H-19 | AELEGVADNIK;ATTLANDR;ATVGALAGDVVK;ATVGALAGDVVKR;CLACQEVVTSTYSYR;DIYIENKAR;ESTTNIAEDIER;ESTTNIAEDIEREPAYLK;FDLLTVDGNDAR;GDDVRPELSTFVLSYTR;IFSMLDNEIHLTIDSSDHQHVLQIYNR;ILITTYDYSGVQQR;ISPMYSFHSR;KNTNLYGVTR;LLYWLEDNR;LLYWLEDNRFDLLTVDGNDAR;LVDCSTTR;LVNVTR;MRPTTADVDNAQPTITVDDLK;NEQPQNSSSFDK;NTNLYGVTR;QIGNGEDANANVEINCGHR;QTLGLVIK;RNEQPQNSSSFDK;SETTDASNVSPR;STVTIEIVDDNHFEHNVECVLDTK;SYADELGDR;TPSPTAVEVNTLAR;VFDVPPVIHGEIFYGPFDVTGLHSYAGPPSLSIIVSNHPSNGSQSK;VLPQHIR;WSYNSLTDGGAFAITGILAR | 0.5882 | 104.7673 | 49 | NA | |
| 3H-140 | ALSHPDWLTIGSFIK;DVDGVASNGGGSVGTGLGGPLGGTSGVNTSNLR;ELFYHSEAGR;ENFVNLVELWCK;ETNYPIPYFQYNLVLSQR;EVAKELFYHSEAGR;GSVSKSGDEMTLSR;KQLCNAIK;NSDKDVTIPK;QLCNAIK;SGDEMTLSR;STGTTVGYTGTTSNAAMK;TATHLLGYK;TATHLLGYKNK;TLDRPTDNAIMQR;YTYMYVNK;YTYMYVNKR | 0.8255 | 56.2991 | 47 | NA | |
| 3H-43 | AVVFSDSR;DLYNLQILR;DQSLELLSHTHR;FGLASVNDENSYK;FVEFGTLVRPAGWGSLSELGK;KDFNVASYESDPLLK;LSLGYQTVVQFYR;LSYDSVVDWER;MFNVDQAIEFFENR;RFGLASVNDENSYK;RLSLGYQTVVQFYR;SSPQTVGLEWWK;SSPQTVGLEWWKR;TLSSMIR;VCHLNAVLDPAFTIDTGNVR;YNSSMDEYR;YNSSMDEYRDQSLELLSHTHR | 0.7124 | 60.4189 | 37 | NA | |
| 3H-107 | ASLKTNTPLEGVPEFVLFHYGNAVK;FFPAETHVEVK;FYSPSCGACEHVK;IDHVPSAVVK;LQLSDWNDNVTTIK;LQLSDWNDNVTTIKK;NYVLNEPPAR;RGILSIR;RIDHVPSAVVK;SPYANAAR;TLDRNYVLNEPPAR;TNTPLEGVPEFVLFHYGNAVK;TNTPLEGVPEFVLFHYGNAVKTLDR;YASDSLMYFECDLSIR | 0.7362 | 44.8125 | 35 | thioredoxin-like protein | |
| 3H-151 | ALQYQTQLEGLLSR;ALQYQTQLEGLLSRVVVVPVQDK;ETCALTTVTASLATISESLGK;ETCALTTVTASLATISESLGKDEALLK;ITEVGGGVAAIKSSLTSVVFTPLSEK;LDAIKSDISGK;LVQIDNTVK;RVVVDPLDTK;SSLTSVVFTPLSEK;SSLTSVVFTPLSEKLDAIK;SSTTEER;VAFGLTR;VEKALQYQTQLEGLLSR;VVVDPLDTKVEK;VVVVPVQDK;VVVVPVQDKITEVGGGVAAIK | 0.6059 | 56.3890 | 34 | Uyr/REP helicase | |
| 3H-62 | ATLEQVFVR;AYVDLK;AYVDLKDFVVTHENLFAR;DFVVTHENLFAR;DGTYAKYDTR;INTGSITDSVQCFNK;KTQTPVNILADIVSLK;LDNNFVDGLK;LFVPGIDLPQPYIGIDTTK;LFVPGIDLPQPYIGIDTTKR;LITDPYQLLQISER;RLFVPGIDLPQPYIGIDTTK;SKAYVDLK;TQTPVNILADIVSLKYTTSTVPNETDR;VCINVNAMTAGDK;YTTSTVPNETDR;YTTSTVPNETDRR | 0.4795 | 57.4874 | 32 | myristylated membrane protein-like protein | |
| 3H-117 | CIPVVAYHAQDADAPTDTGLK;DDAIIIDDLPDVYITQPHR;DYAAVITAELILR;LDSAESEMAR;LEMFLSSDETR;MFAKPEPAIR;NLNALWDLWGADVIIER;NVNGFVPAVIFSDYEVYK;RLEMFLSSDETR;RPHLDEFLSYLFENFR;SRPTVFLDLDNTLICSLR;TAGEEVGGVK;VGVWTAASK | 0.9153 | 46.2307 | 31 | CDT phosphatase transcription factor |  |
| 3H-3 | DYFGWDK;DYFGWDKLEAAYNAK;FNHGTIEHPNCPGDVLPDDCSK;IIQIAPVDLIK;KDYFGWDK;LEAAYNAK;TLLKPTVHLNR;YANVNGFEGEFNSQYSVQSYNK;YGTIER;YGTIERIIQIAPVDLIK | 0.4314 | 30.6569 | 27 | NA | |
| 3H-109 | ALIVKPMNQFR;CVDAVPFK;EVHVSVTTLMK;IAQYPINTK;IDYKETQR;ILSLDAVSVPIDQK;KSISDDLIR;LTFFKK;NSWGEHWGDGGYFK;QAVSSSGCGGGSTYR;SISDDLIR;SQLHFETHESK;SVIPTNATTNAYNAVK;VALIMSIDAVIR;VGGAYFVTVR;YLDSLIPTPR | 0.3966 | 50.4833 | 24 | cathepsin B |  |
| 3H-69 | AAILLGASLVLNK;DESHYMLYTK;DYDTGKDESHYMLYTK;LVYKDYDTGK | 0.4231 | 13.7558 | 24 | NA | |
| 3H-126 | ALNTHDAGAYK;APVTATPVPVKPLYTAR;DTLTLR;ELPIWYDKFEEHVSEYLER;EYFLLSVAQIFR;FDFDIK;FVNDFFTSLFPDPEVK;GPSVVTVAK;HLEGFASLLLK;ISGSDVSQGLR;IYIDNER;LAVTDEIAGSDDINVGQAK;NDEALIR;STAAAITETVAGILK;SVLISNK;TDIDSTVIADK;VFWALK;VTYASIFYDAK;WTLEVINWR | 0.1996 | 56.8369 | 23 | ATPase involved in DNA replication |  |
| 3H-66 | CIADLR;DIIMGHISPNR;DTFAVSPAR;IFLATNTNSHVR;IINDKLK;KVQVDYSPNYDLTVVTVNDHVSTTK;SALVNCYAK;TADGDYDDDER;TVISGDTYIK;VQVDYSPNYDLTVVTVNDHVSTTK;VTVHEVNADLYLCKPR | 0.3135 | 33.2082 | 21 | NA | |
| 3H-71 | LGKPIVVIAPVR;LGKPIVVIAPVRR;LSESVAVIEAK;LSESVAVIEAKR;NEVAWLLR;NLFTNIR;RNLFTNIR;SRNEVAWLLR;VQFPHVVHNPELNVIYWR;YCADIFDIVQHAASL | 0.3606 | 31.1249 | 19 | sulfhydry1 oxidase Erv1 like protein |  |
| 3H-77 | CVENDKPATK;MAFVHLFHIDGILECAER;QWATDELK;VAALKQWATDELK;VHGWWWR;VHGWWWRR;VQKVHGWWWR | 0.6265 | 22.0611 | 17 | NA | |
| 3H-135 | ALNMILK;ALNMILKR;DQVPVLTK;EFPNISCR;KRPIQPTAYNLFYK;RPIQPTAYNLFYK;RPIQPTAYNLFYKDQVPVLTK;YLLDNYNYR | 0.376 | 23.2266 | 15 | yabby-like transcription factor | |
| 3H-137 | AMSNGLGDVILSAK;DTMAMIFK;IFDAFK;IFDAFKR;KIFDAFK;KVSGDVIDK;NALLTLK;TGVDLSVNVGQTYK;VSTTVDPMMPAQVK;YSVAVAK | 0.5062 | 29.5646 | 14 | hemolysin-like protein | |
| 3H-28 | IFMLVLLER;ITALYHNVK;LDEHVPPVAAFIR;LTEWFPNNR;NNLNYDQLYTALR;NVAEYQSR;QHLLDER;TAYDQADVIAYIDGDKEK | 0.2429 | 25.1107 | 14 | NA | |
| 3H-84 | LLSPPVDPHVFIDTTAASIDK;NSEEDSSPAQADQSESVGSSPPTK;SIDTNEGNDDDSTAIALR;SPHLDMAFR;TGTEPKPEHSDDGKNSEEDSSPAQADQSESVGSSPPTK;TMEFVR | 0.3983 | 21.4649 | 14 | ABC-type transport system permease |  |
| 3H-79 | FPFWFER;NEPVSYLAVGNAQFIDR;RNEPVSYLAVGNAQFIDR;SGLNYIITFDSSATEK;SPNCALVFTR;TNPELNDIER;VPVSNTVEFVIVILK | 0.6080 | 24.8040 | 13 | NA | |
| 3H-59 | ALDELMDFK;ALDELMDFKK;KDNEISQLHEELDYATETINDLNDQLR;LQSELTMVR;SGHTDNDDWDYYDIVDETADVIQEPMLR;TAAQQLASYR;TELDHIK | 0.5291 | 21.3989 | 12 | putative nucleic acid-binding protein |  |
| 3H-37 | AGLLELVIK;IDALDDFEVWR;ILEYSNAPNAIAK;LHSQVTNLHPLSK;LSELNVASAYDFRPVFVVPTK;QLEAVLHTK;QTLEEQLPR;YWLVNELFPSLK | 0.3368 | 24.0435 | 11 | bro4 |  |
| 3H-4 | DFNEFIVWVPTK;LNNLIR;LSHDYAYSLR;NQPNNYVMVR;NSDFAWK;NVTFYVTIK;SNLRLNNLIR;SSTVVSTVSSATWVK;VYTNVSDIPSAEK | 0.3644 | 27.2797 | 11 | NA | |
| 3H-15 | DGTVVISEDDKHNEPYGIGK;FFNFKEDFTR;IIPNNESADSR;KTIEALISLPR;SVADVCNPGGVYLGGR;TIEALISLPR;VIVNNPR;YYVDDLVK | 0.2795 | 25.4288 | 10 | NA | |
| 3H-116 | ETFYQVFSQVTSVDHR;FGCLEYR;IAFVGKPGTGK;LWEFADDFPNK;TCLDGIFLFGTGNHDVR;VTTPVVGSSPSESASSPIR | 0.2275 | 20.4932 | 8 | ATPase involved in DNA metabolism |  |
| 3H-118 | EDVIHDR;LNGQLHTLVIPK;SIVAAFATADLPTALYHSETATYR;YAYTDSDGVYVK | 0.3274 | 13.7558 | 8 | NA | |
| 3H-150 | LAVEYNGR;LQVIFDDNDEKR;QHYEYVPR;SADDALLDPTTSPSPVFAR;VRPDWLINPNTK | 0.2532 | 15.5675 | 8 | NA | |
| 3H-182 | APQQIATVK;CDDGELTLHR;DVEFVAR;HTGGLIITAPLVHPAVGTTR;SITLQNTVTK;SLPDPLDIYVR;SSIIHNIQK | 0.1924 | 20.7292 | 8 | NA | |
| 3H-26 | ANSIIDNIER;QIVDPNATMK;SGATYAINNSYTIDALR;TSEIVDVVDATR;TSEIVDVVDATRK;YTDMVLVLHNLNYVPDYIAEFCR | 0.5448 | 20.7742 | 8 | NA | |
| 3H-139 | AHIAPALLNHIESNLLK;ATLDEVSNDPDDIDVER;FVFSARPTVDSSLASGGVR;ILSLTR;QTTVDKWIDSR;VAAYEIAR | 0.2816 | 19.0726 | 7 | putative S1/P1 nuclease | |
| 3H-21 | ATENELDR;EVEDLVLR;FKEVEDLVLR;IASLLVHDIHSSLDDAVK;IGSIAPIELNPPPR | 0.2193 | 16.3280 | 7 | NA | |
| 3H-63 | EYVFGVR;FLILSASGPLK;GSVSDLEFK;LWNASVDSFCYR;SGDEIDGYIIMKPK;TAFSIVNK;YLWTFAPLLEQR | 0.2425 | 21.9608 | 7 | NA | |
| 3H-72 | EFEYFK;ELQPPYSNYFEESLKPEAFVSNIHYK;LSSLLTSIVSSHSGMIHDK;LSSLLTSIVSSHSGMIHDKR;VTTSSILR | 0.3871 | 17.0933 | 7 | phenylalanyl-tRNA synthetase α subunit |  |
| 3H-76 | EFPLVNPAR;ELYPEHVK;FNEYDATLR;ILFEYAPVNVYYSTIESR;LVLGSQSPYNAYVLNYGSAENQLR;SDLNALLR;SLWSTDETLPR | 0.2153 | 21.7802 | 7 | NA | |
| 3H-82 | ATITEVGNR;GGHGSVGEVHTSAVVR;TVSAVGSNEELANTDDSSR;VCAAFQIKPR;YDGEALTR | 0.1896 | 17.5954 | 7 | NA | |
| 3H-83 | AVTVTKGIAR;ESFGTYMGQR;KPAQELIHEYR;MSPELQEAWR | 0.2697 | 13.7558 | 7 | glycosyltransferase | |
| 3H-157 | DLPDTVPVSLDVR;EQITQWLR;GINDLSDAELAYK;NPPLELWSR;TLLLHYR;VLIDTEQKR | 0.3410 | 17.6500 | 6 | tyrosyl-DNA phosphodiesterase |  |
| 3H-23 | INYAATVNNNAHR;SPLNYSHENIER;VIEEIFK;YGTFAVDSAR;YVFNVDSVK | 0.0659 | 14.5068 | 6 | NA | |
| 3H-24 | EGVLEITNSK;IAEIVVK;IDYDYLVSPQLK;LLNYEYFEYIGDSVVNR;SANTTSVNAVNYR;YGNVDSQYYK | 0.0948 | 17.3691 | 6 | RNaseIII |  |
| 3H-171 | ILIVINQR;LGFQEFVYR;LKDFLTLFDVDELR;MFVVQTNR;VIDTFSK | 0.1465 | 15.7029 | 5 | caspase-like protein | |
| 3H-46 | HSDDIDTTIDALVK;KLDQEVLIQEMR;YMEVSSNDTLLYDNVR | 0.1634 | 11.0482 | 5 | NA | |
| 3H-8 | DGYVQGIAAVYYAPHTSAATDQAFK;LNAQDVAVLGTSNPILR;LSDIHPDR;VYIPVPPITR | 0.1379 | 13.3444 | 5 | NA | |
| 3H-113 | AAGFIVIEQDINTDNVTLVHEALR;ALVVDPPQSVVR;EWFVQK;RLDIAFSGTDK | 0.1536 | 11.9840 | 4 | lysophospholipid acetyltransferase |  |
| 3H-124 | GYLLAAIK;INDPVLVIDSAR;TLLEYNSLYR;VGENSAWHNFETR | 0.0655 | 12.4335 | 4 | serine/threonine protein kinase |  |
| 3H-131 | DVAVALEFVNTEQAIR;LPAAEEFQR;SKLPAAEEFQR;WLFEEVLPTGK | 0.1949 | 12.2788 | 4 | bro16 |  |
| 3H-29 | ISPIFDGSVAILHDLSSGLVAAAISK;NHGTSVSIVGDDHK;NSSVVMVK;VFNDINVVK | 0.5816 | 15.0781 | 4 | NA | |
| 3H-70 | CEYVTKSTVCNESNDMLTLTESNPIDR;STVCNESNDMLTLTESNPIDR;YSGAGKPVSPNDFLMR | 0.5119 | 11.0482 | 4 | NA | |
| 3H-90 | EFVDVHSSDTEADEDSHSSDSPSR;LELPEETFIK;TGASTGYSSDSPSR | 0.1765 | 10.0068 | 4 | *iap*-like protein-5 | |

Table S16 Identified HvAV-3h coded proteins from *Spodoptera litura* produced virions (Sl-3)

| Protein ID | Identified peptide sequence | Sequence coverage | Protein Q score | Unique spectra | Function annotation |
| --- | --- | --- | --- | --- | --- |
| 3H-57 | AEMQINTQR;AKLDLVTINSVANK;ALEMKEMWER;ALLEVTDNVVTDR;ALLEVTDNVVTDRVPVR;AQLDVADLER;AQLDVADLERR;AQLQSDLDVLR;ATIDALTANLNSSNSK;CGDITQMNR;CQAELQQR;DEAIWAIK;DEAIWAIKQHPSFANKPNNTDVQQSDLTIAEDADTGTSVTK;DQLLKLWR;DSSFPIVDADNTEQLVVR;DVNLAKSETNALVVEVADLK;EKAQLQSDLDVLR;EKQHAEFLR;ELACNVIPEK;ELACNVIPEKCGDITQMNR;EMYAVLEDELK;GELNAMTR;GRISELLTQLQESNNQR;HANDVR;HENLYLASTTPSR;HIAGVITK;HIAGVITKNIDGAEGFTR;HVGNAQSDGMYTENVALR;IESEAALVESLQR;IQAFVNTEQNILAAGTR;ISELLTQLQESNNQR;ISNSVKNDVESK;ITNEFFDHATLPLLR;KQMGNVVQEIAQR;LDGSDVTLVVDDENLK;LDLVTINSVANK;LDLVTINSVANKVNR;LIDSVVK;LIDSVVKITR;LLISSETLGR;LLKEHDVLER;LLSLDGVR;LLSLDGVRR;LLVSEDK;LNDIAELQSQLTK;LNKELNTVR;LNNELETSITEER;LQDEIK;LQLDYMTLQQR;LSNASVGVQLDR;LTNECQAVSSR;MEFEAVNDALSK;MLELQNLMK;MTAAHLADDR;MTAAHLADDRLSNASVGVQLDR;NAEITNLQDR;NDVESKK;NEIGQLR;NELAATVNSLQMQATK;NIDELR;NIDGAEGFTR;NIDGAEGFTRDEAIWAIK;NISAIRPR;NNVQLQTALQR;NSGVGNAINDR;NSGVGNAINDRLNK;NVNDTTLAELAQSR;QHAEFLR;QHPSFANKPNNTDVQQSDLTIAEDADTGTSVTK;QHPSFANKPNNTDVQQSDLTIAEDADTGTSVTKLLISSETLGR;QIDSLR;QIDSLRR;QLAQNER;QLNALNLR;QMGNVVQEIAQR;QMKHENLYLASTTPSR;QQLEWILVNSNPAIALQDASVR;QSLLETSAQLNDVR;RLLVSEDK;RLNNELETSITEER;RNNVQLQTALQR;RPIRPMADNFAEGYVYERPMGGGAPR;RPSVTDDEDDRIVGTR;RSLSYSLTDDEDDAAQIR;SDVVVLR;SETNALVVEVADLK;SETNALVVEVADLKSQLNR;SEYELSASR;SEYELSASRR;SLSYSLTDDEDDAAQIR;SNANLSPQSIR;SQALYAIK;SRSDVVVLR;STMLER;SYIIELESR;TKEMYAVLEDELK;TNDNLEIK;TQLLTGLR;TQLLTGLRLYR;TYQQEMTQLK;TYQQEMTQLKR;VAYANLEGDMQK;VITGDETVMTYNR;VNRNSGVGNAINDR;VSTDDLTSYSR;YEALMSEQSSK;YESLMTK;YNLIKTNVQQQR;YQAAGTPGNGR | 0.6478 | 427.6553 | 358 | chromosome segregation SMC like protein |
| 3H-5 | ALFFGVR;ALFFGVRNITHSEYLDYYEVGLPK;DSGVALPIGMMVNNR;EIRPCAAFSK;ENVPDTGAGTGFMNMLTDGDFATPPEITK;GQYMYSFALDLK;GSINPSNFNSSISVVIEPSEALR;GTIDSYIYGPDEQVTTYFVR;IGIKCGDR;KIDNGGDFN;KLSDLLIK;LSDLLIK;MPVLLSTGNGSNK;NITHSEYLDYYEVGLPK;NITHSEYLDYYEVGLPKSNNGVR;NRGQYMYSFALDLK;RGTIDSYIYGPDEQVTTYFVR;SDVNKR;SIGVSALSK;SIGVSALSKIGIK;SVDIGAKTLVLPIPLYFSR;SYAAGTESSYFPK;SYAAGTESSYFPKLAHK;TLVLPIPLYFSR;TSCSPHYILMEKPTDIAATEILKPTEAGSTLSYDIEHSSGILK;TVDPKGSINPSNFNSSISVVIEPSEALR;VPMLPAEHFVFTEPYR;VTVEFVFR;VTVEFVFRK;YRVPMLPAEHFVFTEPYR | 0.6681 | 124.7064 | 211 | NA |
| 3H-50 | ASCGTIPDAR;ATHYDPIR;ATHYDPIRDGYYMYALPNGR;DGYYMYALPNGR;DGYYMYALPNGRVDR;DYFLSSK;DYFLSSKYK;FLKPTFWFSR;IRNALVALSNSSMNNEQNYTNR;LPSDSGVAVTTSPTVVDDLDLR;NALVALSNSSMNNEQNYTNR;NVYKDPAR;VDRSLWR;YDNFQCNK;YKDDAR;YNNLSWLLSK;YNNLSWLLSKNVYK | 0.6878 | 62.0024 | 132 | NA |
| 3H-152 | AIYYVSQGK;ASYMNLLIVEQDR;ATCIFIDETHLMR;AVHCLQSSTADPR;CPGNLEAMLAVQR;DMVSPSNADGPR;DNTSYFDPLR;DVAVANSDKTAPR;EHITFNPGR;EISFDMFDALYGNGDDADVR;EKVVQGATAVR;ELLNPEHSAK;ERTSPTLILHVWR;FIYEYNNYTVR;FNLLHDTINSLFEAGVVDR;FSVTVESTR;FSVTVESTRR;FVEHCIIDAYTSNR;FYDDYTQAWNDNEDVLSIPYHYAGADYHDMK;FYTEHLHTDELLNINVR;GAVQVGPNAAIEAVMR;GLPDVYLYCNHDDAPYPGLAANR;GTLMSELFTVDER;HDDTNTVIVQYSVDK;HDDTNTVIVQYSVDKHYLSR;HPPTADTALSAMSR;IYDAPNTNVLYGIANDITSHMIQYK;KGTLMSELFTVDER;LIGMDGVQLETIR;LVYNASLLLR;LVYNASLLLRR;MSAYETSEIR;MSAYETSEIRK;MVWFPEFVSPVSDDAPR;NAAADACDNDSLAPEVR;NHIALFMVR;QLSAHWR;RELLNPEHSAK;RFNLLHDTINSLFEAGVVDR;RGIYSPGMTYDIAHVYPAPGVGYTISVLSNNAPTVDVPR;RLVYNASLLLR;RMSAYETSEIR;RVDDAELER;SGVHSAVDMYMYIR;SIINVNNYAR;SLSDAVQMVK;SLVIGAEQLER;SQTFYNTQR;SQTIDGVGR;STDTNSATFYPK;SVDLVPDGK;SVDLVPDGKEHITFNPGR;TSPTLILHVWR;TVSLFRPPLSLK;TYQALLPQPMR;VDDAELER;VLLHLTAYTILK;VLLHLTAYTILKK;VLPLTAFGK;VLTDSVDTAR;VLTDSVDTARR;VVQGATAVR;YETDFTSDESPFSIVTSIDK;YETDFTSDESPFSIVTSIDKFILKPRPK;YGEWLK;YITELLR | 0.5473 | 261.8369 | 116 | NA |
| 3H-13 | AAVWLATTTGDDSGYR;ALSSVALTCR;AVMNSFR;CVDEFR;CVTWSGEK;DIGIDTGDPSSVQNR;DIISPLILLDAER;DPYDTLNK;DVIHEHVVAHWNDAETEQIIGR;ESPTEYFLR;EVMTVPSIVSALK;EWNATFVVIDEAHDLSVHGVDYPK;FVFPDGTYGAEGYATWMDSK;GANVAIILGSR;GLVLTPNR;HGQLDVDK;HGQLDVDKYIDCNGQYISISK;HLNDFEIDR;HTEGLYPMTR;KAICYNIR;KFAGLVSYLPSIVDVNEVDIVNVGER;KVLLLTATPMR;LSTHDITVEQFNR;LTTYAEFNETQLLYSDDDDRDR;MIQYYGTSTNGDNDIR;NELNCYYR;NITNDNTIPLDAPR;NNLSDLVPLHNLLMK;NTVIVVHDMSDVMSATYHYAGAGER;QRPTFVYPFVLDNVSLYGLDAYR;QRPTFVYPFVLDNVSLYGLDAYRDTLAK;RGLVLTPNR;RPMPHQILVANYTTPR;SDRDVALLR;SFIAQLDR;SFLCLTR;SKLEELGAVITDVTCGVQAK;SPIDGIIVIHGVGTGK;SSIEAQLNNDMLAK;SVINAAER;TFFFNTITAFANTVSR;TLTAILAMINNITAGHDQGMK;TSDVVLHK;TSDVVLHKEWNATFVVIDEAHDLSVHGVDYPK;TVDILMYATSEAK;VIAEGITLK;VLLLTATPMR;YALPLYPIAESTDESWSYEHLDK;YIDCNGQYISISK;YLSKTFFFNTITAFANTVSR;YQSHAYTIADWGSDAKPNVFVYR | 0.6695 | 197.9748 | 96 | DEAD-like helicase |
| 3H-27 | ADNVWVYTEDLQAIK;ALINEDGHSVVTQR;AMIESILK;CLLSITR;DDNSPSNDSVAMK;DPALYEVQLHTCR;DRSLVVLTLQHELR;DSANSLLK;DVLAKTGYANLK;FNIDNVNYASVAHAIALISVVYHMK;FSVLNSPLSR;GGVVTLSGVR;GVVWSEQSQQLVAAAATTK;HIHSVGDPSYNDEVTR;HITYVVVGHALVSHR;HSNVYSDALESFR;IVTSQLLYK;KADNVWVYTEDLQAIK;KCLLSITR;KVDEFISFGR;LYSTANELLADVLYGDGVDLGSTVSETTNR;NAFTSIIWNVDIFK;NLCEIGVVSNVTDVDLLR;RPDAEECFK;RYAIDVNILPAISDTIQK;RYDMDYYNGNMYSDLYATGNSFTLK;SLVLHFGANPAYTIMNTFASPAESPR;SLVVLTLQHELR;SLVVLTLQHELRK;SPPTLTIDDDSILYPLQYIPR;STEDDRPIQMQR;TDYELR;TGYANLK;TLFGDVVQK;VDEFISFGR;VLTAALMHTDADTVNPTTPSVGYESDAVPR;VSDPQTTVR;YAIDVNILPAISDTIQK;YAIDVNILPAISDTIQKSR;YKHFDIR;YTDFDQLWSK;YVDMSQYR;YVPINR | 0.6509 | 170.8501 | 86 | NA |
| 3H-55 | AYELANER;AYELANERGPFSVEVYLNPGTSNTYQYVATTR;FNNTDYDDLPWNYTSGK;FNNTDYDDLPWNYTSGKK;GIASLWGYLK;GPFSVEVYLNPGTSNTYQYVATTR;IGQTPKAYELANER;KVVTATGVVSGGER;LPQSFTR;NKFNNTDYDDLPWNYTSGK;SEIAQDIQVTMYNTNGGSNPLNNSNVTR;SNVDSSSYYQQPPQVVYNNGDLYGSR;TGYSGAELGASIDK;TGYSGAELGASIDKGIASLWGYLK;VVTATGVVSGGER;VVTATGVVSGGERYVFLLR | 0.7200 | 68.4979 | 82 | NA |
| 3H-56 | DMNGIAVSVDAK;EEDGWDVATTEK;IISETVK;IMNGAGAAGGAVVPK;LKEEDGWDVATTEK;LQPAPLDIK;LQPAPLDIKGGRPMVR;LYPALMSPNTITNVIPDAR;LYPALMSPNTITNVIPDARNGVHSTDR;MELHFEFM;NGVHSTDR;SSVMVIGLSEVFMQQTSVTFR;TIIGDLGSLQGPEGGEWLAFFYPAHTVFEK;VGETPDLSSFSQEEISK | 0.6901 | 56.1700 | 61 | NA |
| 3H-19 | AELEGVADNIK;ATVGALAGDVVK;ATVGALAGDVVKR;CLACQEVVTSTYSYR;DIYIENK;DTIRELGK;ESTTNIAEDIER;ESTTNIAEDIEREPAYLK;FDLLTVDGNDAR;GDDVRPELSTFVLSYTR;IFSMLDNEIHLTIDSSDHQHVLQIYNR;ILITTYDYSGVQQR;IMDVIVSDLGTFNGPIPNDADHYMFK;ISPMYSFHSR;KNTNLYGVTR;LLYWLEDNR;LLYWLEDNRFDLLTVDGNDAR;LVDCSTTR;LVNVTR;MRPTTADVDNAQPTITVDDLK;NEQPQNSSSFDK;NTNLYGVTR;QIGNGEDANANVEINCGHR;QTLGLVIK;RLLYWLEDNR;RTVLDAAR;SETTDASNVSPR;SHVDDNEDLAPSK;STVTIEIVDDNHFEHNVECVLDTK;SYADELGDR;TPSPTAVEVNTLAR;VFDVPPVIHGEIFYGPFDVTGLHSYAGPPSLSIIVSNHPSNGSQSK;VLPQHIR | 0.6269 | 126.3084 | 57 | NA |
| 3H-48 | ALLLSQLVSPPLIAK;AMHLLYIEGK;ATQNTVQTLR;CLGQVVTNVR;CSSTAAVDAAVASSSTEALSQK;GSSISTAVSK;LKNWSPK;LQSWVTDHEAVQYGTKPTVPYVIPDNLLSQKPTDNTISR;LYGYMANK;NDITAKNITSK;NEIIAKNDITAK;SVASVSSPGNIASSSVGNK;SVASVSSPGNIASSSVGNKR;TAQSIMQNLMTQIK;THIFNYLDSK;TVNVNQDSQTIK;TVNVNQDSQTIKTHIFNYLDSK;VDQEVYMK | 0.4362 | 75.6834 | 57 | NA |
| 3H-58 | AVSPLTNKPIDVHGR;DCNLSPPPTK;HIVVSNGFQPVR;KFLANK;KLIGTSPIR;KNPFTGR;LIGTSPIR;LIGTSPIRK;LYNDLTK;MVYSMCHGVPYR;NDQNVPTYYTSSRPFADR;NESINPITGK;NESINPITGKK;RDCNLSPPPTK;SIRPEQAAYR;SQLINIANNMNIAELR;SQLINIANNMNIAELRHIVVSNGFQPVR;SVSPYSGRPIK;TAANADTRPDLCATFSR;VAQNTTQSQLLNIVK;VAQNTTQSQLLNIVKFQIR;VLLSPIPDGATPMSR;YMINPNFVPTTTK;YMINPNFVPTTTKK | 0.3426 | 91.4610 | 56 | cofactor of the virion S/T kinase encoded by thioredoxin |
| 3H-110 | ADGTAIR;ATDINPYETK;FLTNEIK;FLTNEIKADGTAIR;GWVPVNGSDAFEFSCK;HLNPQWATDAPILK;IFEQAGCK;IGFISQILPR;KATDINPYETK;KNVDDDTIELR;LGNVSGIITK;LSFDKK;LTFNGTAK;MAGLPLHAK;NVDDDTIELR;TMYAPLSK;TSNKPLIFECILDEPEK;TYKHLNPQWATDAPILK;TYVTLNR;VVLAPEK;VVLAPEKVYEFISR;VYEFISR | 0.7746 | 80.2562 | 54 | NA |
| 3H-140 | ALSHPDWLTIGSFIK;DVDGVASNGGGSVGTGLGGPLGGTSGVNTSNLR;ELFYHSEAGR;ENFVNLVELWCK;ETNYPIPYFQYNLVLSQR;KQLCNAIK;NSDKDVTIPK;QLCNAIK;SGDEMTLSR;STGTTVGYTGTTSNAAMK;TATHLLGYK;TATHLLGYKNK;TLDRPTDNAIMQR;YTYMYVNK;YTYMYVNKR | 0.7830 | 61.6984 | 51 | NA |
| 3H-43 | AVVFSDSR;DFNVASYESDPLLK;DLYNLQILR;DQSLELLSHTHR;FGLASVNDENSYK;FVEFGTLVRPAGWGSLSELGK;FVEFGTLVRPAGWGSLSELGKR;KDFNVASYESDPLLK;LSLGYQTVVQFYR;LSYDSVVDWER;LTIDER;MFNVDQAIEFFENR;RLSLGYQTVVQFYR;SSPQTVGLEWWK;SSPQTVGLEWWKR;TLSSMIR;VCHLNAVLDPAFTIDTGNVR;YNSSMDEYR;YNSSMDEYRDQSLELLSHTHR | 0.7382 | 79.9076 | 44 | NA |
| 3H-107 | ASLKTNTPLEGVPEFVLFHYGNAVK;FFPAETHVEVK;FYSPSCGACEHVK;IDHVPSAVVK;LQLSDWNDNVTTIK;LQLSDWNDNVTTIKK;MMDHVFTLTPSDVTVR;NYVLNEPPAR;RGILSIR;RIDHVPSAVVK;SPYANAAR;TNTPLEGVPEFVLFHYGNAVK;TNTPLEGVPEFVLFHYGNAVKTLDR;YASDSLMYFECDLSIR | 0.8344 | 57.2126 | 35 | thioredoxin-like protein |
| 3H-117 | CIPVVAYHAQDADAPTDTGLK;DDAIIIDDLPDVYITQPHR;DLHDRYT;DYAAVITAELILR;LDSAESEMAR;LEMFLSSDETR;MFAKPEPAIR;NLNALWDLWGADVIIER;NVNGFVPAVIFSDYEVYK;RLEMFLSSDETR;RPHLDEFLSYLFENFR;SRPTVFLDLDNTLICSLR;TAGEEVGGVK;VGVWTAASK | 0.9524 | 59.6838 | 35 | CDT phosphatase transcription factor |
| 3H-62 | ATLEQVFVR;AYVDLK;AYVDLKDFVVTHENLFAR;DFVVTHENLFAR;INTGSITDSVQCFNK;KTQTPVNILADIVSLK;LDNNFVDGLK;LFVPGIDLPQPYIGIDTTK;LFVPGIDLPQPYIGIDTTKR;LITDPYQLLQISER;RLFVPGIDLPQPYIGIDTTK;TQTPVNILADIVSLK;TQTPVNILADIVSLKYTTSTVPNETDR;VCINVNAMTAGDK;YTTSTVPNETDR;YTTSTVPNETDRR | 0.4384 | 65.6506 | 34 | myristylated membrane protein-like protein |
| 3H-151 | ALQYQTQLEGLLSR;ETCALTTVTASLATISESLGKDEALLK;ITEVGGGVAAIK;ITEVGGGVAAIKSSLTSVVFTPLSEK;LDAIKSDISGK;LVQIDNTVK;RVVVDPLDTK;SSLTSVVFTPLSEK;SSLTSVVFTPLSEKLDAIK;SSTTEER;VAFGLTR;VEKALQYQTQLEGLLSR;VVVDPLDTK;VVVDPLDTKVEK;VVVVPVQDK;VVVVPVQDKITEVGGGVAAIK | 0.6059 | 67.2088 | 32 | Uyr/REP helicase |
| 3H-69 | AAILLGASLVLNK;AAILLGASLVLNKVMVS;DESHYMLYTK;DYDTGKDESHYMLYTK;LVYKDYDTGK | 0.4744 | 18.9339 | 32 | NA |
| 3H-3 | DYFGWDK;DYFGWDKLEAAYNAK;FNHGTIEHPNCPGDVLPDDCSK;IIQIAPVDLIK;KDYFGWDK;LEAAYNAK;TLLKPTVHLNR;YANVNGFEGEFNSQYSVQSYNK;YGTIER;YGTIERIIQIAPVDLIK | 0.4314 | 40.2122 | 31 | NA |
| 3H-109 | ALIVKPMNQFR;CVDAVPFK;EVHVSVTTLMK;IAQYPINTK;IDYKETQR;ILSLDAVSVPIDQK;KSISDDLIR;LTFFKK;NSWGEHWGDGGYFK;QAVSSSGCGGGSTYR;SISDDLIR;SQLHFETHESK;SVIPTNATTNAYNAVK;THILNR;VALIMSIDAVIR;VGGAYFVTVR;YLDSLIPTPR | 0.4112 | 63.9928 | 30 | cathepsin B |
| 3H-71 | KYCADIFDIVQHAASL;LGKPIVVIAPVR;LGKPIVVIAPVRR;LSESVAVIEAK;LSESVAVIEAKR;MMVDVK;NEVAWLLR;NLFTNIR;NVHLVLPCASCAHEAYTYSSSHR;RLGKPIVVIAPVR;RNLFTNIR;SRNEVAWLLR;VQFPHVVHNPELNVIYWR | 0.5096 | 49.7453 | 23 | sulfhydry1 oxidase Erv1 like protein |
| 3H-66 | DIIMGHISPNR;DTFAVSPAR;IFLATNTNSHVR;KVQVDYSPNYDLTVVTVNDHVSTTK;SPASDQSYYGESNDGSYK;TADGDYDDDER;TVISGDTYIK;VQVDYSPNYDLTVVTVNDHVSTTK;VTVHEVNADLYLCKPR | 0.3027 | 37.1610 | 19 | NA |
| 3H-77 | CVENDKPATK;MAFVHLFHIDGILECAER;QWATDELK;VAALKQWATDELK;VHGWWWR;VHGWWWRR | 0.5904 | 23.9577 | 18 | NA |
| 3H-126 | ALNTHDAGAYK;APVTATPVPVKPLYTAR;ELPIWYDKFEEHVSEYLER;EYFLLSVAQIFR;FDFDIK;FVNDFFTSLFPDPEVK;HLEGFASLLLK;ISGSDVSQGLR;IYIDNER;LAVTDEIAGSDDINVGQAK;NSAPQSGVITQIR;STAAAITETVAGILK;VFWALK;VTYASIFYDAK;WTLEVINWR | 0.1740 | 55.5203 | 17 | ATPase involved in DNA replication |
| 3H-135 | ALNMILK;ALNMILKR;DQVPVLTK;EFPNISCR;KRPIQPTAYNLFYK;RPIQPTAYNLFYK;RPIQPTAYNLFYKDQVPVLTK;YLLDNYNYR | 0.3760 | 27.9012 | 17 | yabby-like transcription factor |
| 3H-84 | LLSPPVDPHVFIDTTAASIDK;NSEEDSSPAQADQSESVGSSPPTK;SIDTNEGNDDDSTAIALR;SPHLDMAFR;TGTEPKPEHSDDGKNSEEDSSPAQADQSESVGSSPPTK;VVAKPQYDDR | 0.4156 | 26.3317 | 16 | ABC-type transport system permease |
| 3H-28 | GIDDGDAIYTITK;IFMLVLLER;ITALYHNVK;LDEHVPPVAAFIR;LFFDLR;LTEWFPNNR;NITLSPQYCSEYLTLK;NNLNYDQLYTALR;NVAEYQSR;TAYDQADVIAYIDGDK;TAYDQADVIAYIDGDKEK | 0.3220 | 43.2576 | 15 | NA |
| 3H-137 | AMSNGLGDVILSAK;DTMAMIFK;IFDAFK;KIFDAFK;KVSGDVIDK;NALLTLK;TGVDLSVNVGQTYK;VSGDVIDK;VSTTVDPMMPAQVK;YSVAVAK | 0.5000 | 36.7816 | 14 | hemolysin-like protein |
| 3H-4 | DFNEFIVWVPTK;LNNLIR;LSHDYAYSLR;LSHDYAYSLREK;NQPNNYVMVR;NSDFAWK;SNLRLNNLIR;SSTVVSTVSSATWVK;VYTNVSDIPSAEK | 0.3347 | 35.9495 | 14 | NA |
| 3H-79 | FPFWFER;NEPVSYLAVGNAQFIDR;RNEPVSYLAVGNAQFIDR;SGLNYIITFDSSATEK;SPNCALVFTR;TNPELNDIER;VPVSNTVEFVIVILK | 0.6080 | 27.2066 | 14 | NA |
| 3H-139 | AHIAPALLNHIESNLLK;ATLDEVSNDPDDIDVER;ATLDEVSNDPDDIDVERR;FVFSARPTVDSSLASGGVR;ILSLTR;QTTVDKWIDSR;VAAYEIAR;YICNLK | 0.3069 | 28.0680 | 12 | putative S1/P1 nuclease |
| 3H-26 | ANSIIDNIER;QIVDPNATMK;SGATYAINNSYTIDALR;TSEIVDVVDATR;TSEIVDVVDATRK | 0.3731 | 21.9431 | 11 | NA |
| 3H-37 | AGLLELVIK;EFFTLNEDNFTELIK;IDALDDFEVWR;IGYTCNLNDR;ILEYSNAPNAIAK;LHSQVTNLHPLSK;LSELNVASAYDFRPVFVVPTK;QLEAVLHTK;QTLEEQLPR;YWLVNELFPSLK | 0.4236 | 35.7636 | 11 | bro4 |
| 3H-15 | FFNFKEDFTR;IIPNNESADSR;KTIEALISLPR;SKYPALVKPYEEYQR;TIEALISLPR;VIQTPDPQPVVEGK;VIVNNPR;VVIDTKPVWGNMLPQLK;YYVDDLVK | 0.3131 | 34.4951 | 10 | NA |
| 3H-150 | LAVEYNGR;LQVIFDDNDEKR;NIDLIDVPYTIPIDEIPNFIDK;QGVNTLAEYGK;QHYEYVPR;SADDALLDPTTSPSPVFAR;VRPDWLINPNTK | 0.3948 | 27.4759 | 10 | NA |
| 3H-63 | FLILSASGPLK;GSVSDLEFK;KNVGLFIPK;LWNASVDSFCYR;NVGLFIPK;SGDEIDGYIIMKPK;TAFSIVNK;YLWTFAPLLEQR | 0.2492 | 29.3966 | 10 | NA |
| 3H-72 | DQPMAGAVVETTGDWR;EFEYFK;EKYESCIR;ELQPPYSNYFEESLKPEAFVSNIHYK;LSSLLTSIVSSHSGMIHDK;LSSLLTSIVSSHSGMIHDKR;RVTTSSILR;VTTSSILR | 0.5419 | 25.9106 | 10 | phenylalanyl-tRNA synthetase α subunit |
| 3H-59 | ALDELMDFK;ALDELMDFKK;HVSAEIELK;KDNEISQLHEELDYATETINDLNDQLR;LQSELTMVR;TAAQQLASYR;TELDHIK | 0.4186 | 24.7986 | 9 | putative nucleic acid-binding protein |
| 3H-76 | EFPLVNPAR;ELYPEHVK;FNEYDATLR;ILFEYAPVNVYYSTIESR;LVLGSQSPYNAYVLNYGSAENQLR;SDLNALLR;SLWSTDETLPR;VYLMSVEEAK | 0.2401 | 30.0549 | 9 | NA |
| 3H-182 | APQQIATVK;CDDGELTLHR;DVEFVAR;HTGGLIITAPLVHPAVGTTR;SITLQNTVTK;SLPDPLDIYVR;SSIIHNIQK | 0.1924 | 28.9635 | 8 | NA |
| 3H-21 | ATENELDR;EVEDLVLR;FKEVEDLVLR;IASLLVHDIHSSLDDAVK;IGSIAPIELNPPPR;NVETFVR;YNNDSIR | 0.2807 | 25.5122 | 8 | NA |
| 3H-8 | DGYVQGIAAVYYAPHTSAATDQAFK;FLFQR;LNAQDVAVLGTSNPILR;QLQNMYIVVDEFLK;VYIPVPPITR | 0.1632 | 17.9279 | 8 | NA |
| 3H-82 | ATITEVGNR;GGHGSVGEVHTSAVVR;GVHCDNVFDTPK;TVSAVGSNEELANTDDSSR;VCAAFQIKPR;YDGEALTR | 0.2263 | 20.7903 | 8 | NA |
| 3H-157 | DLPDTVPVSLDVR;EQITQWLR;GINDLSDAELAYK;NPPLELWSR;VLIDTEQK;VLIDTEQKR | 0.3006 | 24.7769 | 7 | tyrosyl-DNA phosphodiesterase |
| 3H-29 | ISPIFDGSVAILHDLSSGLVAAAISK;NHGTSVSIVGDDHK;TIADPMLDR;VFNDINVVK | 0.5918 | 15.3073 | 7 | NA |
| 3H-83 | AVTVTKGIAR;ESFGTYMGQR;KPAQELIHEYR;MSPELQEAWR | 0.2697 | 15.5612 | 7 | glycosyltransferase |
| 3H-116 | ETFYQVFSQVTSVDHR;IAFVGKPGTGK;IQEDLFK;LWEFADDFPNK | 0.1264 | 14.3801 | 6 | ATPase involved in DNA metabolism |
| 3H-118 | EDVIHDR;LNGQLHTLVIPK;SIVAAFATADLPTALYHSETATYR;YAYTDSDGVYVK | 0.3274 | 15.7976 | 6 | NA |
| 3H-23 | INYAATVNNNAHR;LLHLVR;SPLNYSHENIER;VIEEIFK;YGTFAVDSAR | 0.062 | 18.6796 | 6 | NA |
| 3H-24 | EGVLEITNSK;IAEIVVK;IDYDYLVSPQLK;SANTTSVNAVNYR;VNADSVYTDETAANVHR;YGNVDSQYYK | 0.0948 | 23.3078 | 6 | RNaseIII |
| 3H-104 | ADGFHTWFFDSVLSNVISSTTMR;ESYDALSVPSHWR;FVSVVGVYALIVHSDDATR | 0.3526 | 13.1659 | 5 | bro12 |
| 3H-166 | FLIQEDIR;LFLISVVEHEK;NIFNVNK;SLLGVLK | 0.1823 | 12.2830 | 5 | NA |
| 3H-46 | HSDDIDTTIDALVK;KLDQEVLIQEMR;YMEVSSNDTLLYDNVR | 0.1634 | 13.1659 | 5 | NA |
| 3H-70 | STVCNESNDMLTLTESNPIDR;YSGAGKPVSPNDFLMR | 0.4405 | 8.7772 | 5 | NA |
| 3H-93 | ALEAAGSVVVPVHVGR;SAYEWYESR;SINGLLLPGGK;SYVVASYVK;YFYDYFVQK | 0.1731 | 17.1770 | 5 | gamma_glutamyl hydrolase-like protein |
| 3H-128 | DVISDQPR;IATNTDLK;QIQYR;VVGVTYK | 0.1045 | 13.4830 | 4 | NA |
| 3H-136 | GAAVAVSNINILSR;LADVDFLVNYR;TFEEFR | 0.0315 | 10.0561 | 4 | lipopolysaccharide modifying enzyme |
| 3H-138 | STLGVNFIR;TLIELEK;VVLDRPQNVADVER | 0.1948 | 9.0147 | 4 | NA |
| 3H-171 | ILIVINQR;LGFQEFVYR;MFVVQTNR;NMTTADLQDIVTLLTK | 0.1306 | 14.3433 | 4 | caspase-like protein |
| 3H-90 | EFVDVHSSDTEADEDSHSSDSPSR;TGASTGYSSDSPSR;YDMLELLK | 0.1691 | 11.7116 | 4 | *iap*-like protein-5 |

Table S17 Identified *Mythimna separata* larval coded proteins associated to HvAV-3h virions (Ms-1)

| Protein ID | Identified peptide sequence | Sequence coverage | Protein Q score | Unique spectra |
| --- | --- | --- | --- | --- |
| Heat shock protein 83 | AADTLYR;DADDDAQVEDAADDKGDAK;EEAADYLQPDTIR;EHAQNYTFQTEVNR;EIFDLEENDQQK;EIFDLEENDQQKER;ELISNGSDALDK;ELISNGSDALDKIR;FQNIAK;GTHITLHVK;IPDTEYDSFWK;KIPDTEYDSFWK;KPSEVKDDEYTSFYK;KQFEPLTTWLGNK;LIINSLYR;LLSLTDRDVLETNSDLSIR;LSTWVTR;MKPNQNHIYYIAGSSR;NELINNLGTIAK;QFEPLTTWLGNK;QHVWESDANAFSVAEDPR;RVQDAPDAPETAR;SPAALAATAFGWTGNMER;TDAEAVLR;TDAEAVLREEEAISPDGLSVAQLR;TETVEEPDADADADDK;TVWDWELMNDNKPIWTR;VQPADSFNR;YSQFINFPIYLWASR | 0.4157 | 102.9271 | 47 |
| Heat shock protein 70 | APAVGIDLGTTYSCVGVFQHGK;ARFEELNADLFR;DAGTISGLNVLR;FEDATVQADMK;FEELNADLFR;FELTGIPPAPR;GEDKTFFPEEVSSMVLTK;HWPFEVVSDGGKPK;IINEPTAAAIAYGLDK;IINEPTAAAIAYGLDKK;ITITNDK;KFEDATVQADMK;LLQDFFNGK;MKETAEAYLGK;MVNHFVQEFK;NALESYCFNMK;NQVAMNPNNTIFDAK;NTTIPTK;QTQTFTTYSDNQPGVLIQVFEGER;SINPDEAVAYGAAVQAAILHGDK;SQIHDIVLVGGSTR;STAGDTHLGGEDFDNR;TFFPEEVSSMVLTK;TTPSYVAFTDTER;TVQNAVITVPAYFNDSQR;WLDSNQLADKEEYEHK | 0.4778 | 94.9353 | 47 |
| Heat shock protein 83 | ADLVNNLGTIAK;APFDLFENKK;DQVANSSFVER;DTSTMGYMAAK;EDLTEYLEEHK;EDLTEYLEEHKIK;EGLELPEDEEEK;EGLELPEDEEEKK;EKYTEDEELNK;GTQIVLHIKEDLTEYLEEHK;GVVDSEDLPLNISR;GYEVVYMTEPIDEYVVQQMR;HFSVEGQLEFR;HIYYITGENR;HIYYITGENRDQVANSSFVER;HNDDEQYVWESSAGGSFTVRPDHGEPLGR;HSQFIGYPIK;IEDVGEDDEEDKK;IRYESLTDPSK;KHLEINPDHSIVETLR;KYYEQFSK;LGIHEDSQNR;LGLGIDEDEPIQVEEANVGDVPPLEGDADDASR;NADDITQEEYGDFYK;NILDNKVEK;RAPFDLFENK;SEGTLTLIDTGIGMTK;SLTNDWEDHLAVK;YESLTDPSK;YESLTDPSKLDSGK;YHTSASGDEACSLK;YTEDEELNK;YYEQFSK | 0.4854 | 118.1054 | 43 |
| Heat shock protein 60 | AAVEEGIVPGGGSALLR;AEQIRDQIQETTSEYEK;ALMLQGVDVLADAVAVTMGPK;ATLSDMAIATGGVVFGDDSNLLK;DKFQNIGAK;DQIQETTSEYEK;DQIQETTSEYEKEK;GVMLAVDAVK;GYISPYFINSSK;IGLQVAAVK;ISNVQTIIPALELANQQR;KATLSDMAIATGGVVFGDDSNLLK;KISNVQTIIPALELANQQR;KPLVIVAEDVDGEALSTLVVNR;LENLQQSDLGQVGEAIITK;LVQNVANNTNEEAGDGTTTATVLAR;TANSDQATGVEIVK;TANSDQATGVEIVKK;TLVDELEIIEGMK;VEDLGPEFGYDALNNEYVNMIEK;VEFQDALVLFSEK;VNDALNATR | 0.4912 | 85.3914 | 33 |
| Prophenoloxidase 1 | ADVSQLETWR;AMVELDK;AVTTTIANR;DPVFYR;EDIGINLHHWHWHLVYPFDSADR;EVSNVVTSGNR;FTHLQHQEFNYVIEVNNTSGQGAMGTVR;FTTGLRPGNNTIR;GELFYYMHQQIIAR;GIDMLGNMMESSIISPNR;GTPQGYPVVLFAMVSNWNEDR;HLEQFGVMGDSATAMR;IFMAPTVNENGEPLAFDEQR;IFMAPTVNENGEPLAFDEQRR;LDFAGIR;LDSQVASR;LIDIFMNMR;LSPYTDDKLDFAGIR;QVPIDEER;RGELFYYMHQQIIAR;SLDSSVTIPYER;WHSYIDDLFALYK;YMNFR;YMNFREPIAEGYFPK | 0.4058 | 86.5055 | 31 |
| Vacuolar proton pump subunit B | AVVGEEALTPDDLLYLEFLTK;GFPGYMYTDLATIYER;GPPILAEAFLDIQGQPINPWSR;HVLVILTDMSSYAEALR;IPASILAEFYPR;IYPEEMIQTGISAIDVMNSIAR;LADGTLR;LALTAAEFLAYQCEK;NFISQGNYENR;NTLCEFTGDILR;QHVLAVSR;QIYPPVNVLPSLSR;RIPASILAEFYPR;TPVSEDMLGR;TVFESLDIGWQLLR;TVSGVNGPLVILDEVK;VFNGSGKPIDK | 0.4608 | 64.8233 | 29 |
| Prophenoloxidase 2 | DLSAQSNDPR;DPFFFR;DYTATDLEEEHR;ESSYVRPYTR;FMDSQVFQQAR;FVVPLNAGENTITR;KMFIEMDR;LLNQTEPNPR;LSTESSVTIPFEQTFR;NLVWALTEQR;NVPIQNFAETFPSK;QIEEAISTGMVTHSDGSR;SALDIDK;SALDIDKLGNMLESSILSPNR;TLTNYPSFSK;TPIIIPR;YLESFGVIADEATTMR | 0.2785 | 64.9393 | 21 |
| Beta-actin | AGFAGDDAPR;DLTDYLMK;DLYANTVLSGGTTMYPGIADR;DSYVGDEAQSKR;EITALAPSTMK;HQGVMVGMGQK;IIAPPERK;IWHHTFYNELR;LCYVALDFEQEMATAASSSSLEK;RGILTLK;SYELPDGQVITIGNER;TTGIVLDSGDGVSHTVPIYEGYALPHAILR;VAPEEHPVLLTEAPLNPK | 0.4920 | 46.5109 | 19 |
| Growth blocking peptide binding protein | AFQHIFNEDAVTIVNK;DNSVLTVTVAEASR;GFSNCYNVNHQYQLVSSGPDKDR;IPISLNNLIQHVDYWGEGK;LAQRPNFNTNINK;LDASVDSMGDR;LDVNTDSMNDR;LLWGHNGTVYNEYER;LMSFAYK;LMTAFPR;QAWGSNNSNEDR;QFPYSETPYQGDYYLEK;SLTIWK;VIVYGVQGNSQEISELAVELR | 0.4465 | 49.5776 | 18 |
| Catalase | AGELASTDPDYSIR;DLYNAIAK;DPTLFPSFIHTQK;EPATDQLVNFKK;FSTVGGESGSADTVR;GAGAFGHFEVTHDITK;IWPHADYPLIEVGK;LFSYSDTHR;LINAQGVAHWVK;VTVANYQR;VVENIVGHLK;YNVSGDVDR;YNVSGDVDRFDSGQTEDNFSQATELYK | 0.2964 | 48.1320 | 17 |
| Peptidyl-prolyl cis-trans isomerase | FGDENFVLK;HTGPGVLSMANAGPNTNGSQFFITTVK;HVVFGTVVEGMDVVK;QVEAFGSQSGKPSK;TCENFR;TSWLDGR;VIPNFMLQGGDFTNHNGTGGK;VYFDVNADGSALGR | 0.6848 | 29.1973 | 16 |
| Peptidase S1 domain-containing protein (Fragment) | AGEWDTQSTK;CFATGWGK;DVASVETHK;EIYPFQDR;EIYPFQDRDVASVETHK;IELPVVK;IEPVNENEPDGQK;IEPVNENEPDGQKLNVYVGGGSLIHPSVVLTAAHYVINAK;KIELPVVK;LNVYVGGGSLIHPSVVLTAAHYVINAK;YQVILK | 0.5057 | 36.7920 | 15 |
| V-type proton ATPase subunit C | FAWDMAK;FPIPDLK;LDTFVESVTR;NIADIISK;NLADLVK;QFGPLVR;QTGSLLTR;QVGQIDADLK;VAQYLGEVLEDQR;VAQYLGEVLEDQRDK;VGTLDQLVGLSDDLGK;YGLPVNFQAVVMVPAR | 0.2898 | 39.2768 | 14 |
| Heat shock protein 27.2 | DEIEFVPVRY;EEIETNVNDVQDADVGLGR;EGLLMIQANHK;ELDDMLADFSR;FPTSVSQEGVEGTEYK;GDLDKDNEVR;HRPYFSNFDTR;ITFPLK;ITFPLKDGVVVK;RFPTSVSQEGVEGTEYK;VTISLPDFEEKDIVVK | 0.4699 | 44.4587 | 14 |
| Epoxide hydrolase | DVQTVPLLMIHGWPGSVR;EFYEAIPLLTK;EYNLDQITTQVPTWALQAK;FSTWTNKDNK;FYAENFSNK;IREYNLDQITTQVPTWALQAK;NELMYQPPSLLNR;TNIQGLDIHFIHIKPQVPK;YNFVER | 0.2421 | 33.4666 | 14 |
| Glyceraldehyde-3-phosphate dehydrogenase | AASYDAIK;AGAEYVVESTGVFTTIEK;GAQQNIIPASTGAAK;GTVDIQDGFLVVNGNK;IGINGFGR;VIDLIK;VIHDNFEIIEGLMTTVHATTATQK;VPVANVSVVDLTVR | 0.3283 | 27.3671 | 11 |
| Carboxylesterase 6 | ENFVGLIAK;FCQIGLWGNK;GLPPLVSEAPAESLK;LGSLGYLSTGQR;SADQFLK;SATGVAALSGAPLSPGAVRPDPAK;SGSTASYGGQHLVQK;VEGADDLR | 0.1701 | 27.4217 | 9 |
| Poly(U)-specific endoribonuclease | ANYLGYIDK;ANYLGYIDKLDLSGK;GITQLNEYPEQVELLR;NVIRPEFVTPNEETEQTTFINTILATGPIR;QVQDQTTDDDILR;SLMNYLVSK;SYEDLLR;YNNVNFSIQTK | 0.2894 | 27.6046 | 8 |
| Ribosomal protein S2 (Fragment) | AFVAIGDNNGHIGLGVK;EDQKEWVPVTK;EVATAIR;EWVPVTK;IDKLESIYLFSLPIK;LESIYLFSLPIK;LSVLPVR | 0.4191 | 19.9845 | 8 |
| QM protein | AQVIEALRR;DQFHIR;FKFPGR;GAFGKPQGTVAR;LQTGMR;YEREEFEK;YRPEHGPLDAWR | 0.2694 | 20.4471 | 8 |
| 40S ribosomal protein S11 | DVELGDIVTIGECRPLSK;DYLHYLPK;EAIEGTYIDK;EAIEGTYIDKK;QATVFLNR;RDYLHYLPK | 0.3026 | 20.5228 | 8 |
| 40S ribosomal protein S7 | ASAAEPDVFETSISQALVELETNSDLK;ELYITK;IKLDGSQLIK;LDGSQLIK;VDTFQSVYK;VHLDKNQQTTIEHK | 0.3474 | 22.6337 | 8 |
| V-type proton ATPase subunit H | IVLAVFR;LEWSPVHK;LMVHNWEYLGK;LNNNEYVQSVAR;LQTSVQDLSSFDQYATEVK;QLSILEQK;TLVHLLEK | 0.1540 | 26.4931 | 7 |
| Cytochrome c oxidase subunit 2 | IILPMNNQIR;ILVTATDVIHSWTIPSLGVK;LLDVDNR;LLYLLDELNNPLITLK;NFINWINNYS | 0.2775 | 15.9529 | 6 |
| 60S ribosomal protein L32 (Fragment) | ELEILMMQNR;GQYLMPNIGYGSNK;HMLPNGFR;VLVHNVR | 0.4333 | 12.9031 | 6 |
| 1,4-beta-N-acetylmuramidase | CDLVAELR;DWVCLVENESGR;MRDWVCLVENESGR;QGFPEDK | 0.2057 | 13.4330 | 5 |
| Aldehyde dehydrogenase (Fragment) | AGSMWVNTYEHVTPQTPFGGFK;ANNSNYGLGAGVITNDVTTALAFVR;ELGEEGITQYLENK;FETFEEVVDR | 0.7634 | 14.9939 | 5 |
| Tubulin beta chain | AILVDLEPGTMDSVR;LHFFMPGFAPLTSR;NSSYFVEWIPNNVK;YLTVAAIFR | 0.1163 | 14.8247 | 4 |

Table S18 Identified *Mythimna separata* larval coded proteins associated to HvAV-3h virions (Ms-2)

| Protein ID | Identified peptide sequence | Sequence coverage | Protein Q score | Unique spectra |
| --- | --- | --- | --- | --- |
| Heat shock protein 70 | APAVGIDLGTTYSCVGVFQHGK;ARFEELNADLFR;DAGTISGLNVLR;FEELNADLFR;FELTGIPPAPR;GEDKTFFPEEVSSMVLTK;IINEPTAAAIAYGLDK;IINEPTAAAIAYGLDKK;KFEDATVQADMK;LLQDFFNGK;LLQDFFNGKELNK;LSKEEIER;MKETAEAYLGK;MVNHFVQEFK;NALESYCFNMK;NQVAMNPNNTIFDAK;NTTIPTK;QTQTFTTYSDNQPGVLIQVFEGER;SINPDEAVAYGAAVQAAILHGDK;SQIHDIVLVGGSTR;STAGDTHLGGEDFDNR;TFFPEEVSSMVLTK;TTPSYVAFTDTER;TVQNAVITVPAYFNDSQR;WLDSNQLADKEEYEHK | 0.4640 | 98.0523 | 55 |
| Heat shock protein 83 | ALLFVPR;DADDDAQVEDAADDKGDAK;DTSPQLAK;DVLETNSDLSIR;EEAADYLQPDTIR;EEEAISPDGLSVAQLR;EHAQNYTFQTEVNR;EIFDLEENDQQK;EIFDLEENDQQKER;ELISNGSDALDK;ELISNGSDALDKIR;EYSTNIK;GDSMTFLADYVER;GTHITLHVK;HPLIVELLR;IPDTEYDSFWK;KIPDTEYDSFWK;KPSEVKDDEYTSFYK;LALSNAHQK;LIINSLYR;LSTWVTR;MKPNQNHIYYIAGSSR;NELINNLGTIAK;QFEPLTTWLGNK;QHVWESDANAFSVAEDPR;SPAALAATAFGWTGNMER;TDAEAVLR;TDAEAVLREEEAISPDGLSVAQLR;TVWDWELMNDNKPIWTR;VLDMLK;VQDAPDAPETAR;YSQFINFPIYLWASR | 0.4309 | 115.2885 | 42 |
| Heat shock protein 83 | ADLVNNLGTIAK;ALLFVPR;APFDLFENK;APFDLFENKK;DQVANSSFVER;EDLTEYLEEHK;EDLTEYLEEHKIK;EGLELPEDEEEK;EGLELPEDEEEKK;EKYTEDEELNK;ELISNSSDALDK;GTQIVLHIK;GTQIVLHIKEDLTEYLEEHK;GYEVVYMTEPIDEYVVQQMR;HFSVEGQLEFR;HIYYITGENR;HNDDEQYVWESSAGGSFTVRPDHGEPLGR;HSQFIGYPIK;IEDVGEDDEEDKK;IRYESLTDPSK;KHLEINPDHSIVETLR;LGLGIDEDEPIQVEEANVGDVPPLEGDADDASR;NADDITQEEYGDFYK;RAPFDLFENK;SEGTLTLIDTGIGMTK;SLTNDWEDHLAVK;YESLTDPSK;YESLTDPSKLDSGK;YTEDEELNK;YYEQFSK | 0.4296 | 110.2281 | 40 |
| Heat shock protein 60 | AAVEEGIVPGGGSALLR;AEQIRDQIQETTSEYEK;ALMLQGVDVLADAVAVTMGPK;ATLSDMAIATGGVVFGDDSNLLK;DGVITVK;DKFQNIGAK;DQIQETTSEYEK;DQIQETTSEYEKEK;GIIDPTK;GVMLAVDAVK;IGLQVAAVK;ISNVQTIIPALELANQQR;KISNVQTIIPALELANQQR;KPLVIVAEDVDGEALSTLVVNR;LENLQQSDLGQVGEAIITK;LENLQQSDLGQVGEAIITKDDTLFLK;LVQNVANNTNEEAGDGTTTATVLAR;MPCMTIAR;NAGIDGSVVVAK;NVILEQSWGSPK;TANSDQATGVEIVK;TLVDELEIIEGMK;VEDLGPEFGYDALNNEYVNMIEK;VEFQDALVLFSEK;VNDALNATR | 0.5599 | 91.2835 | 33 |
| Prophenoloxidase 1 | ADVSQLETWR;AMVELDK;AMVELDKFTTGLRPGNNTIR;EDIGINLHHWHWHLVYPFDSADR;EPIAEGYFPK;FADTTIR;FIQAIESLSITLPNGR;FTHLQHQEFNYVIEVNNTSGQGAMGTVR;FTTGLRPGNNTIR;GELFYYMHQQIIAR;IFMAPTVNENGEPLAFDEQR;IFMAPTVNENGEPLAFDEQRR;LDFAGIR;LDSQVASR;LIDIFMNMR;LSPYTDDKLDFAGIR;RGELFYYMHQQIIAR;WHSYIDDLFALYK;YMNFR | 0.2920 | 68.2801 | 29 |
| Prophenoloxidase 2 | ASQLPSDADFSLFLPK;DPFFFR;DYTATDLEEEHR;EDIGVNLHHWHWHLVYPFTASQR;EPIPEAYFPK;ESSYVRPYTR;EVAAVVPQTVAR;FTHLNNRPFR;FVVPLNAGENTITR;GLDFSDR;KMFIEMDR;LGNMLESSILSPNR;LSTESSVTIPFEQTFR;MFIEMDR;NLVWALTEQR;NVPIQNFAETFPSK;QIEEAISTGMVTHSDGSR;RGELFFYMHQQLIAR;RQIEEAISTGMVTHSDGSR;SALDIDK;TLTNYPSFSK;TPIIIPR;YLESFGVIADEATTMRDPFFFR | 0.3694 | 77.4479 | 27 |
| Vacuolar proton pump subunit B | AVVGEEALTPDDLLYLEFLTK;DFSSQPR;FSEIVQLR;GFPGYMYTDLATIYER;GPPILAEAFLDIQGQPINPWSR;HVLVILTDMSSYAEALR;IPASILAEFYPR;IPIFSAAGLPHNEIAAQICR;LALTAAEFLAYQCEK;NFISQGNYENR;NGSITQIPILTMPNDDITHPIPDLTGYITEGQIYVDRQLHNR;NTLCEFTGDILR;QHVLAVSR;QIYPPVNVLPSLSR;RIPASILAEFYPR;SGQVLEVSGSK;TVFESLDIGWQLLR;TVSGVNGPLVILDEVK;VFNGSGKPIDK | 0.5594 | 66.3698 | 26 |
| Growth blocking peptide binding protein | AFQHIFNEDAVTIVNK;DNSVLTVTVAEASR;IPISLNNLIQHVDYWGEGK;KLMSFAYK;LAQRPNFNTNINK;LAWGDHNQCK;LDASVDSMGDR;LDVNTDSMNDR;LMSFAYK;QFPYSETPYQGDYYLEK;VIVYGVQGNSQEISELAVELR | 0.3256 | 39.9079 | 17 |
| Catalase | AGELASTDPDYSIR;DAAGFIQER;DPTLFPSFIHTQK;EPATDQLVNFKK;FNPFDLTK;FSTVGGESGSADTVR;GAGAFGHFEVTHDITK;IWPHADYPLIEVGK;LFSYSDTHR;LINAQGVAHWVK;TNQGIKNLPVDK;VVENIVGHLK | 0.2702 | 42.5833 | 16 |
| Epoxide hydrolase | DVQTVPLLMIHGWPGSVR;EAFLNK;EFYEAIPLLTK;EYNLDQITTQVPTWALQAK;FSTWTNK;FSTWTNKDNK;FYAENFSNK;IREYNLDQITTQVPTWALQAK;NELMYQPPSLLNR;TNIQGLDIHFIHIKPQVPK;WFIGAFFPSLIVEEHLASR | 0.2851 | 39.8156 | 15 |
| Heat shock protein 27.2 | EEIETNVNDVQDADVGLGR;EGLLMIQANHK;ELKELDDMLADFSR;FPTSVSQEGVEGTEYK;HRPYFSNFDTR;ITFPLK;ITFPLKDGVVVK;LQPYADDNVFDTGR;RFPTSVSQEGVEGTEYK;VTISLPDFEEK;VTISLPDFEEKDIVVK | 0.4578 | 37.7749 | 15 |
| Peptidase S1 domain-containing protein (Fragment) | AGEWDTQSTK;CFATGWGK;DVASVETHKDFNK;EIYPFQDR;EIYPFQDRDVASVETHK;IELPVVK;IEPVNENEPDGQK;LNVYVGGGSLIHPSVVLTAAHYVINAK;VACLPQPK;YQVILK | 0.5682 | 32.2668 | 15 |
| Peptidyl-prolyl cis-trans isomerase | HTGPGVLSMANAGPNTNGSQFFITTVK;HVVFGTVVEGMDVVK;QVEAFGSQSGKPSK;TSWLDGR;VIPNFMLQGGDFTNHNGTGGK;VYFDVNADGSALGR | 0.5939 | 24.6860 | 14 |
| V-type proton ATPase subunit C | DFSYNEADLAAGK;FAWDMAK;FPIPDLK;LDTFVESVTR;LTDMIVPR;NLADLVK;QVGQIDADLK;SNFNDWNVNYEK;VAQYLGEVLEDQR;VAQYLGEVLEDQRDK;VFVESVLR;VGTLDQLVGLSDDLGK | 0.2950 | 43.1794 | 12 |
| Beta-actin | AGFAGDDAPR;AVFPSIVGRPR;DLTDYLMK;DLYANTVLSGGTTMYPGIADR;DSYVGDEAQSK;DSYVGDEAQSKR;EITALAPSTMK;HQGVMVGMGQK;IWHHTFYNELR;SYELPDGQVITIGNER;TTGIVLDSGDGVSHTVPIYEGYALPHAILR;VAPEEHPVLLTEAPLNPK | 0.4229 | 27.1549 | 11 |
| Carboxylesterase 6 | ENFVGLIAK;FNQFLTQQLQTVK;GLPPLVSEAPAESLK;IGESLTIDKDFR;LGSLGYLSTGQR;SATGVAALSGAPLSPGAVRPDPAK;SGSTASYGGQHLVQK;VEGADDLR;VPLLTGVTSAETSR | 0.2075 | 33.4198 | 10 |
| Glyceraldehyde-3-phosphate dehydrogenase | AGAEYVVESTGVFTTIEK;GTVDIQDGFLVVNGNK;IGINGFGR;VIDLIK;VIHDNFEIIEGLMTTVHATTATQK;VIPALNGK | 0.2410 | 21.5975 | 9 |
| Poly(U)-specific endoribonuclease | ANYLGYIDK;ANYLGYIDKLDLSGK;NVIRPEFVTPNEETEQTTFINTILATGPIR;QVQDQTTDDDILR;SDNVLLIDTAYPFY;SNDVLGLHSWLFFAK;SYEDLLR;YNNVNFSIQTK | 0.3009 | 30.1376 | 8 |
| 40S ribosomal protein S11 | DVELGDIVTIGECRPLSK;DYLHYLPK;EAIEGTYIDK;EAIEGTYIDKK;NVGLGFK;QATVFLNR;RDYLHYLPK | 0.3487 | 23.3096 | 8 |
| 1,4-beta-N-acetylmuramidase | DWVCLVENESGR;DYGLYQINDK;HKFEAWYGWK;MRDWVCLVENESGR;QGFPEDK;YWCSNTSTPGK | 0.3688 | 20.5204 | 8 |
| 40S ribosomal protein S7 | ASAAEPDVFETSISQALVELETNSDLK;EVTFEFPEPYL;IKLDGSQLIK;LDGSQLIK;SIIIYVPMPK;VDTFQSVYK | 0.3526 | 20.5561 | 8 |
| V-type proton ATPase subunit H | DHDFIVNLDR;GQELLR;HIIEQLGGK;IVLAVFR;LNNNEYVQSVAR;QLSILEQK | 0.1097 | 18.1047 | 6 |
| Ribosomal protein S2 (Fragment) | EDQKEWVPVTK;EVATAIR;EWVPVTK;IDKLESIYLFSLPIK;LESIYLFSLPIK | 0.2426 | 18.4428 | 6 |
| Ribosomal protein S5 (Fragment) | AQCPIVER;LTNSLMMHGR;QAVDVSPLR;QAVDVSPLRR;TIAECVADELINAAK | 0.2108 | 12.8615 | 5 |
| QM protein | AQVIEALRR;DQFHIR;GAFGKPQGTVAR;YRPEHGPLDAWR | 0.1781 | 11.4274 | 5 |
| Aminopeptidase N 1 | EESLITTLLEK;LILQLESALK;SLVYNMVLR;VVTQEPIPDQPGWQWTHFDR | 0.0464 | 12.0767 | 4 |

Table S19 Identified *Mythimna separata* larval coded proteins associated to HvAV-3h virions (Ms-3)

| Protein ID | Identified peptide sequence | Sequence coverage | Protein Q score | Unique spectra |
| --- | --- | --- | --- | --- |
| Heat shock protein 83 | AADTLYR;DADDDAQVEDAADDKGDAK;DVLETNSDLSIR;EEAADYLQPDTIR;EEEAISPDGLSVAQLR;EHAQNYTFQTEVNR;EIFDLEENDQQK;EIFDLEENDQQKER;ELISNGSDALDK;ELISNGSDALDKIR;EYSTNIK;FQNIAK;GDSMTFLADYVER;GTHITLHVK;HPLIVELLR;IPDTEYDSFWK;KIPDTEYDSFWK;KPSEVKDDEYTSFYK;KVLDMLK;LALSNAHQK;LIINSLYR;LLHIIDSGIGMSR;LLSLTDR;LLSLTDRDVLETNSDLSIR;MKPNQNHIYYIAGSSR;MLEINPR;NELINNLGTIAK;QHVWESDANAFSVAEDPR;SPAALAATAFGWTGNMER;TDAEAVLREEEAISPDGLSVAQLR;TETVEEPDADADADDK;TVWDWELMNDNKPIWTR;VQDAPDAPETAR;VQPADSFNR | 0.4525 | 106.1773 | 44 |
| Heat shock protein 70 | APAVGIDLGTTYSCVGVFQHGK;DNNLLGK;ELEGICNPIITK;FEDATVQADMK;FEELNADLFR;FELTGIPPAPR;GEDKTFFPEEVSSMVLTK;HWPFEVVSDGGKPK;IINEPTAAAIAYGLDK;IINEPTAAAIAYGLDKK;ITITNDK;KFEDATVQADMK;LLQDFFNGK;LLQDFFNGKELNK;LSKEEIER;MKETAEAYLGK;MVNHFVQEFK;MVNHFVQEFKR;NALESYCFNMK;NQVAMNPNNTIFDAK;QTQTFTTYSDNQPGVLIQVFEGER;SINPDEAVAYGAAVQAAILHGDK;SQIHDIVLVGGSTR;STAGDTHLGGEDFDNR;TFFPEEVSSMVLTK;TTPSYVAFTDTER;TVQNAVITVPAYFNDSQR;WLDSNQLADKEEYEHK | 0.4946 | 91.4548 | 43 |
| Heat shock protein 83 | ADLVNNLGTIAK;APFDLFENK;APFDLFENKK;DQVANSSFVER;EDLTEYLEEHK;EDLTEYLEEHKIK;EGLELPEDEEEK;EKYTEDEELNK;ELISNSSDALDK;ELISNSSDALDKIR;GTQIVLHIK;GTQIVLHIKEDLTEYLEEHK;GVVDSEDLPLNISR;GYEVVYMTEPIDEYVVQQMR;HIYYITGENR;HLEINPDHSIVETLR;HNDDEQYVWESSAGGSFTVRPDHGEPLGR;HSQFIGYPIK;IEDVGEDDEEDKK;IRYESLTDPSK;KHLEINPDHSIVETLR;KYYEQFSK;LGIHEDSQNR;NADDITQEEYGDFYK;NILDNKVEK;RAPFDLFENK;SEGTLTLIDTGIGMTK;SLTNDWEDHLAVK;YESLTDPSKLDSGK;YYEQFSK | 0.4045 | 90.1818 | 40 |
| Heat shock protein 60 | AEQIRDQIQETTSEYEK;ALMLQGVDVLADAVAVTMGPK;ATLSDMAIATGGVVFGDDSNLLK;DGVITVK;DKFQNIGAK;DQIQETTSEYEK;DQIQETTSEYEKEK;GIIDPTK;GYISPYFINSSK;IGLQVAAVK;ISNVQTIIPALELANQQR;KISNVQTIIPALELANQQR;KPLVIVAEDVDGEALSTLVVNR;LENLQQSDLGQVGEAIITK;LENLQQSDLGQVGEAIITKDDTLFLK;LVQNVANNTNEEAGDGTTTATVLAR;NAGIDGSVVVAK;NVILEQSWGSPK;TANSDQATGVEIVK;TANSDQATGVEIVKK;TLVDELEIIEGMK;VEDLGPEFGYDALNNEYVNMIEK;VEFQDALVLFSEK;VNDALNATR | 0.5211 | 79.5581 | 36 |
| Prophenoloxidase 1 | ADVSQLETWR;AMVELDKFTTGLRPGNNTIR;AVTTTIANR;DPVFYR;EDIGINLHHWHWHLVYPFDSADR;EPIAEGYFPK;FADTTIR;FIQAIESLSITLPNGR;FTHLQHQEFNYVIEVNNTSGQGAMGTVR;FTTGLRPGNNTIR;GINIPTFAETFPDKFMDPK;GLDFTPR;GTPQGYPVVLFAMVSNWNEDR;IFMAPTVNENGEPLAFDEQR;LIDIFMNMR;LSPYTDDKLDFAGIR;NIALPNLNLPMELPYNEQFSLFIPK;QVPIDEER;RGELFYYMHQQIIAR;WHSYIDDLFALYK;YMNFR | 0.4175 | 63.0607 | 30 |
| Prophenoloxidase 2 | DPFFFR;DYTATDLEEEHR;EDIGVNLHHWHWHLVYPFTASQR;EPIPEAYFPK;ESSYVRPYTR;EVAAVVPQTVAR;FMDSQVFQQAR;FTHLNNRPFR;FVVPLNAGENTITR;KMFIEMDR;LGNMLESSILSPNR;LLNQTEPNPR;LSTESSVTIPFEQTFR;MFIEMDR;NLVWALTEQR;QIEEAISTGMVTHSDGSR;SALDIDKLGNMLESSILSPNR;WHAWIDDICQSHK;YLESFGVIADEATTMRDPFFFR | 0.3175 | 55.8617 | 25 |
| Vacuolar proton pump subunit B | AVVGEEALTPDDLLYLEFLTK;DFSSQPR;FSEIVQLR;GFPGYMYTDLATIYER;GPPILAEAFLDIQGQPINPWSR;HVLVILTDMSSYAEALR;IPASILAEFYPR;IPIFSAAGLPHNEIAAQICR;LADGTLR;NFISQGNYENR;NTLCEFTGDILR;QHVLAVSR;QIYPPVNVLPSLSR;RIPASILAEFYPR;TVFESLDIGWQLLR;VFNGSGKPIDK | 0.4044 | 51.1188 | 23 |
| Growth blocking peptide binding protein | AFQHIFNEDAVTIVNK;DNSVLTVTVAEASR;IPISLNNLIQHVDYWGEGK;KLMSFAYK;LAQRPNFNTNINK;LDASVDSMGDR;LDVNTDSMNDR;LLWGHNGTVYNEYER;LMSFAYK;LMTAFPR;QFPYSETPYQGDYYLEK;SLTIWK;VIVYGVQGNSQEISELAVELR;VVTEEGVR | 0.3860 | 42.2129 | 18 |
| Peptidase S1 domain-containing protein (Fragment) | AGEWDTQSTK;DVASVETHK;DVASVETHKDFNK;EIYPFQDRDVASVETHK;IEPVNENEPDGQK;IEPVNENEPDGQKLNVYVGGGSLIHPSVVLTAAHYVINAK;KIELPVVK;LNVYVGGGSLIHPSVVLTAAHYVINAK;YQVILK | 0.4830 | 27.8946 | 17 |
| Heat shock protein 27.2 | EEIETNVNDVQDADVGLGR;EGLLMIQANHK;ELKELDDMLADFSR;FPTSVSQEGVEGTEYK;FWSDLSK;HRPYFSNFDTR;ITFPLK;ITFPLKDGVVVK;LQPYADDNVFDTGR;RFPTSVSQEGVEGTEYK;VTISLPDFEEKDIVVK | 0.4859 | 34.5030 | 15 |
| Glyceraldehyde-3-phosphate dehydrogenase | AASYDAIK;AGAEYVVESTGVFTTIEK;GTVDIQDGFLVVNGNK;IGINGFGR;LTGMAFR;VIDLIK;VIHDNFEIIEGLMTTVHATTATQK;VIPALNGK;VPVANVSVVDLTVR | 0.3283 | 26.3083 | 14 |
| Peptidyl-prolyl cis-trans isomerase | HTGPGVLSMANAGPNTNGSQFFITTVK;HVVFGTVVEGMDVVK;IVVELR;TSWLDGR;VIPNFMLQGGDFTNHNGTGGK;VYFDVNADGSALGR | 0.5455 | 21.6095 | 14 |
| Epoxide hydrolase | DVQTVPLLMIHGWPGSVR;EFYEAIPLLTK;EYNLDQITTQVPTWALQAK;FYAENFSNK;IREYNLDQITTQVPTWALQAK;NELMYQPPSLLNR;TNIQGLDIHFIHIKPQVPK;YNFVER | 0.2195 | 26.8913 | 13 |
| Catalase | AGELASTDPDYSIR;DPTLFPSFIHTQK;FNPFDLTK;FSTVGGESGSADTVR;IWPHADYPLIEVGK;LFSYSDTHR;LINAQGVAHWVK;VVENIVGHLK | 0.1782 | 25.6945 | 9 |
| Beta-actin | AGFAGDDAPR;AVFPSIVGRPR;CDVDIR;DLYANTVLSGGTTMYPGIADR;EITALAPSTMK;IWHHTFYNELR;SYELPDGQVITIGNER;TTGIVLDSGDGVSHTVPIYEGYALPHAILR;VAPEEHPVLLTEAPLNPK | 0.3564 | 13.8668 | 9 |
| V-type proton ATPase subunit H | DHDFIVNLDR;HIIEQLGGK;IVLAVFR;LNNNEYVQSVAR;LQTSVQDLSSFDQYATEVK;QLSILEQK;SDLLFYLSWLK;TLVHLLEK | 0.1772 | 21.8301 | 8 |
| V-type proton ATPase subunit C | DFSYNEADLAAGK;EHFILDSEYLTTLLVIVPK;LDTFVESVTR;NIADIISK;QFGPLVR;QTGSLLTR;VGTLDQLVGLSDDLGK;VVEEFK | 0.2272 | 22.9696 | 8 |
| Carboxylesterase 6 | ENFVGLIAK;GLPPLVSEAPAESLK;LGSLGYLSTGQR;SATGVAALSGAPLSPGAVRPDPAK;SGSTASYGGQHLVQK;VPLLTGVTSAETSR;VRENFVGLIAK | 0.1548 | 20.4794 | 7 |
| QM protein | DQFHIR;GAFGKPQGTVAR;IGQPIMSVR;YEREEFEK;YRPEHGPLDAWR | 0.2146 | 12.9123 | 7 |
| 40S ribosomal protein S7 | ASAAEPDVFETSISQALVELETNSDLK;ELYITK;IKLDGSQLIK;SIIIYVPMPK;VDTFQSVYK | 0.3263 | 15.4959 | 7 |
| 1,4-beta-N-acetylmuramidase | DWVCLVENESGR;FEAWYGWK;HKFEAWYGWK;MRDWVCLVENESGR;QGFPEDK;YWCSNTSTPGK | 0.2979 | 16.8163 | 6 |
| Ribosomal protein S2 (Fragment) | AFVAIGDNNGHIGLGVK;IDKLESIYLFSLPIK;LESIYLFSLPIK;LSVLPVR | 0.2868 | 13.9636 | 6 |
| 40S ribosomal protein S11 | DVELGDIVTIGECRPLSK;DYLHYLPK;EAIEGTYIDKK;NVGLGFK;RDYLHYLPK | 0.2961 | 13.2722 | 5 |
| Serine/threonine-protein phosphatase 5 | FVTELMEFYK;LENFGYALSDASK;NGLDYVIR;QSYNNAIELYSK | 0.0878 | 10.7564 | 4 |
| V-type proton ATPase subunit a | DLNPDVNAFQR;EVNQNAEALKR;IEDLNTVLGQTQDHR;SGSSVPPILNR | 0.0574 | 10.3239 | 4 |
| Heat-shock protein 21.4 | DGVLTVEAPLPQLAITDR;EFLLPK;ESNNFFK;LGDFSVIDTEFSSIR | 0.2460 | 10.9626 | 4 |
| Ribosomal protein S5 (Fragment) | LTNSLMMHGR;RQAVDVSPLR;VNQAIWLLCTGAR | 0.1618 | 8.4513 | 3 |

Table S20 Identified *Spodoptera exigua* larval coded proteins associated to HvAV-3h virions (Se-1)

| Protein ID | Identified peptide sequence | Sequence coverage | Protein Q score | Unique spectra |
| --- | --- | --- | --- | --- |
| Elongation factor 1-alpha (Fragment) | CLIEALDAILPPARPTDKPLR;EHALLAFTLGVK;EHALLAFTLGVKQLIVGVNK;EVSSYIK;EVSSYIKK;FEEIKK;IGGIGTVPVGR;IGYNPAAVAFVPISGWHGDNMLEASTK;KIGYNPAAVAFVPISGWHGDNMLEASTK;LPLQDVYK;LPLQDVYKIGGIGTVPVGR;MDSTEPPYSESR;MPWFK;QLIVGVNK;QLIVGVNKMDSTEPPYSESR;SGDAAIVNLVPSKPLCVESFQEFPPLGR;STEDNPK;STTTGHLIYK;TIEKFEK;VETGILKPGTIVVFAPANITTEVK;YAWVLDK;YYVTIIDAPGHR;YYVTIIDAPGHRDFIK | 0.5345 | 83.2308 | 50 |
| Moderately methionine rich storage protein A | AAIGLNTFYSDK;DHNLDVIR;DIYIIDENVYDK;DLSWYKPIK;DLSWYKPIKK;DPVFYMLQK;DTDDFNTNNFATMDIK;EDLYFPGVK;EVVNMFHMLYYAK;FEDNYER;FNNYVIAR;FVDVTIFHK;FVDVTIFHKK;GEIFTLHIDR;GFIEINGMR;GIILPPPYEIYPYYFVR;GYWPWLK;IDNVSVDK;IIDLVCLFK;IKDLSWYKPIK;KGLLDLK;KGYWPWLK;LCDFYGIK;LHNGIEFPVR;LLNHVMEPLMYK;LNFVELDTFLYK;LPSYTK;LPSYTKEDLYFPGVK;LRLPSYTK;LSNGLGR;LVDNVDMNVLDVNRK;LYLNEGMFVYALTVAVR;MPLGFPFDR;MPLGFPFDRR;NSYDMHNLVR;NYHTGFPTR;NYKFEDNYER;QLVILK;RIIDLVCLFK;RVESITDMR;SDDMEVLGK;STVLLDKMPLGFPFDR;TDKDIYIIDENVYDK;TDVVKDFLK;TPIESDAYFVDTDLNMK;TPIESDAYFVDTDLNMKVDR;VESITDMR;VESITDMRDFFIK;VHLNFEDK;VHLNFEDKLR;VTFDILSDK;YFVVPTILDQYQTALR;YFVVPTILDQYQTALRDPVFYMLQK;YLLIVMK | 0.5511 | 113.3326 | 48 |
| Vacuolar proton pump subunit B | AVVGEEALTPDDLLYLEFLTK;AVVQVFEGTSGIDAK;EHVLAVSR;FSEIVQLR;GFPGYMYTDLATIYER;GPPILAEDFLDIQGQPINPWSR;IPASILAEFYPR;IPIFSAAGLPHNEIAAQICR;IYPEEMIQTGISAIDVMNSIAR;LALTAAEFLAYQCEK;NFISQGNYENR;NGSITQIPILTMPNDDITHPIPDLTGYITEGQIYVDRQLHNR;NTLCEFTGDILR;QIYPPVNVLPSLSR;RIPASILAEFYPR;TLSAAQANKEHVLAVSR;TPVSEDMLGR;TVFESLDIGWQLLR;TVSGVNGPLVILDEVK;TVSGVNGPLVILDEVKFPK;VFNGSGKPIDK;VFNGSGKPIDKGPPILAEDFLDIQGQPINPWSR | 0.6113 | 92.2213 | 46 |
| H(+)-transporting two-sector ATPase | ALDDFYEK;ALDDFYEKNYAEFVPLR;ASLAETDKITLEVAK;DDFLQQNSYSSYDR;EILQEEEDLSEIVQLVGK;FGFVFAVSGPVVTAEK;GNEMSEVLR;HFPAINWLISYSK;IKADFDQLLEDMAAAFR;ITLEVAK;KHFPAINWLISYSK;LLKDDFLQQNSYSSYDR;LPANHPLLTGQR;NIIAFYDMSR;NYAEFVPLR;TALVANTSNMPVAAR;TGKPLSVELGPGILGSIFDGIQRPLK;TGKPLSVELGPGILGSIFDGIQRPLKDINELTQSIYIPK;TVVSQALSK;VGYNELVGEIIR;VKEILQEEEDLSEIVQLVGK;VTDVVLETEFDGEK;VTDVVLETEFDGEKEK;VTWNVIR;YTMLQVWPVR | 0.4140 | 99.6695 | 43 |
| Heat shock cognate 70 (Fragment) | ARFEELNADLFR;DAGTISGLNVLR;DNNLLGK;FEDATVQADMK;FEELNADLFR;FELTGIPPAPR;HWPFEVVSDGGKPK;IINEPTAAAIAYGLDK;IINEPTAAAIAYGLDKK;ISDSDKQTILDK;ITITNDK;ITITNDKGR;KFEDATVQADMK;LLQDFFNGK;LLQDFFNGKELNK;LSKEEIER;MKETAEAYLGK;MVNHFVQEFK;MVNHFVQEFKR;NTTIPTK;QTQTFTTYSDNQPGVLIQVFEGER;SINPDEAVAYGAAVQAAILHGDK;SQIHDIVLVGGSTR;STAGDTHLGGEDFDNR;TFFPEEVSSMVLTK;TVQNAVITVPAYFNDSQR | 0.5166 | 94.9553 | 43 |
| Arylphorin subunit B | DAYYPYELNVPSYFVQK;DDSVPINEIYEFLDQGK;DEAIALFHVLYFAK;DFDTFYK;DLHQYSYEIIAR;DPAFYQLYQR;DRLGEVFFYYYQQLLAR;EINYVGNYWHMNSDLYAEK;FYELDWFVQK;GENVFESYILDNKPFGYPFDRPVR;GTLGGYPFQLFVFVYPYNSVK;HVLGASPKPFDK;IHNEKNYEYIR;ILALFEHSK;KGENVFESYILDNKPFGYPFDRPVR;KILALFEHSK;KSSEFVLFK;LAAQYGMVK;LKPVEPQFVEYQK;LKPVEPQFVEYQKK;LNHKPFSVK;LPHGLGEIPEFSWYSNFK;LSVLKDR;LVPGENK;LVPGENKIER;MRDEAIALFHVLYFAK;NYEYIR;QAVDEFLLLYR;QLEYHADYYK;QPNVFFK;QYVKPYDHNDLHFVGVK;SDKDLHQYSYEIIAR;SDVASDAVFK;SGYYPQLYSVLAPYAQR;SSEFVLFK;SSEFVLFKDDSVPINEIYEFLDQGK;SYPMNYLVR;YTFMPSALDFYQTSLR;YYPFSIFYQR | 0.5601 | 88.9598 | 43 |
| UDP-glycosyltransferase | DSFGPAAAK;ELLDVFSK;ELSFIYHHR;FDGSLENVPK;GITLPTLYDLK;HLLEAGHEVTYVTAHAYK;IFVTHGGLLSTTETIHYGVPIIGIPLFADQFLNVK;ILVAFPYPGR;LKQTVIWK;LPASYAAIGGYHIEDEIPPLPK;LQDAIQEMLSNPQYR;QTVIWK;RGITLPTLYDLK;SETLPIEIQR;SHSILGEGYVR;SPALHVPVYQK;TLVHWVEHVIETNGAPHLR;VKELSFIYHHR | 0.3996 | 69.3255 | 32 |
| Translation elongation factor | AGIIAGAR;ALLELQLEAEELYQTFQR;AYLPVNESFGFTADLR;DLVFITNPDQR;DSVVAGFQWAAK;EEIDGLLTK;EGLPDLNQYLDKL;EGVMAEENLR;FKIDLVK;FYAFGR;GHVFEESQVAGTPMFVVK;GSVGFGSGLHGWAFTLK;GVQYLNEIK;GVQYLNEIKDSVVAGFQWAAK;IKPILFMNK;IMGPNFTPGK;LWGENFFNAK;NMSVIAHVDHGK;NPADLPK;QFSEMYADK;TGTITTFK;VFDAIMNFK;VNFTVDEIR;YLADKYEYDVTEAR | 0.2974 | 84.8146 | 31 |
| Methionine-rich storage protein | AANDPILMNYYGIK;DDLTYLDTDMLTR;DFDVFMR;DGILNGK;DGILNGKIER;DGTVISLK;DLGLSNNIK;DNMVNFDIK;DPVFWR;DTVGMISK;EDFDFPGVK;EWTIEDNVDK;EWTIEDNVDKYLK;FMHMMHLMK;FTDVMVYR;GEIFVHTNELHMIQAVK;GITLPAPYEIYPYFFVDSHVINK;ILYFAK;KDDLTYLDTDMLTR;KDLGLSNNIK;KPEDIEYLAR;KRPDMLMVAR;LDMVEIDTFK;LETGKNTIVR;LGGFPLQMYVIITPVR;LHTGDEMPVR;LLLPLGR;LLNHILQPTMYDDVR;LLSYNVYNFDK;LRDGILNGK;MLDDVER;MVLGGMGLVSDDAK;NLVVIDWR;NLVVIDWRK;NSVEMHGVIEQRPWTR;RDGTVISLK;RGEIMSYATMQLLAR;RLDMVEIDTFK;RPDMLMVAR;RVTDVFVLFK;SNNMILVTK;TNLLLPTVDMNLMK;TVDVESWWYK;VTDKNLVVIDWR;VTDVFVLFK;VTDVFVLFKK;VTVDVTSDKTVDAVVR;YTREDFDFPGVK | 0.5353 | 57.7570 | 31 |
| Arylphorin subunit A | ADTDGIVLPAPYEVYPEFFFNGK;DAYLPYELNVPSYFLQNK;DAYLPYELNVPSYFLQNKM;DDSVPINEIYEFLDQAK;DFDTFYK;DLHQYSYEIIAR;DPAFYQLYQR;DRLGEVFFYYYQQLLAR;EELQTYPVNYIVR;FSIFYER;FVEYQK;FYELDWFVQK;GDNVFESYILDNKPFGYPFDRPVR;GTLGGYPFQLFVFVYPYNSVK;HVLGASPKPFDK;IIDYLIDYK;KGDNVFESYILDNKPFGYPFDRPVR;KSSEFVLFK;LFEQNEQLDYHSDYYK;LNHKPFSVK;LPHGLGEIPEFSWYSNFK;LSVLKDR;LVPGENK;LVPGENKIER;MQSYPFEDKFAAQYGIVK;MRDEAIALFHVLYYAK;QAVDEFLLLYR;QPNVFFK;QYVKPYDHNDLHFVGVK;SGYNPQLYAHYTNYAQR;SNDYNLHNENNYEYIR;SNKDLHQYSYEIIAR;SSEFVLFK;SSEFVLFKDDSVPINEIYEFLDQAK;TFFQFLQK | 0.5615 | 70.2871 | 28 |
| Epoxide hydrolase | EELFYQPPAVLSAK;EFYEAIPLLTR;FDEAMIK;FSTWTK;FYAENFSHK;IMSLNLDQIPTDVPTWGLQAK;LDNNEWWGPK;NVEVLPLLMIHGWPGSVR;QDNAIRPFK;TLVGAFLPSLIIEEHLASR;TNIQGLDIHFIR;VKFDEAMIK;VKPEVPK | 0.3139 | 47.7546 | 28 |
| 60S ribosomal protein L40 | EGIPPDQQR;ESTLHLVLR;IQDKEGIPPDQQR;MQIFVK;MQIFVKTLTGK;TLSDYNIQKESTLHLVLR | 0.3256 | 21.9636 | 28 |
| Prophenoloxidase-1 | ADVSQLETWR;DLDRPVDQIR;DPVFYR;EDIGINLHHWHWHLVYPFDSADR;EVSNVVTSGVR;FQNFREPIEEGYFPK;FSDTTIPR;GAPLTFDEQR;GAPLTFDEQRR;GLDFTPR;IFMAPVQDER;LDFPGIR;LDSQVASR;LNPYGDDKLDFPGIR;MPVTIPVNYTANDSEPEQR;NTVGTHWELSTVELGR;QIPLDEER;RGELFYYMHQQIIAR;VSSVSIEGAAGR;WHSYIDDLFQLYK | 0.3173 | 73.5758 | 26 |
| Moderately methionine rich storage protein B | AVDCVVR;DFNTFIK;DPVFYMLQK;DVYIIDENIYDKR;EDLYFPGVK;FDNYVIAHDYNR;FTFDVLSDK;GEIFTLHIDR;GIILPPPYEIYPYYFVR;GYIEINGVR;GYWPWLK;HHNGVEFPVR;IDKLDVDR;IDVGNFFTPNMK;IIDLVCLFK;IKEFSWHK;KGYWPWLK;LCDEYER;LCDFYGIK;LIDINVNR;LLNHVMEPLMYK;LNFVELDTFLYK;LPSYTK;LPSYTKEDLYFPGVK;LRLPSYTK;LTNGKNTIVR;LVDNVDINVLDTDRK;LYLNEGMFVYALTVAVR;MPLGFPFDR;MPLGFPFDRR;NSYDMHNLVR;NYHTGFPTR;QLIILK;RIDVGNFFTPNMK;RIIDLVCLFK;STVLLDKMPLGFPFDR;VESITDMR;VESITDMRDFFIK;VYLCDEDKLR;YLLIVMK;YQDMETLGR | 0.4486 | 58.3371 | 24 |
| Peroxisomal membrane protein | DQIIYPQTR;EGSLDAVEDWMDVLSGGEK;ELTFEVR;EQLTLLASFYK;FKHYYTYGR;IALAASAALSAWVIR;ISNADQLLTTDIEK;LFYVPQRPYMTLGTLR;LITYSEEVAFYQGNHR;LSPEEQIQYMIK;NFLNFR;NSQNGSEIPAILAPGAGR;TVLDDVNK;TVLDDVNKGNYTR;VPLVTPNGDVLIK;WSIGELK | 0.2757 | 61.2189 | 21 |
| ATP synthase B | AITPDQEKQTLDR;ALEDAVEAEK;ALEDAVEAEKK;CIADLAALAAR;ENVLLQLEAAYR;EVDAIENDWNSSR;FGPSLAAWLDK;KENVLLQLEAAYR;KFGPSLAAWLDK;LGFIPEEWFQFFHSK;NFPRPVR;QMVDWIVSNVNK;RLDYQLEK;TGVTGPYTFGVGLTTYLFSK | 0.5533 | 56.3372 | 21 |
| Dihydrolipoyllysine-residue acetyltransferase | FVTFTASADHR;GIIELSK;GVPVGQLLCIIVGNEADVAAFK;ILIPAGTK;LGGQGSGLYGSLK;QTIPHYQLTATVNVEK;RLAEIR;RVPTVNSHWMDSFIR;VIDGAVGAQWMK;VNLPALSPTMESGSIINWQK;VPTVNSHWMDSFIR;VSMNDFIVK;VYASPMAR | 0.2922 | 49.4143 | 21 |
| NADPH--cytochrome P450 reductase | DMDLLWDVIGNR;EVGENVLYFGCR;GVTTTWLADNKPEPGKPLPR;LGQLTGANLDEIFSLINTDQESSK;LHSLQVQRPPYDAK;LPLQSQTPIIMVGPGTGLAPFR;LVTHGPDDIQPNNIFTGEIAR;NGEPDLSGLNYAVFGLGNK;NPFLAQITVNR;RLEELGATR;VFELGLGDDDANIEDDFITWK;VPVFIR;VYVTHLLEK;YEAGDHVAVYPINDSNLVER | 0.3193 | 57.1341 | 20 |
| UDP-glucuronosyltransferase | EALNFWVEHVVR;ELSTAYHDSPMKPK;EQGKPVPDYK;FEENLPNQPK;GLLEVFGGLK;HIGGYHIEDEVKPLPEDLKK;HIVPILK;HLLNAGHEVTYITPFASK;NGVIYFSLGSNLK;NLPDELKK;VDLTYNLHK | 0.2510 | 42.1959 | 20 |
| POX-C | DVSLSDYKGK;DYGVLNEETGIPFR;GLFIIDDK;GLFIIDDKQNLR;IGCEVIGASTDSHFTHLAWINTPR;LVQAFQYTDK;PLQLTKPAPQFK;QITVNDLPVGR;TTAVVNGEFK;TTAVVNGEFKDVSLSDYK | 0.5282 | 41.0321 | 20 |
| Heat shock protein 83 | ALLFVPR;APFDLFENK;APFDLFENKK;DQVANSSFVER;EKYTEDEELNK;ELISNSSDALDK;GVVDSEDLPLNISR;IVLHIK;KHLEINPDHSIVETLR;LADLLR;LGIHEDSQNR;NADDITQEEYGDFYK;RAPFDLFENK;SLTNDWEDHLAVK;TLVSVTK;YESLTDPSK;YTEDEELNK;YYEQFSK | 0.2162 | 60.4808 | 19 |
| Catalase | AAELASSDPDYSIR;DAAGFIQER;DAALFPSFIHTQK;DLYNAIAK;FSTVGGESGSADTVR;FSTVGGESGSADTVRDPR;GAGAFGYFEVTHDITK;IFTQVHPDLGSK;LFSYSDTHR;LGANFLQIPVNCPFR;NGPILLQDVNFLDEISSFDR;NGPILLQDVNFLDEISSFDRER;SLKDSPAYITTK;TVENIVGHLK;VAAGLAPFKK;VWPHSEYPLIPVGK;YNLTGDVDR;YNLTGDVDRYDSGQTEDNFSQATILYK | 0.4122 | 63.9471 | 19 |
| Cytochrome b-c1 complex subunit Rieske, mitochondrial | GKPLFIR;GPAPLNLEVPPHSFVEDGLLVVG;HRTENEISTEQAVPVDTLR;KGPAPLNLEVPPHSFVEDGLLVVG;QSFTYLIAGAGGVAGAYAAK;SIVTHFVSSMAAAADVLALAK;TENEISTEQAVPVDTLR;TENEISTEQAVPVDTLRDPQHDNQR | 0.3653 | 33.1916 | 19 |
| Tubulin alpha chain | AVCMLSNTTAIAEAWAR;AVFVDLEPTVVDEVR;DVNAAIATIK;EDAANNYAR;EIVDLVLDR;IHFPLVTYAPVISAEK;LIGQIVSSITASLR;LSVDYGKK;NLDIERPTYTNLNR;QLFHPEQLITGK;QLFHPEQLITGKEDAANNYAR;TVGGGDDSFNTFFSETGAGK;VGINYQPPTVVPGGDLAK | 0.3600 | 50.7864 | 18 |
| Cytochrome p450 CYP324A6 (Fragment) | DLLDALLK;FDAESLAK;FLPENANNIVPYSYLPFGEGPR;FSFWPK;GLVINDPELAR;GWQELYQAK;ILYDELVEAEK;LPYLDAVIK;LRGWQELYQAK;NTVAYFR;RGLVINDPELAR;SSPYVGIWVFWR;VEDLIYR;YFPNPEEFRPER | 0.2530 | 43.5990 | 17 |
| Acetyltransferase | AAQDAGVFK;DEVDNFALR;EGLVTAGTASGISDGAGAIVLAGEEAAK;GIFIVGAK;ITAHLVHELR;ITAHLVHELRR;LGAQFGITR;LGAQFGITRDEVDNFALR;LNVNGGATALGHPLGASGSR;LPPVFK;NTTATELQTAATVAALK;VEVDEHPRPQTTIEGLK;VEVDEHPRPQTTIEGLKK | 0.3409 | 45.3415 | 16 |
| NADH-cytochrome b5 reductase | DPPVTVTAK;FGLPSPQHVLGLPIGQHIHLSAK;GYVDLVIK;KDPPVTVTAK;KLNMIGTGIAPMLQLIR;KSSQLITLQDPNVK;LLFANQTEEDILLR;LLFANQTEEDILLRNELEK;LQYAGNGK;SYTPVSSDDEK;VWYTIDRPSEGWK;YALPLIER;YQSEHPEQFK | 0.4548 | 42.5319 | 16 |
| Fatty acid transport protein | ALLSYIK;DAVVYGVLVPNTEGR;DLSPSLK;DLTLPDIFHDVVNR;GENVSTTEVEAAISR;GNALIHSVNVAK;LGAITPLINTNQR;LLYIYTSGTTGLPK;VIYGNGLRPQIWK;VYPIAIIK;WRGENVSTTEVEAAISR;YLPLGVEEYDKIISGQIR;YVLATPPSPADTQHSVR | 0.2381 | 45.0809 | 16 |
| Tubulin (Fragment) | ALTVPELTQQMFDAK;FPGQLNADLR;FPGQLNADLRK;IMNTYSVVPSPK;LAVNMVPFPR;LHFFMPGFAPLTSR;YLTVAAIFR | 0.3128 | 29.1667 | 16 |
| Prophenoloxidase | ASELPTDADFSLFLPK;DPFFFR;DYTATDLEEEHR;EDIGVNLHHWHWHLVYPFTASQR;FTHLNNRPFR;FVQPLNAGQNTITR;IPIIIPR;LDSLTSSR;LSNVVEPNPR;NLVWSLADQRK;NVEEAIATGR;YLESFGVIADEATTMRDPFFFR | 0.2063 | 44.0566 | 15 |
| UDP-glycosyltransferase 40M3 | DLQDLIDNSK;ELSALYHDR;HGVIYFSMGSFLK;HLLEAGHDVTYITVYPFK;LTKPGPALAYWVEYVVR;NHPYQIQEFATEAAR;NVHIVNWAPQPSILAHPNVK;QTVIWK;VEELWSR;VPLSQNLPK;YIGGFHIEEPVKPLPK | 0.2692 | 37.3716 | 15 |
| Cytochrome p450 CYP4L7 | ANITSLDASTDVGIR;FAQEIVTGHEYISK;FEESLKNPFSFLAFSAGPR;GLLTATGQR;GTSVILNIYQIQR;HAFLDLLLLGEIDGKK;HFHILPVDEPPQLSADLILR;IQIPIFGPNAIFNLTPYK;KANITSLDASTDVGIR;LLSSWLGK;NPFSFLAFSAGPR;STEFLNLLAHYADK;YLELVIK | 0.3124 | 48.1861 | 14 |
| Activated C kinase 1 receptor | AMLWDLNDGK;DETNYGVPQK;DETNYGVPQKR;DVLSVAFSVDNR;EMVEELRPEIINQTQTSK;FSPNHANPIIVSAGWDR;GTLCGHNGWVTQIATNPK;LTRDETNYGVPQK;LWNTLAECK;TLIVWK;VWQVSVSAR;YPDMILSSSR | 0.3856 | 41.9442 | 14 |
| UDP-glucuronosyltransferase | DLNQENLLNAFK;DLNQENLLNAFKK;FLLLYHNFVLTGSR;LFPSNVIEVGGFHVIDAKPLTGDLK;LGIPYNPAYVPFHFLEGGTKPTLYQR;NWLPQVDILAHPK;WEDKTLLVDK;YFDDIPPLDDLAR | 0.2218 | 33.0790 | 14 |
| 60S ribosomal protein L7a | HWGGGVLGNK;KIVNPLFEK;KTCTCVAITHVESGDR;KVAAAPLVVK;LGALVHR;LKVPPPINQFTQTLDK;NFAIGQDIQPTR;TNFNERYEELR;VAAAPLVVK;VPPPINQFTQTLDK;YRPETEAVR | 0.3731 | 33.9881 | 13 |
| Cytochrome p450 CYP6AE97 | AVWPSIFYR;DFYYANGR;EDHITGDSISNVK;ENNPFQLMADEIFESSR;KEDHITGDSISNVK;NDFVDLLLNLK;NDFVDLLLNLKK;NVPHADPLPILGNYGNYILQK;QNLTPLFSSLK;YPVLGNLTR | 0.1905 | 36.2615 | 13 |
| Acetyltransferase | FSEFVFLER;GFTTSLQFFR;GKIPVIYNIQLAFEK;IPVENVPEDEGEAAK;IPVIYNIQLAFEK;ISQGSSYGTEK;LTIYIK;TPPTLTSLLYGKPVNAHLYIER;WLHELFVVK | 0.2533 | 36.0982 | 13 |
| Heat shock protein 70 | ALQDAEVQR;DAGQISGLNVLR;DQGLDIRK;ESADPEEIR;LFEQAYK;LRELLAK;NAVITVPAYFNDSQR;NTTIPTK;QAVTNSGNTFYATK;SQVFSTAADGQTQVEIK;TATSTLQQASLK;TTPSHVAFSK;VQSTVQEIFGR | 0.2012 | 42.1062 | 12 |
| Cytochrome p450 CYP4S8 | AQNKGEAIADVTDGIGTK;FWLHNDFIFNNSSR;GEAIADVTDGIGTK;ILTPTFHFNILK;LGDLLVK;LYPSVPFIGR;SAPYDFLK;SFADNVIMDR;VLHISGPPDVETVLSHSK;YLEAVIK | 0.2110 | 36.5406 | 12 |
| Actin | AGFAGDDAPR;AVFPSIVGRPR;DSYVGDEAQSK;DSYVGDEAQSKR;GYSFTTTAER;IIAPPER;RGILTLK;SYELPDGQVITIGNER;VAPEEHPVLLTEAPLNPK | 0.2400 | 32.3601 | 12 |
| Cytochrome p450 CYP354A14 | DFDHFTDR;FGGIFEGR;KNDFLQILVDFSENER;KPVIFVGNLGPR;LAMVDLVK;QSFHEFQLEVYNYFK;RPLLTILDPDLIK;STLTPVFSSSR | 0.1774 | 28.6764 | 12 |
| DNA topoisomerase I | AVALYFIDK;FYYDGK;GPVFAPDYEPLPENVK;HDNTVTWLASWTENVQGQAK;IEPPGLFR;LSQDAEEVAGFYAR;SSSSSSSKDK;TIALGTSK;TYNASITLQR | 0.1086 | 28.4008 | 11 |
| 14-3-3 protein epsilon (Fragment) | AAFDDAIAELDTLSEESYK;DICSDILGVLDK;DSTLIMQLLR;EAAENSLVAYK;HLIPSSQTGESK;IISSIEQKEETK;NLLSVAYK;NVSDNELTVEER;YLAEFATGNDRK | 0.4865 | 37.3720 | 11 |
| 60S ribosomal protein L6 | LDLGDFK;LDLGDFKLPEHLNDEYFK;LPEHLNDEYFK;LPEHLNDEYFKK;NYDLGNGVVR;SFYPTQDKIR;TRPTLTPGTVCILLAGR;YVIGTSTK | 0.2353 | 27.9449 | 11 |
| Polycalin | GVANFNAADYQGTWIENAR;GVSGFQAAR;NFIGTWYDVGR;TISDTPGLTK;VGSWILSK;VSSWVLSK;VWQLSR;WYELAR;YLGDWK | 0.0746 | 29.8134 | 9 |
| Acetyl-CoA carboxylase | ITDIIGK;ITSENPDEGFKPSSGTVQELNFR;LDPELQR;LGPLGSVK;TLQQAGQVWFPDSAYK;TTLIEDEGESR;VLIANNGIGAVK;VTWILR;WLSFVPK | 0.0407 | 29.5624 | 9 |
| Alpha-1,4 glucan phosphorylase | DYYFALAHTVR;EIWGVEPSWEK;ILIDIEK;LASLIER;LIFLENYR;SGFFSPGEPGR;SPVDFNLK;VAIQLNDTHPALAIPELLR;VDDVEALQK | 0.1082 | 26.5139 | 9 |
| Ribosomal protein L10 | AQVIEALR;AQVIEALRR;DQFHIR;FKFPGR;GAFGKPQGTVAR;IFDLGKK;IGQPIMSVR;YRPEHGPLDAWR | 0.2785 | 22.9439 | 9 |
| UDP-glucuronosyltransferase | DGHEVTYVTPIEYK;ELSYIYHDRPVKPGAELR;HWVQHVVNTR;NGVIYFSMGSNLK;NPPPNLR;SPALLVPLYQR;TLEHENIQR;WAPQPSILAHPK | 0.1801 | 21.3310 | 9 |
| Riboflavin-binding hexamerin | AVPFSLLESAHK;AVPFSLLESAHKFEAVTLYNVLYSAK;DAIEQGYVLNHLGER;DPAFYMIWK;DYDSFYK;ELYDQVSVLVQHPR;EQLAVPQVAIEK;ERVNEHLFAYVLGVAIVNRPDTQGIYIPR;FEAVTLYNVLYSAK;FFLAPK;HTPEIVIPHME;IDDYER;INTHGHR;LHEVFPSYYNNGEILTTAQR;LVESYPSTYK;LVESYPSTYKWDHNVVIR;NIYFQDVLIHHK;QLTDDPLGFPVNRPLYPWQVEGVK;QVDLMILFFHLHEPNHPESWK;RVLGLFQLWQEK;SWSLEK;TAVYLR;VLGLFQLWQEK;VNEHLFAYVLGVAIVNRPDTQGIYIPR;VRDAIEQGYVLNHLGER;VSLGLTIVAR;VWDTANNVYYAFEK;YDSHGYEIPLHVNSHNFLQIDEFVHDLPAGESVIAR;YYNDFISLWK | 0.4710 | 16.1899 | 9 |
| Acetyltransferase | AFAEELVPVPVPQK;AFAEELVPVPVPQKR;GSLASLSASELGAVAVK;LLELDPAK;LNIKPIAR;QAVIFAGLPK;QQQDDYAISSYK;RGDTPYGGIQLIDGIVFDGLTDVYNK | 0.2275 | 28.8459 | 8 |
| Cytochrome p450 CYP6AE10 | AQELAIQDVDNYLQR;EVIEDYTFPTGLK;FLGEEK;IGDVIFDTAR;LYPALGVLTR;NDFVDLILK;QNLTPLFSSAK;VQFEPR;YFPEPEQYRPER | 0.1749 | 26.1848 | 8 |
| Cytochrome p450 CYP6AB61 | FTPAFSTGK;GTDPELVIRDPEIIK;HLVQTVLK;KVQEEVDTVLAK;LTYDAIK;NFVYLPFGEGPR;NIFFADGDLWR;VLIPIQAIHR;VVGFYR | 0.1761 | 25.4797 | 8 |
| Cytochrome p450 CYP332A1 | EILEYDDLLK;FEPAAPVK;FFSQPATEHIR;FINSTDSDNHDFTFLPFGEGPR;KLFHEVVAQR;LFHEVVAQR;SFWDVFYDLSK;TAIAQLITK | 0.1610 | 23.9579 | 8 |
| Glycogen [starch] synthase (Fragment) | AAEGYSDTPPR;DKINEFAR;FSVDEEAGKR;GADIFIEALAR;HVDVATVFTTHATLLGR;LSDLLDWR;NLGIYYR;WTFETAWEVANK | 0.1256 | 26.0506 | 8 |
| Elongation factor Tu | AHDNVEAAVYILSK;ELDKPFLLPVESVHSIPGR;EVDAFIPTPIR;INNNLSEEDR;INNNLSEEDRLK;LGDLTLGTGVITK;SPEIGSDAITQLLK;TLDEAQAGDQLGALVR | 0.2129 | 30.3537 | 8 |
| ADP-ribosylation factor | DAVLLIFANK;HYFQNTQGLIFVVDSNDRER;ILMVGLDAAGK;IRPLWR;LGEIVTTIPTIGFNVETVEYK;LGLHSLR;MLSEDELRDAVLLIFANK;NISFTVWDVGGQDK;TTILYK | 0.5659 | 30.6928 | 8 |
| Acetyltransferase | ASPHIWFER;ELQQEEISKR;FGDAFWNSSR;HNGFLGILQR;LFEYGR;QEILGPSPELNYK | 0.1211 | 20.3724 | 8 |
| Cytochrome p450 CYP6B68 | ALNLSLFPTAIQK;ANDGQVTYESIK;EGLGQNLFHADGETWAALR;FFENLVK;FTPIFTTGK;HDFMDLILALR;VFDETLR | 0.1551 | 22.4972 | 8 |
| UDP-glycosyltransferase 42C2 | DKLEAIIDAVSEIPQR;IVLNPEFR;NIFISNWLPQNEILAHPK;NVNMLLLYTNYVLSGPGLYPPNVR;SHFFVFAPYLK | 0.1516 | 20.3531 | 8 |
| REPAT32 | AEYNAIPLK;DLDHTEAIPSVTAGGVGFSYANIR;EVFFSDPGQQIIK;VKEVFFSDPGQQIIK | 0.4364 | 16.2151 | 8 |
| UDP-glucuronosyltransferase | AKEVSAAYHDNPIKPGVALNFWVEHVVR;DQGKPVPDYNLLK;FEENLPDQPK;GTVDLPDFK;NGVIYFSMGSNLK;NVHIVQWAPQQSILAHPNLK;QIVPILK;QTVIWK;VLGDFNR | 0.2156 | 21.1967 | 7 |
| Fatty acid transport protein | DTLLYIYTSGTTGFPK;EIQTEISDIPLFQYNSPDR;IFSTIYPLTLVK;TGEYVPLTQK;TLDLASLAK;VGAIGFLSR;VLLYTIFR | 0.1256 | 25.4285 | 7 |
| Glucose-6-phosphate isomerase | AVLHIALR;EFSGQVISGQWK;INEQAWSLLLNLAK;INFTEDR;INIQQLFK;IPTPNDGEILLDYSK;TFTTQETITNATSAK | 0.1421 | 24.0133 | 7 |
| Acetyl-CoA deacylase | AHVNYVVTEQGIAELFGK;AYELIK;GKPIIALVSVTK;MFSGFGGQVDFIR;SDAIPIFLQDIPK;VGAGVVTSR | 0.1488 | 21.2586 | 7 |
| Acetyltransferase | AEAQWVESVEEWRR;AQLPLWVR;HLVLADPWGFAERPPNAYEK;KYLNYVPDAER;RVNELDPALPITVLYGSR;VIGSALQPLNPLWAVR | 0.2071 | 22.4274 | 7 |
| Acetyltransferase | FFQDIYAETTK;KPFIHPESKPADSNISVQK;KSDTPLFR;LHLELVK;VHLESTGNSEILR;YLNALRPLLTTK | 0.1074 | 20.9955 | 7 |
| Cytochrome p450 CYP339A1 | ETVVAPLHDSVNK;KCLGQNIAETMLSLLTLR;LFATPAWSSLVK;LLFNEINK;LVPPIPVLTR;NQIYGGLLTAYGEEWHR | 0.1349 | 19.3913 | 7 |
| Cytochrome P450 SE-CYP9A21v4 (Fragment) | AVPILSDLIAIIRPK;DYNLGKPNDSAAR;ILSGISFAK;LDFNSIQSLK;LVQEIK;LVQEIKEHDEK;LWPPFVIIDR;STLSPAFTSSK | 0.1488 | 21.0523 | 7 |
| UDP-glucuronosyltransferase | APVIQISSLGAALDNYANVGASVHPFLYPAVTR;AVWWTEHVLR;FAADRIEVLVK;LTVEEFK;WNDDVLPGR | 0.1349 | 15.9620 | 7 |
| Cytochrome p450 CYP4M15 | AIYDIGSVFFDR;HPYAYIPFSAGPR;IIEELDEIFGDTDRPIK;ILTPAFHFNILK;IQDKIIEELDEIFGDTDRPIK;NDGPVLVNFVK | 0.1375 | 19.0745 | 7 |
| REPAT31 | AEYMGIPFK;DIDHTSGSATVTAGGVGFSFANIR;EVFYSDPGQQIIK;VKEVFYSDPGQQIIK | 0.4364 | 16.6319 | 7 |
| Fatty acyl-CoA reductase | ESTLPAAR;FHNPNTLELATK;FIFSEWK;IYEAPEDPEK;LYWVEYFK;SHPNTYTFTK | 0.1078 | 17.4904 | 6 |
| Growth blocking peptide binding protein (Fragment) | HEYYLEPYLK;ILPVWENGGVTFK;LDANVDNIGDR;NDVLVFFIINK;NDVLVFFIINKR;QVWGSTNSNESR | 0.4754 | 24.0282 | 6 |
| Cytochrome p450 CYP6AB31 | AMFPLIVER;FEADEFDANNK;ILITDFAHFFER;VENDINELVGEILR;VVGFFR;YVYLPFGGGPR | 0.1230 | 17.3584 | 6 |
| Cytochrome P450 9A9 | AFLFANLPEVAK;GGHWLNFRPR;GTGVWIPVFSIHR;KGTGVWIPVFSIHR;LAQEIK;SNDLAAGFATVTESAVGK;STLSPAFTSSK | 0.1325 | 17.5911 | 6 |
| Cytochrome P450 | LNVIPR;NIGVVIHDIYK;NLEVEPHR;NVPGPEPIPFFGNIK;SLNLSLFPK;VFDETLR | 0.1111 | 16.6620 | 6 |
| UDP-glucuronosyltransferase | AVQVLILNQFEK;KAVQVLILNQFEK;LPLDFWGR;SHSILGYGVVNR | 0.0635 | 14.7450 | 6 |
| Dihydrolipoamide acetyltransferase component of pyruvate dehydrogenase complex | IQVLPR;LTYMPIIIK;VDLSTVK;VLTTPSVR;VVPVSGFTK | 0.0818 | 14.0276 | 5 |
| Cytochrome p450 CYP306A1 | IASGVYELLENVEK;LATDPPSTK;LYASIIDR;TWNWFR;VLGLAPVK | 0.0835 | 15.3441 | 5 |
| Cytochrome p450 CYP321A9 | ALFEIFGK;FVFYSLSPK;HDFADIALAIQK;IYTSLGFK;LQVQAGLVHVLR;NFTVQLSK;NISIELIPR;NVQQVLSGDFQSFNHR | 0.1650 | 14.6565 | 5 |
| Microsomal prostaglandin E synthase 2 | IINDADTSNLQLTLFQYR;KVPILLAK;TAGEALETFQWFEK;VLVNTLSPNVYR;VPILLAK | 0.1340 | 18.2645 | 5 |
| Trehalose 6-phosphate synthase | ASAGGLVTAVAPVVIR;ATFIADHWK;LPFILK;NLLVELGGR;VVLLQISVPSR | 0.0617 | 12.4091 | 5 |
| Casp-7 | GMAIIFNHEHFDIHSLK;LDGGITLSR;TGTNVDSDNLSK;TNGGGDEGDALGSHNSSQPTR;VTVLNNLK | 0.2264 | 18.1282 | 5 |
| ABCC2 | ALEQVELK;FFDTNPSGR;ISIIPQEPVLFSASLR;QLVCLAR;SSLISALFR | 0.0365 | 12.6915 | 5 |
| Glutathione peroxidase | FDLFEK;IPTDVPTWGIK;QAIDGALLHK;QLWVLDR;VLDFWQK | 0.0600 | 12.0233 | 5 |
| UDP-glucuronosyltransferase | AIWWTEHVLR;GAYHTLVNIVEMQLK;HLTEELAR;IREFYTYYK;NAILTILNNDSFR;SVIYDQPQPPLER;VFSQLPYDVLWK;WDKDELPGR | 0.1718 | 15.2088 | 4 |
| Acetyltransferase | FNTPVENGPR;FTNFYK;SNRPSLSEENAIAVLER;YTYDLR | 0.1270 | 11.4857 | 4 |
| Cofilin | NAEYDQFLEDLQK;QIDVETVGER;YIQATDLSEASQEAVEEK;YVVFYIKDEK | 0.3446 | 15.7864 | 4 |
| Elongation factor 1 beta | APSASLPHALR;IQEFEDFVQSVDIAAFNKI;SWAAGASPLTAGGK;SYVSGYTPTQADFQVFQQVGK | 0.2915 | 14.7454 | 4 |
| UTP--glucose-1-phosphate uridylyltransferase | GGTLIQYEDKLR;LLASVHETR;LLEIAQVPK;SVIQVR | 0.0709 | 11.9767 | 4 |
| Small G protein | AVLCPVQPIKPR;GLKTVFDEAIR;WFPEVR;YLECSALTQK | 0.2031 | 9.7361 | 4 |

Table S21 Identified *Spodoptera exigua* larval coded proteins associated to HvAV-3h virions (Se-2)

| Protein ID | Identified peptide sequence | Sequence coverage | Protein Q score | Unique spectra |
| --- | --- | --- | --- | --- |
| Elongation factor 1-alpha (Fragment) | CLIEALDAILPPARPTDKPLR;EHALLAFTLGVK;EVSSYIK;EVSSYIKK;FEEIKK;IGGIGTVPVGR;IGYNPAAVAFVPISGWHGDNMLEASTK;KIGYNPAAVAFVPISGWHGDNMLEASTK;LPLQDVYK;LPLQDVYKIGGIGTVPVGR;MDSTEPPYSESR;QLIVGVNKMDSTEPPYSESR;SGDAAIVNLVPSKPLCVESFQEFPPLGR;STEDNPK;STTTGHLIYK;TIEKFEK;VETGILKPGTIVVFAPANITTEVK;YAWVLDK;YYVTIIDAPGHR;YYVTIIDAPGHRDFIK | 0.5222 | 81.1784 | 37 |
| H(+)-transporting two-sector ATPase | ALDDFYEK;ASLAETDKITLEVAK;DDFLQQNSYSSYDR;DINELTQSIYIPK;EILQEEEDLSEIVQLVGK;FGFVFAVSGPVVTAEK;GNEMSEVLR;GTVTYIAPAGNYK;HFPAINWLISYSK;IKADFDQLLEDMAAAFR;ITLEVAK;KHFPAINWLISYSK;LAEMPADSGYPAYLGAR;LLKDDFLQQNSYSSYDR;LPANHPLLTGQR;NIIAFYDMSR;TALVANTSNMPVAAR;TGKPLSVELGPGILGSIFDGIQRPLK;TGKPLSVELGPGILGSIFDGIQRPLKDINELTQSIYIPK;VGSHITGGDLYGIVHENTLVK;VGYNELVGEIIR;VTDVVLETEFDGEK;VTDVVLETEFDGEKEK;YSNSDVIIYVGCGER;YTMLQVWPVR | 0.4773 | 99.2626 | 36 |
| Vacuolar proton pump subunit B | AVVGEEALTPDDLLYLEFLTK;FSEIVQLR;GFPGYMYTDLATIYER;GPPILAEDFLDIQGQPINPWSR;HVLVILTDMSSYAEALR;IPASILAEFYPR;IPIFSAAGLPHNEIAAQICR;LALTAAEFLAYQCEK;NFISQGNYENR;NTLCEFTGDILR;QIYPPVNVLPSLSR;RGFPGYMYTDLATIYER;RIPASILAEFYPR;SGQVLEVSGTK;TLSAAQANKEHVLAVSR;TVSGVNGPLVILDEVK;TVSGVNGPLVILDEVKFPK;VFNGSGKPIDK;VFNGSGKPIDKGPPILAEDFLDIQGQPINPWSR | 0.4615 | 79.3575 | 35 |
| Heat shock cognate 70 (Fragment) | ARFEELNADLFR;DAGTISGLNVLR;FEELNADLFR;FELTGIPPAPR;HWPFEVVSDGGKPK;IINEPTAAAIAYGLDK;IINEPTAAAIAYGLDKK;ISDSDKQTILDK;LLQDFFNGK;LLQDFFNGKELNK;LSKEEIER;MVNHFVQEFK;MVNHFVQEFKR;QTQTFTTYSDNQPGVLIQVFEGER;SINPDEAVAYGAAVQAAILHGDK;SQIHDIVLVGGSTR;TFFPEEVSSMVLTK;TVQNAVITVPAYFNDSQR | 0.3957 | 66.7180 | 32 |
| Moderately methionine rich storage protein a | AAIGLNTFYSDK;DFDTFVK;DLSWYKPIKK;EDLYFPGVK;EVVNMFHMLYYAK;FEDNYER;FNNYVIAR;FVDVTIFHK;GEIFTLHIDR;GFIEINGMR;GIILPPPYEIYPYYFVR;GYWPWLK;IDNVSVDK;IIDLVCLFK;KGLLDLK;KGYWPWLK;LHNGIEFPVR;LIYGNIEK;LNFVELDTFLYK;LPSYTKEDLYFPGVK;LSNGLGR;MPLGFPFDRR;NSYDMHNLVR;NYHTGFPTR;NYKFEDNYER;STVLLDKMPLGFPFDR;TDKDIYIIDENVYDK;TDVVKDFLK;TPIESDAYFVDTDLNMK;TPIESDAYFVDTDLNMKVDR;VESITDMRDFFIK;VHLNFEDK;VHLNFEDKLR;VTFDILSDK;YFVVPTILDQYQTALR;YLLIVMK | 0.4290 | 80.3101 | 31 |
| Arylphorin subunit b | DAYYPYELNVPSYFVQK;DDSVPINEIYEFLDQGK;DFDTFYK;DLHQYSYEIIAR;DPAFYQLYQR;DRLGEVFFYYYQQLLAR;EINYVGNYWHMNSDLYAEK;FLDTYEK;FYELDWFVQK;GENVFESYILDNKPFGYPFDRPVR;GTLGGYPFQLFVFVYPYNSVK;HVLGASPKPFDK;IHNEKNYEYIR;ILALFEHSK;KGENVFESYILDNKPFGYPFDRPVR;KILALFEHSK;KSSEFVLFK;LGEVFFYYYQQLLAR;LKPVEPQFVEYQK;LKPVEPQFVEYQKK;LNHKPFSVK;LPHGLGEIPEFSWYSNFK;LSVLKDR;LVPGENKIER;MQNGLLNEK;MRDEAIALFHVLYFAK;NYEYIR;QAVDEFLLLYR;QLEYHADYYK;QYVKPYDHNDLHFVGVK;SDKDLHQYSYEIIAR;SDVASDAVFK;SGYYPQLYSVLAPYAQR;SSEFVLFK;SSEFVLFKDDSVPINEIYEFLDQGK;SYPMNYLVR;VGKDYDIEANIQNYSNK;VPYDMSVQPDNMPR;YTFMPSALDFYQTSLR | 0.5904 | 78.6451 | 31 |
| UDP-glycosyltransferase 40Q2 | DSFGPAAAK;ELLDVFSK;ELSFIYHHR;FDGSLENVPK;GITLPTLYDLK;HLLEAGHEVTYVTAHAYK;IFVTHGGLLSTTETIHYGVPIIGIPLFADQFLNVK;ILVAFPYPGR;LKQTVIWK;LPASYAAIGGYHIEDEIPPLPK;LQDAIQEMLSNPQYR;QTVIWK;SETLPIEIQR;SHSILGEGYVR;SPALHVPVYQK;TLVHWVEHVIETNGAPHLR;VKELSFIYHHR | 0.3977 | 64.1886 | 28 |
| Translation elongation factor 2 | ALLELQLEAEELYQTFQR;AYLPVNESFGFTADLR;DSVVAGFQWAAK;EGLPDLNQYLDKL;EGVMAEENLR;FKIDLVK;FYAFGR;GSVGFGSGLHGWAFTLK;GVQYLNEIK;GVQYLNEIKDSVVAGFQWAAK;IKPILFMNK;LWGENFFNAK;NPADLPK;SDPVVSYR;STLTDSLVSK;TGTITTFK;VFDAIMNFK;VNFTVDEIR | 0.2109 | 66.7799 | 25 |
| Arylphorin subunit a | ADTDGIVLPAPYEVYPEFFFNGK;DAYLPYELNVPSYFLQNK;DDSVPINEIYEFLDQAK;DFDTFYK;DLHQYSYEIIAR;DPAFYQLYQR;DRLGEVFFYYYQQLLAR;EELQTYPVNYIVR;FAAQYGIVK;FLDTYEK;FSIFYER;FYELDWFVQK;GDNVFESYILDNKPFGYPFDRPVR;GTLGGYPFQLFVFVYPYNSVK;HVLGASPKPFDK;IIDYLIDYK;IYHKDAYLPYELNVPSYFLQNK;KGDNVFESYILDNKPFGYPFDRPVR;KSSEFVLFK;LFEQNEQLDYHSDYYK;LGEVFFYYYQQLLAR;LNHKPFSVK;LPHGLGEIPEFSWYSNFK;LSVLKDR;LVPGENKIER;MRDEAIALFHVLYYAK;QAVDEFLLLYR;QYVKPYDHNDLHFVGVK;SGYNPQLYAHYTNYAQR;SNDYNLHNENNYEYIR;SSEFVLFK;SSEFVLFKDDSVPINEIYEFLDQAK;TFFQFLQK;VGKDYDIEANIQNYSNK;VPYDMSVQPDNMPR | 0.5847 | 63.2254 | 24 |
| Methionine-rich storage protein | AANDPILMNYYGIK;DDLTYLDTDMLTR;DFDVFMR;DGILNGK;DGILNGKIER;DGTVISLK;DLGLSNNIK;DNMVNFDIK;DPVFWR;EDFDFPGVK;EWTIEDNVDK;EWTIEDNVDKYLK;FTDVMVYR;GITLPAPYEIYPYFFVDSHVINK;KDDLTYLDTDMLTR;KDLGLSNNIK;KPEDIEYLAR;KRPDMLMVAR;LDMVEIDTFK;LETGKNTIVR;LGGFPLQMYVIITPVR;LHTGDEMPVR;LLLPLGR;LLNHILQPTMYDDVR;LLSYNVYNFDK;LRDGILNGK;MLDDVER;NLVVIDWR;NSVEMHGVIEQRPWTR;RDGTVISLK;RGEIMSYATMQLLAR;RLDMVEIDTFK;RLLSYNVYNFDK;RVTDVFVLFK;SNNMILVTK;TVDVESWWYK;VTDKNLVVIDWR;VTDVFVLFK;VTDVFVLFKK;VTVDVTSDK;VTVDVTSDKTVDAVVR | 0.4407 | 55.9130 | 24 |
| Epoxide hydrolase | EELFYQPPAVLSAK;EFYEAIPLLTR;FSTWTK;FYAENFSHK;IMSLNLDQIPTDVPTWGLQAK;LDNNEWWGPK;NVEVLPLLMIHGWPGSVR;QDNAIRPFK;TLVGAFLPSLIIEEHLASR;TNIQGLDIHFIR;VKFDEAMIK;VKPEVPK | 0.3139 | 46.0434 | 22 |
| Catalase | AAELASSDPDYSIR;DAALFPSFIHTQK;FSTVGGESGSADTVR;FSTVGGESGSADTVRDPR;FYTDDGNWDLVGNNTPIFFIR;GAGAFGYFEVTHDITK;IFETVGK;IFTQVHPDLGSK;LFSYSDTHR;LGANFLQIPVNCPFR;NGPILLQDVNFLDEISSFDR;SLKDSPAYITTK;TVENIVGHLK;VAAGLAPFK;VAAGLAPFKK;YNLTGDVDRYDSGQTEDNFSQATILYK | 0.4024 | 63.5099 | 21 |
| 60S ribosomal protein L40 | EGIPPDQQR;ESTLHLVLR;IQDKEGIPPDQQR;LIFAGKQLEDGR;MQIFVK;TITLEVEASDTIENVK;TLSDYNIQKESTLHLVLR | 0.5039 | 28.3525 | 21 |
| Moderately methionine rich storage protein b | AVDCVVR;DFNTFIK;DLEEIGK;EDLYFPGVK;FDNYVIAHDYNR;FTFDVLSDK;GEIFTLHIDR;GIILPPPYEIYPYYFVR;GYIEINGVR;GYWPWLK;HHNGVEFPVR;IDVGNFFTPNMK;IIDLVCLFK;IKEFSWHK;KGYWPWLK;LCDEYER;LNFVELDTFLYK;LPSYTKEDLYFPGVK;LVDNVDINVLDTDR;LVDNVDINVLDTDRK;MPLGFPFDRR;NSYDMHNLVR;NYHTGFPTR;RIDVGNFFTPNMK;STVLLDKMPLGFPFDR;VESITDMRDFFIK;VYLCDEDKLR;YLLIVMK;YQDMETLGR | 0.3324 | 50.8457 | 20 |
| ATP synthase b | ALEDAVEAEK;ALEDAVEAEKK;AQGQELLIQAK;CIADLAALAAR;ENVLLQLEAAYR;EVDAIENDWNSSR;FGPSLAAWLDK;KENVLLQLEAAYR;KFGPSLAAWLDK;LGFIPEEWFQFFHSK;NFPRPVR;QMVDWIVSNVNK;TGVTGPYTFGVGLTTYLFSK | 0.5123 | 55.9577 | 20 |
| Dihydrolipoyllysine-residue acetyltransferase | DFKDDSPPGAVKPPVK;FVTFTASADHR;GVPVGQLLCIIVGNEADVAAFK;ILIPAGTK;LGGQGSGLYGSLK;QTIPHYQLTATVNVEK;TAQAAAAPAASAPAPAAPAAAPAAAPAAAAAPPPAAPAAAPDSGR;VIDGAVGAQWMK;VPTVNSHWMDSFIR;VSMNDFIVK | 0.3300 | 40.5052 | 19 |
| POX-C | DVSLSDYK;DYGVLNEETGIPFR;GLFIIDDK;GLFIIDDKQNLR;IGCEVIGASTDSHFTHLAWINTPR;LVQAFQYTDK;PLQLTKPAPQFK;QITVNDLPVGR;TTAVVNGEFK;TTAVVNGEFKDVSLSDYK | 0.5179 | 42.7635 | 17 |
| Heat shock protein 83 | ALLFVPR;APFDLFENK;DQVANSSFVER;EKYTEDEELNK;GVVDSEDLPLNISR;HSQFIGYPIK;IVLHIK;KHLEINPDHSIVETLR;LADLLR;NADDITQEEYGDFYK;RAPFDLFENK;SLTNDWEDHLAVK;TLVSVTK;YTEDEELNK | 0.1757 | 48.4247 | 15 |
| Cytochrome b-c1 complex subunit Rieske, mitochondrial | GPAPLNLEVPPHSFVEDGLLVVG;HRTENEISTEQAVPVDTLR;KGPAPLNLEVPPHSFVEDGLLVVG;QSFTYLIAGAGGVAGAYAAK;SIVTHFVSSMAAAADVLALAK;TENEISTEQAVPVDTLR;TENEISTEQAVPVDTLRDPQHDNQR | 0.3395 | 29.7449 | 15 |
| Fatty acid transport protein | ALLSYIK;DAVVYGVLVPNTEGR;DLTLPDIFHDVVNR;GNALIHSVNVAK;ILWITR;LGAITPLINTNQR;LLYIYTSGTTGLPK;LYYLDLK;QYLGYVDK;VIYGNGLRPQIWK;VYPIAIIK;YLPLGVEEYDKIISGQIR;YVLATPPSPADTQHSVR | 0.2335 | 47.3753 | 14 |
| UDP-glucuronosyltransferase | EALNFWVEHVVR;GLLEVFGGLK;HIGGYHIEDEVKPLPEDLKK;HIVPILK;HLLNAGHEVTYITPFASK;NGVIYFSLGSNLK;VDLTYNLHK | 0.1705 | 29.7449 | 14 |
| Cytochrome p450 CYP4S8 | AQNKGEAIADVTDGIGTK;EITEDFK;FWLHNDFIFNNSSR;GWLGSGLLLSTGLHWHK;ILTPTFHFNILK;KGTTVDVHIYELHR;KILTPTFHFNILK;LYPSVPFIGR;SAPYDFLK;TTLSELVR;VLHISGPPDVETVLSHSK;YLEAVIK | 0.2718 | 40.4082 | 13 |
| NADH-cytochrome b5 reductase | DPPVTVTAK;FGLPSPQHVLGLPIGQHIHLSAK;GYVDLVIK;IDDDLVIR;KSSQLITLQDPNVK;LGYAESQR;LLFANQTEEDILLR;LLFANQTEEDILLRNELEK;SYTPVSSDDEK;SYTPVSSDDEKGYVDLVIK;VWYTIDRPSEGWK;YALPLIER | 0.3903 | 45.4544 | 13 |
| Tubulin alpha chain | AVFVDLEPTVVDEVR;EDAANNYAR;EIVDLVLDR;FDGALNVDLTEFQTNLVPYPR;LDHKFDLMYAK;LIGQIVSSITASLR;LSVDYGKK;QLFHPEQLITGK;QLFHPEQLITGKEDAANNYAR;TVGGGDDSFNTFFSETGAGK;VGINYQPPTVVPGGDLAK | 0.3044 | 43.5109 | 13 |
| NADPH--cytochrome P450 reductase | EVGENVLYFGCR;LGQLTGANLDEIFSLINTDQESSK;LVTHGPDDIQPNNIFTGEIAR;SLVVFYGSQTGTGEEFAGR;TALSHYVEITALPR;TYEHYNAVAIYLDR;VYVTHLLEK;YEAGDHVAVYPINDSNLVER | 0.1930 | 31.5129 | 13 |
| Prophenoloxidase | DPFFFR;DYTATDLEEEHR;EPIPEAYFPK;FVQPLNAGQNTITR;GLDFSDR;IPIIIPR;LAYWR;LSNVVEPNPR;MSTQSSVTIPFEQTFR;NVEEAIATGR;YVINVNNTGSAR | 0.1573 | 40.8737 | 12 |
| UDP-glycosyltransferase 40M3 | ELSALYHDR;HLLEAGHDVTYITVYPFK;LTKPGPALAYWVEYVVR;NHPYQIQEFATEAAR;QTVIWK;SPALHVPLYQR;TFYTQPR;VEELWSR;YIGGFHIEEPVKPLPK | 0.2038 | 28.0672 | 12 |
| Peroxisomal membrane protein (Fragment) | DQIIYPQTR;EQLTLLASFYK;FLAELSALWK;GFTYYQVSNLDNR;ISNADQLLTTDIEK;LITYSEEVAFYQGNHR;TVLDDVNKGNYTR;VPLVTPNGDVLIK;WSIGELK | 0.1580 | 33.2460 | 11 |
| UDP-glucuronosyltransferase | DLNQENLLNAFK;DLNQENLLNAFKK;FLLLYHNFVLTGSR;LFPSNVIEVGGFHVIDAKPLTGDLK;LFPSNVIEVGGFHVIDAKPLTGDLKK;LGIPYNPAYVPFHFLEGGTKPTLYQR;NWLPQVDILAHPK;TIFDFYFR | 0.1946 | 32.7505 | 11 |
| Cytochrome p450 CYP324A6 (Fragment) | DLLDALLK;FDAESLAK;GLVINDPELAR;GWQELYQAK;LPYLDAVIK;LRGWQELYQAK;LTSVFTGSK;NTVAYFR;VEDLIYR;YFPNPEEFRPER | 0.1660 | 35.3502 | 10 |
| Cytochrome p450 CYP6AE97 | AVWPSIFYR;EDHITGDSISNVK;EQVNPNTFFPFGSGPR;KEDHITGDSISNVK;NDFVDLLLNLK;NDFVDLLLNLKK;NVPHADPLPILGNYGNYILQK;QNLTPLFSSLK;TFLTQPLTNCIALK | 0.1829 | 36.7695 | 10 |
| Cytochrome p450 CYP4L7 | ANITSLDASTDVGIR;FAQEIVTGHEYISK;GTSVILNIYQIQR;IQIPIFGPNAIFNLTPYK;ITISEIVK;LLSSWLGK;NPFSFLAFSAGPR;STEFLNLLAHYADK;YLELVIK | 0.2231 | 37.1486 | 10 |
| Prophenoloxidase-1 | DLDRPVDQIR;EPIEEGYFPK;EVSNVVTSGVR;FSDTTIPR;GLDFTPR;LNPYGDDKLDFPGIR;NTVGTHWELSTVELGR;WHSYIDDLFQLYK | 0.1316 | 29.5670 | 10 |
| UDP-glucuronosyltransferase | DGHEVTYVTPIEYK;ELSYIYHDRPVKPGAELR;FEEELPNVPK;GFALQVK;HWVQHVVNTR;LEELWIR;SPALLVPLYQR;TLEHENIQR | 0.1648 | 28.8012 | 10 |
| 14-3-3 protein epsilon (Fragment) | DICSDILGVLDK;DSTLIMQLLR;EAAENSLVAYK;IISSIEQKEETK;NLLSVAYK;NVSDNELTVEER;VFYYK;YLAEFATGNDRK | 0.3694 | 29.2580 | 10 |
| Acetyltransferase | AAQDAGVFK;DEVDNFALR;EGLVTAGTASGISDGAGAIVLAGEEAAK;ITAHLVHELR;ITAHLVHELRR;LPPVFK;TAFGTFGGVFR;VEVDEHPRPQTTIEGLK | 0.2298 | 28.0099 | 8 |
| Ribosomal protein L10 | AQVIEALR;DQFHIR;FKFPGR;GAFGKPQGTVAR;IFDLGKK;LHPFHVIR;YRPEHGPLDAWR | 0.2694 | 20.4682 | 8 |
| Activated C kinase 1 receptor | AMLWDLNDGK;DETNYGVPQKR;DKTLIVWK;DVLSVAFSVDNR;FSPNHANPIIVSAGWDR;LYGHSHFLSDVVLSSDGNYALSGSWDK;TLIVWK | 0.2665 | 25.3267 | 8 |
| Actin | AGFAGDDAPR;AVFPSIVGRPR;DSYVGDEAQSKR;GYSFTTTAER;IIAPPER;IIAPPERK;VAPEEHPVLLTEAPLNPK | 0.1840 | 26.5296 | 8 |
| REPAT32 | AEYNAIPLK;AEYNAIPLKK;DLDHTEAIPSVTAGGVGFSYANIR;EVFFSDPGQQIIK;VKEVFFSDPGQQIIK | 0.4455 | 19.7319 | 8 |
| Polycalin | ANYYVLATDYK;GVSGFQLLNYVGQWQELR;NEGTGSILGSWK;NFIGTWYDVGR;TISDTPGLTK;VGSWILSK;YLGDWK | 0.0683 | 25.5480 | 7 |
| Acetyltransferase | FSEFVFLER;GFTTSLQFFR;GKIPVIYNIQLAFEK;IPVIYNIQLAFEK;ISQGSSYGTEK;TPPTLTSLLYGKPVNAHLYIER;WLHELFVVK | 0.1984 | 25.9187 | 7 |
| Acetyltransferase | AFAEELVPVPVPQK;GSLASLSASELGAVAVK;LLELDPAK;LNIKPIAR;QAVIFAGLPK;QQQDDYAISSYK;RGDTPYGGIQLIDGIVFDGLTDVYNK | 0.2251 | 25.7141 | 7 |
| UDP-glycosyltransferase 42C2 | DKLEAIIDAVSEIPQR;ILEDVFPIYR;IVLNPEFR;SHFFVFAPYLK;SIFHLYFVYLHK;TPANSVPLYQYR | 0.1358 | 26.3883 | 7 |
| UDP-glucuronosyltransferase | AIWWTEHVLR;HLTEELAR;ILGFFPLPSISHQVIFR;IREFYTYYK;NAILTILNNDSFR;SVIYDQPQPPLER;VFSQLPYDVLWK | 0.1583 | 22.2022 | 7 |
| Acetyltransferase | ASPHIWFER;ELQQEEISKR;FGDAFWNSSR;HNGFLGILQR;LYVNILLK;SWNLLTR | 0.1127 | 18.6564 | 7 |
| UDP-glucuronosyltransferase | DVDGAEAFK;LPLDFWGR;NFLEEPSVVK;SHSILGYGVVNR;WAPQQSILAHPNLK | 0.1019 | 15.7508 | 7 |
| Cytochrome p450 CYP354A14 | EVDYDLLAK;FGGIFEGR;KPVIFVGNLGPR;RPLLTILDPDLIK;STLTPVFSSSR | 0.1033 | 18.4050 | 7 |
| 60S ribosomal protein L7a | HWGGGVLGNK;KVAAAPLVVK;LGALVHR;VAAAPLVVK;YRPETEAVR | 0.1343 | 16.5059 | 7 |
| Tubulin (Fragment) | ALTVPELTQQMFDAK;FPGQLNADLR;LAVNMVPFPR;LHFFMPGFAPLTSR;YLTVAAIFR | 0.2555 | 19.5564 | 7 |
| Fatty acyl-CoA reductase | ESTLPAAR;FIFSEWK;IYEAPEDPEK;LYWVEYFK;NVWNSLNR;SHPNTYTFTK | 0.1000 | 20.3199 | 6 |
| Acetyltransferase | EGFGIAYSAFPDK;FFQDIYAETTK;LHLELVK;LPIPELSK;VHLESTGNSEILR;YLNALRPLLTTK | 0.0982 | 21.0212 | 6 |
| Fatty acid transport protein | AILTFAGYADK;ETAPFVQGTTPLSVELK;TGEYVPLTQK;TLDLASLAK;VGAIGFLSR;VLLYTIFR | 0.0968 | 21.8039 | 6 |
| Heat shock protein 70 | ALQDAEVQR;DAGQISGLNVLR;QAVTNSGNTFYATK;SKLESLVGDLIK;TATSTLQQASLK;VQSTVQEIFGR | 0.1020 | 22.9131 | 6 |
| Growth blocking peptide binding protein (Fragment) | HEYYLEPYLK;ILPVWENGGVTFK;LDANVDNIGDR;NDVLVFFIINKR;QVWGSTNSNESR | 0.4754 | 20.9488 | 6 |
| Riboflavin-binding hexamerin | AVPFSLLESAHK;AVPFSLLESAHKFEAVTLYNVLYSAK;DAIEQGYVLNHLGER;DPAFYMIWK;DYDSFYK;ELYDQVSVLVQHPR;EQLAVPQVAIEK;ERVNEHLFAYVLGVAIVNRPDTQGIYIPR;FEAVTLYNVLYSAK;FFLAPK;HTPEIVIPHME;LHEVFPSYYNNGEILTTAQR;LSNGLGEVPELGHNIVK;NIYFQDVLIHHK;QLTDDPLGFPVNRPLYPWQVEGVK;QVDLMILFFHLHEPNHPESWK;RVLGLFQLWQEK;SWSLEK;TAVYLR;VADPVFLQR;VLGLFQLWQEK;VNEHLFAYVLGVAIVNRPDTQGIYIPR;VRDAIEQGYVLNHLGER;VSLGLTIVAR;VWDTANNVYYAFEK;WDHNVVIR;YDSHGYEIPLHVNSHNFLQIDEFVHDLPAGESVIAR;YYNDFISLWK | 0.4752 | 16.0934 | 6 |
| 60S ribosomal protein L6 | AVDDAVVK;GKSFYPTQDK;LDLGDFK;LPEHLNDEYFK | 0.1324 | 12.3881 | 6 |
| REPAT31 | AEYMGIPFK;DIDHTSGSATVTAGGVGFSFANIR;EVFYSDPGQQIIK;VKEVFYSDPGQQIIK | 0.4364 | 16.0046 | 6 |
| Cytochrome p450 CYP6AE10 | EVIEDYTFPTGLK;EVLTQNLFFSYGDR;LYPALGVLTR;NDFVDLILK;VQFEPR | 0.0989 | 13.0717 | 5 |
| Cytochrome p450 CYP6AE47 | AQSFITHPVGGIK;FNHLEPAALNLFATYGDNWR;IAAFVEPPTYLVYVR;ISNELEVR;NINPYVYLPFGEGPR | 0.1368 | 18.5483 | 5 |
| Casp-7 | DTHYKPENLWYYFTADK;GMAIIFNHEHFDIHSLK;LDGGITLSR;TETDGSPSASYR;TGTNVDSDNLSK | 0.2264 | 20.9488 | 5 |
| Cytochrome P450 SE-CYP9A21v4 (Fragment) | AVPILSDLIAIIRPK;LATDSFNLK;LDFNSIQSLK;LVQEIKEHDEK;STLSPAFTSSK | 0.1055 | 15.1584 | 5 |
| DNA topoisomerase I | AVALYFIDK;GPVFAPDYEPLPENVK;LSQDAEEVAGFYAR;TYNASITLQR | 0.0527 | 16.3753 | 5 |
| Acetyl-CoA deacylase | AHVNYVVTEQGIAELFGK;GKPIIALVSVTK;MFSGFGGQVDFIR;SDAIPIFLQDIPK | 0.1174 | 13.9524 | 4 |
| Dihydrolipoamide acetyltransferase component of pyruvate dehydrogenase complex | ELNTLQEK;IQVLPR;VDLSTVK;VEVLLEDR | 0.0608 | 12.1700 | 4 |
| Acetyltransferase | AEAQWVESVEEWRR;AQLPLWVR;SWVDNSSGQVLVEQR;VIGSALQPLNPLWAVR | 0.1262 | 17.5922 | 4 |
| Glutathione peroxidase | EFYELIPK;FDLFEK;FGPNTDPLELVPYLEK;QLWVLDR;VLDFWQK | 0.0644 | 12.5116 | 4 |
| NADH-ubiquinone oxidoreductase chain 1 | KVLGYIQIR;LMYLAWK;VGFMGILQPFSDAIK;VLGYIQIR | 0.0994 | 12.2888 | 4 |
| Glycerol-3-phosphate dehydrogenase [NAD(+)] (Fragment) | DIIQTEYFR;FPLFTAVFR;FVDVFYPGSK;VAEAFVK | 0.0992 | 12.1700 | 4 |

Table S22 Identified *Spodoptera exigua* larval coded proteins associated to HvAV-3h virions (Se-3)

| Protein ID | Identified peptide sequence | Sequence coverage | Protein Q score | Unique spectra |
| --- | --- | --- | --- | --- |
| Moderately methionine rich storage protein a | AAIGLNTFYSDK;DFDTFVK;DHNLDVIR;DLEDIGR;DLSWYKPIKK;DPVFYMLQK;EDLYFPGVK;EVVNMFHMLYYAK;FEDNYER;FNNYVIAR;FVDVTIFHK;FVDVTIFHKK;GEIFTLHIDR;GFIEINGMR;GIILPPPYEIYPYYFVR;GYWPWLK;IDNVSVDK;IIDLVCLFK;KGLLDLK;KGYWPWLK;LCDFYGIK;LHNGIEFPVR;LNFVELDTFLYK;LPSYTK;LPSYTKEDLYFPGVK;LSNGLGR;MPLGFPFDR;MPLGFPFDRR;NSYDMHNLVR;NYHTGFPTR;NYKFEDNYER;QLVILK;RIIDLVCLFK;SDDMEVLGK;STVLLDK;STVLLDKMPLGFPFDR;TDKDIYIIDENVYDK;TDVVKDFLK;TPIESDAYFVDTDLNMK;TPIESDAYFVDTDLNMKVDR;VESITDMRDFFIK;VHLNFEDK;VHLNFEDKLR;VTFDILSDK;YFVVPTILDQYQTALR;YLLIVMK | 0.4834 | 104.9253 | 44 |
| Elongation factor 1-alpha (Fragment) | CLIEALDAILPPARPTDKPLR;EHALLAFTLGVK;EVSSYIK;EVSSYIKK;FEEIKK;IGGIGTVPVGR;IGYNPAAVAFVPISGWHGDNMLEASTK;KIGYNPAAVAFVPISGWHGDNMLEASTK;LPLQDVYK;MDSTEPPYSESR;SGDAAIVNLVPSKPLCVESFQEFPPLGR;STTTGHLIYK;TIEKFEK;VETGILKPGTIVVFAPANITTEVK;YAWVLDK;YYVTIIDAPGHR;YYVTIIDAPGHRDFIK | 0.4852 | 70.4329 | 37 |
| H(+)-transporting two-sector ATPase | ALDDFYEK;ALDDFYEKNYAEFVPLR;ASLAETDKITLEVAK;DVSWEFNPLNVK;EILQEEEDLSEIVQLVGK;FGFVFAVSGPVVTAEK;GNEMSEVLR;GTVTYIAPAGNYK;HFPAINWLISYSK;IKADFDQLLEDMAAAFR;ITLEVAK;KHFPAINWLISYSK;LLKDDFLQQNSYSSYDR;LPANHPLLTGQR;NIIAFYDMSR;NYAEFVPLR;TALVANTSNMPVAAR;TGKPLSVELGPGILGSIFDGIQRPLK;VGSHITGGDLYGIVHENTLVK;VGYNELVGEIIR;VTDVVLETEFDGEKEK;YSNSDVIIYVGCGER;YTMLQVWPVR | 0.4627 | 98.7928 | 36 |
| Heat shock cognate 70 (Fragment) | ARFEELNADLFR;DAGTISGLNVLR;DNNLLGK;ETAEAYLGK;FEDATVQADMK;FEELNADLFR;FELTGIPPAPR;HWPFEVVSDGGKPK;IINEPTAAAIAYGLDK;IINEPTAAAIAYGLDKK;ISDSDKQTILDK;ITITNDKGR;KFEDATVQADMK;LLQDFFNGK;LLQDFFNGKELNK;LSKEEIER;MKETAEAYLGK;MVNHFVQEFK;MVNHFVQEFKR;NTTIPTK;QTQTFTTYSDNQPGVLIQVFEGER;SINPDEAVAYGAAVQAAILHGDK;SQIHDIVLVGGSTR;STAGDTHLGGEDFDNR;TFFPEEVSSMVLTK;TVQNAVITVPAYFNDSQR | 0.5166 | 98.0247 | 36 |
| Arylphorin subunit b | DAYYPYELNVPSYFVQK;DDSVPINEIYEFLDQGK;DFDTFYK;DLHQYSYEIIAR;DPAFYQLYQR;EINYVGNYWHMNSDLYAEK;FLDTYEK;FYELDWFVQK;GENVFESYILDNKPFGYPFDRPVR;GTLGGYPFQLFVFVYPYNSVK;HVLGASPKPFDK;IHNEKNYEYIR;ILALFEHSK;KGENVFESYILDNKPFGYPFDRPVR;KILALFEHSK;KSSEFVLFK;LGEVFFYYYQQLLAR;LKPVEPQFVEYQK;LNHKPFSVK;LPHGLGEIPEFSWYSNFK;LSVLKDR;LVPGENKIER;MRDEAIALFHVLYFAK;QAVDEFLLLYR;QLEYHADYYK;QYVKPYDHNDLHFVGVK;SDKDLHQYSYEIIAR;SDVASDAVFK;SGYYPQLYSVLAPYAQR;SSEFVLFK;SSEFVLFKDDSVPINEIYEFLDQGK;SYPMNYLVR;VGKDYDIEANIQNYSNK;VPYDMSVQPDNMPR;YTFMPSALDFYQTSLR;YYPFSIFYQR | 0.5919 | 79.6937 | 31 |
| Methionine-rich storage protein | AANDPILMNYYGIK;DDLTYLDTDMLTR;DFDVFMR;DGILNGK;DGILNGKIER;DGTVISLK;DLGLSNNIK;DNMVNFDIK;DPVFWR;DTVGMISK;EDFDFPGVK;EWTIEDNVDK;EWTIEDNVDKYLK;FTDVMVYR;GEIFVHTNELHMIQAVK;GITLPAPYEIYPYFFVDSHVINK;ILYFAK;KDLGLSNNIK;KPEDIEYLAR;KRPDMLMVAR;LDMVEIDTFK;LETGKNTIVR;LHTGDEMPVR;LLLPLGR;LLNHILQPTMYDDVR;LLSYNVYNFDK;LRDGILNGK;MLDDVER;NLVVIDWR;NSVEMHGVIEQRPWTR;RGEIMSYATMQLLAR;RLDMVEIDTFK;RLLSYNVYNFDK;RVTDVFVLFK;SNNMILVTK;TNLLLPTVDMNLMK;TVDVESWWYK;TVDVESWWYKTR;VTDKNLVVIDWR;VTDVFVLFK;VTDVFVLFKK;VTVDVTSDKTVDAVVR;YDCLGR | 0.4874 | 59.6681 | 31 |
| UDP-glycosyltransferase 40Q2 | DSFGPAAAK;ELLDVFSK;ELSFIYHHR;FDGSLENVPK;GITLPTLYDLK;HLLEAGHEVTYVTAHAYK;IFVTHGGLLSTTETIHYGVPIIGIPLFADQFLNVK;ILVAFPYPGR;LKQTVIWK;LPASYAAIGGYHIEDEIPPLPK;LQDAIQEMLSNPQYR;QTVIWK;RGITLPTLYDLK;SETLPIEIQR;SHSILGEGYVR;SPALHVPVYQK;TLVHWVEHVIETNGAPHLR;VKELSFIYHHR | 0.3996 | 71.7402 | 30 |
| Vacuolar proton pump subunit B | AVVGEEALTPDDLLYLEFLTK;FSEIVQLR;GPPILAEDFLDIQGQPINPWSR;IPASILAEFYPR;IPIFSAAGLPHNEIAAQICR;KDHSDVSNQLYACYAIGK;LALTAAEFLAYQCEK;NFISQGNYENR;NTLCEFTGDILR;QIYPPVNVLPSLSR;RIPASILAEFYPR;SGQVLEVSGTK;TVSGVNGPLVILDEVK;TVSGVNGPLVILDEVKFPK;VFNGSGKPIDK;VFNGSGKPIDKGPPILAEDFLDIQGQPINPWSR | 0.3947 | 72.6929 | 29 |
| Moderately methionine rich storage protein b | DFNTFIK;DLEEIGK;DPVFYMLQK;DVYIIDENIYDKR;EDLYFPGVK;EVVTMFHMLYYAK;FDNYVIAHDYNR;FTFDVLSDK;GEIFTLHIDR;GIILPPPYEIYPYYFVR;GYIEINGVR;GYWPWLK;HHNGVEFPVR;IDKLDVDR;IDNVNIDK;IDVGNFFTPNMK;IFFGPK;IIDLVCLFK;IKEFSWHK;KGYWPWLK;KVESITDMR;LCDEYER;LCDFYGIK;LNFVELDTFLYK;LPSYTK;LPSYTKEDLYFPGVK;LTNGKNTIVR;LVDNVDINVLDTDR;LVDNVDINVLDTDRK;MPLGFPFDR;MPLGFPFDRR;NSYDMHNLVR;NYHTGFPTR;QLIILK;RIDVGNFFTPNMK;RIIDLVCLFK;STVLLDK;STVLLDKMPLGFPFDR;VESITDMRDFFIK;VTVDSYR;VYLCDEDKLR;YLLIVMK;YQDMETLGR | 0.4433 | 81.6075 | 27 |
| Arylphorin subunit a | ADTDGIVLPAPYEVYPEFFFNGK;DAYLPYELNVPSYFLQNK;DDSVPINEIYEFLDQAK;DFDTFYK;DLHQYSYEIIAR;DPAFYQLYQR;EELQTYPVNYIVR;FAAQYGIVK;FLDTYEK;FSIFYER;FVEYQK;FYELDWFVQK;GDNVFESYILDNKPFGYPFDRPVR;GTLGGYPFQLFVFVYPYNSVK;HVLGASPKPFDK;IIDYLIDYK;KGDNVFESYILDNKPFGYPFDRPVR;KSSEFVLFK;LFEQNEQLDYHSDYYK;LGEVFFYYYQQLLAR;LNHKPFSVK;LPHGLGEIPEFSWYSNFK;LSVLKDR;LVPGENKIER;QAVDEFLLLYR;QYVKPYDHNDLHFVGVK;SDAASDAVFK;SGYNPQLYAHYTNYAQR;SNDYNLHNENNYEYIR;SNKDLHQYSYEIIAR;SSEFVLFK;SSEFVLFKDDSVPINEIYEFLDQAK;TFFQFLQK;VGKDYDIEANIQNYSNK;VPYDMSVQPDNMPR | 0.5832 | 71.8295 | 27 |
| Epoxide hydrolase | EAFLNKFPHFK;EELFYQPPAVLSAK;EFYEAIPLLTR;FSTWTK;FYAENFSHK;IMSLNLDQIPTDVPTWGLQAK;LDNNEWWGPK;NVEVLPLLMIHGWPGSVR;QDNAIRPFK;TLVGAFLPSLIIEEHLASR;TNIQGLDIHFIR;VKPEVPK | 0.3182 | 50.4888 | 27 |
| 60S ribosomal protein L40 | EGIPPDQQR;ESTLHLVLR;IQDKEGIPPDQQR;LIFAGKQLEDGR;MQIFVK;TITLEVEASDTIENVK;TLSDYNIQK;TLSDYNIQKESTLHLVLR | 0.5039 | 31.3310 | 27 |
| Translation elongation factor 2 | AGIIAGAR;ALLELQLEAEELYQTFQR;DLVFITNPDQR;DSVVAGFQWAAK;EGLPDLNQYLDKL;EGVMAEENLR;FKIDLVK;FSVSPVVR;FYAFGR;GGGQIIPTTR;GSVGFGSGLHGWAFTLK;GVQYLNEIK;GVQYLNEIKDSVVAGFQWAAK;IKPILFMNK;LWGENFFNAK;SDPVVSYR;STLTDSLVSK;VFDAIMNFK;VNFTVDEIR;YLADKYEYDVTEAR | 0.2346 | 78.7687 | 25 |
| Catalase | DAAGFIQER;DAALFPSFIHTQK;FSTVGGESGSADTVR;FSTVGGESGSADTVRDPR;FYTDDGNWDLVGNNTPIFFIR;GAGAFGYFEVTHDITK;IFETVGK;IFTQVHPDLGSK;LFSYSDTHR;LGANFLQIPVNCPFR;NGPILLQDVNFLDEISSFDR;NGPILLQDVNFLDEISSFDRER;QVFDDAQK;SLKDSPAYITTK;TVENIVGHLK;VSVSNYQR;YNLTGDVDR;YNLTGDVDRYDSGQTEDNFSQATILYK | 0.4083 | 71.5667 | 23 |
| ATP synthase b | AITPDQEK;ALEDAVEAEK;ALEDAVEAEKK;AQGQELLIQAK;CIADLAALAAR;ENVLLQLEAAYR;EVDAIENDWNSSR;FGPSLAAWLDK;KENVLLQLEAAYR;KFGPSLAAWLDK;LDYQLEK;LGFIPEEWFQFFHSK;QMVDWIVSNVNK;RLDYQLEK;TGVTGPYTFGVGLTTYLFSK | 0.5492 | 57.9355 | 21 |
| POX-C | DVSLSDYK;DYGVLNEETGIPFR;GLFIIDDK;GLFIIDDKQNLR;IGCEVIGASTDSHFTHLAWINTPR;LVQAFQYTDK;PLQLTKPAPQFK;QITVNDLPVGR;TTAVVNGEFK;TTAVVNGEFKDVSLSDYK;YGEVCPANWRPGAK | 0.5897 | 44.8184 | 20 |
| UDP-glucuronosyltransferase | EALNFWVEHVVR;ELSTAYHDSPMKPK;EQGKPVPDYK;GLLEVFGGLK;HIGGYHIEDEVKPLPEDLKK;HIVPILK;HLLNAGHEVTYITPFASK;NGVIYFSLGSNLK;NLPDELKK;VDLTYNLHK | 0.2318 | 39.9065 | 20 |
| Tubulin alpha chain | AVCMLSNTTAIAEAWAR;AVFVDLEPTVVDEVR;EDAANNYAR;EDLAALEK;EIVDLVLDR;FDGALNVDLTEFQTNLVPYPR;IHFPLVTYAPVISAEK;LIGQIVSSITASLR;LSVDYGK;LSVDYGKK;QLFHPEQLITGK;QLFHPEQLITGKEDAANNYAR;TVGGGDDSFNTFFSETGAGK;VGINYQPPTVVPGGDLAK | 0.3711 | 62.0267 | 19 |
| Heat shock protein 83 | ALLFVPR;APFDLFENK;DQVANSSFVER;EKYTEDEELNK;ELISNSSDALDK;GVVDSEDLPLNISR;HLEINPDHSIVETLR;HSQFIGYPIK;IVLHIK;KHLEINPDHSIVETLR;LADLLR;NILDNKVEK;SLTNDWEDHLAVK;TLVSVTK;YESLTDPSK;YYEQFSK | 0.2050 | 55.0779 | 18 |
| Fatty acid transport protein | ALLSYIK;DAVVYGVLVPNTEGR;DLTLPDIFHDVVNR;GENVSTTEVEAAISR;GNALIHSVNVAK;ILWITR;IQASNPAR;LGAITPLINTNQR;LLYIYTSGTTGLPK;LYYLDLK;QYLGYVDK;VIYGNGLRPQIWK;VYPIAIIK;WRGENVSTTEVEAAISR;YVLATPPSPADTQHSVR | 0.2442 | 57.3568 | 18 |
| Cytochrome p450 CYP4S8 | AQNKGEAIADVTDGIGTK;EITEDFK;FWLHNDFIFNNSSR;GTTVDVHIYELHR;GWLGSGLLLSTGLHWHK;ILTPTFHFNILK;KGTTVDVHIYELHR;LGDLLVK;LYPSVPFIGR;SAPYDFLK;SFADNVIMDR;TTLSELVR;VLHISGPPDVETVLSHSK;YLEAVIK | 0.3043 | 56.0938 | 18 |
| Cytochrome b-c1 complex subunit Rieske, mitochondrial | GPAPLNLEVPPHSFVEDGLLVVG;HRTENEISTEQAVPVDTLR;KGPAPLNLEVPPHSFVEDGLLVVG;QSFTYLIAGAGGVAGAYAAK;SIVTHFVSSMAAAADVLALAK;TENEISTEQAVPVDTLR;TENEISTEQAVPVDTLRDPQHDNQR | 0.3395 | 31.8031 | 18 |
| NADPH--cytochrome P450 reductase | EVGENVLYFGCR;GVTTTWLADNKPEPGKPLPR;LGQLTGANLDEIFSLINTDQESSK;LGQLTGANLDEIFSLINTDQESSKK;LHSLQVQRPPYDAK;LPLQSQTPIIMVGPGTGLAPFR;LVTHGPDDIQPNNIFTGEIAR;NGEPDLSGLNYAVFGLGNK;SLVVFYGSQTGTGEEFAGR;TALSHYVEITALPR;VYVTHLLEK;YEAGDHVAVYPINDSNLVER | 0.2830 | 54.5197 | 17 |
| Prophenoloxidase-1 | ADVSQLETWR;DLDRPVDQIR;DPVFYR;EPIEEGYFPK;EVSNVVTSGVR;FQNFREPIEEGYFPK;FSDTTIPR;GAPLTFDEQRR;LDFPGIR;LDSQVASR;LNPYGDDKLDFPGIR;MPVTIPVNYTANDSEPEQR;NTVGTHWELSTVELGR | 0.1886 | 48.5422 | 16 |
| Prophenoloxidase | ASELPTDADFSLFLPK;DLSVQGNDPR;DPFFFR;DYTATDLEEEHR;FMDSQVFQR;FVQPLNAGQNTITR;IPIIIPR;LSNVVEPNPR;MSTQSSVTIPFEQTFR;NLVWSLADQRK;NVEEAIATGR;YVINVNNTGSAR | 0.1919 | 47.0541 | 15 |
| Peroxisomal membrane protein (Fragment) | EQLTLLASFYK;FLAELSALWK;GFTYYQVSNLDNR;IALAASAALSAWVIR;ISNADQLLTTDIEK;LITYSEEVAFYQGNHR;LSPEEQIQYMIK;NSQNGSEIPAILAPGAGR;TNLTLYLYQQYLK;TVLDDVNKGNYTR;VPLVTPNGDVLIK;WSIGELK | 0.2310 | 50.5479 | 15 |
| Dihydrolipoyllysine-residue acetyltransferase | DFKDDSPPGAVKPPVK;FVTFTASADHR;GIIELSK;GVPVGQLLCIIVGNEADVAAFK;ILIPAGTK;LGGQGSGLYGSLK;QTIPHYQLTATVNVEK;VIDGAVGAQWMK;VPTVNSHWMDSFIR;VSMNDFIVK | 0.2545 | 39.4602 | 15 |
| 60S ribosomal protein L7a | HWGGGVLGNK;IVNPLFEK;KHWGGGVLGNK;KIVNPLFEK;KVAAAPLVVK;LGALVHR;LKVPPPINQFTQTLDK;NFAIGQDIQPTR;TNFNERYEELR;VAAAPLVVK;VPPPINQFTQTLDK;YRPETEAVR | 0.3172 | 46.2900 | 14 |
| UDP-glycosyltransferase 40M3 | AKELSALYHDR;ELSALYHDR;HLLEAGHDVTYITVYPFK;LTKPGPALAYWVEYVVR;NHPYQIQEFATEAAR;QTVIWK;SPALHVPLYQR;VEELWSR;VPLSQNLPK;YIGGFHIEEPVKPLPK | 0.2115 | 32.3940 | 14 |
| UDP-glucuronosyltransferase | DLNQENLLNAFK;DLNQENLLNAFKK;FLLLYHNFVLTGSR;LFPSNVIEVGGFHVIDAKPLTGDLK;LFPSNVIEVGGFHVIDAKPLTGDLKK;LGIPYNPAYVPFHFLEGGTKPTLYQR;TIFDFYFR;YFDDIPPLDDLAR | 0.1946 | 31.2437 | 14 |
| UDP-glucuronosyltransferase | DGHEVTYVTPIEYK;ELSYIYHDRPVKPGAELR;FEEELPNVPK;HWVQHVVNTR;LEELWIR;LIVPQIQK;NPPPNLR;QIDVSSNFNALPSDK;SPALLVPLYQR;TLEHENIQR;WAPQPSILAHPK | 0.2318 | 44.5442 | 13 |
| Acetyltransferase | FSEFVFLER;GFTTSLQFFR;GKIPVIYNIQLAFEK;IPVENVPEDEGEAAK;IPVIYNIQLAFEK;ISQGSSYGTEK;LTIYIK;MINLGILK;TPPTLTSLLYGKPVNAHLYIER;WLHELFVVK | 0.2742 | 42.4985 | 12 |
| Cytochrome p450 CYP324A6 (Fragment) | DLLDALLK;FDAESLAK;GWQELYQAK;ILYDELVEAEK;LPYLDAVIK;LRGWQELYQAK;LTSVFTGSK;RGLVINDPELAR;VEDLIYR;YFPNPEEFRPER | 0.1761 | 37.3719 | 12 |
| Activated C kinase 1 receptor | AMLWDLNDGK;DETNYGVPQK;DETNYGVPQKR;DKTLIVWK;DVLSVAFSVDNR;FSPNHANPIIVSAGWDR;IWDLESK;LWDLAAGK;LYGHSHFLSDVVLSSDGNYALSGSWDK;YPDMILSSSR | 0.3448 | 41.2589 | 12 |
| Tubulin (Fragment) | ALTVPELTQQMFDAK;FPGQLNADLR;FPGQLNADLRK;IMNTYSVVPSPK;LAVNMVPFPR;LHFFMPGFAPLTSR;YLTVAAIFR | 0.3128 | 31.8031 | 12 |
| Cytochrome p450 CYP4L7 | ANITSLDASTDVGIR;FAQEIVTGHEYISK;GTSVILNIYQIQR;IQIPIFGPNAIFNLTPYK;ITISEIVK;LLSSWLGK;LYPPVPIIER;NPFSFLAFSAGPR;STEFLNLLAHYADKYGK;YLELVIK | 0.2495 | 38.9334 | 11 |
| Actin | AGFAGDDAPR;AVFPSIVGRPR;DLTDYLMK;DSYVGDEAQSKR;GYSFTTTAER;IIAPPER;SYELPDGQVITIGNER;VAPEEHPVLLTEAPLNPK | 0.2453 | 33.8990 | 11 |
| Polycalin | ANYYVLATDYK;AVPGSTDGTGHLIVNFGGDR;GVANFNAADYQGTWIENAR;GVSGFQAAR;GVSGFQLLNYVGQWQELR;NFIGTWYDVGR;TISDTPGLTK;VGSWILSK;VTFNVNGEVR;VWQLSR | 0.1096 | 40.6000 | 10 |
| Acetyltransferase | ASPHIWFER;ELQQEEISKR;FGDAFWNSSR;GSFEVGGTIYPVAIK;HNGFLGILQR;LYVNILLK;QEILGPSPELNYK;SWNLLTR | 0.1712 | 34.7668 | 10 |
| NADH-cytochrome b5 reductase | DPPVTVTAK;FGLPSPQHVLGLPIGQHIHLSAK;GYVDLVIK;KSSQLITLQDPNVK;LGYAESQR;LLFANQTEEDILLRNELEK;VWYTIDRPSEGWK;YALPLIER | 0.3290 | 33.7254 | 10 |
| UDP-glycosyltransferase 42C2 | DKLEAIIDAVSEIPQR;IVLNPEFR;NIFISNWLPQNEILAHPK;NVNMLLLYTNYVLSGPGLYPPNVR;SHFFVFAPYLK;SIFHLYFVYLHK;TPANSVPLYQYR | 0.1988 | 31.8031 | 10 |
| Acetyltransferase | DEVDNFALR;EGLVTAGTASGISDGAGAIVLAGEEAAK;GIFIVGAK;ITAHLVHELRR;LNVNGGATALGHPLGASGSR;LPPVFK;VEVDEHPRPQTTIEGLK | 0.2500 | 27.6381 | 10 |
| Cytochrome p450 CYP354A14 | DFDHFTDR;EVDYDLLAK;FGGIFEGR;KPVIFVGNLGPR;QSFHEFQLEVYNYFK;RPLLTILDPDLIK;STLTPVFSSSR | 0.1481 | 27.1371 | 10 |
| Dihydrolipoamide acetyltransferase component of pyruvate dehydrogenase complex | ELNTLQEK;FDEEGNVR;IPHFGYSDEYDVSK;IQVLPR;TILEIAR;VDLSTVK;VEVLLEDR;VLTTPSVR;VVPVSGFTK | 0.1572 | 26.2635 | 9 |
| Cytochrome p450 CYP6AE97 | AVWPSIFYR;DFYYANGR;EDHITGDSISNVK;EQVNPNTFFPFGSGPR;KEDHITGDSISNVK;NDFVDLLLNLKK;NVPHADPLPILGNYGNYILQK;QNLTPLFSSLK;YPVLGNLTR | 0.1886 | 37.2651 | 9 |
| DNA topoisomerase I | AVALYFIDK;FYYDGK;IEPPGLFR;LNTQTLNEHLK;LSQDAEEVAGFYAR;QLDELTDASASIPEK;STFNNNFFNDWR;TIALGTSK;TYNASITLQR | 0.1000 | 35.2587 | 9 |
| Glutathione peroxidase | EFYELIPK;FDLFEK;FSAWTDVDNK;IPTDVPTWGIK;QAIDGALLHK;QLWVLDR;VLDFWQK;YYVQGGNYGAK | 0.1025 | 29.9343 | 8 |
| UDP-glucuronosyltransferase | AVQVLILNQFEK;AYYSIYPEIAAK;DVDGAEAFK;LPLDFWGR;SHSILGYGVVNR;SPALNVPLYQK;WAPQQSILAHPNLK | 0.1500 | 29.0208 | 8 |
| 60S ribosomal protein L6 | LDLGDFK;LDLGDFKLPEHLNDEYFK;LPEHLNDEYFK;LPEHLNDEYFKK;NYDLGNGVVR;SFYPTQDKIR;YVIGTSTK | 0.1728 | 28.4899 | 8 |
| UDP-glucuronosyltransferase | APVIQISSLGAALDNYANVGASVHPFLYPAVTR;AVWWTEHVLR;FAADRIEVLVK;ILAVFPIPSISHQVVFRPLTHELAK;LFITQAGLQSTDEAISAGVPLIAIPMFGDQWYNAEK;LTVEEFK | 0.2351 | 23.2289 | 8 |
| Acetyltransferase | EGFGIAYSAFPDK;FFQDIYAETTK;KPFIHPESKPADSNISVQK;LHLELVK;VHLESTGNSEILR;YLNALRPLLTTK | 0.1150 | 25.9427 | 8 |
| Ribosomal protein L10 | AQVIEALRR;DQFHIR;GAFGKPQGTVAR;IFDLGKK;LHPFHVIR;YRPEHGPLDAWR | 0.2466 | 17.9461 | 8 |
| Riboflavin-binding hexamerin | AVPFSLLESAHK;AVPFSLLESAHKFEAVTLYNVLYSAK;DPAFYMIWK;ELYDQVSVLVQHPR;EQLAVPQVAIEK;ERVNEHLFAYVLGVAIVNRPDTQGIYIPR;FEAVTLYNVLYSAK;HTPEIVIPHME;LHEVFPSYYNNGEILTTAQR;LSNGLGEVPELGHNIVK;NIYFQDVLIHHK;QLTDDPLGFPVNRPLYPWQVEGVK;QVDLMILFFHLHEPNHPESWK;RVLGLFQLWQEK;TAVYLR;VLGLFQLWQEK;VNEHLFAYVLGVAIVNRPDTQGIYIPR;VRDAIEQGYVLNHLGER;VSLGLTIVAR;WDHNVVIR;YDSHGYEIPLHVNSHNFLQIDEFVHDLPAGESVIAR;YYNDFISLWK | 0.4158 | 18.1732 | 8 |
| Fatty acyl-CoA reductase | ESTLPAAR;FHNPNTLELATK;FIFSEWK;IYEAPEDPEK;LQEFPK;NVWNSLNR;SHPNTYTFTK | 0.1196 | 22.2609 | 7 |
| Cytochrome p450 CYP6AE47 | AQSFITHPVGGIK;DFYYFNGR;FNHLEPAALNLFATYGDNWR;IAAFVEPPTYLVYVR;NINPYVYLPFGEGPR;YIGAFFGTEPVLIPK | 0.1657 | 23.0705 | 7 |
| Glycogen [starch] synthase (Fragment) | AAEGYSDTPPR;FSVDEEAGKR;GADIFIEALAR;LSDLLDWR;NLGIYYR;VGGIYTVIR | 0.0837 | 21.9611 | 7 |
| Growth blocking peptide binding protein (Fragment) | HEYYLEPYLK;ILPVWENGGVTFK;LDANVDNIGDR;LLWGHNGSVYNEYER;NDVLVFFIINKR;QVWGSTNSNESR | 0.5984 | 25.7946 | 7 |
| 14-3-3 protein epsilon (Fragment) | DICSDILGVLDK;EAAENSLVAYK;HLIPSSQTGESK;IISSIEQKEETK;NLLSVAYK;NVSDNELTVEER;YLAEFATGNDRK | 0.3559 | 27.2598 | 7 |
| Fatty acid transport protein | EIQTEISDIPLFQYNSPDR;IFSTIYPLTLVK;TGEYVPLTQK;TLDLASLAK;VGAIGFLSR;VLLYTIFR | 0.1014 | 23.9014 | 6 |
| Acetyl-CoA carboxylase | KVENPDDFNSAFR;LGPLGSVK;LLYGESPWGLSQIEFDEPK;QFPKFFTFR;VLIANNGIGAVK;VTWILR | 0.0281 | 19.3402 | 6 |
| Elongation factor Tu | EHLLLAK;ELDKPFLLPVESVHSIPGR;EVDAFIPTPIR;INNNLSEEDR;INNNLSEEDRLK;LGDLTLGTGVITK | 0.1333 | 21.8629 | 6 |
| Cytochrome p450 CYP4M15 | AIYDIGSVFFDR;EYTSQFDGIFR;HPYAYIPFSAGPR;IIEELDEIFGDTDRPIK;ILTPAFHFNILK;NDGPVLVNFVK | 0.1514 | 21.6751 | 6 |
| ADP-ribosylation factor | DAVLLIFANK;HYFQNTQGLIFVVDSNDRER;LGEIVTTIPTIGFNVETVEYK;LGLHSLR;NISFTVWDVGGQDK;TTILYK | 0.4286 | 22.7165 | 6 |
| REPAT31 | AEYMGIPFK;DIDHTSGSATVTAGGVGFSFANIR;EVFYSDPGQQIIK;VKEVFYSDPGQQIIK | 0.4364 | 18.1732 | 6 |
| REPAT32 | AEYNAIPLK;AEYNAIPLKK;DLDHTEAIPSVTAGGVGFSYANIR;VKEVFFSDPGQQIIK | 0.4455 | 16.4418 | 6 |
| UDP-glucuronosyltransferase | AIWWTEHVLR;EFYTYYK;HLTEELAR;ILGFFPLPSISHQVIFR;NAILTILNNDSFR;SVIYDQPQPPLER;VFSQLPYDVLWK | 0.1544 | 19.2787 | 5 |
| Acetyl-CoA deacylase | AYELIK;GKPIIALVSVTK;MFSGFGGQVDFIR;SDAIPIFLQDIPK;VGAGVVTSR | 0.1111 | 18.2965 | 5 |
| Acetyltransferase | AFAEELVPVPVPQK;LLELDPAK;LNIKPIAR;QAVIFAGLPK;QQQDDYAISSYK | 0.1232 | 20.6858 | 5 |
| Cytochrome p450 CYP6AE10 | AQELAIQDVDNYLQR;DYYFFSGR;LYPALGVLTR;NDFVDLILK;SIVTAPIGGIR | 0.1008 | 15.4561 | 5 |
| Elongation factor 1 beta' | APSASLPHALR;IQEFEDFVQSVDIAAFNKI;SYVSGYTPTQADFQVFQQVGK;TAQGLNELNNFLADK;WYNQIASYSAEER | 0.3543 | 21.2513 | 5 |
| Cytochrome P450 | FFENLVDNIITQR;NIGVVIHDIYK;NLEVEPHR;NVPGPEPIPFFGNIK;SLNLSLFPK;VFDETLR | 0.1250 | 20.3815 | 5 |
| Heat shock protein 70 | ALQDAEVQR;NAVITVPAYFNDSQR;NTTIPTK;QAVTNSGNTFYATK;SKLESLVGDLIK;TATSTLQQASLK | 0.1006 | 21.1369 | 5 |
| Cytochrome c oxidase subunit II | IMVTATDVIHSWTVPSLGVK;LLDVDNR;LLYLLDELNNPLITLK;NFINWINNYS | 0.2335 | 15.2428 | 5 |
| UDP-glucuronosyltransferase | DVPVILVSSLGAAPGNYDVIGAADHPLLYPSVLR;IQILVK;LHNLSLWEK;VFSQLPYDVLWK;VTELYSHYK | 0.1344 | 14.0597 | 4 |
| Cytochrome p450 CYP6B68 | ALNLSLFPTAIQK;FFENLVK;FTPIFTTGK;LNIVPR;VFDETLR | 0.0835 | 14.8274 | 4 |
| Cytochrome p450 CYP306A1 | LATDPPSTK;LYASIIDR;TWNWFR;VLGLAPVK | 0.0575 | 12.0995 | 4 |
| Microsomal prostaglandin E synthase 2 | EAPEYAAHYK;IINDADTSNLQLTLFQYR;KVPILLAK;VPILLAK | 0.0928 | 12.6111 | 4 |
| Serpin-2 | ALSSAIAQFSAK;DGSYDLQPSLK;LVLINALYFK;VQVTIPK | 0.1064 | 15.7258 | 4 |
| Alpha-1,4 glucan phosphorylase | DYYFALAHTVR;FAHIADVLLHHDR;LASLIER;SPVDFNLK | 0.0464 | 12.9041 | 4 |
| Glucose-6-phosphate isomerase | AVLHIALR;INFTEDR;LNPETALFIIASK;TWFLEAAK | 0.0647 | 12.3895 | 4 |
| Apolipophorin-III | DAPPANTLLQDIEK;EAYENFSK;LQAAVQNTAQEVQK;TFSEQLNSIANSK | 0.2606 | 16.1665 | 4 |
| Glutathione S-transferase S protein | AAIASYEKDEAVK;HLLVVPDLEK;LLLAYGDEGFKDHR;YAQSLSIAR | 0.2255 | 18.1732 | 4 |
| Glycerol-3-phosphate dehydrogenase [NAD(+)] (Fragment) | DIIQTEYFR;IKPTAAALSLIK;LTEIINETHENVK;VAEAFVK | 0.1161 | 16.0904 | 4 |

Table S23 Identified *Spodoptera frugiperda* larval coded proteins associated to HvAV-3h virions (Sf-1)

| Protein ID | Identified peptide sequence | Sequence coverage | Protein Q score | Unique spectra |
| --- | --- | --- | --- | --- |
| SFRICE_003260 (Fragment) | AAAGLLDLPNLPR;AAALEAFQADPCAGK;ADFELEAR;AEAQPFNNELDLDLYVK;AGTGYSIEASIQLVK;AHMLFIDAELAYPTSTGLPLK;APAYGADVDLYLNYEK;AQLLGEYVYSK;ASNTNLEKDIINYEGQVELK;AVYDKNGVEVFAK;DINFDSVAEGNVQK;DIVSADKSNLENYIDFK;DKHNIPISGQVVEEIGGGK;DTQVLQSSMNVETK;DVTQSNDKYSGHAEIVLSDAAK;ESFELLFDAEGAK;EVYLGVGALAGAFCR;FDIGAIETTTLYSSHAK;FEIYAVPLK;FEYSGQK;FLVDMDVTNEK;FLVDMDVTNEKK;FLVNTPLVK;FTLQDGR;GFDLQVGLPIDK;GFDLQVGLPIDKQEIVTASNDLVYFYAEK;GHFEYEPVMR;GKYDANELYGK;GNGMIIVDFK;GSPTTQSFLNGHK;GSYGDDTNFELAGVLQAK;GVETFFNMAIGER;HLEDALIDK;HNIPISGQVVEEIGGGK;HQSPPGIDKPIR;IDMSKPGVITNVVSAGFDLR;IDMSKPGVITNVVSAGFDLRK;IDVDVEGQYQDK;IHFSTNTKR;IKGSYGDDTNFELAGVLQAK;IVSTVLYVQPENK;KADFELEAR;KHQSPPGIDKPIR;KIDVDVEGQYQDK;KLEFSVHSK;KPSELTVVVK;KYSFSEEESFNIDALGLGGNIEK;LDIIGAATGR;LEFSVHSK;LEVATNIDIR;LFGTDAVFLSLGDDKGFDFDK;LGIAPEFGLMIK;LGIAPEFGLMIKDEVSDQR;LLGQVSVSSVGNCVHELAVQNLVISGPDGK;LLGQVSVSSVGNCVHELAVQNLVISGPDGKK;LLLEGSPTHLK;LLVPHVDPVVLDVNYK;LPQYTLDLHINK;LPQYTLDLHINKEDK;LVFFSLSNAR;MIIEGSGAVK;NDLVTGVFNPSAEIK;NDVNIEFQNR;NEIDLIYGGDLR;NGVEVFAK;NKFEIYAVPLK;NLLDTEPVHQVGNFISTSLR;NLLNTFSLK;NNNDKIHFSTNTK;NPSPDTYISNVVVDADGR;NVIYSQDSFLPR;NVLSGSTSYISK;NYQVEIASK;QEIVTASNDLVYFYAEK;QLDSEIR;QTAVAEFGFNHPK;RLEFHATNDNK;RVFLDGLIR;SAGSFAQLVR;SAVISMDAEVAPR;SGITIQLPQR;SKELGPIEQK;SNLENYIDFK;SSTQVDIFGTCPTEVSSSQEGSAVLVHR;TEMGALLAFNSPMK;TGPEICAEEGDTR;THGMIEIVLPTER;TILGEGNEK;TKVEEDNVVAVLK;TTNKDDLMNVYSHVK;VAATEEYLYRPFSVGENGAR;VEEDNVVAVLK;VENIRDINFDSVAEGNVQK;VFLDGLIR;VHGSVLHPQQNAIK;VIVNLHSSTGGHVVAK;VMALESVVSYPTNK;VTNLEYK;VVFGQMLK;VYKPIAEYQMPGEK;YDANELYGK;YDVTGNLLPKPVSLVASGTYVDSLTESDDKYR;YELSGVLLHK;YHMHVYNTPEFGNAK;YKPNDMNVGAIGHLK;YLDDFTVNVR;YLSSYPLTGK;YNYGVEGTVTVFLTGASNQETSVK;YSFSEEESFNIDALGLGGNIEK;YSGHAEIVLSDAAK;YTSPSTPKPSR | 0.5373 | 467.3881 | 156 |
| SFRICE_015484 (Fragment) | ALIPTPEIKDFTDALHSYVTK;AVIMLGDK;AVVVNGQHFFTFDGR;DFTDALHSYVTK;DLDNLEAR;DNFNYFYSALR;DNFNYFYSALRK;DNGQVTLNGANHGFPVIEK;EAVHQACDLAR;EIADDKVLK;EKYQELAIPEQALALLGDAYNTIR;EMPIFALLK;EYLDSLIEVVAR;FPYPIIYDTDQR;GLLGDGNNEPYDDFR;GLLGDGNNEPYDDFRLPNGK;HKPELEELTNALTAIFK;LLVAQIK;LSIVGHVQPEK;LSIVGHVQPEKR;LSTAITSLIQFIR;MLEEVLNSTKPLVDNILK;NFKDIVVPLVGQVLDGLK;NMVHGALFLDDSLNLK;NPAQIVGYTER;NVGESYELKVPNK;QADIVVAFETTK;QADIVVAFETTKDNEK;QFLSTAAAAINQR;RVDVSADLILGGEK;SFNPLDEVPAK;SYNDDLAK;VAALTSELGQSIK;VDVSADLILGGEK;VDVSADLILGGEKK;VNGKNPAQIVGYTER;VQFDDESR;YFKEYLDSLIEVVAR;YLYEVASIVDALR;YSALTADFYEK | 0.4249 | 165.5386 | 65 |
| SFRICE_011490 | ATHSYEEPVYYFVSDGQYK;DNKDIENVR;DSYVFSEPDENGNIVAQK;EGNTALPVGVLAVK;ESTTPELLGLK;FQPAQALADTSTLVPTTVYLVATAHAVVIR;FQPPHHIITLAQGQAHHITLR;GYDVSGSVTSFGSPIR;HDSLFIK;HFTIEFEPK;HNVAVTQDHTNVPTFEHTGYVLR;LEPGPGPHNPGLMHVLPR;LEPSQAVVQVK;LIGIQPR;LLALPTAPGQVAISYNVKPESVPFTVR;LLESLAAAAAK;LQCSLSK;LTQVTSEDGKYHFGPLDAAK;NAFEVTGFTVVGSVVK;NTVTTVGGDFYFTPVIPGK;QVTDGSVVVYTSNPTHYR;SVGGPGLSGAR;VCGAVTPPSAR;VHPPAGWSFEPSEVDLSVDGTTDR;VITAGQNQGPSGIAVQLINDK;VITAGQNQGPSGIAVQLINDKGDVR;VSSPVEVDDLVLHVVTDGAPPK;VSVGNGPVSGVTFSQLK;WKLEPSQAVVQVK | 0.3767 | 119.0061 | 39 |
| Dolichyl-diphosphooligosaccharide--protein glycosyltransferase subunit 2 | ALHFDGGLPATATLLAAIHR;AYKVELNVGGIAK;DATIADIYHAVFTLK;DKYDKPVLVQQAFVR;FAEYLLTR;FITSESDSIEFSISDLVGRPLR;GARPELAHTFR;GLAELNADIPNICETLK;GSVYDKEDALK;KDATIADIYHAVFTLK;KPSPLTEEQK;LLKPDEVLAQSGTR;LQADHLQK;LSMFDTAR;LTDKLQADHLQK;LTTVAYPNK;QPLTQKPNEPTTFVLNLSK;SQDITSLYYSVK;TTNVNVAVLGEIR;VASVAGDETIFVAEPDNTK;VELNVGGIAK;VSTVEVGVGDVDGTTSPK;YVAPLAALTFLAGHR | 0.4548 | 102.5296 | 38 |
| SFRICE_019251 | DFLAGGISAAVSK;DFLAGGISAAVSKTAVAPIER;EQGLLSFWR;GAFSNVLR;GFGVSVQGIIIYR;GIVDAFVR;GTGGAFVLVLYDEIKK;HTQFWR;IPKEQGLLSFWR;LAADVGKGDGQR;LLLQVQHVSK;LLLQVQHVSKQIAADQR;NTIHCWATIAK;QVFLGGVDKHTQFWR;TAVAPIER;TAVAPIERVK;TEGTGAFFK;YFAGNLASGGAAGATSLCFVYPLDFAR;YFPTQALNFAFK;YFPTQALNFAFKDK;YKGIVDAFVR;YKQVFLGGVDK | 0.6300 | 96.8533 | 38 |
| Heat shock 70 kDa protein cognate 4 (Fragment) | APAVGIDLGTTYSCVGVFQHGK;ARFEELNADLFR;DAGTISGLNVLR;FEELNADLFR;FELTGIPPAPR;HWPFEVVSDGGKPK;IINEPTAAAIAYGLDK;IINEPTAAAIAYGLDKK;ISDSDKQTILDK;ITITNDK;KFEDATVQADMK;LLQDFFNGK;LLQDFFNGKELNK;MKETAEAYLGK;MVNEAEKYR;MVNHFVQEFK;NQVAMNPNNTIFDAK;QTQTFTTYSDNQPGVLIQVFEGER;SINPDEAVAYGAAVQAAILHGDK;SQIHDIVLVGGSTR;STAGDTHLGGEDFDNR;TFFPEEVSSMVLTK;TVQNAVITVPAYFNDSQR;WLDSNQLADKEEYEHK | 0.4625 | 95.7655 | 37 |
| Mitochondrial 60 kDa heat shock protein-like protein | AAVEEGIVPGGGSALLR;AEQIRDQIQETTSEYEK;ALMLQGVDILADAVAVTMGPK;ATLSDMAIATGGVVFGDDANLIK;DGVITVKDGK;DKFQNIGAK;DQIQETTSEYEKEK;GANPIEIR;GVMLAVDAVK;GVMLAVDAVKDK;IGLQVAAVK;ISNVQTIIPALELANQQR;KISNVQTIIPALELANQQR;KPLVIVAEDVDGEALSTLVVNR;LENLQASDLGQVGEVVITK;LKIGLQVAAVK;LVQNVANNTNEEAGDGTTTATVLAR;RGVMLAVDAVK;TANSDQATGVEIVK;TANSDQATGVEIVKK;TLVDELEVIEGMK;VEFQDALVLFSEK;VEFQDALVLFSEKK;VNDALNATR | 0.4642 | 101.1073 | 35 |
| Protein disulfide-isomerase | DDDVIIAK;FDEDLFK;ILEFFGMK;ITSFPTIK;LGEHFEKDDDVIIAK;LIALEQDMAK;MDATANELEHTK;NFEEPSVK;NGDFDKYTEELKPVAK;NGSPIDYTGGR;QADDIITWLKK;QHLLSEDLPADWAAKPVK;SLAPEYAK;TFTSVASAVDDHVFALVSDPK;TGPPAVEVSSAEQAK;TLVATNFDEVVFDNSK;TLVATNFDEVVFDNSKK;TWVFVQSMPTIVEFSHETASK;VMSVAIDADEDEHQR;YDAEKFDEDLFK;YTEELKPVAK | 0.4575 | 89.0880 | 34 |
| ATP synthase subunit alpha | APGIIPR;DGQITPESDAALK;DGQITPESDAALKK;DNGKHALIIYDDLSK;EAYPGDVFYLHSR;EVAAFAQFGSDLDAATQQLLNR;GHLDKLDPSK;GIRPAINVGLSVSR;GMALNLEPDNVGVVVFGNDK;HALIIYDDLSK;IVTDFLATFTQAAQ;QGQYVPMAIEEQVAIIYCGVR;QMSLLLR;RTGAIVDVPVGEQLLGR;TALAIDTIINQQR;TGAIVDVPVGEQLLGR;TSHQGLLATIAK;VSVREPMQTGIK;VVDALGNAIDGK;VVDALGNAIDGKGPIDTK | 0.4111 | 86.3184 | 32 |
| H(+)-transporting two-sector ATPase | ALDDFYEK;ASLAETDKITLEVAK;DAMGNVLYQLSSMK;DDFLQQNSYSSYDR;DINELTQSIYIPK;EILQEEEDLSEIVQLVGK;GNEMSEVLR;GTVTYVAPSGNYK;HFPAINWLISYSK;LAEMPADSGYPAYLGAR;LLKDDFLQQNSYSSYDR;LPANHPLLTGQR;MSGSAMYELVR;NIIAFYDMSR;TALVANTSNMPVAAR;TGKPLSVELGPGILGSIFDGIQRPLK;TGKPLSVELGPGILGSIFDGIQRPLKDINELTQSIYIPK;TVVSQALSK;VGSHITGGDLYGIVHENTLVK;VTDVVLETEFDGEKEK;VTWNVIR;YTMLQVWPVR | 0.4448 | 88.2731 | 30 |
| Vacuolar proton pump subunit B (Fragment) | AVVGEEALTPDDLLYLEFLTK;AVVQVFEGTSGIDAK;GFPGYMYTDLATIYER;GPPILAEDFLDIQGQPINPWSR;HVLVILTDMSSYAEALR;IPASILAEFYPR;IPIFSAAGLPHNEIAAQICR;LALTAAEFLAYQCEK;NFISQGNYENR;NTLCEFTGDILR;QIYPPVNVLPSLSR;RIPASILAEFYPR;TVFESLDIGWQLLR;TVSGVNGPLVILDEVK;TVSGVNGPLVILDEVKFPK;VFNGSGKPIDK;VFNGSGKPIDKGPPILAEDFLDIQGQPINPWSR | 0.4731 | 76.8314 | 29 |
| SFRICE_024780 | AVVGPDGNVASNYGVIDPR;EIHLVIITTK;FGQIDLVK;FYPPEPAQLQEELTR;IQTDKELINTK;KLDASHGHVDVSR;KLEPTFAHVDSVK;LDASHGHVDVSR;LDLTNAHVDSVPGK;LRPGEFEQFESTVGFK;NLIDPTLALLDSER;QAVADPGVGFK;SGHILFTSGVIDPK;TDSSLAQQFSIVDKDAPK;TDSSLAQQLTVVDRDAPK;TGHLIVSSGVVDPK;TGNILITTAVVDPK;TGNILLLSGVVDPR;TGNLLITSGVVDPK;TGNLLLTSGVVDPQTGQVDPNLGQQISVVDKPK;TGNLLLTSSVVDPVSGHVDPTLGQQISVVDKPK;VDSSLAQQFAIVDKDR;VWVDLEK;YVNLVIVTSK | 0.1212 | 106.3630 | 28 |
| Dolichyl-diphosphooligosaccharide--protein glycosyltransferase subunit 1 | ALLPYPTAITQQEDQLVK;DAINSGATPLVTVETVFTK;IIDHIFDDMQVDEVVTK;IILPEGSTSVK;ITLENTGASPVK;KNVVENHIQDFQLR;LIEVSHWGNIAVEEVIEVEHTGAK;LVDISSQLVK;NNVAFVGAK;NVVENHIQDFQLR;SVETFTK;THYTLGYNVPSYEYLYQSGSDYLLK;VNLPYPVTR;YDYQQDHHSGPASVR;YIGNLYTYSPYYITSMK;YPLFGGWR | 0.4584 | 66.4411 | 28 |
| NADH-ubiquinone oxidoreductase 75 kDa subunit, mitochondrial | ALAQSLQKPVSGK;ALSEIIGER;AQQTLAAVSPPGK;ATYVNAEGR;AVEDKDVGPLIK;DLASGTSSHEVMK;FACDGLK;FASEVAGVDDFGTTGR;FEAPLLNAR;FTDIHFSGK;IDSVDVLDPLGSNIVVATR;LLAVAGDLADAESLVALK;LSQISPALVTYGNVEDNNYFAQAR;RIDSVDVLDPLGSNIVVATR;RPVVILGADQLK;TASQVAALDIGYKPGVGPVLQDGPK;TPEGAAILAYTQELALR;VDLTYDYTHVGDSASILK;VLNVLQR;VVYLLGADSGVVSR | 0.3630 | 86.9647 | 27 |
| 3-oxoacyl-[acyl-carrier-protein] reductase (Fragment) | AAAAPQQDLLHAVANILGIK;AAAAPQQDLLHAVANILGIKDPSK;AGGVEFR;ALLVEVAPHALLQAVLK;ALLVEVAPHALLQAVLKR;DAADALQHLLAAAGR;EPFRPDAPAYAAQVER;GFLWEVPR;GTPGLASHVR;GYDVVLGVQEIR;LAEETFAEAK;LGPVGGVFNLAAVLR;LRPSDVVYVEAHGTGTK;LSDANYHVNNLLSPVR;SEAAVAVLLQR;SGENVIEYDVSRPDDGFITGHNIDGR;TEEAVEALTSLAAR;TTELVGAAAGPHGAEALAAAAGSFYR;VLFPATGYLTLVWR;VLIDPAAQLAAVAAAGQPAQLDVR;WDHSLEWSVAHFGNASR;WVSSSLPR;YAGATELPAR;YIAAAAPLLR | 0.1337 | 99.5362 | 26 |
| SFRICE016243.2 (Fragment) | AKFEELNMDLFR;DAGAIAGLQVMR;DVDEIVLVGGSTR;EFFNGKEPSR;EWSDSTVQHDVK;FDLTGIPPAPR;FEELNMDLFR;IEIESFFEGEDFSETLTR;IFAPEEISAMVLTK;IINEPTAAAIAYGLDK;IINEPTAAAIAYGLDKK;ITPSYVAFTADGER;IVITNDQNR;LTPEDIER;LYQGQGGVPPPGAGGDDEDFKDEL;MKETAEAYLGK;NELESYAYSIK;NQLTTNPENTVFDAK;NQLTTNPENTVFDAKR;SLEDVVQPIIAK;SQIFSTASDNQHTVTIQVYEGERPMTK;TKMEEAIDAAIK;VTHAVVTVPAYFNDAQR;WLEDNQDVDSEEYKK | 0.4476 | 95.7986 | 26 |
| Heat shock protein 83 | ADLVNNLGTIAK;ALLFVPR;APFDLFENK;DQVANSSFVER;EDLTEYLEEHK;EGLELPEDEEEK;EGLELPEDEEEKK;EKYTEDEELNK;ELISNSSDALDK;ELISNSSDALDKIR;HIYYITGENR;HIYYITGENRDQVANSSFVER;HSQFIGYPIK;IVLHIKEDLTEYLEEHK;KHLEINPDHSIVETLR;LGIHEDSQNR;NADDITQEEYGDFYK;SLTNDWEDHLAVK;YESLTDPSK;YESLTDPSKLDSGK;YHTSASGDEACSLK;YTEDEELNK | 0.2734 | 89.1129 | 24 |
| Malate dehydrogenase | CFVGEINGVDPR;DDLFNVNAGIVR;DLTQCIAVHAPK;GLKGEENVVECAYVK;GPDELPSAIADSHVIVIPAGVPR;IALPELKK;IEVLTK;ILGVTTLDIVR;IPVIGGHSGVTIIPVLSQCKPAPQIQEQNK;LNAYEEK;MDPLVSTLALYDLAPITPGVASDLSHMDTPAK;RIQEAGTEVVK;VLGYKGPDELPSAIADSHVIVIPAGVPR;VMVAGAAGGIGQPLSLLLK;YFANPICFGK | 0.6246 | 57.5986 | 21 |
| SFRICE_001118 | EADYATPLYTPQNR;KTFGTTPVDEK;LDSESEISGVPPIHADGAGEAGPYTK;LPDDNGDPGLWVSYEDPDTAGQR;MVSLNENLDVDR;NPLLNADAVVNHWIQGGAPAHK;STWGSIWHGIK;TAFVNSGVLLAEQHGFDGIDLAWQFPK;TEGILSYPEVCAK;TFGTTPVDEKEAEHR;VLLSVGGDSDTEEAQKYNLLLESPQAR | 0.4401 | 49.6590 | 21 |
| Enoyl-CoA hydratase | DEFVTLSK;DKYLANLLPTLEYEK;ELEAVIPK;GVGQIQTGLANSVK;HAILATNTSAIPITK;HPGTSDDTAAAAVGVGLR;ILHSPLEGDVGAVFGLGFPPFTGGPFR;ILSTMLSEAVR;LGIVDLLVSPLGPGLGNPEENTMR;LPALTSVPTTLDLALTGK;MQLLEIIR;NMIFNK;TGFGLPEVMLGLLPGGGGTQR;VIGMHYFSPVDK;YLANLLPTLEYEK;YLETVAIQVAR | 0.1805 | 64.5568 | 20 |
| Voltage-dependent anion-selective channel protein 3 (Fragment) | ANDVFGK;DFNLHTNVDNGKDFGGSIYQK;GYHFGVFK;LRPGVVLTLSAAIDGQNFNAGGHK;NNFALGYQSK;SETGVEFSSGITSNQESGK;TKSETGVEFSSGITSNQESGK;VFGSLSSK;VTLEGTFAPQTGSK;WNTDNTLATDITIQDK;WTAGSADTLFGVGAK | 0.4417 | 44.1979 | 20 |
| SFRICE_000291 | AAQLGSAHEVQR;ALLEDAYK;DLSTVQTLLTK;DLTGVQNLLK;DVDETKDWIAEK;ILETAEDIQER;KHEALLSDLEAFGNTIK;LIEGQHYAADDVAQR;LLDSLQAQQLFR;LLQLQDQFR;LLVASDDYGR;LQEAAAQQQYNR;LQHLQQQWTQLQEK;LQQALVLVQFLR;LVLEGHPER;QIEELYLTFAK;TYIAAVK;YQQFER | 0.0811 | 72.1944 | 19 |
| Pyruvate carboxylase | AEELSFPK;AGGVVEAAISYTGDVSDPNKK;AGTHVLAIK;AGTVSSVAITNGQK;EANLLLGDIIK;EVFFELNGQLR;LDTNIALQTVSEYSAYWEQAR;LQVEHTITEEVTGIDLVQSQIR;QLIPNIPFQMLLR;SVFIRDENASK;TFLVGPTVGETIEVK;TVAVSDEMTAAGER;VFDSLNYLPNLILGMDAAGK;VISHAHDLPSSAAK;VVGDLAQFMVQNK | 0.2338 | 65.8826 | 19 |
| SFRICE_000735 | AGWERPAFFLPGEK;ATITDVTK;DGDYIGNEHVTR;DHDVDLYIK;FAGVPSLPLIPFK;GDHSFDFSK;GYHLWNSDLR;KYAFFTLDNK;NAAALFNMSYYGK;NYNIVFPHDEHLAGR;RGDYAFHLDKPIGIGYVTNK;VQQYDWGGAHSDYPR;VTWSGELGWELHVPSAHAIPVYK;VVYSLLLNDK;YTDAGLSNDAFPVNTHR | 0.2164 | 58.2333 | 19 |
| SFRICE_030637 (Fragment) | AFDEAASVSGRPTAIIAK;AFGLHSLVVDGHDVSELVK;APISEAPR;GHAAPILYAAWAEAGLFPVEELK;KLDSDLEGHPTPR;LAYGTALK;LAYGTALKK;LDNLVVIFDVNR;LDSDLEGHPTPR;LGQSEPTSLQHQLDVYDSR;LNFVDVGTGSLGQGLAVAAGMAYVGK;NFPGIEDLENWHGK;YFDQAPYR | 0.4062 | 56.6865 | 19 |
| SFRICE_010867 | ALEDAVEAEK;ALEDAVEAEKK;AQGQELLIEAK;CIADLAALAAR;ENVLLQLEAAYR;FGPSLAAWLDK;KENVLLQLEAAYR;LDYQLEK;LGFIPEEWFQFFHSK;LMNAYTEVK;LMNAYTEVKR;QMVDWIVSNVTK;TGVTGPYTFGVGLTTYLFSK | 0.4959 | 56.7590 | 19 |
| Adipocyte plasma membrane-associated protein (Fragment) | FTSIEIQSPQPR;GEVYTGLATGEIVK;KGQSEVFADGLPGTPDNARPLPDGSGILVALYTVFEEDRPPLTK;LFNSVALAK;LLGPEAFQLYK;LTPQGHVTFVTK;NGDLYWTDSSSDFELK;SLGHISDAIVFNGK;VETKPVQQPPVK;VGPLATNGALNNGGK;YYLSGPK | 0.2318 | 44.3725 | 19 |
| Heat shock 70 kDa protein cognate 5 | ALQDAEVQR;ASNGDAWVQGSDGK;EQQIVIQSSGGLSK;ERVEVSNQAEGVLHDTETK;ETAEAYLNTSVK;LFEQAYKK;NAVITVPAYFNDSQR;QAVTNSGNTFYATK;RFDDPEVQK;SDIGEVLLVGGMTR;SKLESLVGDLIK;SQVFSTAADGQTQVEIK;TATSTLQQASLK;VEVSNQAEGVLHDTETK;VINEPTAAALAYGMDK;VQSTVQEIFGR;VYSPSQIGAFVLMK | 0.3061 | 72.9444 | 18 |
| V-type proton ATPase subunit C (Fragment) | EFAYNEADLAAGK;EFAYNEADLAAGKNEITK;EHFILDSEYLTTLLVIVPK;FPIPDLK;GNLQNLEK;LDTFVEGVTR;NIADIISK;QFGPLVR;QVGQIDADLK;VAQYLGEVLEDQR;VAQYLGEVLEDQRDK;VFVESVLR;VGTLDQLVGLSDDLGK;VGTLDQLVGLSDDLGKLDTFVEGVTR;YGLPVNFQAVVMVPSR | 0.3198 | 59.8056 | 18 |
| 2-phospho-D-glycerate hydro-lyase | AAVPSGASTGVHEALELR;EIDDFMLK;FGLDSTAVGDEGGFAPNIQNNK;HLADLAGNADIVLPVPAFNVINGGSHAGNK;LAEVYLDFIK;LGANAILGVSLAVAK;MGSEVYHYLK;NGTYDLDFK;NGTYDLDFKNPK;NINDIIAPELLK;SNPADYLSSEK;TPIQIVGDDLTVTNPK;VNQIGSVTESIDAHLLAK | 0.4203 | 58.6635 | 18 |
| SFRICE_012231 | AGMYWLR;EQTVFYAK;IDAVTVQNVR;IPIHELDAR;IVLAGAGGVDHQK;LVDLANK;NNGVAHFLEHMAFK;RIPIHELDAR;SQGGGVNNASYLAR;SQTDLELLVENMGAHLNAYTSR;VASEDSGAATATVGLWIDAGSR;YTGSEIR | 0.2876 | 44.9152 | 18 |
| SFRICE_001000 (Fragment) | AEITPVTLTVK;DEVDNFALR;EGLVTAGTASGISDGAGAIVLAGEEAAK;ISVAGGVENMSQAPFAVR;ITAHLVHELR;ITAHLVHELRR;LNVNGGATALGHPLGASGSR;RDEVDNFALR;TAFGTFGGVFR;VEVDEHPRPQTTIEGLK;VEVDEHPRPQTTIEGLKK | 0.3265 | 50.6866 | 18 |
| P-type Ca(2+) transporter (Fragment) | EFDDLPVSEQR;EIVPGDVVEVSVGDK;EIVPGDVVEVSVGDKIPADIR;FSVPVVLLDEVLK;GAPEGVLDR;GLSPDQVKR;GVVIGTGLNTAIGK;IDQSILTGESVSVIK;KAEIGIAMGSGTAVAK;KEVFDSIVR;MNPFNVPK;NAESAIEALK;TEMSETEEIK;TGTLTTNQMSVSR;YGPNELPAEEGK | 0.1660 | 61.6653 | 17 |
| Citrate synthase | AELPAHVVTMLNNMPSK;ALGVLAQLIWSR;EMNYYTVMFGVSR;GLSAESTNLK;GLVWETSVLDPDEGIR;HLPNDPLFK;KQVGDNFTEEGLK;LHPMSQFSAAVTALNSESSFAK;LPVIAATIYR;LVAAVYK;NPWPNVDSHSGVLLQYYGLK;QVGDNFTEEGLKEFIWK;VVPPILTELGK | 0.3548 | 56.2119 | 17 |
| SFRICE_000152 (Fragment) | AAGAVGLAR;AVLSPDGK;IFEGTNDILR;IPIENVLGGIGNGFK;KLSEFGTIQEK;LAEAWVEEAVTR;LAELGGAVAAAGGQPTPNPLNL;LASSAIDAYTAAAVMSR;LFVALTGIQFAGSHLQELQR;LSEFGTIQEK;NPTAHLGLIFSEAGR;SFGGVSSGPPENK;TVDKVTAFIVER;VAMNILNNGR | 0.3234 | 58.9573 | 16 |
| SFRICE_003762 | AAVNQAYETTLK;AFAAGADIK;ALNALCGPLFVELGQAVR;AMEIVLTGNFIDATEAER;FGQPEINIGTIPGAGGTQR;IAAIIITGNEK;IGTHSPLIVK;KNVGLIQLNRPK;NVGLIQLNRPK;VFPVDK;VFPVDKLLEETIK | 0.4192 | 40.9176 | 16 |
| SFRICE_004571 | ATIEHEMELR;DAVAAIHK;DIAIATK;FSLLEAVK;IGKPTLVNETSR;IKEYEAAIEQAK;KGVLLFIDEADAFLR;KPSDALAGVVLAPTLER;LYFDTFVLQPASEGK;LYFDTFVLQPASEGKR;TTILEGIK | 0.1805 | 46.7681 | 16 |
| SFRICE_000420 | ADVDIAVK;AGTVWVNDYNVLSNQVPFGGYK;ANNSEYGLAAAVFTQDIDK;ANNSEYGLAAAVFTQDIDKANYVVQR;ENGPYGINNYTEVK;FSQLDEVVER;ILSLIESGK;ILSLIESGKK;ITLELGGK;LGSPWR;SPNIIFADTDLER;TENPATGQAIADVQQAGK;VVGDPFKPETEQGPQIDAEQQNR;YYAGWADK | 0.3261 | 57.4388 | 15 |
| SFRICE_007807 | ANIGVVPQDTVLFNNSVR;DAIGAPDLLVR;ELQLELFK;FYDVNEGAVLVDGQDVR;GAIVNYQAEEFK;ILTFPDAYDTQVGER;LNAPASDIISAAK;LVLSNISFK;SVDSLLNYETVK;TILKDPAIVLLDEATSALDTNTER;TTIIIAHR;VPPGSTVALVGPSGAGK;YNIQYGK | 0.2017 | 52.6241 | 15 |
| Isocitrate dehydrogenase [NADP] | AFAHSSFQVALQK;AKLDDTPELER;DATDDQVTIDAAHAILK;DLVICIHGLK;EGMYLHTEDFLLAIADQLER;IIWSMIK;IWYEHR;LDCLYYDLGLPHR;LDDTPELER;LIDDMVAQAIK;TVESEAAHGTVTR;VVPGWTQAMVIGR | 0.3088 | 37.1397 | 15 |
| ATP synthase subunit O, mitochondrial | APVQVFGLEGR;EFIVNPTLK;GEVTCEVVTAKPLDQTQR;LEAVINAFK;LKEFIVNPTLK;LSPTTSNLLGLMAENGR;QNLEAALK;QNLEAALKK;TLDAVEKELSQFQQSIK;YASALYSAATK | 0.4928 | 36.1861 | 15 |
| Peptidyl-prolyl cis-trans isomerase | DTNGSQFFITTTK;FEDENFK;FEDENFKLR;HYGAGWLSMANAGK;MGDENIGDIVIGLFGK;SIYGERFEDENFK;TEVVSEPFSVTK;TPWLDGR;TVENFVQLAQKPEGQGYK;VIDNFMIQGGDFTK | 0.5317 | 40.2464 | 15 |
| Calreticulin | FYALSR;FYNDPEEDKGLQTSEDAR;GPWVHPEIDNPEYTPDANLYK;GPWVHPEIDNPEYTPDANLYKR;HEQDIDCGGGYLK;KPEDWDDRPTIPDPEDK;KVHVIFSYK;RDELCAVGLDLWQVK;SGTIFDNFLIGDSIEEAAER;VESGDLEADWDFLPPK | 0.3383 | 44.8080 | 15 |
| Dolichyl-diphosphooligosaccharide--protein glycosyltransferase 48 kDa subunit | EGEKESSNTYTITDTVVYR;EYPHAVGR;FIDDGGNLLMAGNAAAGDVYR;IEIEELKNGK;KKPNGVYEAIFK;LADDPNLVLSK;LYHATQVSVRPLQHTQYER;WQPFDANDVQLEFVR | 0.2650 | 34.4895 | 15 |
| Elongation factor 1-alpha | CLIEALDAILPPARPTDKPLR;EHALLAFTLGVK;IGGIGTVPVGR;IGYNPAAVAFVPISGWHGDNMLEASTK;KIGYNPAAVAFVPISGWHGDNMLEASTK;LPLQDVYK;QTVAVGVIK;STEDNPK;VETGILKPGTIVVFAPANITTEVK;YAWVLDK;YYVTIIDAPGHR | 0.3002 | 41.0193 | 14 |
| SFRICE_002792 | AGALLLVLPK;APQVVTTTPPNAGVTGALER;DLQHSFITDDK;EPEFTLYGPTR;HWYTYLSSQPR;INLADELLAWQHER;KINLADELLAWQHER;MTAFTLSSLQSHKDPAR;VQDISIEQFMEIR;WSPDFEDILR;YMDVTVSTHTVDKR | 0.2400 | 52.2483 | 14 |
| SFRICE_008643 (Fragment) | AGYKNDVVLFRPK;DFTDPLVVAYYDVDYVK;ENFHGLVGIR;EQTEVTFAVSDKDDFTHELNEYGIDYAK;ETDLKGDFVK;FEDSSVVYKGEPETYSLK;GEPETYSLK;GEVSQEYNGPR;KDEVLVIGFFEK;KGEVSQEYNGPR;LKPEYAVAAGVLK | 0.4006 | 46.4040 | 14 |
| SFRICE_000208 | DKVQAIIDAVSEIPQR;DLNEENLLNAFKK;FLLLYHNFVLTGSR;ILEDVFPIYR;ILGIFPSLDR;IVLNPEFR;LGIPYNPAYVPFHFLEGGTKPTLYQR;LYTNNWLPQVDILAHPK;SHFFVFAPYLK;VQAIIDAVSEIPQR | 0.0788 | 42.3886 | 14 |
| H(+)-transporting two-sector ATPase (Fragment) | AIAELGIYPAVDPLDSTSR;FLSQPFQVAEVFTGHAGK;ILQGEYDHLPEVAFYMVGPIEEVVAK;IMDPNIIGAEHYNVAR;SLQDIIAILGMDELSEEDKLTVAR | 0.5954 | 23.7492 | 14 |
| SFRICE_008973 (Fragment) | ALIELFESFK;AQLQQGLVK;AVNEALNSLLIDEEDYQGLR;AVTFFTK;EFQGAVDSAR;ESWEDLVR;HELTEFR;IAAYLYK;IVLYAK;LAITLVHLK;NLQNLLILTAIK;NNLAGAEELFVR;NNRPEEGPLQTR;VGYTPDYIYLLR | 0.1062 | 49.4681 | 13 |
| Catalase | DPASDQLVNYKK;FSTVGGESGSADTVR;FYTDDGNWDLVGNNTPIFFIR;GAGAFGYFEVTHDITK;IAAGLAPFKK;IFTQVHPDLGSK;LGANFLQIPVNCPFR;MVNAQGVAHWVK;NGPTLLQDVNFLDEISAFDRER;NLKDSPGYITTK;QVFDDAQK;VSVSNYQR | 0.3215 | 48.0216 | 13 |
| SFRICE_010943 | DLGLFGLYK;DVPFSAIYFPAYAHVK;FGISLPR;FTLGSVAGAVGASAVYPIDLVK;GLVPQLIGVAPEK;GVLIQILESTYR;KPHHIGGYQVALPVLTGLETK;LPFNMESTFVR;SGQTTYNGVIDAAR;SSPQFGVTLVTYEILQR;VINASGFPHGER | 0.2242 | 46.4783 | 13 |
| SFRICE_032354 | DYGVSGFPTIK;KTYDSAEDYNGGR;NDYLAILAR;SADAFVDAALSAVK;SSDSDVITLTDDNFK;SSDSDVITLTDDNFKK;TSSDIVSFALEK;TYDSAEDYNGGR;VGALDATVHQEMAGR | 0.2795 | 37.9901 | 13 |
| Glyceraldehyde-3-phosphate dehydrogenase | AGAEYIVESTGVFTTTEK;GAQQNIIPAATGAAK;IGINGFGR;LISWYDNEYGYSNR;VIHDNFEIVEGLMTTVHATTATQK;VIPALNGK;VKEAAQGPLK;VPVANVSVVDLTVR | 0.3343 | 32.7388 | 13 |
| SFRICE_007086 | AEEEFNIEK;AEEIDAKAEEEFNIEK;HMMAFIEQEANEK;IMEYYEK;IQSSNMLNQAR;KIQSSNMLNQAR;LELIAQQLLPEIR;SLVESVLSK | 0.3097 | 31.6180 | 13 |
| SFRICE_027297 | ALVVYFITSMFR;EFYSNLDDSDLIWK;MESFFAITK;QTGIIYGDWLGGPNNDGTFTHSTTIKPSDSLK;SAEEIIK;SSEDAVDVNAQIDEQR;SVAHLPWR;THNLLTGQTEK;VNEFGFSGEMEQQQK;WQLYTAQAMR | 0.1879 | 40.0965 | 12 |
| SFRICE_003397 | FDYIFFTGGTSIGK;FVELTVLTNVVGSDK;IAIGGSYDANDKFVELTVLTNVVGSDK;IVYEAAVK;KVLQEWYGPEPQK;MLNLLLR;NLTPVTLELGGK;QKFDYIFFTGGTSIGK;QQNVAEAVER;VLQEWYGPEPQK | 0.1646 | 44.2674 | 12 |
| V-type proton ATPase subunit a | DLNPDVNAFQR;EIIDLEAHLEK;ELASWTIMVR;GFQNLIDAYGVASYR;IIFLNSYK;QNYLELTELR;RAELDKPLEDPATGNEIYK;SLISDETGQQAATR;TENEILELSHNAVNLK;TVFVAFFQGEQLK;VPAFER | 0.2177 | 39.3148 | 12 |
| SFRICE_030091 | AADAALAGPQLER;AAPPDVLLQHAER;ADAAAPAALAALR;AELQALQVAAAGLLR;GWDAAAPAGSSSVLAR;LLHPELR;LPDVQLFR;LQALHAEELLAVGHAQLGLK;VLWGLVAELPALR | 0.1486 | 37.4644 | 12 |
| V-type proton ATPase subunit | AIIITINSFGTELSK;AVAEYYAEYQALFEGAGTNVGDK;EKLVIEFQHLR;LHLQGTDYGTFLANEPSPLSVSTIDDK;LHLQGTDYGTFLANEPSPLSVSTIDDKLR;LNPDGLAALAR;LVIEFQHLR;NIVWISECVAQK;QADYLNLVQCETLEDLK | 0.3391 | 38.1132 | 12 |
| V-type proton ATPase subunit a | AIYHTLNLFNLDVTQK;DLNPDVNAFQR;EMIDLEATFEK;EMIDLEATFEKLENELR;EVNQNAEALKR;FTSAFQNLIYAYGVATYR;IDSEIWNIFFGGR;LHWVEFQSK;NYLELTELK;SVFIIFFQGDQLK | 0.1395 | 40.9694 | 12 |
| Prohibitin | AADFNIILDDVSLTELSFGK;DLQMVNISLR;FNASQLITQR;GGPPGLNAGIK;IVQAEGEAEAAEMLGK;QLGTDYDEK;QVAQQEAQR;VLSRPDASSLPTMYR;VPWFQYPIIYDIR | 0.3883 | 35.6999 | 12 |
| Ubiquinol-cytochrome C reductase | HRTENEISTEQAVPVDTLR;KGPAPLNLEVPPHSFVDEGLLVVG;QSFTYLIAGAGGVAGAYAAK;SIVTHFVSSMAAAADVLALAK;TENEISTEQAVPVDTLRDPQHDNQR | 0.3395 | 21.2529 | 12 |
| SFRICE_032560 | EVDPYYQK;HNNLIGTVPPQILANYEAK;LRPLLAILEQPSQYK;LTDFDTHYR;NIPYFWNK;SPGYVQLK;TAYEWKDDANYLLR;VDPFNPDNSDIILEAK;VFDTIFQAVHK;VFDTIFQAVHKK | 0.2137 | 37.0958 | 11 |
| SFRICE_001599 | ELINAAHVFTTDLLTK;EQYLYSK;GTVDKLEVINK;IEIMEFVNFLK;ITTGAQDDLKK;LINPTDIGVK;NFEQAIER;NPQQYIDLGAK;TAELIDSEVR;VHLSPLK | 0.1316 | 40.4287 | 11 |
| Mitochondrial heat shock protein 75 kDa | AILYVPEGKPGLFELSR;ELAESSPYFESLK;ELLQNSALIVK;EYSIFLK;GVVDSEDIPLNLSR;LLETDRPLEIR;SIYYLAAPSR;SLYSDKEVFIR;TDVPLSIR;YISQSYDKPR | 0.1580 | 42.8588 | 11 |
| SFRICE_014898 (Fragment) | AYEGENMLYEK;CDILVPAAIEQVIHK;DIVHSGLDYTMER;ESNYHLLESVQESLER;FNLGLDLR;IETGTIVGFPGAK;IIAEAANGPTTPAADK;ISGASEKDIVHSGLDYTMER;NLNHVSYGR | 0.5094 | 38.5229 | 11 |
| SFRICE_000170 | EEFPTATIFR;ESTTDLVDASIPTLEDLGVTLTK;LVNLISPSYPLGGLHWEAIEK;MEDQVPWELKPFR;SDFYDAQR;VAGDLGQVLFTPYDLRDEESIAK;VGEFPNPPNPK;YKDVHVDGPR;YSNVVINLVGR | 0.3250 | 41.4433 | 11 |
| SFRICE_013982 (Fragment) | ALLELQLEAEELYQTFQR;FKIDLVK;FSVSPVVR;FYAFGR;GSVGFGSGLHGWAFTLK;IKPILFMNK;LWGENFFNAK;VFDAIMNFK;VNFTVDEIR | 0.1782 | 36.8133 | 11 |
| SFRICE_014874 | AKQDLLAALK;FDPENYSIWYAEYK;FDPENYSIWYAEYKYPEELAK;GTFNMDDFKR;KLVQDYFSWTGVDK;LVQDYFSWTGVDK;VLDAHLLTR;VLLSESNAIAYYVSNAALR;VPAYESADGK | 0.2109 | 37.9020 | 11 |
| Prohibitin | AAQFGLILDDISITHLTFGK;AVVAQFDAGELITQR;EFTQAVELK;IEAAEDIAYQLSK;IGQMGLGVALVGGVVNSALYNVDGGHR;ILFRPIPDQLPK;NVTYLPQGQNVLLNLPTQN;RIEAAEDIAYQLSK;SFGQAGEGLVELR | 0.4708 | 36.8831 | 11 |
| ER membrane protein complex subunit 1 | EGSIWAAFSR;HEETGNGLIISLNPITGSLIGER;HVFESASTGNIQLLQITEK;LAYILPAAPAAAAVTHTER;RTEIATVELYEGK;TEIATVELYEGK;VQGYAIIYNGQTVEALR;VVTVSGSNPYLVR | 0.1247 | 35.3457 | 11 |
| SFRICE_002106 | EDTGALPDETSFNLVNEASVDDLNTR;GAKPYDEDNWK;LLQEKPVK;LNVNLAEIVALQKPK;LPEKWEQVGVLK;NANTEPLQELKK;VLMVVDDKDNFITAR;WEQVGVLK | 0.2964 | 30.5226 | 11 |
| SFRICE_023417 (Fragment) | DELDYSKPLDGQTK;HTLAETNIETGETK;KHTLAETNIETGETK;LPFEQHWR;RDELDYSKPLDGQTK;YANGTISTADLR | 0.2762 | 23.7065 | 11 |
| SFRICE_003104 | AWQELFLPIIELK;DYVADKTPVVVATIDQYAPGLVDNVQSYASTGFQTIK;FSNAPLVWLK;KVGVLYSNTK;MTVLQYK;SHVSSYFHWFR;SLSDVLHDAGK;TPVVVATIDQYAPGLVDNVQSYASTGFQTIK;YKDIYFNK;YSNDYYQLTAEYLK | 0.1825 | 39.7046 | 10 |
| SFRICE_002276 | DHDFIVNLDQR;ELPDRNPETCAEVFLNLLTHISK;GKHIIEQLGGK;IVLAVFR;LMVHNWEYLGK;MGNPWQPFLNLLNR;NLIEKPEDHQVAK;SDLHFYLSWLK;SDLHFYLSWLKDQLK;VMYLLSHEDPNVR | 0.2165 | 45.4781 | 10 |
| 40S ribosomal protein S12 | GINQVSPK;KVLQLFR;LNNPTGGWR;QINNGVFVR;QNIICVEDLIHEIFSVGEK;TALIHGGLVHGLHEAAK;TIHYVDGGDFGNR;TIHYVDGGDFGNREDK;VPITSNTIVEQK;YASNFLWPFK | 0.256 | 40.9398 | 10 |
| C-1-tetrahydrofolate synthase, cytoplasmic | DGPLFDR;HGNDTPAELNLVK;ITPLSPPPSDIVIAR;IVGTPVSELLK;KITVGQSPTEK;LLAPTWPLRPLR;SKFDFLYPLQLSIQEK;TADILVVAIGR;TGVTIAGK | 0.1115 | 37.5464 | 10 |
| Formylglycinamide ribonucleotide amidotransferase | DPTSVELFDLAQSNSEHSR;ELVEPEGAVVYTK;FFSSEAFSAHK;FGEPLISGFVQSYGLK;GLLDPAAGAR;LHEALAAPLHDR;NLQGNQIVIEIGPR;TQVFLDHNTSER;YLISFGK | 0.0852 | 36.2598 | 10 |
| SFRICE_018970 (Fragment) | FGLYEVFK;GLAPLWGR;IQTMPGFASTLR;MVQNEGYGTFYK;NVVNGFK;TATVGGIMGK;VSVAEEGMR;VTYSGLLDEETAYTYR;YVVPKPR | 0.3038 | 35.8495 | 10 |
| SFRICE_019735 (Fragment) | GVADFAIIHYAGK;IEAPLVLDQLR;IPFQEFR;KAQLEELQIVLTK;LQSEAETAAAQLEAAELNASAAHK;LTQKEDELR;NWQWWR;QAEAQLQEIAAK;VKPLLEVTK | 0.0824 | 33.6925 | 10 |
| SFRICE_021217 (Fragment) | DLALYALGIGASVLNEGDLK;FVKPVQPGQTLVTEMWLEGK;GSSAISITNSEIYQNK;GSSAISITNSEIYQNKK;IQDFEER;KKPSDPVTIESVK;SYWSSYTYNSK;TAEDQAALYR;TQQHIFVLGQGGFNGPR | 0.2050 | 33.2728 | 10 |
| Elongation factor Tu | AHDNVEAAVYILSK;EHLLLAK;ELDKPFLLPVESVHSIPGR;GITINVAHVEYQTEAR;INNNLSEEDR;INNNLSEEDRLK;SPEIGSEAISQLLK;TLDEAQAGDQLGALVR;VLADVNLAQK | 0.2323 | 38.8332 | 10 |
| Cytochrome P450 CYP4C1-like protein transcript variant 1 | AFADNVIMDR;EITEDFK;GTTVDVHIYELHR;ILTPTFHFNILK;LAMLDLLLEAEEK;LDNDQTSATVDYK;LYPSVPFIGR;TTLSELVR;YLEAVIK | 0.1879 | 36.3821 | 10 |
| Adenosylhomocysteinase | FPQYLEGIK;GISEETTTGVHNLYK;HNVYPVGVHTLPK;KLDEEVAALHLDHLGVK;KVNIKPQVDR;VNIKPQVDR;YELDNGNHIIVLAAGR;YIGVPVEGPYKPDHYR | 0.2233 | 31.5312 | 10 |
| SFRICE_001276 | ADLIFR;ENPGTLDEIHKK;KLQPLPFTFALSGMANQAK;LALGTELVYQSGAR;LGATYIGTK;QISPSEAFPVILGDVDPSGNVNFSLIHQLTPEIR;SAVFVGHYLQSVTK;VMGGEIAITSAAAR | 0.3873 | 25.8116 | 10 |
| NADH dehydrogenase [ubiquinone] iron-sulfur protein 3, mitochondrial | ADQAGPQAETRPTVAK;EIWDMYGVFFANHPDLR;FEIIYNLLSLR;ILTDYGFEGHPFR;LHLIDFGK;RFELSAPWEQFPNFR;RVVVEPLELAQEFR;SNPASEEVVNPADEK | 0.4113 | 33.8767 | 10 |
| Electron transfer flavoprotein subunit alpha | DPEAPIFQVSDHGLVADLFK;GLTPETLTPLILATQK;IGGDVAVLVAGTK;KIGGDVAVLVAGTK;LDVSPITDIIGVK;LLYDLADKLNAAVGASR;TDLVQFVSQELAK;TIYAGNAILTLEAK | 0.3242 | 32.9279 | 10 |
| Triosephosphate isomerase | ALLPAIGSNWDK;DIGANWVILGHSER;QVTEIVETLK;QVTEIVETLKK;TATPQQAQDVHASLR;TEEVVFR;VVLAYEPVWAIGTGK | 0.2984 | 33.2489 | 10 |
| SFRICE_020830 (Fragment) | AMTTEFLTEIETQYK;FFLMNLFGIPK;LNFDQYTER;LNFDQYTEREEPK;LNVPELITSADDAYER;SPYSDFVFLEAMYR;YSQYIDALTHLLNTGQGVVLER | 0.1402 | 33.2489 | 10 |
| Serine hydroxymethyltransferase | AGVIFYR;LAQEITK;QATTPEFVEYQK;SALHPSGIR;SLKEEIENYSR;TGFIDYDKLAETAR;YYGGNEYIDEIELLAQKR | 0.1677 | 26.2010 | 10 |
| SFRICE_038524 (Fragment) | DVITQHVCVR;FGISAPHIR;NAAHLVLK;TSRPNTAILYPNDEVFK;VLDPFTIKPLDEAAIQQHAK;VVVVEDHYQAGGLGEAVLSALALQR | 0.5779 | 24.9614 | 10 |
| Dihydrolipoyllysine-residue acetyltransferase | FVTFTASADHR;ILIPAGTK;LGGQGSGLYGSLK;QTIPHYQLTATVNVEK;RVPTVNSHWMDSFIR;VPTVNSHWMDSFIR | 0.1263 | 25.9608 | 10 |
| ATP synthase subunit beta | IGLFGGAGVGK;IINVIGEPIDER;IPSAVGYQPTLATDMGTMQER;TVLIMELINNVAK;VALVYGQMNEPPGAR | 0.0965 | 21.97004 | 10 |
| 40S ribosomal protein S4 | AYISLPR;DSTGHTFATR;ERHPGSFDIVHIK;LNNVFIIGK;NVPFLVTHDGR;VGTIVSR;VNDSVQLDIATSK;YALTGNEVLK;YPDPLIK | 0.3308 | 33.1909 | 9 |
| Glycerol-3-phosphate dehydrogenase | AIMQLDYDQYK;DKIPINLSK;LVQDFGLEMEVAQHLAK;MNLAIALTAAR;MYDVVAGDR;RGDVLSAWSGIRPLVSDPNKPDTQSLAR;TALVEADDFASGTSSR;TAVDMIAR;VIFFLPWLK | 0.1565 | 34.1870 | 9 |
| SFRICE_025754 (Fragment) | AQQLIDHVAR;EPWTYANK;ETANFPK;GADTIVQLIK;KAEFLTPPEK;SLGYESVYHHAVIIDAGSSGSR;SVLPAVDWEPR;VPLTLYGPSNLDLVEEFFEQSKPGLSSYADDPAK;YGVFQTEASQADTQEFR | 0.3177 | 35.4467 | 9 |
| SFRICE_003033 | ANRDEIEALYENK;DALQNLNDR;DQVFQQELQETR;ELSLGGYQILR;HAEDLASLEQELAR;IDSLNATLNDLENK;LYEENDDLKK;NSTAATVAVEELR | 0.1558 | 34.3891 | 9 |
| SFRICE_004313 | EAGFPPGVLNVIPGYGPTAGAAISHHPGIDK;EEIFGPVQSILK;FETFEEVVDR;LFINNEWVDAVSK;LIMGAASAVNLKR;QAEGEVLWSAGIIR;SALVVFNDADVEK;TVGNPYTDVQQGPQIDNDMFTK;VMGYIDAGK | 0.2551 | 33.9922 | 9 |
| SFRICE_000126 | AFAGVVSR;EGYFVEPTIVTGLPHDSPLVK;IGDPLLSETLIGPLHTPAAVEAYK;IGDPLLSETLIGPLHTPAAVEAYKK;ILPEAIGEVVEYIHVCDLALGLSR;LQPLGQLVSLEMGK;QIGDALR;YNNEVEQGLSSSLFTENLGDVFK | 0.1730 | 28.3303 | 9 |
| Carnitine O-palmitoyltransferase | EFEETIAVK;HQLGYQDAMAGR;LLKPAEIQIQIQQILDDKSEPVVGEER;LPLPAVK;LYSFQGSLPR;VLSGWNKPK;VLVYSR;YLELESPFLQEVFNEPWR | 0.1261 | 34.6580 | 9 |
| SFRICE_006893 (Fragment) | ALGNGPVTK;ANMVAQHLNLSSSEVSAEASK;CAVAVVGGSQER;EMGGDLAHVAIAVQGAKAGSPQALALAVAAK;GLGNSLFISPK;KPSLNAAAIQAGK;QIDALSPNDVSQALSSAAANK;WGSDNSPLAK | 0.4655 | 37.9987 | 9 |
| SFRICE_001242 | AVQSAAITDGSEEK;AVQSAAITDGSEEKKPEEKPAEPPMDPNVK;FYSEVHALECK;GIPDFWYTIFK;KPEEKPAEPPMDPNVK;LYKPLFEK;NEMLAAITNR | 0.1763 | 25.8053 | 9 |
| Dolichyl-diphosphooligosaccharide--protein glycotransferase | EADYYTPSGEFR;FESVIHEFDPYFNYR;FYSLLDPSYAK;GAEIGNKDFNLDVLEEAYTTEHWLVR;IIFDDFR;VGQAMASTEDK;YLTEEGFYNFHNWFDDR | 0.1400 | 27.3692 | 9 |
| CAAX prenyl protease | FVPLPDGSLR;IVLFDTLLEK;IVLFDTLLEKFDEEK;LHFPLSQIYIVEGSKR;LYSAWYHSHPTLLHR;QTAGFFIK;SAHSNAYFSGLFGNK | 0.1642 | 26.6748 | 9 |
| Fructose-bisphosphate aldolase (Fragment) | AWAGKPENVLAGQQELIK;AWAGKPENVLAGQQELIKR;FTPSYQAIMENANVLAR;IVPIVEPEVLPDGEHDLDR;RPWALTFSYGR;VTEVVLAAVYK;YASICQSQR | 0.3629 | 29.5063 | 9 |
| Probable methylmalonate-semialdehyde dehydrogenase [acylating], mitochondrial (Fragment) | AVSFVGGDAAGK;EHTLNQLAGAAFGAAGQR;NHGVIMPDANKEHTLNQLAGAAFGAAGQR;TLADAEGDVLR;TNNWIELTNPATNEVIGR;VNAGHVPATDVGPVISPK;VPEATKDELNAALESAK | 0.2909 | 33.2489 | 9 |
| Glycine cleavage system P protein | GLVAHEFIIDVR;LDALGLEVNVVPDVR;LGVPLGYGGPHAGFFAAEHQLVR;LHPQTLAVVLTR;QSNDVYFDTLHVVPLSDHDAAAIK;VHNATLVLDHGIR;VSFQPNSGAQGEYAGLR | 0.1394 | 29.3335 | 9 |
| Retinol dehydrogenase 14-like protein | EAIALIR;GNINFDDLNIR;GNINFDDLNIRK;IDVLINNAGVVVPLNQDLK;IVVVSSTLHER;TFLITGANSGIGYETAK;YNFDKDVETK | 0.2269 | 28.0937 | 9 |
| SFRICE_014294 (Fragment) | ELSALYHDR;LTKPGAALVYWVEHVVR;NHPYQIQEFAQEAVR;QTVIWK;SPALHVPLYQR;VEELWSR;VLPELPK | 0.0504 | 23.5097 | 9 |
| Heat shock protein 83 | ALLFVPR;AQNYTFQTEVNR;DVLDANSDLSIR;EEAADYLQPDTIR;EIFDLEENEQQKER;ELISNGSDALDK;ELISNGSDALDKIR;GEEMTFLADYVAR;HPLIAELLR;IPDSEYESFWK;KIPDSEYESFWK;KPNEVNDEEYTQFYK;KVLDMLK;LGVIEDPSNR;LIINSLYR;LLHIIDSGIGMTR;LLSLTERDVLDANSDLSIR;MKPNQNHIYYIAGSSR;QFEPLTTWLGNK;QHVWESDASAFSVAEDPR;QHVWESDASAFSVAEDPRGDTLR;SGTAEFLSK;SPAALAATAFGWTGNMER;TVWDWELMNDNKPIWTR;VQEAPDSPDTAR | 0.3473 | 23.7492 | 9 |
| Sodium/potassium-transporting ATPase subunit alpha | AAVPDAVAK;AEDLVLGDIVEVK;AINDLTDSYGQEWTYR;EAFNNAYLELGGLGER;ELNSDQLDEILK;EVAGDASEAALLK;LNIPVSEVNPR;NLAFFSTNAVEGTAK | 0.1014 | 32.8994 | 8 |
| SFRICE_000701 | AAADASNAINSTVDNTLK;DTLANTVHSTVDTTK;DTVAHTFDSTK;DTVAHTLDTTK;DTVANTVHATVDTTK;ETVASTISTTVDTTK;NVAAAVVEK;NVAASAFEK | 0.1004 | 32.5775 | 8 |
| Cation-transporting ATPase | ALEEAPR;DPIQAEIVK;GSIHPDVWFIFQK;GYLDDEAIDIAEK;HADVGVAILANAPERPR;QIMSDQLLPGDVVSLIR;QKEFVVVTLK;VLALGYR | 0.0802 | 24.9943 | 8 |
| SFRICE_020749 (Fragment) | ASVVHLTEEVVGR;FEQTSQQGGTCTTADR;IIIAVGHSGYLR;KIPVVQAYAYTK;TTLGYNELACTK;VIMPSFLSTGGDGFSMFLGLPK;VLAVPNDVEYIDEIEALNK;YSPIHPQIEGR | 0.0978 | 30.5342 | 8 |
| 60S acidic ribosomal protein P0 | APARPGAIAPLSVVIPAHNTGLGPEK;DHLETNPALEK;GHSIVLMGK;GTIEIINDVHILKPGDK;IIQLLDEYPK;QVYDSGTIFAPAILDIKPEDLR;TNYFTK;TSFFQALSIPTK | 0.3587 | 35.9207 | 8 |
| Alpha-1,4 glucan phosphorylase | DYYFALAHTVR;FLHLADYDAYIEAQEK;IEHGEQQEEPDDWLR;QLLNILHVITLYNR;VAIQLNDTHPALAIPELLR;VDDVEALQK;VLYPNDNFFEGK | 0.1141 | 30.7176 | 8 |
| SFRICE_035016 | DGVTSMESMLR;FNMGEYVWR;HFGAGLR;IIQTTPNSITVR;QNIVMFAETR;TVLSEEDEPQPNGR;VDTIIYMEDQLK | 0.2107 | 22.3203 | 8 |
| UDP-glucuronosyltransferase | AFNELIHPIILK;ANELWNQVK;EAIIEVTSNK;EAIIEVTSNKR;HLLNAGHEVTYISPFPYK;ILVVFPLPSR;SLLQMFGTLK | 0.1341 | 26.2106 | 8 |
| SFRICE_008303 | EAAFSLAEAK;FTTGDFNQVVLQNVTK;KQAALLAGQDLADAANLLDEGDEDLLF;RVNAIEHVIIPR;TLAYIISELDELER;TLAYIISELDELEREEFYR;VNAIEHVIIPR | 0.3415 | 33.2489 | 8 |
| Annexin | ADAEALASAGEGQWGTDESVFNSILITR;LEIAETFK;QIFAEYEALTGK;QIFAEYEALTGKDIEETIK;SLESDLKGDTSGHFK;TISAFYEQLYNK;VGFFAER | 0.2755 | 28.4090 | 8 |
| Aminomethyltransferase | AGTELLVNIR;ALIALQGPK;DGSSSLTVFLNDK;SASIFDVSHMLQTNVR;TPLFNLHTK;VNAETLYVVSNAGR;VVDFAGFLLPVQYSDLSLSESHLFTR | 0.2354 | 33.2489 | 8 |
| SFRICE_003274 | ELLDENGPK;IWLRPGQELSVK;LSVEGEANVDLLR;VTLLDAIGR;VVPLKPGVITIDVR;YAVEAPK;YLTVGKPPEWR | 0.0437 | 27.7298 | 8 |
| Proton-translocating NAD(P)(+) transhydrogenase | AVIEAAAHFPR;FAIHPVAGR;FFSGQMTAAGR;IHGVTHIGLTDMPSR;VAVVPAVVSK;VLVVGGGVAGLAAAAQAR;VRPLIDHEVQSVRPEGTLISFLYPAQNQELIK | 0.1011 | 25.3984 | 8 |
| Complex I-49kD (Fragment) | FAERLDEVEDVLTTNR;HPPYHGVVAPGEK;LLNIDIPLR;LVLELDGETVR;NMVLNFGPQHPAAHGVLR;TLFAEITR;TYTQALPYFDR | 0.2857 | 28.1077 | 8 |
| Peptidyl-prolyl cis-trans isomerase | HTGPGVLSMANAGPNTNGSQFFITTVK;HVVFGTVVEGMDVVK;QVESYGSQSGK;TSWLDGR;VIPNFMLQGGDFTNHNGTGGK;VYFDVSADGSALGR | 0.5758 | 27.4577 | 8 |
| SFRICE_011143 | DYGVLNEETGIPFR;GLFVIDDKQNLR;IGCEVIGASTDSHFTHLAWINTPR;LVQAFQYTDK;QGGLGPMNIPLISDK;QITVNDLPVGR;TTAVVNGEFKDIALSDYK | 0.2946 | 26.7199 | 8 |
| SFRICE_035634 (Fragment) | DPALVFFR;HGVPLLYSGESDENEIYGFFEK;LHAVWESVGATLK;LYNPAKDPALVFFR;MDANLAGVNTAK;SFVIFAQDWYK | 0.2802 | 25.8086 | 8 |
| SFRICE005413.2 (Fragment) | AAQLDPNNSDIYHHR;ASLFTQLENTER;MDEATAEFAK;NEALLLR;STFYLLLGR;TYFSAFSEDPITK | 0.1204 | 24.4446 | 8 |
| SFRICE_026740 (Fragment) | ALHAALLAGVLR;APSIGFFDCTPVGR;DGEVSEAGSYQQLLEK;GAFAEFLLHHLSDAER;LGTEFQNK;SPIYSHFGESITGASTIR;TPEATPNELKEK;TSPEELDEIKQDLESK;VLGPNGLLK | 0.2068 | 24.2029 | 8 |
| ATP synthase subunit gamma | AARPYGEGAVQFYER;AEVAPPEDDPK;GLCGAVHTGVSK;HIISVCNEIGR;IASAILESGYEFASGK;SVVSYQQSDLPLFSQK | 0.2746 | 26.7199 | 8 |
| SFRICE_000822 | DGPDEEGNYFERPGK;KGGEDYIFALLTGYMDAPAGVVLR;LAYKPVSK;LSDYLPSPYPNENAAR;NLVNVTHTEEEAK;NLVNVTHTEEEAKAEAAEAMIK | 0.2615 | 26.5817 | 8 |
| SFRICE_033731 | DGDYVLVLK;GVAHDEYIPYSIK;INALYVMR;LYYNEEESDIR;LYYNEEESDIRGGK;VNTKDEILYQTER | 0.2151 | 28.4991 | 8 |
| UDP-glucuronosyltransferase | AGVPLLAVPVFGDQPANAER;GIVDALLQGGHTVTWVTPFPEK;KLINHYVELAIETK;LFASLPYTVLWK;LIDISPVRR;LINHYVELAIETK | 0.1481 | 23.9114 | 8 |
| SFRICE_003046 | AFSPATVNTGR;FLEVIKPFCSILPEIAKPER;GTEFEGAVIALFHLLATRPDK;IIEVGDTPK;IIEVGDTPKDR | 0.1324 | 23.7492 | 8 |
| SFRICE_008127 | ETHFFLR;GNIITVLFIK;IVDELIPSVLR;LLDYFLGEEIGTHYNR;VTDHVNDLDKEEVNIISGALDLR | 0.0931 | 21.2568 | 8 |
| SFRICE027725.2 (Fragment) | AAIASYEKDEAVK;HLLVVPDLEK;LLLAFGDEGFEDHR;LNEIITK;VAFNDWPEFKPK | 0.2745 | 18.4931 | 8 |
| SFRICE_009042 | APHSVQMTLVAEFFK;DVESMLGYVEDQR;ELNEDNWR;FVVTALPTIFHVIDGEFR;QTDPVPAWK | 0.1785 | 19.1370 | 8 |
| Complex I-B22 | AVELLKEGEDELFK;EGEDELFK;LFPTSQGGVAHQR;NLEAYYDRR;VVTPPDWVIDYWHPLEK | 0.3655 | 18.1563 | 8 |
| Aspartate aminotransferase | EFHIYLTK;HGHQIILAQSFAK;ILPFFDMAYQGFATGSVDNDAFAVR;IVTEILSNPDLK | 0.2302 | 17.6941 | 8 |
| Lon protease homolog, mitochondrial | DYDDLPGFIR;FQQTEEVK;ILEFIAVSQLK;IVKEETQSLK;LIEISNPALIDLIR;LNQPYVGIFLR;VTEANLTELVGKPTFK | 0.0830 | 30.0179 | 7 |
| SFRICE_006078 | DIEVIEDALEK;LFPNALPSTFESTDQK;LIQDVLGR;LLFIEVR;LLVTTIGDLFR;VLNASTIGTSAMLR;VPPSLLLLSR | 0.1016 | 26.2792 | 7 |
| SFRICE_010556 | FEHPLHHLGK;QFLLDLYSGK;QLTDPVVTFGALK;QNVNFLTADGVR;SISDLPLIAIDSFR;SVDAFAEFIK;SVDAFAEFIKK | 0.1720 | 26.0784 | 7 |
| Aspartate aminotransferase (Fragment) | AFGVQTLSGTGSLR;AIDFDGLIEDLR;GQPWVLPVVR;INLSIGAYRDEK;NFGLYNER;VGAELLNK;VGNLAIVVSDASVVQALK | 0.2628 | 28.0545 | 7 |
| SFRICE_015281 | EEIFGPVQSILK;FDTLDEAIDR;FDTLEEAIDR;LFIDNEWVDAK;LIQQASGASNLKR;VAEGDKADIDLAVAAAK;VSFTGSVEVGK;VTGETIPADGEVFSYTLR | 0.1060 | 29.1505 | 7 |
| SFRICE_036980 | AMVVNAAQLSTYSQAR;EGFVALYTGLSAGLLR;LGVYNYLFDSYK;LGVYNYLFDSYKEK;LPVEQR;NVADALIR;TLLGVAAGSVGAFVGTPAEVALIR | 0.4058 | 26.2432 | 7 |
| SFRICE_006684 | GITCFIVER;IGTIYEGTSNMQLQTIAK;KDGDHYVISGSK;KLATEQIAPLVK;LATEQIAPLVK;SIYSFQGISYQIAHLQTQLEAAR;YAAGFLNEGR | 0.2010 | 31.2285 | 7 |
| SFRICE_013269 (Fragment) | AVHLVYEK;ENRFDAWTILIR;FDAWTILIR;LATSIDEFGR;LEWEAASK;LLTTIGDEAIFK;QFVLSNEK | 0.0394 | 30.4918 | 7 |
| 97 kDa heat shock protein | DVQPTAEGESQK;EFSVTDVQPYAVR;HASTTLNQDEAVSR;LAWDATK;LRPEDIHSVEIVGGSTR;NTVFGFK;YAHLADVDVQK | 0.0974 | 28.2827 | 7 |
| Pyruvate dehydrogenase E1 component subunit beta | ELAGSAGIECEVINLR;GTDTALNAAK;HTDVIAAVNAVLGNK;SIRPLDFDTIAR;TFYMSAGEVPVPIVFR;VLMPYTSEDAK | 0.2133 | 26.9374 | 7 |
| Vesicle-fusing ATPase | AFEEADKNSPAIIFIDELDAIAPK;EIDIGIPDATGR;ETVVEVPNVTWGDIGGLQNVK;LAGESESNLRK;LDQLIYIPLPDEK;LIVEEAVSDDNSVVALSQAK | 0.1253 | 27.4577 | 7 |
| SFRICE_001400 (Fragment) | AYQALTDDEAR;ITDNEEVPMLLR;LSPMIVQGLWEYK;QIIIEQSQPYK;QSLILHPDKETGDEK;SPLLQLPYITEDHLK | 0.1174 | 23.0429 | 7 |
| SFRICE_023375 | AVWWTEHVLR;IFLASTK;ILAVFPNPSISHQVVFRPLTQELAR;ITEFYNDFMLNR;KIFLASTK;LTVEDFTNAINTVINDK;TLSQLPYDILWK;WNGDVLPGQTPNIK | 0.1245 | 27.1937 | 7 |
| SFRICE_018917 | DKGGNGGTILNLASIYGYR;FAVMGFTK;GGNGGTILNLASIYGYR;KIFENYK;VDQYLPVYQASK;VVVITGGAEGIGYEVADR | 0.1248 | 26.0058 | 7 |
| Gamma-glutamyl kinase (Fragment) | ASAIHTLADLLVNKQEEILEANTK;GPVGVEGLLTTK;GVGADDAISLVSTR;HIDLIIPR;LASQLTFGPPPAR;TLSVGLK | 0.1285 | 20.5970 | 7 |
| SFRICE_015719 | AINTVLSNK;AVWWTEHVLR;ILAVYPVPSISHQITFRPITLELIK;LEELWNFYR;LLELYNIYR;NAINTIINDESYR;RGHQVTVITPDPAFPK;WEKLEELWNFYR | 0.0624 | 25.7419 | 7 |
| NADH-cytochrome b5 reductase | AYTPVSSDDEKGYVDLVIK;IDDDLVIR;LLFANQTEEDILLRNELEK;VWYTLDRPSEGWK;YALPLIER;YQSEHPEQFK | 0.2355 | 24.8987 | 7 |
| Annexin | AEIDLGSIK;DPTVVGVPNFNATEDAAALR;EDAQALYDAGEAK;ILAHESFAQLSQIFEEYK;LLTLIVTGAR;WGTDEEVFNR | 0.2116 | 24.6122 | 7 |
| SFRICE_001003 | AGIPQEKPALGISR;FGVPLGVK;GIFIVAAK;ITAHLAHELR;ITAHLAHELRR;QNGFTPLVR | 0.0627 | 22.2247 | 7 |
| 5-aminoimidazole-4-carboxamide ribonucleotide formyltransferase | AALWWMR;DGQVVGIGAGQQSR;ITADLFK;LALLSVSDK;TIFGLTLEQR;TLHPAVHAGILSR | 0.1010 | 25.6785 | 7 |
| SFRICE_008279 | AYDLLAK;DKTNSESLADIEK;LYSDPGFTR;VIELSSRPNNQGIGILTSENR;YIEENKAPEGSSK;YLLTVRPFLSDDEYAR | 0.1219 | 22.7030 | 7 |
| SFRICE_036126 | DNYVFEFIPENR;DTLKDVVAPEQALVAWGGK;DVDGSLLFIFK;DVVAPEQALVAWGGK;EGLFFTHGR;ILIYWFDR | 0.1294 | 25.8350 | 7 |
| SFRICE_009400 | FSEFVFLER;GFTTSLQFFR;IPVIYNIQLAFEK;ISQGSSYGTEK;TPPTLTSLLYGKPVNAHLYIER;WLHELFVVK | 0.1932 | 28.4991 | 7 |
| Casein kinase II subunit alpha | FNDILGR;LIDWGLAEFYHPGQDYNVR;TPALIFEHVNNTDFK;VLGTEELFEYLDKYHIELDPR;YYLYELLK | 0.1983 | 19.9797 | 7 |
| Microsomal signal peptidase 25 kDa subunit | ESFALIDGR;GVFVVAK;HDDKYNLVLVMR;SFANFIDTNGTIVQNLVVNEITK;VWEASSYVK | 0.3261 | 21.0480 | 7 |
| 60S ribosomal protein L5 | IEGDHIVCAAYSHELPR;LGVNADAIEATYKK;LSNKDVTCQVAYSR;SLEADDEDSFKR;VGLTNYAAAYCTGLLLAR | 0.2508 | 21.2444 | 7 |
| SFRICE_009010 (Fragment) | KLQQIGASVTASGDR;QLVSGQEFRPWELTDNEPR;TFVAALDNGSPVTR;YANSFILAR;YEPQTELGVAHMLR | 0.4437 | 23.7492 | 7 |
| SFRICE_012732 (Fragment) | DVDTVITAYR;GDYIPGIWVDGMDVLATR;LYEQLAILR;NFYGGNGIVGAQVPLGAGIGFAHK;RIETASGNLYR | 0.1915 | 22.4439 | 7 |
| FK506-binding protein | IIQVPLDLYFK;KQIAGGVSIEDLK;MFWGLIMEPNKR;SGDSVSFLTNGK;VVMVYYEGR | 0.1380 | 23.7492 | 7 |
| SFRICE_019264 | GVSQDVMLDIK;LKPMELELK;NYEGIGEAAK;RLEDLSEAIVQDFTLMR;VDYIIR | 0.2585 | 13.6840 | 7 |
| Moesin/ezrin/radixin homolog 1 | EEWEQSITNWWQEHR;GDLTWIK;GPDDLVDPVADRR;IGFPWSEIR;IQQEVELKDSETR | 0.0991 | 19.6443 | 7 |
| SFRICE_000916 | AAQTLFWTELAR;EPATINYPFEK;GFAVTLAHIFK;LLSNGDKWESEIATNIR | 0.2329 | 18.9994 | 7 |
| Nucleoside diphosphate kinase | EIALWFGDK;NIIHGSDSVESAK;NIIHGSDSVESAKK;TFLMIKPDGVQR | 0.2288 | 16.5062 | 7 |
| ATP synthase subunit d, mitochondrial | ANLAAFK;ISQSAVNWAALAER;NAVPIPGMVDTFQK;SIEAFIQESNANIAK;VPYPADTQTAQVEAQWAEVK;VPYPADTQTAQVEAQWAEVKK | 0.4080 | 28.4991 | 6 |
| Calcium-transporting ATPase | AGQILWIR;EASDIILTDDNFSSIVK;ERHPEESFTR;GASEIVLKK;VDESSLTGESDHVK;YGDLLPADGILLQSNDLK | 0.0644 | 23.5173 | 6 |
| SFRICE_013096 (Fragment) | AEAALAYELQAAK;DVYDDVQYLASLGK;IAAEVSAPLAK;LPAEAEAYR;QIEVEQQEILR;VLGLAEATAIGDVGK | 0.2239 | 27.4577 | 6 |
| SFRICE_000544 | DLQIHGMVR;ESIEPAVAESLANFK;GKSDPYAIITVGAQQWK;ILLQVWPNVNSYAR;LDDLPAWVFFPDVER;SDPYAIITVGAQQWK | 0.1813 | 24.2019 | 6 |
| Malate dehydrogenase | ENFTAMTR;IVQGLTITDFAR;KDLLAANVR;STFVTTVQK;VLVVGNPANTNALICSK;YAPSIPK | 0.1851 | 23.7514 | 6 |
| Actin (Fragment) | AGFAGDDAPR;AVFPSIVGRPR;DSYVGDEAQSKR;IIAPPER;IWHHTFYNELR;SYELPDGQVITIGNER;TTGIVLDSGDGVSHTVPIYEGYALPHAILR;VAPEEHPVLLTEAPLNPK | 0.3059 | 23.7492 | 6 |
| SFRICE_005246 | ESLASTSQNATEALTR;FEVEHQER;QHEIVSQNLAETK;TLLSYNSGDPDLISFR;YVSAESALEEFKK | 0.0508 | 23.7492 | 6 |
| SFRICE_001395 | AEQFYR;IEPPNVGK;IIDDHLAVQAIIR;YADQLITEGVVTAAEVK;YHLGTYIER | 0.0673 | 16.446 | 6 |
| SFRICE_003674 | EGSSEGQIPTLFASISK;GKEEGVWHIDLK;KVDIVSDAAYVILSK;LPGTIYTAAEEIEALGGK;TYTGNFAIDEDIVK | 0.1784 | 22.7078 | 6 |
| Mannosyltransferase | ILQAVISTAADYSFYK;LGHYEQESPK;LIVTPWEFFR;QAPDLLVLYDR;SYLYPSVVAGLYTLLK | 0.1248 | 23.7492 | 6 |
| SFRICE_027087 (Fragment) | AELPQFETLAVSVPK;EIGLVSEVFPDKDTALTK;EVDVGLAPDVGTLQR;INLVYSQSRPNK;VLEIAENIASK | 0.1972 | 23.7492 | 6 |
| SFRICE_002345 | QAELALTTAR;SLEMYIQALK;TQDTQEFLMFVGHPK;VETFWNTLQEVWIK;VGLIDETATDK | 0.0972 | 23.7492 | 6 |
| SFRICE_010318 | EANNPFFTPK;LGESEYFFGNRPSSFDATVFAYLAPLVK;LLTNFEEVVDHLK;QAAEASAFTQYLR;SLHYSTDVHLNTK | 0.2429 | 20.1909 | 6 |
| AIR carboxylase (Fragment) | KATILVFEILEK;LATGSFLK;NLTTVTAADLDTVK;NLTTVTAADLDTVKR;SAGINTAFVK | 0.0549 | 21.2529 | 6 |
| SFRICE_026018 (Fragment) | DDASIQHGHYVVAGVPAR;EFDNVLK;GSDLAADHTYYSYWR;TGHTGYVALFNPTEDDQR;VVDDASEAPGTGSSVFTVNEEGGR | 0.1416 | 17.8204 | 6 |
| Fatty acyl-CoA reductase (Fragment) | LIPIAGDVGEENLGLSPQDR;LLESNSTDIFKK;SHPNTYTFTK;VISLVGTLNDEGLLEVEPK;VYEAPEDPEK | 0.2700 | 22.1876 | 6 |
| SFRICE_014941 (Fragment) | AQAVAELQNEVQK;AQIAENNLELLKEESEK;DAAALAQQHAADLAQQLQDANR;LSESEAALVAQLTALQGELNAHALEAGQR;QQLTTEIASLR | 0.1707 | 21.9188 | 6 |
| SFRICE_036227 (Fragment) | ESLDLFK;LEEYFADFAR;SRLEEYFADFAR;TISVILFLNK;YFIRDEFLR | 0.1784 | 16.9429 | 6 |
| Glucose-6-phosphate 1-dehydrogenase | EPFGTEGR;IKPVPYVYGSR;LASYDFK;LSNHLAGLFK;VIIEKPFGR | 0.1025 | 17.6043 | 6 |
| SFRICE_008092 (Fragment) | DIHEADYFTER;IAEGEHPKDIHEADYFTER;LTYLEEAYTTEHWLVR;TVPTSNTISK;YDSGGSPPGYDR | 0.2088 | 16.6900 | 6 |
| Signal peptidase complex subunit 3 | ERNDLGFLTFDLK;NDLGFLTFDLK;TDLSHLFNWNVK;YYFWDDGNGLK | 0.2011 | 17.2202 | 6 |
| SFRICE_004073 (Fragment) | ALSALMTFK;FFDMVEYFFHR;FSTDVSRDEVK;LKDIPTHANPR | 0.2500 | 17.0090 | 6 |
| SFRICE_038072 | AEYNAIPLK;DLDHTEAIPSVTAGGVGFSYANIR;EVFFSDPGQQIIK;VKEVFFSDPGQQIIK | 0.4364 | 16.4681 | 6 |
| SFRICE_019553 | LTELQLR;QLLDQVHQITK;SFFEAK;VHLDIQVGEHAVDYANVAHK | 0.2028 | 12.4058 | 6 |
| Cytochrome P450 CYP9E2-like protein | FGGIFEGR;KPVIFVGNLGPR;RPLLTILDPDLIK;STLTPVFSSSR | 0.0849 | 16.6527 | 6 |
| SFRICE_000610 | ASVVFATTDK;ENILGQPGLGFK;GISAFIVPKPIK;IAMMTLDAGR;LVIAGQLIK | 0.1299 | 15.2416 | 5 |
| MICOS complex subunit MIC60 | AAAKPWLDAAR;GDTDLLLVYTLK;IQYLQNQLAELQTVR;RSQSLWAAAEALLAATR;YVNLLQGAPR | 0.0894 | 21.4376 | 5 |
| SFRICE_004002 | AGAGVDNIDVATAGQR;AVIATPHLGASTK;DELLQEIPK;IIGYDPFVTAEQCASFHATK;VGQEIAEQLVNLVKPGTYNTPLAEVTR | 0.2615 | 21.4839 | 5 |
| SFRICE_013101 | DTLLYIYTSGTTGFPK;IFSTIYPLTLVK;TGEYVPLTQK;VLLYTIFR;VTTALINTNLK | 0.0862 | 20.8456 | 5 |
| SFRICE_008237 | KPLFQDTWLAGDR;LHLLLTK;NLILWFR;QWEIDNLLR;VVMGTFISQLR | 0.0887 | 21.9188 | 5 |
| Transaldolase | ISLNEAQFR;LLQELAASEQPLKR;TYEGKDDPGVLSVTR;VSVEVDAR;VYNYYK | 0.1566 | 17.5536 | 5 |
| SFRICE_005734 | FSPAYNNK;LNTAPVIMHFPAK;SIVSLNLNK;TQGYPYSFLFK;VQQLTDLTVKK | 0.1566 | 22.444 | 5 |
| Succinate dehydrogenase [ubiquinone] iron-sulfur subunit, mitochondrial | DLVPDLTNFYR;LKDTYSVYR;QYQSIEPWLQR;WNPDEPDK;YLGPAVLMQAYR | 0.1809 | 18.0047 | 5 |
| SFRICE_032051 (Fragment) | AHANIPDDENVK;LLIGFVQGK;SLTEEDLWALNPQDSSTEVVPK;TALTSAIYR;TSASVNFKPESEK | 0.1303 | 23.7492 | 5 |
| Cytochrome b-c1 complex subunit 7 | ALQLSLTK;EEWTKWEEDQLYLTPIVDQVK;LPQHVVDER;WAYNLSGFNK;WEEDQLYLTPIVDQVK | 0.4444 | 18.6499 | 5 |
| SFRICE_002195 | AADINVAEGKR;AAILESEGVR;ILEPGLNLLWPIVDK;LIGGALAQR;SELGQISLDK | 0.1319 | 15.6372 | 5 |
| SFRICE_016401 (Fragment) | AYGAEVPDSFDTLGVAVNK;RSEPSVVAIVYPFTADHK;SEPSVVAIVYPFTADHK;YALDVEAITTR;YYFGNK | 0.0863 | 18.9634 | 5 |
| SFRICE_017305 (Fragment) | AEDTEEKDFHVTK;DSFTAAAQSYR;EAAAATINEWANEK;SIDTATLK;YLGGADFETAYK | 0.1355 | 20.1052 | 5 |
| Glutathione peroxidase | APFAPPLDSAGFSYGFNSLFLPK;EFYELIPK;HNLFFQPDSILR;IPTDVPTWGIK;VLDFWQK | 0.0847 | 19.4513 | 5 |
| SFRICE_024866 | GTNVYGILR;HLVADESWLATLSR;LGIALESILR;SLNNLLER;TSSLEAIVVSVPYR | 0.0762 | 16.3951 | 5 |
| NADH dehydrogenase [ubiquinone] flavoprotein 1, mitochondrial | GEFYNEASNLQVAIAEAYQAGLIGK;GGAWFASFGR;GNDWIVNELK;HAGGVIGGWDNLLAIIPGGSSTPLIPK;IFTNLYGR | 0.1660 | 20.0585 | 5 |
| Staphylococcal nuclease domain-containing protein | EVIFTADKPPNSATR;GIWGPDLQDHVR;LVELEDVAK;QDGESEPYAEEAR;VIALSGITAPK | 0.0671 | 18.9910 | 5 |
| SFRICE_002773 | DAGAIVVASTNLPELLVWQETR;IIAGDKAPLLK;ISQETTTTENAPEPYYHR;NEVAEEDSEAVKR;TGEDQETMLSLGFISK | 0.1530 | 21.5789 | 5 |
| Butyryl-CoA dehydrogenase | QSVFDFAQK;SYLYSVAK;TDPSTKPQHGISAFLIEK;VALDAIQILGGNGYINDYPTGR;VPAANLLGEENK | 0.1667 | 19.6508 | 5 |
| SFRICE_026071 (Fragment) | LGQLTGANLDEIFSLINTDQESSK;NPFLAQITVNR;TALSHYVEITALPR;VPVFIR;VYVTHLLEK | 0.1031 | 18.1499 | 5 |
| Succinate--CoA ligase [ADP-forming] subunit beta, mitochondrial | DALLIEVNPYAEDAITGK;FHVATTADEVVK;FRFDDNADYR;LIAESGLR;NLNIQLPVIVR | 0.1305 | 14.7737 | 5 |
| SFRICE_022541 | ALLPGTVVAVR;ALQLYNVYR;LGITSLIR;LLLGPLGVK;TLASAYAALAPLLAELR | 0.0328 | 15.2247 | 5 |
| SFRICE_002594 | ALDAAADAFQTWK;FLIHEDVYDQFVEGFK;FLIHEDVYDQFVEGFKER;GVINVVTCSR;VALELGGNAPFIVFPSADIKR | 0.1281 | 18.0606 | 5 |
| SFRICE_001226 | AADVVLAAHLASEAALR;KTDEVYQLK;LAGQIVNR;LIEPFQVLYERPGELVAQFK;TIAEDLVVTK | 0.1689 | 20.4536 | 5 |
| SFRICE_024301 (Fragment) | ACDDVISGK;LMPGLTALR;LYGSQTLR;SIMNFPIGGIEER;VNMEFGLDK | 0.2172 | 18.4958 | 5 |
| Tubulin alpha chain | AVFVDLEPTVVDEVR;EIVDLVLDR;FDGALNVDLTEFQTNLVPYPR;LIGQIVSSITASLR;VGINYQPPTVVPGGDLAK | 0.1711 | 20.5182 | 5 |
| Cleft lip and palate transmembrane protein 1-like protein | EFDEIIR;ERPHLEGSTADSVSK;FDYLNPATFDIK;FLPILQHNVLK;VSFSGWR | 0.0965 | 20.1396 | 5 |
| Multidrug resistance-associated protein 4-like protein | DLAAAAHAEHIK;ILVTHQLHHLK;LLVLDEATANVDPNTDALIQK;SSLISALFR;SVTSLSSAVQSGGSNFSAGQR;YDKDADPVLK | 0.0617 | 19.3060 | 5 |
| SFRICE_002878 | AAFDDAIAELDTLSEESYK;DICSDILGVLDK;DSTLIMQLLR;EAAENSLVAYK;IISSIEQKEETK;NLLSVAYK | 0.2748 | 18.9994 | 5 |
| SFRICE_013294 | FVSEAMAR;KYPDQPLFNPIPLLDK;SADLVVEAIVENISVK;YPDQPLFNPIPLLDK | 0.1278 | 16.6878 | 5 |
| SFRICE_011011 (Fragment) | LAPPLVTLLSAEPEVQYVALR;LASQANIAQVLGELK;RPADTQELVQHVLSLATQDSDNPDLR;YNDPIYVK | 0.1004 | 17.6941 | 5 |
| SFRICE_002919 (Fragment) | ADFAQAILDAGLR;AGNVVHLYER;FIGPSPFVVQQMGDK;SVAIYSEQDKLQMHR | 0.1636 | 17.0090 | 5 |
| V-type proton ATPase subunit G | AQQEAVIK;AQQEAVIKDILNLVYDIKPELHTNYR;DILNLVYDIKPELHTNYR;EEAQDEVEKYR | 0.3162 | 15.1670 | 5 |
| Uricase | FELSDHGYGK;TADFDNAWLTVK;TTQSAFVDFVQDEYTTLTDAAER;VPEINWIK | 0.1573 | 18.9994 | 5 |
| SFRICE_003059 | KYDAFLASESLIK;LLGPGLNK;NFLETVELQIGLK;YDAFLASESLIK | 0.0926 | 17.1689 | 5 |
| Cysteine desulfurase, mitochondrial | AIGTDEDLAHSSIR;HVITTQIEHK;ITYLPVK;VRVEPIQSGGGQER | 0.0987 | 11.7366 | 5 |
| Coiled-coil domain-containing protein 47 | LHKPLLDDNFTLVGETGSDVVTASSDR;NTSIATNFLK;QDLVHVLLGVVRPTPDTLLVR;RQDLVHVLLGVVRPTPDTLLVR | 0.1105 | 18.9994 | 5 |
| Proteasome subunit alpha type | AINQGGLTSVALR;ATDANAYLEK;GTDAAVVAAQR;LLDPASVTHLFPLTK | 0.1992 | 18.9994 | 5 |
| SFRICE_014997 | DVLSVAFSVDNR;FSPNHANPIIVSAGWDR;IWDLESK;YPDMILSSSR | 0.1442 | 13.9177 | 5 |
| SFRICE_034158 | ELTQALLDELAEDKPVR;GVTSLVTALK;HYTPLLAAFAQSAK;LLEFLPLNR | 0.1767 | 17.9580 | 5 |
| Annexin | AIFQEYQYLTGHDIDDAIK;SEDFHVDAEK;SEIDLGDISDAFQAK;SLLQAGELR | 0.1073 | 16.8290 | 5 |
| SFRICE_037205 | AAADAVQIFGGNGFNTEYPVEK;IYQIYEGTSQIQR;KTFGVPIAR;TRPPVAAGATGLAQR | 0.3207 | 14.5252 | 5 |
| SFRICE_010679 (Fragment) | ALQLWSR;GLEIAQTNAAFLLDNGEVR;LGDYHYYGLGTQQDLEAAAYHYR;LPAALALAR | 0.2601 | 15.4197 | 5 |
| SFRICE_032134 | EKLPGAGGVTTPGAVFK;GWESGADVQNTKPNK;IIIADIDDDKSLK;VTGTNPGYGATVVALLMAAHTILK | 0.1725 | 15.4054 | 5 |
| Peptidyl-prolyl cis-trans isomerase | LETDLVPK;MESLGSASGKPTK;VFFDVTADGEPLGR;VFQDENFK | 0.2299 | 17.6941 | 5 |
| Cytochrome c oxidase subunit 2 | IILPMNNQIR;IMVTATDVIHSWTVPSLGVK;LLYLLDELNNPLITLK;NFINWINNYS | 0.2467 | 16.9213 | 4 |
| SFRICE_012915 | KQQDDYAIISYK;LLGIDLEK;NLNLSR;VIGYADGEREPIDFPIAPSVAIPK | 0.1263 | 14.3808 | 4 |
| SFRICE_026609 (Fragment) | AYLPVNESFGFTADLR;DSVVAGFQWAAK;EGLPDLNQYLDKL;EGVMAEENLR | 0.2134 | 13.6925 | 4 |
| 3-hydroxyisobutyrate dehydrogenase | KGALLIDSSTVDPNVPK;QFHCGEVGSGQVAK;SPIPLGAVATQLYR;VLLDVLNNSSAR | 0.1781 | 16.1788 | 4 |
| Phosphohydroxythreonine aminotransferase | ASIYNAVSVEEVETLVK;GAEALGLIQLK;IYNFGAGPAK;TGTADYVVTGDWSAK | 0.1456 | 18.9994 | 4 |
| AP-2 complex subunit alpha | ESSILAVLK;GLAVFISDIR;LVNLFPEIK;VGGYILGEFGNLIAGDTR | 0.0496 | 17.9580 | 4 |
| Poly(U)-specific endoribonuclease | ANYLGYIDKLDLSGK;KIWFTK;NVIRPEFVTPNEETEQVTFINTILATGPVR;SLMNFLVSK | 0.1386 | 15.3029 | 4 |
| Adenylosuccinate lyase | GTTGTQASFLQLFK;HGLDLLLPR;KVDLEVIAALSGLGATVHK;VAELAGFDKR | 0.1179 | 17.1689 | 4 |
| SFRICE_005530 | AQAAIAVEVAEEIVK;AQSELNSAANEK;HVPTLAVLRPGIVTVTENDGK;IFVSSGTVTVNDDSSVQVLAEEAHPLENLDR | 0.4907 | 17.6941 | 4 |
| SFRICE_037014 (Fragment) | ITSVWYK;VAGGGIIFAR;VANVFSAFR;VFAATSSIIFR | 0.0987 | 17.4378 | 4 |
| SFRICE_012829 | IQDAILEK;TDLNNLQVLLINNPR;VIHPELTSR;WISAHSSPK | 0.0501 | 16.1788 | 4 |
| SFRICE_002689 | HVVITGGSSGIGK;LGAHVTVIGR;TDPDVIK;VQHVALDVTSDYK | 0.1307 | 16.4611 | 4 |
| SFRICE_021822 | DADAGFATVEESHIGK;EHFVDLIAK;GLLLLHGDEWK;IVPFTYLPFGLGPR;YANDVIASCAFGLK | 0.1208 | 18.9994 | 4 |
| SFRICE_013539 | GISNEFVNK;ITFNVNHTVDSDDLEGEVQPEK;LLESVSQFTIGK;SRPQFEVDLVR | 0.1856 | 17.4378 | 4 |
| T-complex protein 1 subunit eta | AFEAIPR;LKPFVEEGVHPR;LLDIVHPAAK;VSGGALEDSFLVSGVAFK | 0.0897 | 14.6454 | 4 |
| SFRICE_012577 (Fragment) | DAIDAGYR;REDIFITSK;SIGLSNFNKK;SKPGEVEQAVK | 0.1258 | 13.5721 | 4 |
| SFRICE_003905 | ELATTLK;ESSGMYTFSAPTSGR;RFFEVQR;TVKHEQEYMQVR | 0.1981 | 14.6252 | 4 |
| SFRICE_000871 | FASAITSNEAGK;LFDFTK;NVEQALQSLR;VAIVTASTEGIGYAIAK | 0.1607 | 14.8945 | 4 |
| SFRICE_011249 | ATLEVILLR;GTNLNDAGDNVAQNEVTK;LTNPSNDTVLFK;VEIAITPQPVYVDPNEK | 0.2014 | 17.6941 | 4 |
| T-complex protein 1 subunit gamma | AVAQALEIIPR;DVLNEIER;GIWEPLAVK;TEELKESDVGTQAGK | 0.0798 | 12.5968 | 4 |
| SFRICE030301.2 (Fragment) | ANNGQVTYESIK;FFDNLVK;GPKPVALFGNIK;LTEEIDESLK;NDFMDLILALR;NTIVLISPR;VPGTDLTIEK;YLNLSLFPTSIK | 0.1861 | 14.3828 | 4 |
| Sulfite oxidase | ATDSNFNTQPER;LPDLPTFR;LQVAHFDTPNELFFVR;VVVPGAPAVR | 0.0821 | 11.4406 | 4 |
| SFRICE_013145 | AGVVGMTLPIAR;GLVGLVTGGASGLGR;NALQTTVDK;TIEVNLIGTFNVIR | 0.1961 | 12.1351 | 4 |
| SFRICE_000926 | AYPTIMFIK;ETHPVFFGYVGK;LSQADSLGYLK;YQPHTWFYAMSHELLK | 0.1129 | 15.2817 | 4 |
| SFRICE_021907 | ASFCQTFAEFQAPTGEWK;EWVGYGFNGQPNYVDRPDFPMPAVR;IFVYSPLPESLSEENQK;VNPIDGLASK | 0.3933 | 15.2265 | 4 |
| SFRICE_025541 | DLPHALDEHGLIER;EKDNIDITLQWLIAHSK;GVNAIVYMVDAADPDKIEASR;QQLTGIPVLVLGNK | 0.3529 | 16.5070 | 4 |
| SFRICE_030420 | APHLDALSFR;ASGLETHPELGGLLVNAELQAR;EAAPLGAVLPPYLAAEAAR;ELWREPDLAAR | 0.0960 | 14.7062 | 4 |
| UV excision repair protein RAD23 | AVIQQNPNLLNAVLQQIGQTNPALLQAISQHQSAFVR;ILLDDDKLSTYNIDEK;LKIEVEK;SSDVPASTTSAPEAGESASTESGDGKPK | 0.2794 | 16.4101 | 4 |
| SFRICE_015797 | ADYMGIPFK;DIDHTSGSATVTAGGVGFSFANIR;EVFYSDPGQQIIK;VKEVFYSDPGQQIIK | 0.4364 | 15.6302 | 4 |
| SFRICE_013406 | NAEYDQFLEDLQK;QIDVETVGER;TTYEEIKK;YIQATDLSEASQEAVEEK | 0.3245 | 17.4378 | 4 |
| SFRICE_008822 | FLDTYEK;IIDYLIDYK;LVPGENKIER;TFFQFLQK | 0.0491 | 14.6623 | 4 |
| Dynamin-like 120 kDa protein, form S1 | ASVEQQADAFK;EWAESELPNASIR;TIFVLTK;VLQLNALQDR | 0.0518 | 15.8761 | 4 |
| SFRICE_000655 | AITSAYYR;ELRDHADQNILIMLVGNK;GAVGALLVYDIAK;NEFNLESK | 0.176 | 17.5476 | 4 |
| SFRICE_034460 (Fragment) | AALDHFTR;IFDDVFR;LDVLVNNAAIGAR;LVSLELAPK | 0.1814 | 15.0065 | 4 |
| Alkaline phosphatase (Fragment) | LQSLAALALDANK;QFWLDLAHNELEEALK;SAGFVTTMR;VTHATPSPLYAHSASR | 0.108 | 15.3898 | 4 |
| NADH dehydrogenase [ubiquinone] 1 beta subcomplex subunit 10 | ALYNTIDGPVTYFR;AVDSEILGILR;DRAVDSEILGILR;YGDLGAYGDAR | 0.239 | 15.1392 | 4 |
| Adenylate kinase | APVAPAVK;EEVASGSELGR;LDVLLAK;NGFLLDGFPR | 0.1457 | 13.9398 | 4 |
| SFRICE_003127 (Fragment) | DLEDLFDQLK;GFPWLGGSER;HTTGIHLWSQPFK;SFVVTQAEPALK | 0.0809 | 16.5031 | 4 |
| UDP-glucuronosyltransferase (Fragment) | HWVQHVVNTR;LIVPQIQK;QTVIWK;SIGGYHIEEEVKPLPEDLEK;SPALAVPLYQR | 0.1757 | 16.1324 | 4 |
| SFRICE_031534 (Fragment) | AHVHYVVTEQGIADLFGK;AYELIK;MFSGFGGQVDFIR;VGAGVVTSR | 0.2500 | 15.4617 | 4 |
| Dihydrolipoyllysine-residue succinyltransferase component of 2-oxoglutarate dehydrogenase complex, mitochondrial | EFYVNNGDTVK;GLVVPVVR;HAQSIETASVK;LELTEGGPPPK | 0.0874 | 13.0309 | 4 |
| SFRICE_024442 (Fragment) | IKLEQAEFGYFLSR;LEQAEFGYFLSR;LQLTAALR;TNVVQSLLAR | 0.1103 | 16.6741 | 4 |
| Electron transfer flavoprotein subunit beta | EIDGGLEVIK;EYETLQPLHVAK;LPAVVSADLR;TGVVTDGVK | 0.1621 | 16.3089 | 4 |
| ER membrane protein complex subunit 4 | DTDSNLLLIK;KLWDVALGPLK;VEHIGGGLSLI;WALDFNQK | 0.2326 | 18.9994 | 4 |
| Ubiquitin-activating enzyme E1 | DEFEGLFR;ESYANPEFLIADFGK;KPLLESGTLGTK;NVILGGVK | 0.0413 | 14.7566 | 4 |
| SFRICE_034364 (Fragment) | GYDTPILNK;SGEFASAFFK;TKEEFIDSEGR;VGAPTSGTDITLLDWDEGGYR | 0.1342 | 16.2580 | 4 |
| Prophenoloxidase subunit 1 | DLDRPVDQIR;FAGTTIR;NTLGTFWELSTVDLGR;VSSVSLEGAAGR | 0.0658 | 14.6240 | 4 |
| 40S ribosomal protein S2 | ATYAAIAK;GTGIVSAPVPK;LSVLPVR;TYAYLTPDLWR | 0.1418 | 16.6878 | 4 |
| Ribosomal protein L15 | INWIVNAVHK;QGFVIFR;SQGVNQLKPTR;YIQELYR | 0.1716 | 16.5358 | 4 |

Table S24 Identified *Spodoptera frugiperda* larval coded proteins associated to HvAV-3h virions (Sf-2)

| Protein ID | Identified peptide sequence | Sequence coverage | Protein Q score | Unique spectra |
| --- | --- | --- | --- | --- |
| SFRICE_003260 (Fragment) | AAAGLLDLPNLPR;ADFELEAR;AHMLFIDAELAYPTSTGLPLK;APAYGADVDLYLNYEK;AQLLGEYVYSK;ASNTNLEKDIINYEGQVELK;DKHNIPISGQVVEEIGGGK;DSAENFNVITK;DTQVLQSSMNVETK;DVTQSNDKYSGHAEIVLSDAAK;ESFELLFDAEGAK;EVYLGVGALAGAFCR;FDADIDPK;FDIGAIETTTLYSSHAK;FEIYAVPLK;FLVNTPLVK;GFDLQVGLPIDK;GGAASIISYK;GHFEYEPVMR;GSYGDDTNFELAGVLQAK;GVETFFNMAIGER;HLEDALIDK;HNIPISGQVVEEIGGGK;HQSPPGIDKPIR;IDMSKPGVITNVVSAGFDLR;IKFDADIDPK;IKGSYGDDTNFELAGVLQAK;IVSTVLYVQPENK;KADFELEAR;KHQSPPGIDKPIR;KIDVDVEGQYQDK;KLEFSVHSK;KNDVNIEFQNR;KPGDYSIK;KPSELTVVVK;LDQDGDKSDNHVELK;LEFSVHSK;LEVATNIDIR;LLVPHVDPVVLDVNYK;LNPFINFR;LPQYTLDLHINKEDK;LVFFSLSNAR;NDLVTGVFNPSAEIK;NEIDLIYGGDLR;NGVEVFAK;NLLDTEPVHQVGNFISTSLR;NNNDKIHFSTNTK;NPSPDTYISNVVVDADGR;NYQVEIASK;QEIVTASNDLVYFYAEK;QTAVAEFGFNHPK;RGGAASIISYK;RLEFHATNDNK;RVFLDGLIR;SEVDSFDK;SGITIQLPQR;SNLENYIDFK;SSTQVDIFGTCPTEVSSSQEGSAVLVHR;TEMGALLAFNSPMK;THGMIEIVLPTER;TKVEEDNVVAVLK;TPNKFNTDVR;TTNKDDLMNVYSHVK;VAATEEYLYRPFSVGENGAR;VAEHLFGPR;VEEDNVVAVLK;VEEDNVVAVLKGIR;VFLDGLIR;VHGSVLHPQQNAIK;VVFGQMLK;YDANELYGK;YDVTGNLLPKPVSLVASGTYVDSLTESDDKYR;YELSGVLLHK;YHMHVYNTPEFGNAK;YLDDFTVNVR;YLSSYPLTGK;YNYGVEGTVTVFLTGASNQETSVK;YSIENIK;YTSPSTPKPSR | 0.3999 | 327.4001 | 100 |
| SFRICE_015484 (Fragment) | ALVLEDK;AVIMLGDK;AVIMLGDKK;AVVVNGQHFFTFDGR;DLDNLEAR;DNFNYFYSALR;DNGQVTLNGANHGFPVIEK;EAVHQACDLAR;EKYQELAIPEQALALLGDAYNTIR;EMPIFALLK;EYLDSLIEVVAR;FPYPIIYDTDQR;HKPELEELTNALTAIFK;ISTSESEFGNAYR;LSIVGHVQPEKR;MLEEVLNSTKPLVDNILK;NFKDIVVPLVGQVLDGLK;NMVHGALFLDDSLNLK;QADIVVAFETTKDNEK;QFLSTAAAAINQR;RVDVSADLILGGEK;SFNPLDEVPAK;SYNDDLAK;VDVSADLILGGEK;VDVSADLILGGEKK;VQFDDESR;VYLVGITPK;YLYEVASIVDALR | 0.3276 | 112.2809 | 35 |
| H(+)-transporting two-sector ATPase | ALDDFYEK;ASLAETDKITLEVAK;DAMGNVLYQLSSMK;DINELTQSIYIPK;EASIYTGITLSEYFR;GNEMSEVLR;HFPAINWLISYSK;ITLEVAK;LAEMPADSGYPAYLGAR;LASFYER;LLKDDFLQQNSYSSYDR;LPANHPLLTGQR;NIIAFYDMSR;NYPEFVPLR;TALVANTSNMPVAAR;TGKPLSVELGPGILGSIFDGIQRPLK;VGSHITGGDLYGIVHENTLVK;VGYHELVGEIIR;VTDVVLETEFDGEK;VTDVVLETEFDGEKEK;YSNSDVIIYVGCGER;YTMLQVWPVR | 0.4448 | 88.6717 | 32 |
| Protein disulfide-isomerase | ELIAANNVIIFGFFSDSNTAK;FDEDLFK;ILEFFGMK;LGEHFEKDDDVIIAK;LIALEQDMAK;MDATANELEHTK;NGSPIDYTGGR;QADDIITWLK;QADDIITWLKK;QHLLSEDLPADWAAKPVK;QLAPIYDK;TFTSVASAVDDHVFALVSDPK;TGPPAVEVSSAEQAK;TLVATNFDEVVFDNSK;TLVATNFDEVVFDNSKK;VDATQEQELAESYGVR;VMSVAIDADEDEHQR;YDAEKFDEDLFK;YHLLLFLSK | 0.4433 | 79.0872 | 30 |
| ATP synthase subunit alpha | AAEISTILEER;APGIIPR;AVDSLVPIGR;DGQITPESDAALK;DGQITPESDAALKK;DNGKHALIIYDDLSK;EAYPGDVFYLHSR;EFTQHIK;EVAAFAQFGSDLDAATQQLLNR;GHLDKLDPSK;HALIIYDDLSK;IVTDFLATFTQAAQ;QMSLLLR;STVAQIVK;TALAIDTIINQQR;TGAIVDVPVGEQLLGR;TSHQGLLATIAK;VGIKAPGIIPR;VVDALGNAIDGK;VVDALGNAIDGKGPIDTK | 0.3609 | 82.8583 | 28 |
| Heat shock 70 kDa protein cognate 4 (Fragment) | APAVGIDLGTTYSCVGVFQHGK;DNNLLGK;ELEGICNPIITK;FELTGIPPAPR;GEDKTFFPEEVSSMVLTK;HWPFEVVSDGGKPK;IINEPTAAAIAYGLDKK;ISDSDKQTILDK;KFEDATVQADMK;LLQDFFNGK;LSKEEIER;MKETAEAYLGK;MVNHFVQEFK;MVNHFVQEFKR;NQVAMNPNNTIFDAK;QTQTFTTYSDNQPGVLIQVFEGER;SQIHDIVLVGGSTR;STAGDTHLGGEDFDNR;TFFPEEVSSMVLTK;TTPSYVAFTDTER;TVQNAVITVPAYFNDSQR;VEIIANDQGNR;WLDSNQLADKEEYEHK | 0.4456 | 77.2932 | 27 |
| Mitochondrial 60 kDa heat shock protein-like protein | AEQIRDQIQETTSEYEK;ATLSDMAIATGGVVFGDDANLIK;DDTLFLK;DKFQNIGAK;DQIQETTSEYEKEK;DRVNDALNATR;IGLQVAAVK;ISNVQTIIPALELANQQR;KISNVQTIIPALELANQQR;KPLVIVAEDVDGEALSTLVVNR;LVQNVANNTNEEAGDGTTTATVLAR;RGVMLAVDAVK;TANSDQATGVEIVKK;TLVDELEVIEGMK;TLVDELEVIEGMKFDR;VEDLGPEFGYDALNNEYVNMIEK;VEFQDALVLFSEK;VEFQDALVLFSEKK;VNDALNATR | 0.3874 | 74.7673 | 26 |
| SFRICE_019251 | AKADILYK;DFLAGGISAAVSK;EQGLLSFWR;GFGVSVQGIIIYR;GTGGAFVLVLYDEIKK;IPKEQGLLSFWR;LAADVGKGDGQR;LLLQVQHVSK;NTIHCWATIAK;QVFLGGVDK;SDGLIGLYR;TAVAPIER;TEGTGAFFK;YFAGNLASGGAAGATSLCFVYPLDFAR;YFPTQALNFAFK;YFPTQALNFAFKDK;YKGIVDAFVR | 0.6033 | 72.0295 | 25 |
| Dolichyl-diphosphooligosaccharide--protein glycosyltransferase subunit 2 | ALHFDGGLPATATLLAAIHR;AQYGLYK;DKYDKPVLVQQAFVR;IALSAGSK;KDATIADIYHAVFTLK;KPSPLTEEQK;LADDVVVLSK;LLKPDEVLAQSGTR;LSMFDTAR;LTDKLQADHLQK;QPLTQKPNEPTTFVLNLSK;VASVAGDETIFVAEPDNTK;VSTVEVGVGDVDGTTSPK;YVAPLAALTFLAGHR | 0.3081 | 54.2915 | 23 |
| NADH-ubiquinone oxidoreductase 75 kDa subunit, mitochondrial | ALAQSLQKPVSGK;AQQTLAAVSPPGK;ATYVNAEGR;DLASGTSSHEVMK;FASEVAGVDDFGTTGR;FEAPLLNAR;FTDIHFSGK;IDSVDVLDPLGSNIVVATR;LLAVAGDLADAESLVALK;LPYDSLDEVRDR;LSQISPALVTYGNVEDNNYFAQAR;RIDSVDVLDPLGSNIVVATR;RPVVILGADQLK;TASQVAALDIGYKPGVGPVLQDGPK;TNEEINEEWLSDK;TNEVLR;TPEGAAILAYTQELALR;VDLTYDYTHVGDSASILK | 0.3384 | 71.8576 | 20 |
| SFRICE_011490 | APPVLEVK;GYDVSGSVTSFGSPIR;HFTIEFEPK;HNVAVTQDHTNVPTFEHTGYVLR;LEPGPGPHNPGLMHVLPR;LEPSQAVVQVK;LIGIQPR;LLESLAAAAAK;LYLTADSSQAALYSAR;NAFEVTGFTVVGSVVK;QVTDGSVVVYTSNPTHYR;SVGGPGLSGAR;VITAGQNQGPSGIAVQLINDK;VITAGQNQGPSGIAVQLINDKGDVR;VSSPVEVDDLVLHVVTDGAPPK;VSVGNGPVSGVTFSQLK | 0.1939 | 67.6253 | 19 |
| Voltage-dependent anion-selective channel protein 3 (Fragment) | ANDVFGK;DFNLHTNVDNGK;DFNLHTNVDNGKDFGGSIYQK;DYGLTFTEK;FALDADASLHAK;GYHFGVFK;GYHFGVFKLDLK;LRPGVVLTLSAAIDGQNFNAGGHK;NNFALGYQSK;SETGVEFSSGITSNQESGK;TKSETGVEFSSGITSNQESGK;VFGSLSSK;VTLEGTFAPQTGSK;WNTDNTLATDITIQDK | 0.4724 | 59.9606 | 19 |
| SFRICE_001118 | EADYATPLYTPQNR;GLCTGDKYPILR;LDSESEISGVPPIHADGAGEAGPYTK;LPDDNGDPGLWVSYEDPDTAGQR;LVLGIPTAAR;MVSLNENLDVDR;NLGGVSIVDLSMDDFR;NPLLNADAVVNHWIQGGAPAHK;STWGSIWHGIK;TAFVNSGVLLAEQHGFDGIDLAWQFPK;TKYPGLK;YNLLLESPQAR | 0.4401 | 52.4334 | 18 |
| SFRICE_001000 (Fragment) | AEITPVTLTVK;DEVDNFALR;EGLVTAGTASGISDGAGAIVLAGEEAAK;ISVAGGVENMSQAPFAVR;ITAHLVHELR;ITAHLVHELRR;KLPPVFK;LNVNGGATALGHPLGASGSR;NTTATELQTAATVAALK;RDEVDNFALR;RTAFGTFGGVFR;TAFGTFGGVFR;VDSIVVGQVMTASQTDGIFIPR;VEVDEHPRPQTTIEGLK | 0.4447 | 51.9401 | 16 |
| Dolichyl-diphosphooligosaccharide--protein glycosyltransferase subunit 1 | ALLPYPTAITQQEDQLVK;DAINSGATPLVTVETVFTK;IILPEGSTSVK;KNVVENHIQDFQLR;LIEVSHWGNIAVEEVIEVEHTGAK;LVDISSQLVK;LVETTVK;NNVAFVGAK;THYTLGYNVPSYEYLYQSGSDYLLK;TSVVLNTK;VNLPYPVTR;YDYQQDHHSGPASVR;YIGNLYTYSPYYITSMK;YPLFGGWR | 0.4136 | 50.5240 | 16 |
| Malate dehydrogenase | DDLFNVNAGIVR;DLTQCIAVHAPK;GLKGEENVVECAYVK;GPDELPSAIADSHVIVIPAGVPR;IALPELK;IALPELKK;LNAYEEK;SDLTDAK;VLGYKGPDELPSAIADSHVIVIPAGVPR;VMVAGAAGGIGQPLSLLLK;YFANPICFGK | 0.3460 | 40.4389 | 16 |
| Pyruvate carboxylase | AEELSFPK;AGTVSSVAITNGQK;EANLLLGDIIK;LDTNIALQTVSEYSAYWEQAR;LQVEHTITEEVTGIDLVQSQIR;QELTGENTAVQPPK;SVFIRDENASK;TFLVGPTVGETIEVK;TNIPFLLNVLENQK;VISHAHDLPSSAAK;VTTEDPANNFQPSTGR;VVGDLAQFMVQNK;YYLNLADELVK | 0.1983 | 53.7064 | 15 |
| Enoyl-CoA hydratase | ELEAVIPK;EVNQEAVNILK;HAILATNTSAIPITK;HPGTSDDTAAAAVGVGLR;IVNGVYVVTLDSPNTK;LPALTSVPTTLDLALTGK;MQLLEIIR;TGFGLPEVMLGLLPGGGGTQR;TGVDKGPTAGYEAEAQGFGELAVTPQSR;TLDSLTK;VIGMHYFSPVDK;WVDQFGADK;YLETVAIQVAR | 0.1520 | 48.2163 | 15 |
| Heat shock protein 83 | ALLFVPR;AQNYTFQTEVNR;EIFDLEENEQQKER;KPNEVNDEEYTQFYK;LIINSLYR;LLHIIDSGIGMTR;LLSLTERDVLDANSDLSIR;NKEIFLR;QFEPLTTWLGNK;QHVWESDASAFSVAEDPR;RGTHITLHVK;SGTAEFLSK;TDAEAVLR;TDHIKLYVR | 0.2048 | 52.4888 | 15 |
| Dolichyl-diphosphooligosaccharide--protein glycosyltransferase 48 kDa subunit | ETHSQFFK;EYPHAVGR;FIDDGGNLLMAGNAAAGDVYR;GYTLTYK;KTVLIAALQAR;LADDPNLVLSK;LSQWAFGER;LYHATQVSVRPLQHTQYER;TVLIAALQAR;VPDVWGVYQFK;WQPFDANDVQLEFVR;YGEYLYK | 0.2926 | 40.8709 | 15 |
| Citrate synthase | ALGLPIERPK;ALGVLAQLIWSR;EMNYYTVMFGVSR;HLPNDPLFK;KQVGDNFTEEGLK;LHPMSQFSAAVTALNSESSFAK;LPVIAATIYR;LVAAVYK;NPWPNVDSHSGVLLQYYGLK;QVGDNFTEEGLKEFIWK;VVPPILTELGK | 0.2839 | 44.8129 | 15 |
| SFRICE_000420 | ADVDIAVK;AGTVWVNDYNVLSNQVPFGGYK;ANNSEYGLAAAVFTQDIDK;ANYVVQR;ENGPYGINNYTEVK;ILSLIESGK;ILSLIESGKK;ITLELGGK;LADLVER;LGSPWR;TENPATGQAIADVQQAGK;VVGDPFKPETEQGPQIDAEQQNR;YYAGWADK | 0.2947 | 48.8523 | 14 |
| SFRICE_010943 | DLGLFGLYK;FGISLPR;FITSEDFVR;GVLIQILESTYR;IVYNDLNSITPEQYFK;LFFVDFGGSRPAGSELHVATPIEESVK;LPFNMESTFVR;LTVNDFVR;SGQTTYNGVIDAAR;SSPQFGVTLVTYEILQR;TGSFIGEVAYR;VINASGFPHGER;VYLNATNGTR | 0.2373 | 58.0312 | 14 |
| Heat shock protein 83 | ADLVNNLGTIAK;ALLFVPR;DQVANSSFVER;EDLTEYLEEHK;EDLTEYLEEHKIK;EGLELPEDEEEKK;EKYTEDEELNK;ELISNSSDALDK;HLEINPDHSIVETLR;NADDITQEEYGDFYK;SLTNDWEDHLAVK;YESLTDPSK;YESLTDPSKLDSGK;YYEQFSK | 0.1994 | 56.9775 | 14 |
| SFRICE016243.2 (Fragment) | AKFEELNMDLFR;EWSDSTVQHDVK;FDLTGIPPAPR;IFAPEEISAMVLTK;IINEPTAAAIAYGLDKK;ITPSYVAFTADGER;LTPEDIER;MKETAEAYLGK;SLEDVVQPIIAK;SQIFSTASDNQHTVTIQVYEGERPMTK;VEIIANDQGNR;VLEDADMNKK;WLEDNQDVDSEEYKK | 0.2640 | 40.7766 | 14 |
| SFRICE_008643 (Fragment) | DFTDPLVVAYYDVDYVK;ENFHGLVGIR;EQTEVTFAVSDKDDFTHELNEYGIDYAK;FEDSSVVYK;KGEVSQEYNGPR;LKPEYAVAAGVLK;STCEQFSVSGYPTLK | 0.3133 | 30.7669 | 14 |
| SFRICE_024780 | AVVGPDGNVASNYGVIDPR;EGYVLTETGQIFINSGAIDPK;EVQVVAITGK;IQTDKELINTK;LDASHGHVDVSR;NLIDPTLALLDSER;SGHILFTSGVIDPK;TAVGIIDPATGEIVVTDPK;TGHLIVSSGVVDPK;TGNILITTAVVDPK;TGNILLLSGVVDPR;VWVDLEK;YVNLVIVTSK | 0.0622 | 51.1944 | 13 |
| Vacuolar proton pump subunit B (Fragment) | AVVGEEALTPDDLLYLEFLTK;FSEIVQLR;GPPILAEDFLDIQGQPINPWSR;HVLVILTDMSSYAEALR;IPASILAEFYPR;IPIFSAAGLPHNEIAAQICR;IYPEEMIQTGISAIDVMNSIAR;QIYPPVNVLPSLSR;RIPASILAEFYPR;TVSGVNGPLVILDEVK;TVSGVNGPLVILDEVKFPK;VFNGSGKPIDK;VFNGSGKPIDKGPPILAEDFLDIQGQPINPWSR | 0.3591 | 52.3090 | 13 |
| 3-oxoacyl-[acyl-carrier-protein] reductase (Fragment) | AAAAPQQDLLHAVANILGIKDPSK;EPFRPDAPAYAAQVER;GFLWEVPR;GYNYGGIFR;KGPLLLGSVK;LGPVGGVFNLAAVLR;SEAAVAVLLQR;VDASLAAGPAPAPYAAAVK;VLIDPAAQLAAVAAAGQPAQLDVR;WDHSLEWSVAHFGNASR;WVSSSLPR;YAGATELPAR | 0.0697 | 46.3930 | 13 |
| ATP synthase subunit O, mitochondrial | ANETLQLTAK;GEVTCEVVTAKPLDQTQR;KYTELISTAV;LDKLEAVINAFK;LEAVINAFK;LKEFIVNPTLK;LSPTTSNLLGLMAENGR;QNLEAALK;QNLEAALKK;TLDAVEK;TLDAVEKELSQFQQSIK | 0.4976 | 40.3764 | 13 |
| Calreticulin | FYALSR;FYNDPEEDKGLQTSEDAR;GLQTSEDAR;GPWVHPEIDNPEYTPDANLYK;HEQDIDCGGGYLK;KPEDWDDRPTIPDPEDK;KVHVIFSYK;QIDNPAYK;SGTIFDNFLIGDSIEEAAER;VESGDLEADWDFLPPK | 0.3208 | 39.6267 | 13 |
| UDP-glucuronosyltransferase 40Q2 | GFALEVPLAYDTPAK;GITLPNLYDLK;HLLEAGHEVTYITAHPYK;ILVAFAYPGK;KGFALEVPLAYDTPAK;LPTNYVGIGGYHIQDEIPPLPK;QILITSVIDKFEFAQSLDVTK;SETIPVELK;SHSILGEGYVR;TQLLNVFSK | 0.2428 | 44.3991 | 13 |
| SFRICE_000735 | ATITDVTK;DHDVDLYIK;GVNAVLLER;GYHLWNSDLR;KDGDYIGNEHVTR;KYAFFTLDNK;NAAALFNMSYYGK;NYNIVFPHDEHLAGR;VAVFGQEAIFR;VQQYDWGGAHSDYPR;YAFFTLDNK;YTDAGLSNDAFPVNTHR | 0.1421 | 51.1462 | 12 |
| Adipocyte plasma membrane-associated protein (Fragment) | GEVYTGLATGEIVK;LFHYDAAK;LFNSVALAK;LLGPEAFQLYK;LMAAAPLFR;LTPQGHVTFVTK;NGDLYWTDSSSDFELK;VETKPVQQPPVK;VGPLATNGALNNGGK;YYLSGPK | 0.1578 | 44.1409 | 12 |
| V-type proton ATPase subunit | AVAEYYAEYQALFEGAGTNVGDK;EKLVIEFQHLR;LHLQGTDYGTFLANEPSPLSVSTIDDK;LHLQGTDYGTFLANEPSPLSVSTIDDKLR;LNPDGLAALAR;LVIEFQHLR;NIVWISECVAQK;QADYLNLVQCETLEDLK | 0.2960 | 33.0264 | 12 |
| Heat shock 70 kDa protein cognate 5 | ALQDAEVQR;EQQIVIQSSGGLSK;ERVEVSNQAEGVLHDTETK;KDSADPEEIR;LESLVGDLIK;LFEQAYK;LFEQAYKK;SQVFSTAADGQTQVEIK;TATSTLQQASLK;VEVSNQAEGVLHDTETK;VQSTVQEIFGR | 0.1603 | 39.9980 | 11 |
| SFRICE_000291 | AAQLGSAHEVQR;ALDEFASK;DLISWVSDIR;DLSTVQTLLTK;ILETAEDIQER;KHEALEAELAAR;LFGAHEIQR;LWELLLSR;TYIAAVK;VSTLDGEAAR | 0.0401 | 36.7740 | 11 |
| SFRICE_003397 | EKPLVLYVFSK;FDYIFFTGGTSIGK;FVELTVLTNVVGSDK;IAIGGSYDANDKFVELTVLTNVVGSDK;IVYEAAVK;LQALVDASKDK;MLNLLLR;NLTPVTLELGGK;QQNVAEAVER;SIIHNLEDWAKPEKPEK | 0.2071 | 41.1537 | 11 |
| SFRICE_000152 (Fragment) | GPDLAPHVAPELR;IPIENVLGGIGNGFK;LAEAWVEEAVTR;LASSAIDAYTAAAVMSR;LFVALTGIQFAGSHLQELQR;NPTAHLGLIFSEAGR;SFGGVSSGPPENK;TVDKVTAFIVER;VTAFIVER | 0.2175 | 38.2944 | 11 |
| Isocitrate dehydrogenase [NADP] | AQDLVIPKPGK;DATDDQVTIDAAHAILK;IIWSMIK;LDCLYYDLGLPHR;LDDTPELER;LIDDMVAQAIK;LIFPYVK;VVPGWTQAMVIGR | 0.2028 | 33.2312 | 11 |
| P-type Ca(2+) transporter (Fragment) | ADEGLISGWLFFR;EFDDLPVSEQR;EIVPGDVVEVSVGDKIPADIR;FSVPVVLLDEVLK;GVVIGTGLNTAIGK;IGVFTEDEDTTGK;LDEFGEQLSK;MFIFEK;TEMSETEEIK;TGTLTTNQMSVSR | 0.1211 | 42.3040 | 10 |
| SFRICE_002792 | AGALLLVLPK;EPEFTLYGPTR;HWYTYLSSQPR;INLADELLAWQHER;KINLADELLAWQHER;NLADNIALTAR;SASFNLEGR;SLTSWSTSR;WSPDFEDILR | 0.1564 | 37.3206 | 10 |
| SFRICE_032354 | FQVQGYPTIK;KTYDSAEDYNGGR;NDYLAILAR;NLEPHWAK;SADAFVDAALSAVK;SSDSDVITLTDDNFK;SSDSDVITLTDDNFKK;TYDSAEDYNGGR;VGALDATVHQEMAGR | 0.2640 | 39.2557 | 10 |
| Elongation factor Tu | AHDNVEAAVYILSK;EHLLLAK;ELDKPFLLPVESVHSIPGR;INNNLSEEDR;LGDLTLGTGVITK;QIGIQHVVVFINK;SPEIGSEAISQLLK;TLDEAQAGDQLGALVR;VLADVNLAQK | 0.2495 | 37.1485 | 10 |
| SFRICE_020830 (Fragment) | AMTTEFLTEIETQYK;EVDWADQR;FFLMNLFGIPK;LNFDQYTER;LNFDQYTEREEPK;QVYYELR;VVVVEGPVAVGK;YSQYIDALTHLLNTGQGVVLER | 0.1356 | 32.7670 | 10 |
| Fructose-bisphosphate aldolase (Fragment) | ALQASVLR;AWAGKPENVLAGQQELIK;FTPSYQAIMENANVLAR;IVPIVEPEVLPDGEHDLDR;RPWALTFSYGR;TVPAAVPGVTFLSGGQSEEEASVNLNAINTVDLK;VTEVVLAAVYK;YASICQSQR | 0.5359 | 33.7533 | 10 |
| SFRICE_006684 | DGDHYVISGSK;DTPGLTVAKPETK;KDGDHYVISGSK;KLATEQIAPLVK;LATEQIAPLVK;VDPAVAAYVDIHNTLVNSLFMK;VHESSILGEYGK;YAAGFLNEGR | 0.1938 | 34.7918 | 10 |
| SFRICE_007086 | AEEEFNIEK;HMMAFIEQEANEK;IQSSNMLNQAR;KIQSSNMLNQAR;LELIAQQLLPEIR;NLLDEAR | 0.2389 | 25.9648 | 10 |
| Peptidyl-prolyl cis-trans isomerase | HTGPGVLSMANAGPNTNGSQFFITTVK;HVVFGTVVEGMDVVK;QVESYGSQSGK;TSWLDGR;VIPNFMLQGGDFTNHNGTGGK;VYFDVSADGSALGR | 0.5758 | 27.2643 | 10 |
| 2-phospho-D-glycerate hydro-lyase | AAVPSGASTGVHEALELR;DALFLIQDAIQQAGYTGK;FGLDSTAVGDEGGFAPNIQNNK;IATAVEK;LAEVYLDFIK;MGSEVYHYLK;SNPADYLSSEK;TPIQIVGDDLTVTNPK;YNQILR | 0.2725 | 36.2871 | 9 |
| SFRICE_014997 | AMLWDLNDGK;DKTLIVWK;DVLSVAFSVDNR;FSPNHANPIIVSAGWDR;INHLGHSGYLNTVTVSPDGSLCASGGK;IWDLESK;LWDLAAGK;VWQVSVSAR;YPDMILSSSR | 0.3386 | 30.8863 | 9 |
| V-type proton ATPase subunit C (Fragment) | EFAYNEADLAAGK;FPIPDLK;GNLQNLEK;KVVEEFK;NIADIISK;QVGQIDADLK;VAQYLGEVLEDQR;VGTLDQLVGLSDDLGKLDTFVEGVTR;YGLPVNFQAVVMVPSR | 0.2432 | 31.3042 | 9 |
| Glyceraldehyde-3-phosphate dehydrogenase | AGAEYIVESTGVFTTTEK;IAVFCER;IGINGFGR;LGKPASYDAIK;VIHDNFEIVEGLMTTVHATTATQK;VIPALNGK;VKEAAQGPLK;VPVANVSVVDLTVR | 0.3012 | 29.8947 | 9 |
| Adenosylhomocysteinase | ESLLDGIK;ESLLDGIKR;GISEETTTGVHNLYK;HNVYPVGVHTLPK;KFPQYLEGIK;KLDEEVAALHLDHLGVK;KVNIKPQVDR;VNIKPQVDR | 0.1721 | 29.0447 | 9 |
| SFRICE_003762 | AMEIVLTGNFIDATEAER;EGMTAFVEK;EMQNNTFSHNTK;FGQPEINIGTIPGAGGTQR;IAAIIITGNEK;KNVGLIQLNRPK;NVGLIQLNRPK;SGLQFEK | 0.3024 | 29.7004 | 9 |
| SFRICE_002276 | DHDFIVNLDQR;GKHIIEQLGGK;HIIEQLGGK;LEWSPVHK;MGNPWQPFLNLLNR;NLIEKPEDHQVAK;QDEFVQHMTAR;TLVHLLER | 0.1394 | 32.4509 | 9 |
| SFRICE_014874 | AKQDLLAALK;ALIAAQYSGADVK;FDPENYSIWYAEYK;KLVQDYFSWTGVDK;LDPASPDTK;LVQDYFSWTGVDK;TFLVTER;VLLSESNAIAYYVSNAALR | 0.1950 | 28.6581 | 9 |
| ATP synthase subunit gamma | AARPYGEGAVQFYER;ELIEIISGAAALD;GLCGAVHTGVSK;HIISVCNEIGR;IASAILESGYEFASGK;LSEPGAENIK;LTLTFNR;QLYIAMTSDR | 0.3186 | 33.3246 | 9 |
| SFRICE_004571 | AALNAFLYR;FSLLEAVK;IGKPTLVNETSR;KGVLLFIDEADAFLR;KPSDALAGVVLAPTLER;LYFDTFVLQPASEGKR;VFDWANTSK;YEEQLVQQQK | 0.1534 | 35.0528 | 9 |
| Mitochondrial heat shock protein 75 kDa | EYSIFLK;GVVDSEDIPLNLSR;IEVYTK;IIVHLK;NLIVHINELLVK;SFIEEIKK;WSSDGSGVYEIQEADGVAIGTK;YISQSYDKPR | 0.1199 | 26.2268 | 9 |
| SFRICE_018970 (Fragment) | FGLYEVFK;IQTMPGFASTLR;LGWAGVWK;MVQNEGYGTFYK;TATVGGIMGK;VSVAEEGMR;VTYSGLLDEETAYTYR | 0.2560 | 29.9884 | 9 |
| Glycine cleavage system P protein | AHLAPFLPSHPVVDPLADLGDAAHSFGSVSAAPFGSSAILPISWAYIK;EQAAFPAPFVK;GLVAHEFIIDVR;LGVPLGYGGPHAGFFAAEHQLVR;LHPQTLAVVLTR;VHNATLVLDHGIR;VSFQPNSGAQGEYAGLR | 0.1635 | 30.7669 | 9 |
| 97 kDa heat shock protein | HASTTLNQDEAVSR;HDLNSYVEQEGK;LRPEDIHSVEIVGGSTR;NALEEYVYELR;NALLDAANIAGLNVLR;NALNWLESAR;YAHLADVDVQK | 0.1094 | 30.0429 | 9 |
| NADH dehydrogenase [ubiquinone] iron-sulfur protein 3, mitochondrial | DFPLSGYVEVR;FEIIYNLLSLR;ILTDYGFEGHPFR;RFELSAPWEQFPNFR;TYTDELTPVDSANDVFK;VVVEPLELAQEFR | 0.3019 | 24.6623 | 9 |
| SFRICE_030091 | AALQALLR;AALQLLPALR;AAPPDVLLQHAER;AELQALQVAAAGLLR;LLGLAGTR;LPDVQLFR;LQALHAEELLAVGHAQLGLK;WAGALADDAAGAGGGAEGAR | 0.1285 | 31.1923 | 8 |
| SFRICE_001400 (Fragment) | AYQALTDDEAR;ELFGDTTIK;ITDNEEVPMLLR;LSPMIVQGLWEYK;QIIIEQSQPYK;QSLILHPDKETGDEK;SLLQLAQLPGEER;SLLQLAQLPGEERR | 0.1296 | 30.9465 | 8 |
| SFRICE_012231 | AGMYWLR;EMQDVESNLQEVVFDHLHATAFQGTPLGQTILGPTR;LCNTVTEGEVER;LSVLDNGIR;NNGVAHFLEHMAFK;RIPIHELDAR;SQGGGVNNASYLAR;VASEDSGAATATVGLWIDAGSR | 0.2661 | 29.5114 | 8 |
| Alpha-1,4 glucan phosphorylase | EIWGVEPSWEK;FLHLADYDAYIEAQEK;IEHGEQQEEPDDWLR;IGEDWTVHLEK;LIFLENYR;QLLNILHVITLYNR;SGFFSPGEPGR;VLYPNDNFFEGK | 0.1165 | 33.2282 | 8 |
| 5-aminoimidazole-4-carboxamide ribonucleotide formyltransferase | EALGLPAATSFK;EVSDGIVAPGYTPEALEILK;ITADLFK;LALLSVSDK;NAGLAVTEVSDITK;TIFGLTLEQR;TLHPAVHAGILSR;VALASDAFFPFR | 0.1633 | 31.6768 | 8 |
| SFRICE_010867 | ALEDAVEAEK;ALEDAVEAEKK;CIADLAALAAR;ENVLLQLEAAYR;KENVLLQLEAAYR;LDYQLEK;LMNAYTEVK;LMNAYTEVKR | 0.2131 | 34.5869 | 8 |
| Casein kinase II subunit alpha | FNDILGR;FVHSENQHLVSPEALDFLDR;GGTNIITLQAVVK;LIDWGLAEFYHPGQDYNVR;QLYQTLSDYDIR;TPALIFEHVNNTDFK;VLGTEELFEYLDK | 0.2805 | 26.7186 | 8 |
| Elongation factor 1-alpha | CLIEALDAILPPARPTDKPLR;IGGIGTVPVGR;IGYNPAAVAFVPISGWHGDNMLEASTK;KIGYNPAAVAFVPISGWHGDNMLEASTK;LPLQDVYK;QTVAVGVIK;VETGILKPGTIVVFAPANITTEVK;YYVTIIDAPGHR | 0.2441 | 29.6044 | 8 |
| SFRICE_001276 | ENPGTLDEIHK;ENPGTLDEIHKK;GSDYTLAMAVGKPDFNEK;LALGTELVYQSGAR;LTATQGTLEYK;QISPSEAFPVILGDVDPSGNVNFSLIHQLTPEIR;SAVFVGHYLQSVTK | 0.3270 | 31.8083 | 8 |
| V-type proton ATPase subunit a | DLNPDVNAFQR;EMIDLEATFEKLENELR;EVNQNAEALK;FTSAFQNLIYAYGVATYR;LHWVEFQSK;NYLELTELK;QAEIDTPLEDPSSSDQVYK;SVFIIFFQGDQLK | 0.1263 | 28.2614 | 8 |
| SFRICE_003046 | AFSPATVNTGR;DNSMIHELNR;GQYSSYPIK;GTEFEGAVIALFHLLATRPDK;IIEVGDTPK;IIEVGDTPKDR | 0.1303 | 24.1401 | 8 |
| SFRICE_014898 (Fragment) | AYEGENMLYEK;CDILVPAAIEQVIHK;DIVHSGLDYTMER;ESNYHLLESVQESLER;FNLGLDLR;IIAEAANGPTTPAADK | 0.3726 | 23.9495 | 8 |
| Dolichyl-diphosphooligosaccharide--protein glycotransferase | DFNLDVLEEAYTTEHWLVR;FESVIHEFDPYFNYR;FGLVYTEGGRPPGFDR;FYSLLDPSYAK;HENNLVFR;YLTEEGFYNFHNWFDDR | 0.1216 | 25.1907 | 8 |
| SFRICE_023417 (Fragment) | HTLAETNIETGETK;KHTLAETNIETGETK;LPFEQHWR;RDELDYSKPLDGQTK | 0.2099 | 16.2243 | 8 |
| H(+)-transporting two-sector ATPase (Fragment) | AIAELGIYPAVDPLDSTSR;FLSQPFQVAEVFTGHAGK;LVPLEETIK;SLQDIIAILGMDELSEEDKLTVAR | 0.4046 | 14.8790 | 8 |
| SFRICE_011143 | DYGVLNEETGIPFR;GLFVIDDKQNLR;HGEVCPANWQPGSK;IGCEVIGASTDSHFTHLAWINTPR;LVQAFQYTDK;QITVNDLPVGR;TTAVVNGEFK;TTAVVNGEFKDIALSDYK | 0.2918 | 28.0590 | 7 |
| SFRICE_003104 | DYVADKTPVVVATIDQYAPGLVDNVQSYASTGFQTIK;EFAYLALK;FSNAPLVWLK;SHVSSYFHWFR;TPVVVATIDQYAPGLVDNVQSYASTGFQTIK;VGVLYSNTK;YKDIYFNK | 0.1252 | 30.2477 | 7 |
| Peptidyl-prolyl cis-trans isomerase | FEDENFKLR;ILEGMDVVR;MGDENIGDIVIGLFGK;TEVVSEPFSVTK;TPWLDGR;TVENFVQLAQKPEGQGYK;VIDNFMIQGGDFTK | 0.4146 | 27.1911 | 7 |
| Annexin | ADAEALASAGEGQWGTDESVFNSILITR;DENQGVDEGAAK;LEIAETFK;LYYSMK;QIFAEYEALTGK;TISAFYEQLYNK;VGFFAER | 0.2632 | 25.8526 | 7 |
| Catalase | AVDNIVGNLK;FYTDDGNWDLVGNNTPIFFIR;GAGAFGYFEVTHDITK;IAAGLAPFKK;LFSYSDTHR;NLKDSPGYITTK;VSVSNYQR | 0.1696 | 26.7467 | 7 |
| SFRICE_002919 (Fragment) | ADFAQAILDAGLR;AGNVVHLYER;ENDVDAVHPGYGLLSER;FIGPSPFVVQQMGDK;HIEVQLLGDK;VPIVPGTDGPVTTK | 0.2438 | 27.2643 | 7 |
| ATP synthase subunit d, mitochondrial | ANLAAFK;INWAAYK;NAVPIPGMVDTFQK;SIEAFIQESNANIAK;VPYPADTQTAQVEAQWAEVK;VPYPADTQTAQVEAQWAEVKK | 0.3678 | 24.8145 | 7 |
| Retinol dehydrogenase 14-like protein | GNINFDDLNIR;GNINFDDLNIRK;IDVLINNAGVVVPLNQDLK;IVVVSSTLHER;TFLITGANSGIGYETAK;YNFDKDVETK | 0.2060 | 27.2643 | 7 |
| SFRICE_013982 (Fragment) | ALLELQLEAEELYQTFQR;EEIDGLLTK;GSVGFGSGLHGWAFTLK;KEEIDGLLTK;LWGENFFNAK | 0.1054 | 21.1596 | 7 |
| SFRICE_009010 (Fragment) | KLQQIGASVTASGDR;LQQIGASVTASGDR;QLVSGQEFRPWELTDNEPR;TFVAALDNGSPVTR;YEPQTELGVAHMLR | 0.3875 | 22.7202 | 7 |
| SFRICE_012732 (Fragment) | DVDTVITAYR;GDYIPGIWVDGMDVLATR;LYEQLAILR;NFYGGNGIVGAQVPLGAGIGFAHK;RIETASGNLYR | 0.1915 | 22.7202 | 7 |
| SFRICE_002106 | EDTGALPDETSFNLVNEASVDDLNTR;GFPELLAVQPTIR;LLQEKPVK;LNVNLAEIVALQKPK;LPEKWEQVGVLK | 0.2216 | 20.2417 | 7 |
| Ubiquinol-cytochrome C reductase | HRTENEISTEQAVPVDTLR;QSFTYLIAGAGGVAGAYAAK;SIVTHFVSSMAAAADVLALAK;TENEISTEQAVPVDTLR;TENEISTEQAVPVDTLRDPQHDNQR | 0.2509 | 22.7202 | 7 |
| Actin (Fragment) | AGFAGDDAPR;AVFPSIVGRPR;DLTDYLMK;GYSFTTTAER;IWHHTFYNELR;TTGIVLDSGDGVSHTVPIYEGYALPHAILR;VAPEEHPVLLTEAPLNPK | 0.2606 | 18.1762 | 7 |
| 40S ribosomal protein S4 | AYISLPR;DSTGHTFATR;ERHPGSFDIVHIK;HPGSFDIVHIK;NVPFLVTHDGR;VNDSVQLDIATSK | 0.2053 | 20.8141 | 6 |
| AP-2 complex subunit alpha | ALLLSTYIK;ESSILAVLK;IGLFYGNK;LLQNYTPPSEEPGVR;LVNLFPEIK;VGGYILGEFGNLIAGDTR | 0.0733 | 22.8550 | 6 |
| SFRICE_000170 | EEFPTATIFR;LVNLISPSYPLGGLHWEAIEK;MEDQVPWELKPFR;SDFYDAQR;VAGDLGQVLFTPYDLR;YSNVVINLVGR | 0.1975 | 22.1848 | 6 |
| SFRICE_002973 | AEGPFAFYK;FFVMESLR;FGVFEQAR;LYMLTEDGTLSTTGK;SHGVFGLYR;TIVREDGVGGLYK | 0.1962 | 24.6652 | 6 |
| SFRICE_035016 | DGYKPGIK;FNMGEYVWR;HFGAGLR;QNIVMFAETR;TVLSEEDEPQPNGR;VDTIIYMEDQLK | 0.1685 | 25.1907 | 6 |
| Glycerol-3-phosphate dehydrogenase | LVQDFGLEMEVAQHLAK;MNLAIALTAAR;NYLNPDVEVR;SSYYLSK;TAVDMIAR;VIFFLPWLK | 0.0822 | 21.9864 | 6 |
| SFRICE_000208 | DKVQAIIDAVSEIPQR;ILEDVFPIYR;LGIPYNPAYVPFHFLEGGTKPTLYQR;LYTNNWLPQVDILAHPK;VQAIIDAVSEIPQR;VQEVLNVMK | 0.0492 | 24.7582 | 6 |
| Aminomethyltransferase | AGTELLVNIR;GGIIDDLIVTK;RTDDNFPGSSIILR;SASIFDVSHMLQTNVR;VNAETLYVVSNAGR;VVDFAGFLLPVQYSDLSLSESHLFTR | 0.2209 | 27.2643 | 6 |
| Cation-transporting ATPase | ALEEAPR;FLLELMPHIK;GYLDDEAIDIAEK;NHFSSALK;NHFSSALKR;VLALGYR | 0.0397 | 18.3335 | 6 |
| Electron transfer flavoprotein subunit alpha | AAVDAGFVANDLQIGQTGK;GLTPETLTPLILATQK;KIGGDVAVLVAGTK;LDVSPITDIIGVK;LLYDLADKLNAAVGASR;TIYAGNAILTLEAK | 0.2818 | 26.2228 | 6 |
| SFRICE_021217 (Fragment) | DLALYALGIGASVLNEGDLK;KKPSDPVTIESVK;MAEVTVDLVER;TAEDQAALYR;TWTLDLK;VPEVYEGQPK | 0.1266 | 21.9858 | 6 |
| SFRICE_033731 | DGDYVLVLK;GVAHDEYIPYSIK;INALYVMR;LYYNEEESDIR;VNTKDEILYQTER;YHHSTFGYDIPANR | 0.2566 | 24.9234 | 6 |
| Multidrug resistance-associated protein 4-like protein | ILVTHQLHHLK;QNILFGLPYDR;SPLFAELLQEEEQPEDPK;SPLFAELLQEEEQPEDPKVQYLR;SSLISALFR;SVTSLSSAVQSGGSNFSAGQR | 0.0551 | 21.1464 | 6 |
| Translationally-controlled tumor protein homolog | DIITGDEMFSDTYK;IEGFNPSAEEADEGTDTAVESGVDIVLNHR;LVDEVIYEVTGK;LVETYAFGDK;LVETYAFGDKK;SYTLYLK | 0.4302 | 24.6623 | 6 |
| 60S ribosomal protein L18 | EGMIAVVVGTITNDVR;FNTIILR;ILAAGGEILTFDQLALR;INRPPLSLSR;MSVAALHVTEK | 0.3333 | 21.0795 | 6 |
| Vesicle-fusing ATPase | EIDIGIPDATGR;ETVVEVPNVTWGDIGGLQNVK;GILMYGPPGTGK;LAGESESNLRK;LDQLIYIPLPDEK | 0.0856 | 20.4819 | 6 |
| NADH dehydrogenase [ubiquinone] flavoprotein 1, mitochondrial | GEFYNEASNLQVAIAEAYQAGLIGK;GGAWFASFGR;IFTNLYGR;LIMFYK;LVEGCLIAGR | 0.1224 | 16.0674 | 6 |
| SFRICE_014294 (Fragment) | LTKPGAALVYWVEHVVR;NHPYQIQEFAQEAVR;QTVIWK;SPALHVPLYQR;TFYTQPEER;VEELWSR | 0.0455 | 16.8668 | 6 |
| SFRICE_002773 | HVEDLGPLTK;IIAGDKAPLLK;ISQETTTTENAPEPYYHR;NEVAEEDSEAVKR;RNEVAEEDSEAVK | 0.1013 | 20.7339 | 6 |
| Calcium-transporting ATPase | AGQILWIR;EASDIILTDDNFSSIVK;ERHPEESFTR;LSAHSAAGLR;YGDLLPADGILLQSNDLK | 0.0533 | 20.1183 | 6 |
| AIR carboxylase (Fragment) | ATILVFEILEK;KATILVFEILEK;LATGSFLK;NLTTVTAADLDTVKR;SAGINTAFVK | 0.0549 | 21.6788 | 6 |
| SFRICE_026018 (Fragment) | GSDLAADHTYYSYWR;TGHTGYVALFNPTEDDQR;VIVDLIPNYVPLTHMWFR;VVDDASEAPGTGSSVFTVNEEGGR;WPALNLR | 0.1416 | 20.5009 | 6 |
| Prohibitin | AAQFGLILDDISITHLTFGK;AAVIAAEGDAQAAVLLAK;AVVAQFDAGELITQR;EFTQAVELK;NLVVGEGTHFFVPWVQRPIIFDIR | 0.3139 | 20.6467 | 6 |
| Very-long-chain enoyl-CoA reductase | DLGPQISWK;IVVNESTTIK;LLETLFVHR;RAILPFIL;SLKDEETLK | 0.1500 | 22.7202 | 6 |
| SFRICE_001599 | DFVNLYLHK;ITTGAQDDLKK;LHPGAALDGK;LINPTDIGVK;VHLSPLK | 0.0606 | 17.3039 | 6 |
| Moesin/ezrin/radixin homolog 1 | APDFVFFAPR;EEWEQSITNWWQEHR;IGFPWSEIR;LFYLQVK;LQLEIAAR | 0.0852 | 18.9557 | 6 |
| SFRICE_032051 (Fragment) | AHANIPDDENVK;KESTVGEIVNLMSVDAQR;TALTSAIYR;TSASVNFKPESEK | 0.1042 | 18.1762 | 6 |
| SFRICE_000786 | AADRPALPAALK;ALLANAATSALR;IDVALWAIR;LLISLVEFQSR | 0.1477 | 18.1762 | 6 |
| 60S ribosomal protein L18a | AHSIQIIK;FWYFLR;KLPSESEPKPPLYK;LNTFAYK;NFGIWLR | 0.2373 | 15.5632 | 5 |
| SFRICE_026609 (Fragment) | AYLPVNESFGFTADLR;DSVVAGFQWAAK;EGLPDLNQYLDKL;EGVMAEENLR;YLTDKYEYDVTEAR | 0.2720 | 20.7050 | 5 |
| Lon protease homolog, mitochondrial | DGPSAGVTIATALTSLALR;DYDDLPGFIR;FQQTEEVK;ILEFIAVSQLK;TGTENPLVLIDEVDK | 0.0654 | 19.3379 | 5 |
| SFRICE_002630 | DAVLLIFANK;HYFQNTQGLIFVVDSNDR;HYFQNTQGLIFVVDSNDRER;LGEIVTTIPTIGFNVETVEYK;LGLHSLR | 0.3187 | 20.3653 | 5 |
| Nucleoside diphosphate kinase | EIALWFGDK;GLVGTIIER;HYSDLSSKPFFPGLVK;NIIHGSDSVESAK;TFLMIKPDGVQR | 0.3856 | 19.5189 | 5 |
| C-1-tetrahydrofolate synthase, cytoplasmic | ASGDTPHCAVIVSTVR;DITETELLAK;KFSPIQLR;SGNPVTADDLGMTGALLVLLR;TDPDTLTEGEK | 0.0707 | 16.9049 | 5 |
| SFRICE_000701 | DTVASTAQSTVDSTK;ETVANTVHSTIDTTK;NVAAAAVEK;NVAASAVDQSTTFIGAAK;QAAASAVDTGVSYAASAK | 0.0731 | 21.4207 | 5 |
| Gamma-glutamyl kinase (Fragment) | ASAIHTLADLLVNKQEEILEANTK;GPVGVEGLLTTK;GVGADDAISLVSTR;HIDLIIPR;LASQLTFGPPPAR | 0.1170 | 21.6788 | 5 |
| CAAX prenyl protease | IVLFDTLLEK;IVLFDTLLEKFDEEK;LHFPLSQIYIVEGSK;LYSAWYHSHPTLLHR;SAHSNAYFSGLFGNKR | 0.1268 | 22.7202 | 5 |
| SFRICE005413.2 (Fragment) | AFHAGEYDK;ASLFTQLENTER;NEALLLR;STFYLLLGR;YKNEALLLR | 0.0712 | 16.9695 | 5 |
| SFRICE_015281 | ANATNYGLAAGIFTTNLNNALQFSK;EEIFGPVQSILK;FDTLDEAIDR;LIQQASGASNLK;LIQQASGASNLKR;VLGYIEK | 0.0696 | 17.9914 | 5 |
| Dihydrolipoyllysine-residue acetyltransferase | FVTFTASADHR;LGGQGSGLYGSLK;QTIPHYQLTATVNVEK;VPTVNSHWMDSFIR;VSMNDFIVK | 0.1263 | 21.4207 | 5 |
| Staphylococcal nuclease domain-containing protein | FFLESK;IFLASIRPPR;LLEAELK;LVELEDVAK;QDGESEPYAEEAR | 0.0503 | 15.9486 | 5 |
| ATP citrate synthase | EQQVGAAVGK;HFIVEPFVK;IYLLDLAAK;LLTTFILALYR;NWGEVTFPPPFGR | 0.1327 | 19.9134 | 5 |
| SFRICE_003274 | ASIPDATASIQAR;TSVEGLSK;VLSAPFEMLQVSSSGHAR;VTLLDAIGR;VVPLKPGVITIDVR | 0.0361 | 19.8601 | 5 |
| Probable methylmalonate-semialdehyde dehydrogenase [acylating], mitochondrial (Fragment) | AVSFVGGDAAGK;EHTLNQLAGAAFGAAGQR;EWIPDLVAR;TNNWIELTNPATNEVIGR;VPEATKDELNAALESAK | 0.2050 | 19.3000 | 5 |
| SFRICE_014941 (Fragment) | AELHTLQAR;AQAVAELQNEVQK;DAAALAQQHAADLAQQLQDANR;DWLQAFAR;VESIVESLRDELSAAQR | 0.1280 | 17.7823 | 5 |
| SFRICE_037205 | AAADAVQIFGGNGFNTEYPVEK;AAWMADHGVR;GITFEDVR;IAMGAFDK;KTFGVPIAR | 0.3098 | 13.8689 | 5 |
| SFRICE_032134 | GWESGADVQNTKPNK;KYDLASWGVAGR;NTNLINK;TGEDISSVK;VTGTNPGYGATVVALLMAAHTILK | 0.1675 | 19.4749 | 5 |
| SFRICE_027928 | ANIGVVPQDTVLFNNSVR;ILTFPDAYDTQVGER;LVLSNISFK;TILKDPAIVLLDEATSALDTNTER;TTIIIAHR | 0.1373 | 21.6788 | 5 |
| SFRICE_036126 | DNYVFEFIPENR;DTLKDVVAPEQALVAWGGK;DVDGSLLFIFK;DVVAPEQALVAWGGK;EGLFFTHGR | 0.1118 | 22.7202 | 5 |
| Ribosomal protein L7 | EDKINELLR;IAEPYIAWGYPNLK;LNNPTGGWR;TIHYVDGGDFGNR;YASNFLWPFK | 0.2099 | 18.2842 | 5 |
| SFRICE_005246 | ANSEEQIVELNAK;ESLASTSQNATEALTR;IQAFEEELISVSR;YVSAESALEEFKK | 0.0424 | 16.6156 | 5 |
| Transaldolase | ILDWYVEHTKK;LLQELAASEQPLKR;TYEGKDDPGVLSVTR;VSVEVDAR | 0.1446 | 14.9248 | 5 |
| ATP synthase subunit beta | FTQAGSEVSALLGR;IGLFGGAGVGK;IPSAVGYQPTLATDMGTMQER;TVLIMELINNVAK | 0.0791 | 18.1762 | 5 |
| SFRICE_016401 (Fragment) | HQEATVLVNFASLR;LYRPGSVAYVSR;WPAWPVTR;YALDVEAITTR | 0.0719 | 15.8353 | 5 |
| SFRICE_022336 | AYALLLASR;IDILINNAGILR;MSDQDWDLIHLVHLK;TTHAAWEHFR | 0.2222 | 18.1762 | 5 |
| SFRICE_006893 | ANMVAQHLNLSSSEVSAEASK;AVANILK;TPAEIAK;WGSDNSPLAK | 0.1636 | 14.6638 | 5 |
| SFRICE_034158 | AEIALLTK;ELTQALLDELAEDKPVR;RELTQALLDELAEDKPVR;SEDVLAASFASR | 0.1343 | 16.6156 | 5 |
| Isocitrate dehydrogenase [NADP] | DIFQEIYER;IIWDLIK;LVTGWEKPIIIGR;TVEAEAAHGTVTR | 0.0968 | 15.1195 | 5 |
| Eukaryotic translation initiation factor 3 subunit E | FDLTFK;HLVFPLLEFLAAK;SEALTSLVER;YLATAVIINR | 0.0876 | 15.0612 | 5 |
| SFRICE_001395 | AEQFYR;IEPPNVGK;LGVESIIMGMPHR;LSGEDVER | 0.0445 | 14.8502 | 4 |
| Pyruvate kinase | GVNLPGLPVDLPAVSEK;LTTDAAYQER;NGAALQEIR;TGDSVVVVTGWK | 0.0896 | 15.8353 | 4 |
| Glucose-6-phosphate isomerase | AVLHIALR;INIQQLFK;QDPAFVEIQK;TWFLEAAK | 0.0582 | 10.7940 | 4 |
| SFRICE_002878 | AAFDDAIAELDTLSEESYK;DSTLIMQLLR;HLIPSSQTGESK;IISSIEQKEETK;YLAEFATGNDRK | 0.2481 | 18.1762 | 4 |
| SFRICE_009741 | EALSTLGADIAYIDFK;EDIKEALSTLGADIAYIDFK;ESTLDDLLK;VLEGEEETTYLQK | 0.1105 | 15.8625 | 4 |
| Triosephosphate isomerase | DIGANWVILGHSER;QVTEIVETLKK;TATPQQAQDVHASLR;VVLAYEPVWAIGTGK | 0.2218 | 16.6156 | 4 |
| Mannosyltransferase | ILQAVISTAADYSFYK;LGHYEQESPK;LIVTPWEFFR;QAPDLLVLYDR | 0.0931 | 17.1348 | 4 |
| SFRICE_035634 (Fragment) | HGVPLLYSGESDENEIYGFFEK;LHAVWESVGATLK;LYNPAKDPALVFFR;SFVIFAQDWYK | 0.2335 | 15.6701 | 4 |
| SFRICE_027297 | EFYSNLDDSDLIWK;SSEDAVDVNAQIDEQR;THNLLTGQTEK;WQLYTAQAMR | 0.0715 | 14.2234 | 4 |
| SFRICE_004313 | EEIFGPVQSILK;FETFEEVVDR;QAEGEVLWSAGIIR;TVGNPYTDVQQGPQIDNDMFTK;TVSFSLPK | 0.1229 | 15.6524 | 4 |
| Succinate dehydrogenase [ubiquinone] iron-sulfur subunit, mitochondrial | ETQLLQDVKDR;QYQSIEPWLQR;TFAVYR;WIIDSR | 0.1206 | 14.2797 | 4 |
| SFRICE_011091 | APSNIYEIR;ENADYAALEK;TSVEFVHSQK;YVGGFEK | 0.1314 | 15.2359 | 4 |
| SFRICE_008127 | IPVYEGTR;LLDYFLGEEIGTHYNR;QDVIQHIK;VTDHVNDLDKEEVNIISGALDLR | 0.0764 | 14.2068 | 4 |
| Aspartate aminotransferase (Fragment) | AFGVQTLSGTGSLR;AIDFDGLIEDLR;NFGLYNER;VGNLAIVVSDASVVQALK | 0.1667 | 16.4108 | 4 |
| Formylglycinamide ribonucleotide amidotransferase | DLEPWFVVPLQK;ELVEPEGAVVYTK;GLLDPAAGAR;NLQGNQIVIEIGPR | 0.0366 | 15.1484 | 4 |
| SFRICE_013145 | ENVAFVPVDVTSEK;ENVAFVPVDVTSEKDVK;TIEVNLIGTFNVIR;VVTIAPGLFR | 0.1608 | 18.1762 | 4 |
| Aconitate hydratase | FLETAGAK;LSDPFADELPAR;NTIVTSYNR;SKVPFNVTPGSEQIR | 0.0759 | 12.6309 | 4 |
| ER membrane protein complex subunit 1 | HVFESASTGNIQLLQITEK;IIVLVTDVGK;VVTVSGSNPYLVR;WSGAGAAFTSLWAER | 0.0624 | 16.2243 | 4 |
| SFRICE_008303 | EAAFSLAEAK;FTTGDFNQVVLQNVTK;LAIFPSR;TLAYIISELDELEREEFYR | 0.2114 | 15.7075 | 4 |
| SFRICE_008775 | FEIWDTAGQER;GAQAAIVVYDITNQDTFGR;QASPSIVIALAGNK;TAMNVNDIFLAIANK | 0.2770 | 16.5354 | 4 |
| SFRICE_025754 (Fragment) | AIMQLGYDVAPDGVK;EPWTYANK;ISDEEPVYLFK;YGVFQTEASQADTQEFR | 0.1256 | 13.8618 | 4 |
| SFRICE_030420 | ASGLETHPELGGLLVNAELQAR;EAAPLGAVLPPYLAAEAAR;ELWREPDLAAR;TVTQLFR | 0.0913 | 16.1026 | 4 |
| SFRICE_023928 | IKDLDTLKPLEEFEYLK;LTHLNLSGNK;NLDLFNNEVTSIEEYR;VQDGEENADGAPEESTR | 0.1974 | 15.5742 | 4 |
| SFRICE_003080 | GSLVETLK;VDSNGLLYITGR;VGDLQPTYDR;VNSETSEALDDLDNETR | 0.0648 | 15.9723 | 4 |
| SFRICE_013096 | AEAALAYELQAAK;DVYDDVQYLASLGK;LPAEAEAYR;TLTGLDLSQVLK | 0.1472 | 18.1762 | 4 |
| V-type proton ATPase subunit a | DLNPDVNAFQR;LGFVAGVVNR;QNYLELTELR;TVFVAFFQGEQLK;VPAFER | 0.0818 | 15.8497 | 4 |
| Prohibitin | AAFVVER;IVQAEGEAEAAEMLGK;VLSRPDASSLPTMYR;VPWFQYPIIYDIR | 0.1753 | 16.8767 | 4 |
| 40S ribosomal protein SA | ADGTHVINLR;AVVAIENPADVFVISSRPFGQR;EQVVAVAPPK;SSHSIGLMWWLLAR | 0.1824 | 16.6156 | 4 |
| Succinate--CoA ligase [ADP-forming] subunit beta, mitochondrial | FDDNADYR;FHVATTADEVVK;MYDLFLK;NLNIQLPVIVR | 0.0841 | 11.3048 | 4 |
| FK506-binding protein | EVISGWDVGIAGMK;IIQVPLDLYFK;KQIAGGVSIEDLK;VVMVYYEGR | 0.1138 | 18.1762 | 4 |
| Alkaline phosphatase (Fragment) | LQSLAALALDANK;QFWLDLAHNELEEALK;VAVSETLAMEK;VTHATPSPLYAHSASR | 0.1120 | 14.5420 | 4 |
| SFRICE_001226 | AADVVLAAHLASEAALR;LAGQIVNR;SIIQNPSEAQR;VLEQVIAK | 0.1161 | 16.0934 | 4 |
| SFRICE_000822 | DVATFLR;LAYKPVSK;LSDYLPSPYPNENAAR;NLVNVTHTEEEAK | 0.1354 | 15.8625 | 4 |
| Adenylate kinase | EEVASGSELGR;IDNIFK;NGFLLDGFPR;TSEQVFNK | 0.1417 | 14.3157 | 4 |
| Mitochondrial import inner membrane translocase subunit TIM50 | ELTFYEK;EVLTYYSQFDDPIAAFR;GPDQPLTR;VIVIDWNK | 0.1044 | 14.7467 | 4 |
| Complex I-49kD (Fragment) | HPPYHGVVAPGEK;LLNIDIPLR;TLFAEITR;TYTQALPYFDR | 0.1362 | 16.4108 | 4 |
| UDP-glucuronosyltransferase | GIWWIEHVLR;IQTLINAFSK;IYNLTIYEK;TYLDSSK | 0.0695 | 14.9677 | 4 |
| SFRICE_024442 (Fragment) | AAVAAAALPGVR;LNVLIDR;LQLTAALR;TNVVQSLLAR | 0.1276 | 12.0551 | 4 |
| Tubulin alpha chain | AVFVDLEPTVVDEVR;EIVDLVLDR;LIGQIVSSITASLR;NLDIERPTYTNLNR | 0.1156 | 16.4108 | 4 |
| 6-phosphogluconate dehydrogenase, decarboxylating | AGSAVDEFVR;IIGATSLEDMVAK;LISYAQGFMLLR;SAFLGNIK | 0.0890 | 16.8767 | 4 |
| Cytochrome P450 CYP4C1-like protein transcript variant 1 | ILTPTFHFNILK;LDNDQTSATVDYK;LYPSVPFIGR;TTLSELVR | 0.0869 | 15.4940 | 4 |
| 60S acidic ribosomal protein P0 | AIKDHLETNPALEK;APARPGAIAPLSVVIPAHNTGLGPEK;GHSIVLMGK;LLPHIK | 0.1746 | 13.2486 | 4 |

Table S25 Identified *Spodoptera frugiperda* larval coded proteins associated to HvAV-3h virions (Sf-3)

| Protein ID | Identified peptide sequence | Sequence coverage | Protein Q score | Unique spectra |
| --- | --- | --- | --- | --- |
| SFRICE_003260 (Fragment) | AEAQPFNNELDLDLYVK;AGTGYSIEASIQLVK;AHMLFIDAELAYPTSTGLPLK;AIISLLQTEQK;APAYGADVDLYLNYEK;ASNTNLEKDIINYEGQVELK;AVYDKNGVEVFAK;DIINYEGQVELK;DINFDSVAEGNVQK;DKHNIPISGQVVEEIGGGK;DSAENFNVITK;DVTQSNDKYSGHAEIVLSDAAK;ESFELLFDAEGAK;FDADIDPK;FDIGAIETTTLYSSHAK;FEIYAVPLK;FLVNTPLVK;FTLQDGR;GFDLQVGLPIDK;GFDLQVGLPIDKQEIVTASNDLVYFYAEK;GHFEYEPVMR;GKYDANELYGK;GNGMIIVDFK;GSPTTQSFLNGHK;GSYGDDTNFELAGVLQAK;GVETFFNMAIGER;HLEDALIDK;HNIPISGQVVEEIGGGK;HQSPPGIDKPIR;IDMSKPGVITNVVSAGFDLR;IETSASISHPILGGDR;IKFDADIDPK;IVSTVLYVQPENK;KHQSPPGIDKPIR;KIDVDVEGQYQDK;KLEFSVHSK;KNDVNIEFQNR;KPSELTVVVK;KQTAVAEFGFNHPK;LAHCVSDNNVK;LDIIGAATGR;LEFHATNDNK;LGIAPEFGLMIKDEVSDQR;LLVPHVDPVVLDVNYK;LNPFINFR;LPQYTLDLHINKEDK;LVFFSLSNAR;MNLPSVK;NAIAANGQFK;NGVEVFAK;NKFEIYAVPLK;NKFEYSGQK;NLLDTEPVHQVGNFISTSLR;NLLNTFSLK;NPSPDTYISNVVVDADGR;NVIYSQDSFLPR;NVLSGSTSYISK;NYQVEIASK;QLDSEIR;QTAVAEFGFNHPK;RLEFHATNDNK;RVFLDGLIR;SAVISMDAEVAPR;SDSIIVFSK;SGITIQLPQR;SKELGPIEQK;TEEPQELYEK;TEMGALLAFNSPMK;THGMIEIVLPTER;TTNKDDLMNVYSHVK;VAATEEYLYRPFSVGENGAR;VAEHLFGPR;VEEDNVVAVLK;VFLDGLIR;VHGSVLHPQQNAIK;VMALESVVSYPTNK;VVDLEGTATVNENLQK;VVFGQMLK;YELSGVLLHK;YHMHVYNTPEFGNAKSGITIQLPQR;YLDDFTVNVR;YLSSYPLTGK;YNYGVEGTVTVFLTGASNQETSVK;YSGHAEIVLSDAAK;YSIENIK;YTSPSTPKPSR | 0.4216 | 352.8846 | 119 |
| SFRICE_015484 (Fragment) | ALIPTPEIKDFTDALHSYVTK;ALVLEDK;AVVVNGQHFFTFDGR;DFTDALHSYVTK;DLDNLEAR;DNFNYFYSALR;EAVHQACDLAR;EKYQELAIPEQALALLGDAYNTIR;EYLDSLIEVVAR;FPYPIIYDTDQR;GLLGDGNNEPYDDFR;GLLGDGNNEPYDDFRLPNGK;HKPELEELTNALTAIFK;ISTSESEFGNAYR;LLTGDAPDVLALIK;LSIVGHVQPEKR;LSTAITSLIQFIR;MLEEVLNSTKPLVDNILK;NFKDIVVPLVGQVLDGLK;NMVHGALFLDDSLNLK;NPAQIVGYTER;QADIVVAFETTK;QADIVVAFETTKDNEK;QFLSTAAAAINQR;SFNPLDEVPAK;VDVSADLILGGEK;VDVSADLILGGEKK;YFKEYLDSLIEVVAR;YVLAHDHVDR | 0.3448 | 113.8270 | 41 |
| Dolichyl-diphosphooligosaccharide--protein glycosyltransferase subunit 2 | ALHFDGGLPATATLLAAIHR;DATIADIYHAVFTLK;FAEYLLTR;FITSESDSIEFSISDLVGRPLR;GARPELAHTFR;GSVYDKEDALK;KDATIADIYHAVFTLK;KPSPLTEEQK;LADDVVVLSK;LGLNVAR;LLKPDEVLAQSGTR;LSMFDTAR;LTDKLQADHLQK;LTTVAYPNK;QPLTQKPNEPTTFVLNLSK;TTNVNVAVLGEIR;VELNVGGIAK;VSTVEVGVGDVDGTTSPK;YVAPLAALTFLAGHR | 0.3758 | 76.6286 | 28 |
| Heat shock 70 kDa protein cognate 4 (Fragment) | ARFEELNADLFR;DAGTISGLNVLR;FEELNADLFR;FELTGIPPAPR;HWPFEVVSDGGKPK;IINEPTAAAIAYGLDK;IINEPTAAAIAYGLDKK;ISDSDKQTILDK;KFEDATVQADMK;LLQDFFNGK;LLQDFFNGKELNK;MKETAEAYLGK;MVNHFVQEFK;NQVAMNPNNTIFDAK;NTTIPTK;QTQTFTTYSDNQPGVLIQVFEGER;SINPDEAVAYGAAVQAAILHGDK;SQIHDIVLVGGSTR;STAGDTHLGGEDFDNR;TFFPEEVSSMVLTK;TTPSYVAFTDTER;TVQNAVITVPAYFNDSQR;WLDSNQLADKEEYEHK | 0.4349 | 81.7524 | 26 |
| Protein disulfide-isomerase | DDDVIIAK;EEIPSAR;FDEDLFK;ILEFFGMK;KTGPPAVEVSSAEQAK;LGEHFEKDDDVIIAK;MDATANELEHTK;NGDFDKYTEELKPVAK;NGSPIDYTGGR;QHLLSEDLPADWAAKPVK;TFTSVASAVDDHVFALVSDPK;TGPPAVEVSSAEQAK;TLVATNFDEVVFDNSK;TLVATNFDEVVFDNSKK;VMSVAIDADEDEHQR;YDAEKFDEDLFK;YTEELKPVAK | 0.3401 | 65.1596 | 26 |
| H(+)-transporting two-sector ATPase | ALDDFYEK;ASLAETDKITLEVAK;DINELTQSIYIPK;EILQEEEDLSEIVQLVGK;GNEMSEVLR;GTVTYVAPSGNYK;LAEMPADSGYPAYLGAR;LASFYER;LLKDDFLQQNSYSSYDR;LPANHPLLTGQR;NIIAFYDMSR;NYPEFVPLR;TGKPLSVELGPGILGSIFDGIQRPLK;VGSHITGGDLYGIVHENTLVK;VGYHELVGEIIR;VKEILQEEEDLSEIVQLVGK;VTDVVLETEFDGEKEK;YTMLQVWPVR | 0.3815 | 69.6541 | 25 |
| SFRICE_011490 | APPVLEVK;ATHSYEEPVYYFVSDGQYK;DNKDIENVR;EGNTALPVGVLAVK;ESTTPELLGLK;FQPAQALADTSTLVPTTVYLVATAHAVVIR;FQPPHHIITLAQGQAHHITLR;GYDVSGSVTSFGSPIR;HNVAVTQDHTNVPTFEHTGYVLR;LLALPTAPGQVAISYNVKPESVPFTVR;LLESLAAAAAK;LTQVTSEDGK;NAFEVTGFTVVGSVVK;NTVTTVGGDFYFTPVIPGK;QVTDGSVVVYTSNPTHYR;SVGGPGLSGAR;VITAGQNQGPSGIAVQLINDK;VITAGQNQGPSGIAVQLINDKGDVR;VSVGNGPVSGVTFSQLK | 0.2594 | 82.4778 | 24 |
| Heat shock protein 83 | AADTLYR;ALLFVPR;EEAADYLQPDTIR;EIFDLEENEQQKER;ELISNGSDALDK;IPDSEYESFWK;KIPDSEYESFWK;KPNEVNDEEYTQFYK;KVLDMLK;LALSNAHQK;LLHIIDSGIGMTR;MKPNQNHIYYIAGSSR;NELINNLGTIAK;QFEPLTTWLGNK;QHVWESDASAFSVAEDPR;RGTHITLHVK;SGTAEFLSK;TETVEESDADADAEDK;VQEAPDSPDTAR | 0.2723 | 75.4179 | 24 |
| SFRICE_001118 | EADYATPLYTPQNR;EGFTALVR;FGTYAFR;GLCTGDKYPILR;LDSESEISGVPPIHADGAGEAGPYTK;LPDDNGDPGLWVSYEDPDTAGQR;MVSLNENLDVDR;NLGGVSIVDLSMDDFR;NPLLNADAVVNHWIQGGAPAHK;STWGSIWHGIK;TAFVNSGVLLAEQHGFDGIDLAWQFPK;TEGILSYPEVCAK;TFGTTPVDEKEAEHR;VLLSVGGDSDTEEAQKYNLLLESPQAR | 0.5369 | 55.1575 | 23 |
| SFRICE_024780 | AVVGPDGNVASNYGVIDPR;EIHLVIITTK;IHTNYGIIDPK;IQTDKELINTK;LVPASAATPDLEEK;LVVVTSK;SGHILFTSGVIDPK;TDSSLAQQLTVVDR;TGHLIVSSGVVDPK;TGKPDSSLAQQFTIVEK;TGNILITTAVVDPK;TGNILLLSGVVDPR;TGNLLITSGVVDPK;TGNLLLTSGVVDPQTGK;TGNLLLTSGVVDPQTGQVDPNLGQQISVVDKPK;TNQIWIAGPK;VEPSYAQQLSIVDK;VWVDLEK;YVNLVIVTSK | 0.0917 | 74.8095 | 22 |
| Mitochondrial 60 kDa heat shock protein-like protein | AAVEEGIVPGGGSALLR;AEQIRDQIQETTSEYEK;ATLSDMAIATGGVVFGDDANLIK;DDTLFLK;DKFQNIGAK;DQIQETTSEYEKEK;GANPIEIR;GVMLAVDAVK;GYISPYFINSSK;ISNVQTIIPALELANQQR;KISNVQTIIPALELANQQR;KPLVIVAEDVDGEALSTLVVNR;LENLQASDLGQVGEVVITK;LENLQASDLGQVGEVVITKDDTLFLK;LVQNVANNTNEEAGDGTTTATVLAR;NVILEQSWGSPK;TANSDQATGVEIVKK;TLVDELEVIEGMK;VEFQDALVLFSEK | 0.4241 | 69.4288 | 22 |
| Voltage-dependent anion-selective channel protein 3 (Fragment) | DFNLHTNVDNGK;DFNLHTNVDNGKDFGGSIYQK;FALDADASLHAK;GYHFGVFK;KANDVFGK;LRPGVVLTLSAAIDGQNFNAGGHK;NNFALGYQSK;SETGVEFSSGITSNQESGK;SLIGLGYQQK;TKSETGVEFSSGITSNQESGK;VTLEGTFAPQTGSK;WNTDNTLATDITIQDK;WTAGSADTLFGVGAK | 0.4877 | 53.7913 | 22 |
| ATP synthase subunit alpha | APGIIPR;AVDSLVPIGR;EAYPGDVFYLHSR;EVAAFAQFGSDLDAATQQLLNR;HALIIYDDLSK;IVTDFLATFTQAAQ;KIVTDFLATFTQAAQ;QMSLLLR;TALAIDTIINQQR;TGAIVDVPVGEQLLGR;TSHQGLLATIAK;VVDALGNAIDGK;VVDALGNAIDGKGPIDTK | 0.2585 | 47.8452 | 20 |
| Dolichyl-diphosphooligosaccharide--protein glycosyltransferase subunit 1 | ALLPYPTAITQQEDQLVK;DAINSGATPLVTVETVFTK;DSVELDLRPR;IIDHIFDDMQVDEVVTK;IILPEGSTSVK;KNVVENHIQDFQLR;LIEVSHWGNIAVEEVIEVEHTGAK;LVDISSQLVK;NVVENHIQDFQLR;THYTLGYNVPSYEYLYQSGSDYLLK;YDYQQDHHSGPASVR;YIGNLYTYSPYYITSMK | 0.3838 | 48.7007 | 20 |
| NADH-ubiquinone oxidoreductase 75 kDa subunit, mitochondrial | ALAQSLQKPVSGK;ALSEIIGER;AQQTLAAVSPPGK;ATYVNAEGR;FEAPLLNAR;FTDIHFSGK;LLAVAGDLADAESLVALK;LPYDSLDEVRDR;LSQISPALVTYGNVEDNNYFAQAR;RIDSVDVLDPLGSNIVVATR;TASQVAALDIGYKPGVGPVLQDGPK;TNEEINEEWLSDK;TPEGAAILAYTQELALR;VDLTYDYTHVGDSASILK;VLNVLQR;VVYLLGADSGVVSR | 0.3151 | 65.1681 | 18 |
| Vacuolar proton pump subunit B (Fragment) | AVVQVFEGTSGIDAK;FSEIVQLR;GPPILAEDFLDIQGQPINPWSR;IPASILAEFYPR;IPIFSAAGLPHNEIAAQICR;IYPEEMIQTGISAIDVMNSIAR;LALTAAEFLAYQCEK;NFISQGNYENR;QIYPPVNVLPSLSR;RIPASILAEFYPR;TVFESLDIGWQLLR;TVSGVNGPLVILDEVK;VFNGSGKPIDK | 0.3892 | 57.0533 | 18 |
| Dolichyl-diphosphooligosaccharide--protein glycosyltransferase 48 kDa subunit | ETHSQFFK;EYPHAVGR;FIDDGGNLLMAGNAAAGDVYR;IEIEELKNGK;KKPNGVYEAIFK;KPNGVYEAIFK;KTVLIAALQAR;LADDPNLVLSK;LSQWAFGER;LYHATQVSVRPLQHTQYER;VPDVWGVYQFK;WQPFDANDVQLEFVR | 0.3111 | 46.3220 | 16 |
| SFRICE_019251 | ASYFGFYDTAR;DFLAGGISAAVSK;EQGLLSFWR;GAFSNVLR;GTGGAFVLVLYDEIKK;HTQFWR;IPKEQGLLSFWR;LAADVGKGDGQR;QVFLGGVDKHTQFWR;SDGLIGLYR;TAVAPIER;TEGTGAFFK;YFAGNLASGGAAGATSLCFVYPLDFAR;YFPTQALNFAFK | 0.5067 | 55.5876 | 15 |
| Heat shock protein 83 | ADLVNNLGTIAK;ALLFVPR;APFDLFENKK;DQVANSSFVER;EDLTEYLEEHK;EKYTEDEELNK;ELISNSSDALDK;HLEINPDHSIVETLR;LGIHEDSQNR;NADDITQEEYGDFYK;SEGTLTIIDTGIGMTK;SLTNDWEDHLAVK;YESLTDPSK | 0.2120 | 50.0084 | 15 |
| SFRICE_010867 | ALEDAVEAEK;ALEDAVEAEKK;AQGQELLIEAK;ENVLLQLEAAYR;EVDTIENEWNSSR;FGPSLAAWLDK;KENVLLQLEAAYR;LGFIPEEWFQFFHSK;LMNAYTEVKR;QMVDWIVSNVTK;TGVTGPYTFGVGLTTYLFSK | 0.4754 | 46.0096 | 15 |
| SFRICE_000420 | ADVDIAVK;AGTVWVNDYNVLSNQVPFGGYK;ANNSEYGLAAAVFTQDIDK;ENGPYGINNYTEVK;FSQLDEVVER;GSDTGYFVQPTVFSDVK;ILSLIESGK;ILSLIESGKK;LGSPWR;TENPATGQAIADVQQAGK;VVGDPFKPETEQGPQIDAEQQNR;YYAGWADK | 0.3045 | 51.4963 | 14 |
| SFRICE_010943 | DGTGFISAGDFR;DLGLFGLYK;FITSEDFVR;GVLIQILESTYR;HEGIFGLYR;IVYNDLNSITPEQYFK;LTVNDFVR;SGQTTYNGVIDAAR;SSPQFGVTLVTYEILQR;TGSFIGEVAYR;VINASGFPHGER;VYLNATNGTR | 0.2023 | 51.8588 | 14 |
| SFRICE_001000 (Fragment) | AEITPVTLTVK;AGIPQDKPALGVNR;DEVDNFALR;EGLVTAGTASGISDGAGAIVLAGEEAAK;ISVAGGVENMSQAPFAVR;ITAHLVHELR;ITAHLVHELRR;LNVNGGATALGHPLGASGSR;NTTATELQTAATVAALK;RDEVDNFALR;VEVDEHPRPQTTIEGLK;VEVDEHPRPQTTIEGLKK | 0.3779 | 47.0832 | 14 |
| Enoyl-CoA hydratase | GVGQIQTGLANSVK;HAILATNTSAIPITK;HPGTSDDTAAAAVGVGLR;ILHSPLEGDVGAVFGLGFPPFTGGPFR;ILSTMLSEAVR;LGIVDLLVSPLGPGLGNPEENTMR;LPALTSVPTTLDLALTGK;MQLLEIIR;NMIFNK;TGFGLPEVMLGLLPGGGGTQR;YLANLLPTLEYEK;YLETVAIQVAR | 0.1554 | 45.6003 | 14 |
| V-type proton ATPase subunit C (Fragment) | EFAYNEADLAAGK;FPIPDLK;ITDMIVPR;KVVEEFK;LDTFVEGVTR;NIADIISK;QVGQIDADLK;VAQYLGEVLEDQR;VAQYLGEVLEDQRDK;VGTLDQLVGLSDDLGK;VGTLDQLVGLSDDLGKLDTFVEGVTR | 0.2117 | 38.6498 | 14 |
| Adipocyte plasma membrane-associated protein (Fragment) | KGQSEVFADGLPGTPDNARPLPDGSGILVALYTVFEEDRPPLTK;LLGPEAFQLYK;LTPQGHVTFVTK;SLGHISDAIVFNGK;VETKPVQQPPVK;VGPLATNGALNNGGK;YYLSGPK | 0.1606 | 29.9595 | 14 |
| P-type Ca(2+) transporter (Fragment) | ADEGLISGWLFFR;EFTLEFSR;EIVPGDVVEVSVGDKIPADIR;FSVPVVLLDEVLK;GVVIGTGLNTAIGK;IDQSILTGESVSVIK;IGVFTEDEDTTGK;LDEFGEQLSK;NILFSGTNVAAGK;TEMSETEEIK;VGEATETALIVLAEK;YGPNELPAEEGK | 0.1533 | 48.7042 | 13 |
| SFRICE016243.2 (Fragment) | AKFEELNMDLFR;EWSDSTVQHDVK;FDLTGIPPAPR;IFAPEEISAMVLTK;IINEPTAAAIAYGLDK;IINEPTAAAIAYGLDKK;ITPSYVAFTADGER;IVITNDQNR;KSLEDVVQPIIAK;MKETAEAYLGK;NELESYAYSIK;NQLTTNPENTVFDAK;SLEDVVQPIIAK;VTHAVVTVPAYFNDAQR;WLEDNQDVDSEEYKK | 0.2595 | 49.2095 | 13 |
| SFRICE_008643 (Fragment) | EEVTFAHTSAK;ENFHGLVGIR;EQTEVTFAVSDKDDFTHELNEYGIDYAK;KDEVLVIGFFEK;KGEVSQEYNGPR;NDVVLFRPK;TVADYEAFLK | 0.2771 | 29.8371 | 13 |
| Calreticulin | FYALSR;FYNDPEEDKGLQTSEDAR;GPWVHPEIDNPEYTPDANLYK;HEQDIDCGGGYLK;KPEDWDDRPTIPDPEDK;KVHVIFSYK;RDELCAVGLDLWQVK;SGTIFDNFLIGDSIEEAAER;VESGDLEADWDFLPPK | 0.3383 | 36.9033 | 12 |
| 2-phospho-D-glycerate hydro-lyase | AAVPSGASTGVHEALELR;DALFLIQDAIQQAGYTGK;EIDDFMLK;FGLDSTAVGDEGGFAPNIQNNK;IATAVEK;LGANAILGVSLAVAK;MGSEVYHYLK;TPIQIVGDDLTVTNPK;VNQIGSVTESIDAHLLAK;YNQILR | 0.3187 | 38.1175 | 11 |
| SFRICE_002276 | DHDFIVNLDQR;GKHIIEQLGGK;HIIEQLGGK;IVLAVFR;MGNPWQPFLNLLNR;NLIEKPEDHQVAK;SDLHFYLSWLK;SDLHFYLSWLKDQLK;TLVHLLER;VMYLLSHEDPNVR | 0.1688 | 38.7010 | 11 |
| SFRICE_000291 | AAQLGSAHEVQR;DLISWVSDIR;DLSTVQTLLTK;DLTGVQNLLK;LLDSLQAQQLFR;LQHLQQQWTQLQEK;LQQALVLVQFLR;LWELLLSR;RDEITLAWQK | 0.0406 | 35.2097 | 11 |
| SFRICE_003762 | ALNALCGPLFVELGQAVR;AMEIVLTGNFIDATEAER;EGMTAFVEK;EMQNNTFSHNTK;FGQPEINIGTIPGAGGTQR;IGTHSPLIVK;LLEETIK;NVGLIQLNRPK;VFPVDK | 0.3780 | 36.9769 | 11 |
| SFRICE_003397 | EKPLVLYVFSK;FDYIFFTGGTSIGK;KVLQEWYGPEPQK;LQALVDASKDK;QKFDYIFFTGGTSIGK;QQNVAEAVER;SIIHNLEDWAKPEKPEK;VLQEWYGPEPQK;YLDNDAIIVVEGGPQETTELLK | 0.1770 | 33.0505 | 11 |
| SFRICE_002792 | AGALLLVLPK;APQVVTTTPPNAGVTGALER;EPEFTLYGPTR;HWYTYLSSQPR;INLADELLAWQHER;KINLADELLAWQHER;NLADNIALTAR;SASFNLEGR;WSPDFEDILR | 0.1764 | 38.5970 | 11 |
| SFRICE_012231 | AGMYWLR;IDAVTVQNVR;IPIHELDAR;LSVLDNGIR;SDLQNYIK;SQGGGVNNASYLAR;SQTDLELLVENMGAHLNAYTSR;YTGSEIR | 0.1845 | 31.0390 | 11 |
| ATP synthase subunit O, mitochondrial | APVQVFGLEGR;GEVTCEVVTAKPLDQTQR;LDKLEAVINAFK;LEAVINAFK;LSPTTSNLLGLMAENGR;QNLEAALK;QNLEAALKK;TLDAVEKELSQFQQSIK | 0.4019 | 36.1251 | 11 |
| SFRICE_023375 | AVWWTEHVLR;ILAVFPNPSISHQVVFRPLTQELAR;ITEFYNDFMLNR;LTVEDFTNAINTVINDK;RGHEVTVITTDPVFPEGK;TLIQDEPMSPLER;WLPQPDLLIHPK;WNGDVLPGQTPNIK | 0.1537 | 30.3220 | 11 |
| Citrate synthase | LHPMSQFSAAVTALNSESSFAK;LVAAVYK;NPWPNVDSHSGVLLQYYGLK;QVGDNFTEEGLKEFIWK;SGQVVPGYGHAVLR;VVPPILTELGK | 0.1957 | 24.0663 | 11 |
| Heat shock 70 kDa protein cognate 5 | ALQDAEVQR;DAGQISGLNVLR;EQQIVIQSSGGLSK;ERVEVSNQAEGVLHDTETK;LFEQAYKK;NAVITVPAYFNDSQR;NTTIPTK;QAVTNSGNTFYATK;SKLESLVGDLIK;SQVFSTAADGQTQVEIK;VEVSNQAEGVLHDTETK | 0.1851 | 42.8276 | 10 |
| Pyruvate carboxylase | AGTHVLAIK;LDTNIALQTVSEYSAYWEQAR;LQVEHTITEEVTGIDLVQSQIR;QLIPNIPFQMLLR;SVVEYLQGAIGIPHGGFPEPLR;VFDSLNYLPNLILGMDAAGK;VISHAHDLPSSAAK;VTTEDPANNFQPSTGR;VVGDLAQFMVQNK | 0.1616 | 38.2090 | 10 |
| Elongation factor 1-alpha | EHALLAFTLGVK;IGGIGTVPVGR;IGYNPAAVAFVPISGWHGDNMLEASTK;LPLQDVYK;QTVAVGVIK;VETGILKPGTIVVFAPANITTEVK;YAWVLDK;YYVTIIDAPGHR | 0.2376 | 34.8377 | 10 |
| SFRICE_004571 | AALNAFLYR;FSLLEAVK;IGKPTLVNETSR;IKEYEAAIEQAK;KPSDALAGVVLAPTLER;LYFDTFVLQPASEGK;TTILEGIK;VFDWANTSK | 0.1438 | 34.3527 | 10 |
| Prohibitin | AAQFGLILDDISITHLTFGK;AAVIAAEGDAQAAVLLAK;AVVAQFDAGELITQR;DLQNVNITLR;IEAAEDIAYQLSK;ILFRPIPDQLPK;NVTYLPQGQNVLLNLPTQN;VLPSITSEVLK | 0.4307 | 33.5503 | 10 |
| SFRICE_032354 | KTYDSAEDYNGGR;NDYLAILAR;SADAFVDAALSAVK;SSDSDVITLTDDNFK;SSDSDVITLTDDNFKK;TSSDIVSFALEK;TYDSAEDYNGGR;VGALDATVHQEMAGR | 0.2453 | 31.9210 | 10 |
| Elongation factor Tu | AHDNVEAAVYILSK;EHLLLAK;ELDKPFLLPVESVHSIPGR;INNNLSEEDR;LGDLTLGTGVITK;SPEIGSEAISQLLK;TLDEAQAGDQLGALVR;VLADVNLAQK | 0.2215 | 33.3678 | 10 |
| UDP-glucuronosyltransferase 40Q2 | ELSFIYHHR;HLLEAGHEVTYITAHPYK;ILVAFAYPGK;LPTNYVGIGGYHIQDEIPPLPK;SETIPVELK;SHSILGEGYVR;TQLLNVFSK;WAPQPSILAHPNAK | 0.1900 | 30.9790 | 10 |
| Ubiquitin-40S ribosomal protein S27a | EGIPPDQQR;ESTLHLVLR;IQDKEGIPPDQQR;TITLEVEPSDTIENVK;TLSDYNIQKESTLHLVLR | 0.3013 | 18.3129 | 10 |
| Ubiquinol-cytochrome C reductase | HRTENEISTEQAVPVDTLR;QSFTYLIAGAGGVAGAYAAK;SIVTHFVSSMAAAADVLALAK;TENEISTEQAVPVDTLR;TENEISTEQAVPVDTLRDPQHDNQR | 0.2509 | 22.5782 | 10 |
| Malate dehydrogenase | DDLFNVNAGIVR;GPDELPSAIADSHVIVIPAGVPR;IALPELK;IALPELKK;IEVLTK;ILGVTTLDIVR;LNAYEEK;RIQEAGTEVVK;VMVAGAAGGIGQPLSLLLK | 0.2845 | 31.8101 | 9 |
| NADH dehydrogenase [ubiquinone] iron-sulfur protein 3, mitochondrial | ADQAGPQAETRPTVAK;DFPLSGYVEVR;DNHSAQFTNLVDIAGMDVPNRPNR;EIWDMYGVFFANHPDLR;FEIIYNLLSLR;LHLIDFGK;RFELSAPWEQFPNFR;SNPASEEVVNPADEK;VVVEPLELAQEFR | 0.4906 | 37.2010 | 9 |
| SFRICE_000735 | DHDVDLYIK;FYLTGPDAQR;GVNAVLLER;GYHLWNSDLR;NGEPVGYLR;TADLAFTADLSK;VAVFGQEAIFR;YAFFTLDNK;YTDAGLSNDAFPVNTHR | 0.1049 | 32.7848 | 9 |
| 3-oxoacyl-[acyl-carrier-protein] reductase (Fragment) | AAAAPQQDLLHAVANILGIKDPSK;AEHEPELR;EPFRPDAPAYAAQVER;GTPGLASHVR;LAEETFAEAK;LGPVGGVFNLAAVLR;SEAAVAVLLQR;TEEAVEALTSLAAR;WDHSLEWSVAHFGNASR | 0.0509 | 36.5265 | 9 |
| SFRICE_007086 | AEEEFNIEK;HMMAFIEQEANEK;ICNTLESR;IQSSNMLNQAR;KIQSSNMLNQAR;LELIAQQLLPEIR;NLLDEAR;SLVESVLSK | 0.3142 | 30.1283 | 9 |
| ATP synthase subunit gamma | AARPYGEGAVQFYER;AEVAPPEDDPK;GLCGAVHTGVSK;HIISVCNEIGR;IASAILESGYEFASGK;LSEPGAENIK;QLYIAMTSDR;SVVSYQQSDLPLFSQK | 0.3424 | 32.0118 | 9 |
| V-type proton ATPase subunit a | AIYHTLNLFNLDVTQK;DLNPDVNAFQR;EMIDLEATFEK;EVNQNAEALKR;IEDLNTVLGQTQDHR;METLEDPPTYNR;QAEIDTPLEDPSSSDQVYK;SGSSVPPILNR;SVFIIFFQGDQLK | 0.1418 | 33.5120 | 9 |
| Glycine cleavage system P protein | DISAVLLQCPDTR;GLVAHEFIIDVR;IWPTVGR;LDALGLEVNVVPDVR;LHPQTLAVVLTR;TANIEPGDIAK;VHNATLVLDHGIR;VSFQPNSGAQGEYAGLR | 0.1202 | 27.2119 | 9 |
| Catalase | DPASDQLVNYKK;FSTVGGESGSADTVR;IAAGLAPFK;IFTQVHPDLGSK;LGANFLQIPVNCPFR;MVNAQGVAHWVK;VSVSNYQR | 0.1637 | 29.2807 | 9 |
| Aspartate aminotransferase | AQWLADVK;EFHIYLTK;HGHQIILAQSFAK;ILPFFDMAYQGFATGSVDNDAFAVR;IVTEILSNPDLK | 0.2619 | 20.8623 | 9 |
| SFRICE_008303 | EAAFSLAEAK;FTTGDFNQVVLQNVTK;KQAALLAGQDLADAANLLDEGDEDLLF;LAIFPSR;LLVELASLQTSFVTLDEVIK;RVNAIEHVIIPR;TLAYIISELDELEREEFYR;VNAIEHVIIPR | 0.4512 | 33.7600 | 8 |
| SFRICE_013096 (Fragment) | AEAALAYELQAAK;AQFDQEVNTAK;DVYDDVQYLASLGK;IAAEVSAPLAK;LPAEAEAYR;QIEVEQQEILR;TDEIVLVGDSGATGEVAR;TLTGLDLSQVLK | 0.3037 | 30.3557 | 8 |
| SFRICE_032560 | DDANYLLR;IASYYIK;LHQALVAK;LRPLLAILEQPSQYK;LTDFDTHYR;SPGYVQLK;TAYEWKDDANYLLR;VFDTIFQAVHK | 0.1412 | 33.0654 | 8 |
| MICOS complex subunit MIC60 | AAAKPWLDAAR;GDTDLLLVYTLK;IQYLQNQLAELQTVR;LDTFDIINR;SQSLWAAAEALLAATR;YHMDHGNLPAALR;YVNLLQGAPR | 0.1183 | 29.2807 | 8 |
| Alpha-1,4 glucan phosphorylase | EIWGVEPSWEK;FFNSGDYIQAVLDR;FLHLADYDAYIEAQEK;IGEDWTVHLEK;SGFFSPGEPGR;VAIQLNDTHPALAIPELLR;VLYPNDNFFEGK | 0.1118 | 29.5172 | 8 |
| SFRICE_008127 | ETHFFLR;GNIITVLFIK;IIANTGTEK;IVDELIPSVLR;LLDYFLGEEIGTHYNR;QDVIQHIK;VTDHVNDLDKEEVNIISGALDLR | 0.1167 | 26.9519 | 8 |
| Peptidyl-prolyl cis-trans isomerase | DTNGSQFFITTTK;HYGAGWLSMANAGKDTNGSQFFITTTK;ILEGMDVVR;MGDENIGDIVIGLFGK;TEVVSEPFSVTK;TVENFVQLAQKPEGQGYK;TVTAANDRPVK | 0.4537 | 31.6094 | 8 |
| Dihydrolipoyllysine-residue acetyltransferase | FVTFTASADHR;ILIPAGTK;LGGQGSGLYGSLK;QTIPHYQLTATVNVEK;VIDGAVGAQWMK;VPTVNSHWMDSFIR;VSMNDFIVK | 0.1663 | 28.3404 | 8 |
| Fructose-bisphosphate aldolase (Fragment) | ATVTALLR;AWAGKPENVLAGQQELIK;IVPIVEPEVLPDGEHDLDR;RPWALTFSYGR;TYTPMDIGR;VTEVVLAAVYK;YASICQSQR | 0.3586 | 26.2209 | 8 |
| SFRICE_003274 | ELLDENGPK;IWLRPGQELSVK;LSVEGEANVDLLR;VADISVR;VNIAWESEIVTNIATISGGSGR;VTLLDAIGR;VVPLKPGVITIDVR | 0.0501 | 22.7117 | 8 |
| SFRICE_021217 (Fragment) | APDAVVEQR;DLALYALGIGASVLNEGDLK;GSSAISITNSEIYQNK;RAPDAVVEQR;SYSVDTLDK;TQQHIFVLGQGGFNGPR;TWTLDLK | 0.1408 | 28.1872 | 8 |
| 97 kDa heat shock protein | EFSVTDVQPYAVR;HASTTLNQDEAVSR;NALLDAANIAGLNVLR;NALNWLESAR;NLIEQVFNK;NTVFGFK;YAHLADVDVQK | 0.0962 | 26.5234 | 8 |
| SFRICE_002773 | ELKEDLFSK;HGILTESALTLAK;IIAGDKAPLLK;ISQETTTTENAPEPYYHR;LIEEQYLPK;RNEVAEEDSEAVK | 0.1396 | 27.0938 | 8 |
| SFRICE_000152 (Fragment) | GIVDEQFILNR;LAEAWVEEAVTR;LFVALTGIQFAGSHLQELQR;LSEFGTIQEK;NPTAHLGLIFSEAGR;VTAFIVER | 0.1413 | 27.0938 | 8 |
| Glyceraldehyde-3-phosphate dehydrogenase | AGAEYIVESTGVFTTTEK;IGINGFGR;LISWYDNEYGYSNR;VIHDNFEIVEGLMTTVHATTATQK;VIPALNGK | 0.2169 | 19.2470 | 8 |
| SFRICE_011143 | DYGVLNEETGIPFR;GLFVIDDKQNLR;IGCEVIGASTDSHFTHLAWINTPR;LVQAFQYTDK;QITVNDLPVGR;TTAVVNGEFK | 0.2295 | 21.2908 | 8 |
| ATP synthase subunit beta | FTQAGSEVSALLGR;IGLFGGAGVGK;IINVIGEPIDER;IPSAVGYQPTLATDMGTMQER;TVLIMELINNVAK | 0.0952 | 20.7513 | 8 |
| H(+)-transporting two-sector ATPase (Fragment) | AIAELGIYPAVDPLDSTSR;FLSQPFQVAEVFTGHAGK;IMDPNIIGAEHYNVAR;SLQDIIAILGMDELSEEDK;SLQDIIAILGMDELSEEDKLTVAR | 0.4451 | 22.5782 | 8 |
| Triosephosphate isomerase | DIGANWVILGHSER;FVVGGNWK;QVTEIVETLKK;TATPQQAQDVHASLR;TEEVVFR;VAHALESGLK;VVLAYEPVWAIGTGK | 0.3226 | 29.8371 | 7 |
| SFRICE_013982 (Fragment) | ALLELQLEAEELYQTFQR;DLVFITNPDQR;IKPILFMNK;LWGENFFNAK;TGTITTFK;VFDAIMNFK;VNFTVDEIR | 0.1418 | 29.3316 | 7 |
| SFRICE_007807 | ELQLELFK;FYDVNEGAVLVDGQDVR;HVSFGYGPER;ILTFPDAYDTQVGER;LVLSNISFK;TILKDPAIVLLDEATSALDTNTER;TLLPFLWPR | 0.1085 | 23.3619 | 7 |
| SFRICE005413.2 (Fragment) | AAQLDPNNSDIYHHR;AFHAGEYDK;ASLFTQLENTER;STFYLLLGR;TELEMTHLFSLK;TYFSAFSEDPITK;YKNEALLLR | 0.1442 | 27.9933 | 7 |
| SFRICE_018970 (Fragment) | FGLYEVFK;GLAPLWGR;LGWAGVWK;MVQNEGYGTFYK;TATVGGIMGK;TLELLYK;YVVPKPR | 0.2048 | 24.83549 | 7 |
| SFRICE_026018 (Fragment) | DDASIQHGHYVVAGVPAR;EFDNVLK;EGNSFDDAGNR;GSDLAADHTYYSYWR;LMELLQR;TGHTGYVALFNPTEDDQR;VVDDASEAPGTGSSVFTVNEEGGR | 0.1727 | 26.8998 | 7 |
| SFRICE_013269 (Fragment) | AVHLVYEK;FDAWTILIR;LATSIDEFGR;LLTTIGDEAIFK;NSFNVDR;NVDLANWR;SWQASPSTLK | 0.0434 | 26.7552 | 7 |
| SFRICE_001599 | ITTGAQDDLKK;LINPTDIGVK;NFEQAIER;NPQQYIDLGAK;QIFVPAPDIK;VHLSPLK;VIWFAIGSVDSFER | 0.0916 | 24.4999 | 7 |
| Mitochondrial heat shock protein 75 kDa | ELISNASDALEK;EYSIFLK;IIVHLK;NLIVHINELLVK;SFIEEIKK;TDVPLSIR;YISQSYDKPR | 0.0889 | 24.2033 | 7 |
| Cytochrome P450 CYP4C1-like protein transcript variant 1 | ILTPTFHFNILK;LAMLDLLLEAEEK;LDNDQTSATVDYK;LYPSVPFIGR;SAPYDFLK;TTLSELVR;YLEAVIK | 0.1434 | 24.9137 | 7 |
| V-type proton ATPase subunit | AIIITINSFGTELSK;AVAEYYAEYQALFEGAGTNVGDK;AYLEAFYDFCK;LHLQGTDYGTFLANEPSPLSVSTIDDK;LVIEFQHLR;QADYLNLVQCETLEDLK | 0.2931 | 26.0524 | 7 |
| SFRICE_035634 (Fragment) | DPALVFFR;HGVPLLYSGESDENEIYGFFEK;LHAVWESVGATLK;LPSFLFFR;LYNPAKDPALVFFR;MDANLAGVNTAK | 0.2685 | 26.0524 | 7 |
| SFRICE_004313 | EAGFPPGVLNVIPGYGPTAGAAISHHPGIDK;EEIFGPVQSILK;ELGEDGILQYLENK;QAEGEVLWSAGIIR;SALVVFNDADVEK;TYVQSGIYDK;VAFTGSTEVGR;VTLELGGK | 0.2104 | 25.3779 | 7 |
| SFRICE_008973 (Fragment) | ALIELFESFK;AVNEALNSLLIDEEDYQGLR;EHLELFWSR;ESWEDLVR;LAITLVHLK;VGYTPDYIYLLR | 0.0512 | 26.0524 | 7 |
| SFRICE_000208 | ILEDVFPIYR;ILGIFPSLDR;IVLNPEFR;SHFFVFAPYLK;SILHMYFIYLHK;VQAIIDAVSEIPQR | 0.0410 | 22.5642 | 7 |
| ATP synthase subunit d, mitochondrial | ANLAAFK;INWAAYK;ISQSAVNWAALAER;SIEAFIQESNANIAK;VPYPADTQTAQVEAQWAEVK;VPYPADTQTAQVEAQWAEVKK | 0.3678 | 24.7408 | 7 |
| Annexin | ADAEALASAGEGQWGTDESVFNSILITR;AIIDVLCR;DENQGVDEGAAK;DIEETIKK;QIFAEYEALTGK;TISAFYEQLYNK | 0.2477 | 23.0456 | 7 |
| SFRICE_006893 (Fragment) | AVANILK;GLGNSLFISPK;KPSLNAAAIQAGK;QIDALSPNDVSQALSSAAANK;TPAEIAK;WGSDNSPLAK | 0.2509 | 22.6576 | 7 |
| SFRICE_025754 (Fragment) | AIMQLGYDVAPDGVK;AQQLIDHVAR;EPWTYANK;GADTIVQLIK;SVLPAVDWEPR;YGVFQTEASQADTQEFR | 0.1749 | 25.5222 | 7 |
| SFRICE_001276 | ENPGTLDEIHKK;GSDYTLAMAVGKPDFNEK;LALGTELVYQSGAR;LGATYIGTK;SAVFVGHYLQSVTK;VMGGEIAITSAAAR | 0.2571 | 24.6052 | 7 |
| Probable methylmalonate-semialdehyde dehydrogenase [acylating], mitochondrial (Fragment) | AVSFVGGDAAGK;EWIPDLVAR;LFIDGQFVESK;TLADAEGDVLR;TNNWIELTNPATNEVIGR;VNAGHVPATDVGPVISPK | 0.2188 | 21.6023 | 7 |
| SFRICE_002106 | EDTGALPDETSFNLVNEASVDDLNTR;GFPELLAVQPTIR;LNVNLAEIVALQKPK;LPEKWEQVGVLK;NANTEPLQELKK;NSVLTLEHPNMEK | 0.2725 | 23.8654 | 7 |
| Peptidyl-prolyl cis-trans isomerase | FGDENFVLK;HTGPGVLSMANAGPNTNGSQFFITTVK;HVVFGTVVEGMDVVK;TSWLDGR;VYFDVSADGSALGR | 0.4364 | 19.7100 | 7 |
| SFRICE_020749 (Fragment) | ASVVHLTEEVVGR;FEQTSQQGGTCTTADR;IIIAVGHSGYLR;VLAVPNDVEYIDEIEALNK;YSPIHPQIEGR | 0.0594 | 22.5782 | 7 |
| SFRICE_011011 (Fragment) | KGEIFELK;LAHANAAVVLSAVK;LASQANIAQVLGELK;RPADTQELVQHVLSLATQDSDNPDLR | 0.0904 | 16.0445 | 7 |
| NADH-cytochrome b5 reductase | AYTPVSSDDEK;AYTPVSSDDEKGYVDLVIK;EVQAAPPK;IDDDLVIR;VWYTLDRPSEGWK;YALPLIER | 0.1713 | 22.8118 | 6 |
| Carnitine O-palmitoyltransferase | EFEETIAVK;LLNDVDLR;LYSFQGSLPR;SHWAHIR;VLVYSR;YLELESPFLQEVFNEPWR | 0.0746 | 19.6057 | 6 |
| Prohibitin | DLQMVNISLR;EYTAAVEAK;FNASQLITQR;IVQAEGEAEAAEMLGK;QQVSLLIR;VLSRPDASSLPTMYR | 0.2337 | 25.5222 | 6 |
| Succinate--CoA ligase [ADP-forming] subunit beta, mitochondrial | DALLIEVNPYAEDAITGK;FDDNADYR;FHVATTADEVVK;LASQMLNQLLVTK;MYDLFLK;QKELFSLR | 0.1460 | 19.3443 | 6 |
| Isocitrate dehydrogenase [NADP] | DLVICIHGLK;FKDIFEEIYQSDYK;LDCLYYDLGLPHR;LIDDMVAQAIK;TVESEAAHGTVTR;VELVYTAEDGSVER | 0.1728 | 22.9689 | 6 |
| C-1-tetrahydrofolate synthase, cytoplasmic | DVEDIKER;IVGTPVSELLK;KITVGQSPTEK;TADILVVAIGR;TSNSLTGDPAVK | 0.0568 | 19.8209 | 6 |
| SFRICE_000170 | LVNLISPSYPLGGLHWEAIEK;NGLETIK;SDFYDAQR;YDPIMFPLK;YSNVVINLVGR | 0.1400 | 20.2032 | 6 |
| Transaldolase | ISLNEAQFR;LASTWEGIQAAR;LINMFAEYGIK;LLQELAASEQPLKR;VSVEVDAR | 0.1627 | 19.6607 | 6 |
| Adenosylhomocysteinase | ESLLDGIKR;GISEETTTGVHNLYK;HNVYPVGVHTLPK;KVNIKPQVDR;VNIKPQVDR | 0.1093 | 19.4659 | 6 |
| SFRICE_002919 (Fragment) | ADFAQAILDAGLR;AGNVVHLYER;ENDVDAVHPGYGLLSER;HIEVQLLGDK;TVEYKPIR | 0.1790 | 17.0767 | 6 |
| SFRICE_020830 (Fragment) | DYTWLR;LNFDQYTEREEPK;LNVPELITSADDAYER;VVVVEGPVAVGK;YSQYIDALTHLLNTGQGVVLER | 0.1063 | 16.8719 | 6 |
| Proton-translocating NAD(P)(+) transhydrogenase | AQYPIAELVDILK;AVIEAAAHFPR;EMSQEFLDAER;FFSGQMTAAGR;VAVVPAVVSK | 0.0534 | 22.5782 | 6 |
| UDP-glucuronosyltransferase | AGVPLLAVPVFGDQPANAER;GIVDALLQGGHTVTWVTPFPEK;QYEDLFAPIAAAR;VFITHGGQLSSLEAVR;VFTTRPTTPK | 0.1558 | 20.8058 | 6 |
| SFRICE_009010 (Fragment) | KLQQIGASVTASGDR;LQQIGASVTASGDR;TFVAALDNGSPVTR;YEPQTELGVAHMLR | 0.2687 | 18.0625 | 6 |
| SFRICE_005246 | ESLASTSQNATEALTR;ESLYLSK;IQAFEEELISVSR;LIGTINSLQR;YVSAESALEEFKK | 0.0455 | 19.0900 | 5 |
| SFRICE_026609 (Fragment) | AYLPVNESFGFTADLR;EGLPDLNQYLDKL;GVQYLNEIK;GVQYLNEIKDSVVAGFQWAAK;YLTDKYEYDVTEAR | 0.2678 | 19.1618 | 5 |
| SFRICE_002630 | DAVLLIFANK;ILMVGLDAAGK;LGEIVTTIPTIGFNVETVEYK;LGLHSLR;MLSEDELR | 0.3132 | 20.2494 | 5 |
| SFRICE_002195 | AADINVAEGK;ESLNVAIVDAINK;ILEPGLNLLWPIVDK;LIGGALAQR;SELGQISLDK | 0.1367 | 20.4860 | 5 |
| SFRICE_014874 | LVQDYFSWTGVDK;SIPYFWEK;VLDAHLLTR;VLLSESNAIAYYVSNAALR;VPAYESADGK | 0.1338 | 20.5921 | 5 |
| SFRICE027725.2 (Fragment) | AAIASYEKDEAVK;HLLVVPDLEK;LLLAFGDEGFEDHR;VAFNDWPEFKPK;VVDAVYTIPK | 0.2892 | 18.0756 | 5 |
| SFRICE_014294 (Fragment) | EGLLEIFGK;ELSALYHDR;HGVIYFSMGSFLK;QTVIWK;SPALHVPLYQR;VEELWSR | 0.0385 | 18.9257 | 5 |
| Staphylococcal nuclease domain-containing protein | AIIEYVR;EVIFTADKPPNSATR;LVELEDVAK;QDGESEPYAEEAR;QVLSGDTIVIR | 0.0615 | 19.2470 | 5 |
| SFRICE_030420 | APHLDALSFR;EAAPLGAVLPPYLAAEAAR;EDGVALR;VAVLGGGFLACELSAALAER;VVGVLLWNLFNR | 0.1053 | 20.2252 | 5 |
| SFRICE_000822 | DVATFLR;KGGEDYIFALLTGYMDAPAGVVLR;LAYKPVSK;NLVNVTHTEEEAK;NLVNVTHTEEEAKAEAAEAMIK | 0.1877 | 19.5185 | 5 |
| SFRICE_023417 (Fragment) | HTLAETNIETGETK;KHTLAETNIETGETK;LPFEQHWR;RDELDYSKPLDGQTK;YANGTISTADLR | 0.2762 | 19.4349 | 5 |
| SFRICE_036126 | DVDGSLLFIFK;DVVAPEQALVAWGGK;EGLFFTHGR;ILIYWFDR;SVGANDINESNIR | 0.1228 | 21.2908 | 5 |
| SFRICE_033731 | IKVEFIK;INALYVMR;LPPLVWPENETEEPRDEVEEK;VNTKDEILYQTER;YHHSTFGYDIPANR | 0.2377 | 19.5185 | 5 |
| Moesin/ezrin/radixin homolog 1 | APDFVFFAPR;FYPEDVADELIQEITLK;IGFPWSEIR;KAPDFVFFAPR;LFYLQVK | 0.0765 | 19.2199 | 5 |
| Casein kinase II subunit alpha | LIDWGLAEFYHPGQDYNVR;TPALIFEHVNNTDFK;VLGTEELFEYLDK;VYADVNSQRPR | 0.1643 | 18.0625 | 5 |
| 40S ribosomal protein S4 | ERHPGSFDIVHIK;LNNVFIIGK;VNDSVQLDIATSK;YALTGNEVLK | 0.1711 | 15.0593 | 5 |
| Actin (Fragment) | AGFAGDDAPR;AVFPSIVGRPR;DLTDYLMK;DSYVGDEAQSK;IIAPPER;SYELPDGQVITIGNER;TTGIVLDSGDGVSHTVPIYEGYALPHAILR;VAPEEHPVLLTEAPLNPK | 0.2952 | 18.0625 | 5 |
| SFRICE_014898 (Fragment) | ESNYHLLESVQESLER;IETGTIVGFPGAK;IIAEAANGPTTPAADK;NLNHVSYGR | 0.2547 | 15.5740 | 5 |
| Vesicle-fusing ATPase | AFEEADKNSPAIIFIDELDAIAPK;EIDIGIPDATGR;LAGESESNLRK;LDQLIYIPLPDEK | 0.0744 | 18.0625 | 5 |
| SFRICE_001400 (Fragment) | AYQALTDDEAR;ELFGDTTIK;QIIIEQSQPYK;SPLLQLPYITEDHLK | 0.0701 | 18.0625 | 5 |
| SFRICE_001242 | AVQSAAITDGSEEK;EFVDIEAK;LYKPLFEK;NEMLAAITNR | 0.1008 | 16.7751 | 5 |
| SFRICE_028969 (Fragment) | IRPELFVTGSR;KTEFDSEFESK;LLHTSYSEANAPGLHLLK;LYSLLK | 0.0830 | 13.1469 | 5 |
| SFRICE_003104 | KYSNDYYQLTAEYLK;TPVVVATIDQYAPGLVDNVQSYASTGFQTIK;VGVLYSNTK;YSNDYYQLTAEYLK | 0.0830 | 18.0625 | 5 |
| SFRICE_035016 | DGYKPGIK;HFGAGLR;TVLSEEDEPQPNGR;VDTIIYMEDQLK | 0.1152 | 15.6876 | 5 |
| Nucleoside diphosphate kinase | EIALWFGDK;HYSDLSSKPFFPGLVK;QMLGATNPADSLPGTIR;TFLMIKPDGVQR | 0.3529 | 17.0211 | 5 |
| SFRICE_004073 (Fragment) | ALSALMTFK;FFDMVEYFFHR;FSTDVSRDEVK;LKDIPTHANPR | 0.2500 | 15.3712 | 5 |
| SFRICE_018917 | FAVMGFTK;FVFLLDINEK;GGNGGTILNLASIYGYR;VDQYLPVYQASK | 0.0916 | 15.9798 | 5 |
| SFRICE_036980 | AMVVNAAQLSTYSQAR;EGFVALYTGLSAGLLR;LGVYNYLFDSYK;TLLGVAAGSVGAFVGTPAEVALIR | 0.3285 | 18.0625 | 5 |
| SFRICE_014997 | FSPNHANPIIVSAGWDR;IWDLESK;VWQVSVSAR;YPDMILSSSR | 0.1348 | 12.7723 | 5 |
| SFRICE_000903 | FTTFDLGGHQQAR;IDKPGAASEDELR;QFFNLYQQTTGK;QGYGEGFR | 0.2383 | 16.7751 | 5 |
| SFRICE_013406 | QIDVETVGER;SLVGVQK;TTYEEIKK;YIQATDLSEASQEAVEEK | 0.2848 | 14.5326 | 5 |
| Retinol dehydrogenase 14-like protein | EAIALIR;IDVLINNAGVVVPLNQDLK;IVVVSSTLHER;TFLITGANSGIGYETAK | 0.1612 | 13.7986 | 5 |
| Translationally-controlled tumor protein homolog | IEGFNPSAEEADEGTDTAVESGVDIVLNHR;LVDEVIYEVTGK;LVETYAFGDKK;SYTLYLK | 0.3488 | 15.6876 | 5 |
| SFRICE_000610 | ASVVFATTDK;GISAFIVPKPIK;ITEIYEGTSEIQR;LADMALQLESAR | 0.1152 | 17.0211 | 4 |
| SFRICE_004797 (Fragment) | FFQDIYAETTK;LPVPELSK;STNMLISALR;VFPLDMSQFVGLFGATR | 0.0817 | 16.7751 | 4 |
| SFRICE_003674 | GKEEGVWHIDLK;LCLPYLK;LPGTIYTAAEEIEALGGK;TAIATAAIEMLTGTSSTSR | 0.1315 | 14.3204 | 4 |
| SFRICE_002973 | AEGPFAFYK;FFVMESLR;FGVFEQAR;LYMLTEDGTLSTTGK | 0.1266 | 16.0764 | 4 |
| SFRICE_003033 | DALQNLNDR;IDSLNATLNDLENK;LQQSLQELR;NSTAATVAVEELR | 0.0731 | 15.9703 | 4 |
| SFRICE_002689 | AIEQSFISIEEK;HVVITGGSSGIGK;NEDLTKPEETK;VQHVALDVTSDYK | 0.1489 | 17.0211 | 4 |
| Glycerol-3-phosphate dehydrogenase | SSYYLSK;TALVEADDFASGTSSR;TAVDMIAR;WWQVPYYWFGIK | 0.0570 | 13.3851 | 4 |
| SFRICE_000701 | DTVAHTFDSTK;DTVASTAQSTVDSTK;DTVASTVQATVDTTK;ETVANTVHSTIDTTK | 0.0546 | 16.3467 | 4 |
| Aconitate hydratase | DGIAQTLR;FLETAGAK;LSDPFADELPAR;VAGILTVK | 0.0621 | 10.4789 | 4 |
| 5-aminoimidazole-4-carboxamide ribonucleotide formyltransferase | ITADLFK;TIFGLTLEQR;TLHPAVHAGILSR;VALASDAFFPFR | 0.0707 | 13.1830 | 4 |
| SFRICE_006747 | FLGLTGSK;IGGQFELVNTEGK;LPPVQPLFISVDPQR;NTDFLGK | 0.1693 | 16.4126 | 4 |
| V-type proton ATPase subunit a | DLNPDVNAFQR;LGFVAGVVNR;QNYLELTELR;SLISDETGQQAATR;TVFVAFFQGEQLK | 0.0949 | 18.0625 | 4 |
| Aminomethyltransferase | ALIALQGPK;GGIIDDLIVTK;VNAETLYVVSNAGR;VVDFAGFLLPVQYSDLSLSESHLFTR | 0.1456 | 17.0211 | 4 |
| 40S ribosomal protein SA | ADGTHVINLR;AVVAIENPADVFVISSRPFGQR;EQVVAVAPPK;FTPGAFTNQIQAAFR | 0.1857 | 14.7890 | 4 |
| SFRICE_000373 | EYLELKR;LEPVSVLDMVR;LLIAAQHQK;YEALALNNK | 0.1118 | 15.7338 | 4 |
| Alkaline phosphatase (Fragment) | LQSLAALALDANK;QFWLDLAHNELEEALK;SAGFVTTMR;VTHATPSPLYAHSASR | 0.108 | 16.3467 | 4 |
| Electron transfer flavoprotein subunit alpha | AAVDAGFVANDLQIGQTGK;GLTPETLTPLILATQK;LDVSPITDIIGVK;QFNFTHILAPATAFGK | 0.1939 | 15.6876 | 4 |
| SFRICE_009042 | AADPVDRPEVR;DVESMLGYVEDQR;ELNEDNWR;FVVTALPTIFHVIDGEFR | 0.1416 | 16.0445 | 4 |
| SFRICE_014941 (Fragment) | AELHTLQAR;AQAVAELQNEVQK;AQIAENNLELLKEESEK;DAAALAQQHAADLAQQLQDANR | 0.1132 | 17.0211 | 4 |
| UDP-glucuronosyltransferase | GIWWIEHVLR;HFGPDIPHITELQNR;ILAVYPVPSISHQITFRPITLELIK;IQTLINAFSK;TYLDSSK;WLPQSDLLK | 0.1467 | 16.0188 | 4 |
| SFRICE_024442 (Fragment) | AAVAAAALPGVR;LNVLIDR;LQLTAALR;TNVVQSLLAR | 0.1276 | 15.3712 | 4 |
| Complex I-B22 | EGEDELFK;LFPTSQGGVAHQR;NLEAYYDRR;VVTPPDWVIDYWHPLEK | 0.3241 | 12.4002 | 4 |
| ER membrane protein complex subunit 4 | ALFATQSTFK;DTDSNLLLIK;KLWDVALGPLK;VEHIGGGLSLI | 0.2442 | 16.7751 | 4 |

Table S26 Identified *Spodoptera litura* larval coded proteins associated to HvAV-3h virions (Sl-1)

| Protein ID | Identified peptide sequence | Sequence coverage | Unique Peptide |
| --- | --- | --- | --- |
| Heat shock protein 70 cognate | QTQTFTTYSDNQPGVLIQVFEGER;ARFEELNADLFR;LLQDFFNGK;TFFPEEVSSMVLTK;KFEDATVQADMK;FEELNADLFR;DNNLLGK;QTILDK;IINEPTAAAIAYGLDKK;FELTGIPPAPR;LLQDFFNGK;ISDSDKQTILDK;MVNHFVQEFK;HWPFEVVSDGGKPK;QTQTFTTYSDNQPGVLIQVFEGER;DAGTISGLNVLR;TFFPEEVSSMVLTK;NQVAMNPNNTIFDAK;ELEGICNPIITK;STAGDTHLGGEDFDNR;SQIHDIVLVGGSTR;ETAEAYLGK;TVQNAVITVPAYFNDSQR;APAVGIDLGTTYSCVGVFQHGK;MVNHFVQEFK;LSKEEIER;KFEDATVQADMK;IINEPTAAAIAYGLDK;WLDSNQLADKEEYEHK;FEELNADLFR | 0.5590 | 30 |
| ATP synthase subunit beta | VVDLLAPYAK;IGLFGGAGVGK;EGNDLYHEMIESGVISLK;LVLEVAQHLGENTVR;IINVIGEPIDERGPIPTDK;FLSQPFQVAEVFTGHAGK;TVLIMELINNVAK;TIAMDGTEGLVR;IMDPNIIGAEHYNVAR;IPVGAETLGR;AHGGYSVFAGVGER;VALTGLTVAEYFR;LVPLEETIK;TAAIHAEAPEFVDMSVQQEILVTGIK;FTQAGSEVSALLGR | 0.4225 | 15 |
| Heat shock protein 83 | ADLVNNLGTIAK;DQVANSSFVER;ELISNSSDALDK;GVVDSEDLPLNISR;ELISNSSDALDKIR;APFDLFENK;YESLTDPSK;EGLELPEDEEEK;SEGTLTIIDTGIGMTK;IVLHIK;YYEQFSK;ALLFVPR;LGIHEDSQNR;YTEDEELNK | 0.2064 | 14 |
| Glyceraldehyde-3-phosphate dehydrogenase | VIPALNGK;VPVANVSVVDLTVR;IGINGFGR;LISWYDNEFGYSNR;LTGMAFR;EAAQGPLK;LGKPASYDAIK;IGINGFGR;IAVFCER;VIHDNFEIVEGLMTTVHATTATQK;GAQQNIIPAATGAAK;VIPALNGK;AGAEYIVESTGVFTTTEK;VIDLIK | 0.4699 | 14 |
| ABCC1-like protein | TALTSAIYR;NINLHVPR;VLGPSGLLK;AHANIPDDENVK;GSLVAIVGAVGSGK;LMNEVLNGIKVLK;ALEYAHLKPFVQGLPAGLR;HEVAEGGENLSVGQR;SSLLSALLGEMNK;LTLAYR;STLTLGLFR;TSASVNFKPESEK | 0.0923 | 12 |
| Catalase | DAAGFIQER;TVDNIVGHLK;AAELASSDPDYSIR;DLYNAIAK;VSVSNYQR;LFSYSDTHR;FSTVGGESGSADTVR;IFETVGK;DPASDQLVNYK;IFTQVHPDLGSK;NGPTLLQDVNFLDEISSFDR;DASLFPSFIHTQK | 0.2682 | 12 |
| Elongation factor 1-alpha | MDSTEPPYSESR;EHALLAFTLGVK;LPLQDVYK;IHINIVVIGHVDSGK;EVSSYIK;YYVTIIDAPGHR;IHINIVVIGHVDSGK;VETGILKPGTIVVFAPANITTEVK;IGGIGTVPVGR;MDSTEPPYSESR;QTVAVGVIK | 0.2959 | 11 |
| Ubiquitin | IGYNPAAVAFVPISGWHGDNMLEASTK;TITLEVEPSDTIENVKAK;TLSDYNIQKESTLHLVLR;EGIPPDQQR;TLTGKTITLEVEPSDTIENVK;MQIFVK;MQIFVK;TITLEVEPSDTIENVK;TLSDYNIQK;IQDKEGIPPDQQR;ESTLHLVLR | 2.0526 | 11 |
| Arylphorin subunit | QYVKPYNHNDLHFVGVK;VGKDYDIEANIQNYSNK;LGEVFFYYYQQLLAR;TFFQFLQK;IIDYLIDYK;DLHQYSYEIIAR;FSIFYER;LVPGENKIER;FYELDWFVQK;FLDTYEK;DPAFYQLYQR | 0.1748 | 11 |
| Thioredoxin peroxidase | PLQLTKPAPQFK;QITVNDLPVGR;DIALSDYK;QGGLGPMNIPLISDK;LVQAFQYTDK;QITVNDLPVGR;IGCEVIGASTDSHFTHLAWINTPR;GLFVIDDK;TTAVVNGEFK;SVEETLR | 0.5949 | 10 |
| Putative acetyltransferase ACT5 | LNVKPLAR;TPIGSFK;APSNLSLNEVVIVSAVR;INVHGGAVSLGHPIGMSGTR;AFDAEVIPVPVPQK;EPIDFPIAPSVAIPK;GGAPVIFAEDEEYKR;KQQDDYAIISYK | 0.2720 | 8 |
| Translationally-controlled tumor protein homolog | LEEKAPDQVDVFK;LVETYAFGDK;IEGFNPSAEEADEGTDAAVESGRDIVLNHR;LVETYAFGDKK;IEGFNPSAEEADEGTDAAVESGVDIVLNHR;HGLEEEKF;LVDEVIYEVTGK;APDQVDVFK | 0.7151 | 8 |
| phosphopyruvate hydratase | LGANAILGVSLAVAK;GVLTAIK;QNLEVTQQK;TPIQIVGDDLTVTNPK;DALFLIQDAIQQAGYTGK;NGTYDLDFK;SNPADYLSSEK;FGLDSTAVGDEGGFAPNIQNNK | 0.2471 | 8 |
| Nucleoside diphosphate kinase | TFLMIKPDGVQR;NIIHGSDSVESAK;QMLGATNPADSQPGTIR;GLVGTIIER;HYSDLSSKPFFPGLVK;EIALWFGDK;GDLCIEVGR | 0.4775 | 7 |
| Complex I-49kD | TYTQALPYFDR;LDEVEDVLTTNR;APGFAHLAALEK;HPPYHGVVAPGEK;TLFAEITR;LVLELDGETVR;LLNIDIPLR | 0.1634 | 7 |
| Actin | SYELPDGQVITIGNER;DSYVGDEAQSK;IWHHTFYNELR;VAPEEHPVLLTEAPLNPK;GYSFTTTAER;AVFPSIVGRPR | 0.2048 | 6 |
| Trypsin-like serine protease | IVGGSVTTIDR;LGSTWANSGGVVHNVNANLVHGSYNSR;AASIAGANYWPGDNSAAWAAGWGTTSSGGSASEQLR;LGSTWANSGGVVHNVNANLVHGSYNSR;AILTAAHCTVGDAVSR;AASIAGANYWPGDNSAAWAAGWGTTSSGGSASEQLR | 0.6024 | 6 |
| 14-3-3 zeta | YLAEVATGETR;DSTLIMQLLR;AYQDAFEISK;DSTLIMQLLR;NLLSVAYK | 0.1984 | 5 |
| Glutathione S-transferase sigma 4 | EYAQSLAISR;YADFVK;LNAIFVK;NVFPDLLEK;LLLAYGGQEFDDRR | 0.2255 | 5 |
| Cytochrome b-c1 complex subunit Rieske, mitochondrial | SIVTHFVSSMAAAADVLALAK;QSFTYLIAGAGGVAGAYAAK;TPNEISTEQAVPVDTLRDPQHDNQR;TPNEISTEQAVPVDTLR;QSFTYLIAGAGGVAGAYAAK | 0.3801 | 5 |
| Cytochrome c oxidase subunit 2 | IMVTATDVIHSWTIPSLGVK;IMVTATDVIHSWTIPSLGVK;LLDVDNR;NFINWINNYS;LLYLLDELNNPLITLK | 0.3216 | 5 |
| Mitochondrial NADH dehydrogenase iron-sulfur protein 8 | AAQTLFWTELAR;GFAVTLAHIFK;LLSNGDKWESEIATNIR;EPATINYPFEK;LLSNGDKWESEIATNIR | 0.3105 | 5 |
| Apolipophorin-3 | DAPPANTLLQDIEK;EVASNVEETNEK;TFSEQLNSIANSK;LQAAVQNTAQEVQK | 0.2819 | 4 |
| Glutathione S-transferase delta 4 | LYFDIGTLYQR;AIITYLVNK;ALNLNLNLK;AVLLTAK | 0.1667 | 4 |
| Glutathione S-transferase s2 protein | YVFHYFDGK;QYAQSLSIAR;AAIASYEKDEAVK;VVDAVYSIPK | 0.2059 | 4 |
| SP1 | GIVGGSVTTIDR;SASTFSFNNNVR;SASTFSFNNNVR;SASTFSFNNNVR | 0.2043 | 4 |
| Methionine-rich storage protein | ILYFAK;NLVVIDWR;VTDVFVLFK | 0.0306 | 3 |
| Chymotrypsin-like | STCQGDSGGPLVVTR | 0.0505 | 1 |

Table S27 Identified *Spodoptera litura* larval coded proteins associated to HvAV-3h virions (Sl-2)

| Protein ID | Identified peptide sequence | Sequence coverage | Protein Q score | Unique spectra |
| --- | --- | --- | --- | --- |
| 70 kDa heat shock protein | APAVGIDLGTTYSCVGVFQHGK;ARFEELNADLFR;DAGTISGLNVLR;DNNLLGK;ELEGICNPIITK;ETAEAYLGK;FEDATVQADMK;FEELNADLFR;FELTGIPPAPR;HWPFEVVSDGGKPK;IINEPTAAAIAYGLDK;IINEPTAAAIAYGLDKK;ISDSDKQTILDK;ITITNDK;ITITNDKGR;LLQDFFNGK;LLQDFFNGKELNK;LSKEEIER;MKETAEAYLGK;MVNHFVQEFK;NQVAMNPNNTIFDAK;NTTIPTK;QKETIQAK;QTILDK;QTQTFTTYSDNQPGVLIQVFEGER;SQIHDIVLVGGSTR;TFFPEEVSSMVLTK;TTPSYVAFTDTER;TVQNAVITVPAYFNDSQR;WLDSNQLADKEEYEHK | 0.4747 | 99.3764 | 53 |
| Ubiquitin (Fragment) | EGIPPDQQR;ESTLHLVLR;IQDKEGIPPDQQR;LIFAGKQLEDGR;MQIFVK;TLSDYNIQK;TLSDYNIQKESTLHLVLR;TLTGKTITLEVEPSDTIENVK | 0.9211 | 26.3397 | 36 |
| Heat shock protein 83 | ALLFVPR;APFDLFENKK;DQVANSSFVER;EDLTEYLEEHK;EGLELPEDEEEK;EKYTEDEELNK;ELISNSSDALDK;ELISNSSDALDKIR;GVVDSEDLPLNISR;HIYYITGENR;HSQFIGYPIK;IEDVGEDDEEDKK;IRYESLTDPSK;IVLHIK;LADLLR;LGIHEDSQNR;SEGTLTIIDTGIGMTK;YESLTDPSK;YHTSASGDEACSLK;YTEDEELNK;YYEQFSK | 0.2664 | 61.2240 | 22 |
| Putative acetyltransferase ACT5 | AFDAEVIPVPVPQK;AFDAEVIPVPVPQKK;APSNLSLNEVVIVSAVR;GGAPVIFAEDEEYK;GGAPVIFAEDEEYKR;GSLANVTATELGAIVVR;INVHGGAVSLGHPIGMSGTR;IVGHLCHALK;KQQDDYAIISYK;LLDIDLEK;LNVKPLAR;LPTVFK;QAAIFGGLEK;QQDDYAIISYK;TPIGSFK;VIGYADGEREPIDFPIAPSVAIPK | 0.4257 | 49.4187 | 21 |
| ATP synthase subunit beta | AHGGYSVFAGVGER;FLSQPFQVAEVFTGHAGK;IGLFGGAGVGK;IINVIGEPIDERGPIPTDK;IMDPNIIGAEHYNVAR;IPVGAETLGR;LVLEVAQHLGENTVR;LVPLEETIK;TAAIHAEAPEFVDMSVQQEILVTGIK;TIAMDGTEGLVR;TVLIMELINNVAK;VALTGLTVAEYFR;VVDLLAPYAK | 0.3605 | 44.0656 | 20 |
| ABCC1-like protein | ALEYAHLKPFVQGLPAGLR;ALHAALLAGVLR;FVEESER;FYVATSR;GINLSGGQK;GSLVAIVGAVGSGK;ITIIPQDPVLFSGTLR;LLIGFVQGK;LTLAYR;NINLHVPR;SPIYSHFGESITGASTIR;SSLLSALLGEMNK;STLTLGLFR;TSASVNFKPESEK;VLGPSGLLK;WGVYKHYLMSVGVLSSVITVLMNLILQVFQVGSNYWLTEWANDDK | 0.1412 | 49.4595 | 19 |
| Glyceraldehyde-3-phosphate dehydrogenase | AGAEYIVESTGVFTTTEK;GAQQNIIPAATGAAK;IAVFCER;IGINGFGR;LGKPASYDAIK;LISWYDNEFGYSNR;LTGMAFR;VIDLIK;VIHDNFEIVEGLMTTVHATTATQK;VIPALNGK;VPVANVSVVDLTVR | 0.3976 | 35.6539 | 17 |
| Elongation factor 1-alpha | EHALLAFTLGVK;EVSSYIK;FEEIKK;IGGIGTVPVGR;IGYNPAAVAFVPISGWHGDNMLEASTK;KIGYNPAAVAFVPISGWHGDNMLEASTK;MDSTEPPYSESR;MPWFK;QTVAVGVIK;STTTGHLIYK;SVEMHHEALQEAVPGDNVGFNVK;VETGILKPGTIVVFAPANITTEVK;YAWVLDK;YYVTIIDAPGHR | 0.3585 | 43.7946 | 16 |
| Thioredoxin peroxidase | DIALSDYK;GLFVIDDK;IGCEVIGASTDSHFTHLAWINTPR;LVQAFQYTDK;PLQLTKPAPQFK;QITVNDLPVGR;SVEETLR;TTAVVNGEFK;TTAVVNGEFKDIALSDYK | 0.4615 | 27.8156 | 14 |
| Arylphorin subunit | DPAFYQLYQR;FLDTYEK;FSIFYER;FYELDWFVQK;IIDYLIDYK;LGEVFFYYYQQLLAR;LVPGENKIER;NYEYIR;QYVKPYNHNDLHFVGVK;SSEFVLFK;TFFQFLQK | 0.1533 | 32.7463 | 13 |
| Catalase | AAELASSDPDYSIR;DAAGFIQER;DASLFPSFIHTQK;DLYNAIAK;DPASDQLVNYK;DPASDQLVNYKK;FSTVGGESGSADTVR;FYTDDGNWDLVGNNTPIFFIR;IFETVGK;IFTQVHPDLGSK;LFSYSDTHR;TVDNIVGHLK | 0.2564 | 37.7925 | 12 |
| 2-phospho-D-glycerate hydro-lyase | AAVPSGASTGVHEALELR;DALFLIQDAIQQAGYTGK;FGLDSTAVGDEGGFAPNIQNNK;GVLTAIK;IATAVEK;LAEVYLDFIK;LGANAILGVSLAVAK;NGTYDLDFK;NINDIIAPELLK;QNLEVTQQK;SNPADYLSSEK;TPIQIVGDDLTVTNPK | 0.3557 | 38.2711 | 12 |
| Actin | AVFPSIVGRPR;DLTDYLMK;DSYVGDEAQSK;GYSFTTTAER;IIAPPER;IWHHTFYNELR;QEYDESGPSIVHR;SYELPDGQVITIGNER;TTGIVLDSGDGVSHTVPIYEGYALPHAILR;VAPEEHPVLLTEAPLNPK | 0.3590 | 28.3691 | 11 |
| Translationally-controlled tumor protein homolog | APDQVDVFK;HGLEEEKF;IEGFNPSAEEADEGTDAAVESGRDIVLNHR;IEGFNPSAEEADEGTDAAVESGVDIVLNHR;LEEKAPDQVDVFK;LVDEVIYEVTGK;LVETYAFGDK;LVETYAFGDKK | 0.3779 | 27.2567 | 10 |
| Glutathione S-transferase s2 protein | AAIASYEKDEAVK;LLLAFGDEGFEDHR;QYAQSLSIAR;VVDAVYSIPK;YVFHYFDGK | 0.2745 | 18.0666 | 9 |
| Complex I-49kD | APGFAHLAALEK;GEFGIYLVSDGGSKPYR;LDEVEDVLTTNR;LLNIDIPLR;LVLELDGETVR;TLFAEITR;TSMEALIHHFK | 0.1720 | 23.1636 | 8 |
| Trypsin-like serine protease | AASIAGANYWPGDNSAAWAAGWGTTSSGGSASEQLR;AILTAAHCTVGDAVSR;ILNQNTCR;IVGGSVTTIDR;LGSTWANSGGVVHNVNANLVHGSYNSR;SASTFSFNNNVR | 0.4331 | 17.1159 | 8 |
| Glutathione S-transferase sigma 4 | ALGESVR;EYAQSLAISR;LLLAYGGQEFDDR;LLLAYGGQEFDDRR;LNAIFVK;NVFPDLLEK;YADFVK | 0.2598 | 20.5562 | 7 |
| Moderately methionine rich storage protein | EDLYFPGVK;FNNYVIAR;GEIFTLHIDR;GYWPWLK;LIDININR;LNFVELDTFLYK;VTFDILSDK | 0.0838 | 19.4359 | 7 |
| Methionine-rich storage protein | DPVFWR;ILYFAK;LLLPLGR;LLSYNVYNFDK;TVDVESWWYK;VTDVFVLFK | 0.0652 | 17.7407 | 6 |
| Nucleoside diphosphate kinase | GDLCIEVGR;GLVGTIIER;HYSDLSSKPFFPGLVK;NIIHGSDSVESAK | 0.2640 | 13.7558 | 6 |
| Mitochondrial NADH dehydrogenase iron-sulfur protein 8 | AAQTLFWTELAR;EPATINYPFEK;GFAVTLAHIFK;LLSNGDKWESEIATNIR | 0.2329 | 11.9539 | 6 |
| 14-3-3 zeta | DSTLIMQLLR;EICYDVLGLLDK;NLLSVAYK;QAFDDAIAELDTLNEDSYK;YLAEVATGETR | 0.2429 | 15.8291 | 5 |
| Glutathione S-transferase delta 4 | AIITYLVNK;AVLLTAK;LNPQHTVPTLVDDGFSIWESR;LYFDIGTLYQR | 0.2222 | 12.8407 | 4 |

Table S28 Identified *Spodoptera litura* larval coded proteins associated to HvAV-3h virions (Sl-3)

| Protein ID | Identified peptide sequence | Sequence coverage | Protein Q score | Unique spectra |
| --- | --- | --- | --- | --- |
| Heat shock protein 70 cognate | APAVGIDLGTTYSCVGVFQHGK;ARFEELNADLFR;DAGTISGLNVLR;DNNLLGK;ELEGICNPIITK;ETAEAYLGK;FEELNADLFR;FELTGIPPAPR;HWPFEVVSDGGKPK;IINEPTAAAIAYGLDK;IINEPTAAAIAYGLDKK;ISDSDKQTILDK;ITITNDKGR;KFEDATVQADMK;LLQDFFNGK;LSKEEIER;MKETAEAYLGK;MVNHFVQEFK;MVNHFVQEFKR;NQVAMNPNNTIFDAK;NTTIPTK;QTILDK;QTQTFTTYSDNQPGVLIQVFEGER;SQIHDIVLVGGSTR;STAGDTHLGGEDFDNR;TFFPEEVSSMVLTK;TTPSYVAFTDTER;TVQNAVITVPAYFNDSQR;VEIIANDQGNR;WLDSNQLADKEEYEHK | 0.5008 | 121.5262 | 50 |
| Ubiquitin (Fragment) | EGIPPDQQR;ESTLHLVLR;IQDKEGIPPDQQR;LIFAGKQLEDGR;MQIFVK;TITLEVEPSDTIENVK;TLSDYNIQK;TLSDYNIQKESTLHLVLR;TLTGKTITLEVEPSDTIENVK | 0.9211 | 33.1469 | 34 |
| Heat shock protein 83 | ADLVNNLGTIAK;ALLFVPR;APFDLFENK;DQVANSSFVER;EDLTEYLEEHK;EGLELPEDEEEK;EGLELPEDEEEKK;EKYTEDEELNK;ELISNSSDALDK;ELISNSSDALDKIR;GVVDSEDLPLNISR;HFSVEGQLEFR;HIYYITGENR;IEDVGEDDEEDKK;IVLHIK;LADLLR;LGIHEDSQNR;NADDITQEEYGDFYK;NILDNKVEK;SEGTLTIIDTGIGMTK;YESLTDPSK;YHTSASGDEACSLK;YTEDEELNK;YYEQFSK | 0.3180 | 91.0296 | 31 |
| ATP synthase subunit beta | AHGGYSVFAGVGER;EGNDLYHEMIESGVISLK;FLSQPFQVAEVFTGHAGK;GPIPTDK;IGLFGGAGVGK;IINVIGEPIDERGPIPTDK;ILQDYK;IMDPNIIGAEHYNVAR;IPVGAETLGR;LVLEVAQHLGENTVR;LVPLEETIK;TAAIHAEAPEFVDMSVQQEILVTGIK;TIAMDGTEGLVR;TVLIMELINNVAK;VALTGLTVAEYFR;VVDLLAPYAK | 0.4070 | 60.4640 | 21 |
| ABCC1-like protein | AHANIPDDENVK;ALEYAHLKPFVQGLPAGLR;ALHAALLAGVLR;EVTCAVAPR;FVEESER;FYVATSR;GSLVAIVGAVGSGK;HEVAEGGENLSVGQR;ITIIPQDPVLFSGTLR;LLIGFVQGK;NINLHVPR;QLVCLAR;SLTEEDLWALNPQDSSTEVVPK;STLTLGLFR;TALTSAIYR;TSASVNFKPESEK;VLGPSGLLK | 0.1299 | 67.0994 | 20 |
| Putative acetyltransferase ACT5 | AFDAEVIPVPVPQK;AFDAEVIPVPVPQKK;APSNLSLNEVVIVSAVR;GGAPVIFAEDEEYKR;GSLANVTATELGAIVVR;INVHGGAVSLGHPIGMSGTR;IVGHLCHALK;KQQDDYAIISYK;LLDIDLEK;LNVKPLAR;RLNVKPLAR;TPIGSFK;VIGYADGEREPIDFPIAPSVAIPK | 0.3879 | 49.4262 | 19 |
| 2-phospho-D-glycerate hydro-lyase | AAVPSGASTGVHEALELR;DALFLIQDAIQQAGYTGK;FGLDSTAVGDEGGFAPNIQNNK;GVLTAIK;HLADLAGNADIVLPVPAFNVINGGSHAGNK;IATAVEK;KGVPLYK;LAEVYLDFIK;LGANAILGVSLAVAK;NGTYDLDFK;NINDIIAPELLK;QIFDSR;QNLEVTQQK;SNPADYLSSEK;TPIQIVGDDLTVTNPK;VNQIGSVTESIDAHLLAK;YNQILR | 0.5104 | 59.4029 | 18 |
| Arylphorin subunit | DLHQYSYEIIAR;DPAFYQLYQR;DYDIEANIQNYSNK;FLDTYEK;FSIFYER;FYELDWFVQK;IIDYLIDYK;LGEVFFYYYQQLLAR;LVPGENKIER;NYEYIR;QYVKPYNHNDLHFVGVK;SDVASDAVFK;SSEFVLFK;TFFQFLQK;YYLER | 0.2120 | 52.3542 | 17 |
| Glyceraldehyde-3-phosphate dehydrogenase | AGAEYIVESTGVFTTTEK;GTVDIQDGYLVVNGNK;IGINGFGR;LGKPASYDAIK;LISWYDNEFGYSNR;LTGMAFR;VIDLIK;VIHDNFEIVEGLMTTVHATTATQK;VIPALNGK | 0.3373 | 33.7490 | 15 |
| Elongation factor 1-alpha | EHALLAFTLGVK;IGGIGTVPVGR;IGYNPAAVAFVPISGWHGDNMLEASTK;MDSTEPPYSESR;QLIVGVNK;STTTGHLIYK;SVEMHHEALQEAVPGDNVGFNVK;VETGILKPGTIVVFAPANITTEVK;YAWVLDK;YYVTIIDAPGHR | 0.3153 | 38.7579 | 14 |
| Actin | AGFAGDDAPR;AVFPSIVGRPR;DLTDYLMK;GYSFTTTAER;IIAPPER;IWHHTFYNELR;QEYDESGPSIVHR;SYELPDGQVITIGNER;TTGIVLDSGDGVSHTVPIYEGYALPHAILR;VAPEEHPVLLTEAPLNPK | 0.3564 | 37.0727 | 12 |
| Thioredoxin peroxidase | GLFVIDDK;IGCEVIGASTDSHFTHLAWINTPR;LVQAFQYTDK;PLQLTKPAPQFK;QITVNDLPVGR;TTAVVNGEFK | 0.3846 | 22.5816 | 11 |
| Methionine-rich storage protein | DGTVISLK;ILYFAK;LLLPLGR;NLVVIDWR;RVTDVFVLFK;TGYWPK;TNLLLPTVDMSLMKER;TVDVESWWYK;VTDVFVLFK | 0.0945 | 31.5075 | 9 |
| Nucleoside diphosphate kinase | EIALWFGDK;GDLCIEVGR;GLVGTIIER;HYSDLSSKPFFPGLVK;NIIHGSDSVESAK;NIIHGSDSVESAKK;QMLGATNPADSQPGTIR;TFLMIKPDGVQR | 0.4831 | 31.5854 | 9 |
| Glutathione S-transferase s2 protein | AAIASYEKDEAVK;DWPDFKPK;LLLAFGDEGFEDHR;QYAQSLSIAR;VAFKDWPDFKPK;VVDAVYSIPK;YVFHYFDGK | 0.3333 | 28.6984 | 9 |
| Complex I-49kD | APGFAHLAALEK;HPPYHGVVAPGEK;LDEVEDVLTTNR;LLNIDIPLR;LVLELDGETVR;TLFAEITR;TSMEALIHHFK | 0.1634 | 30.7204 | 9 |
| Glutathione S-transferase sigma 4 | AAVVQYEPDEAVK;EYAQSLAISR;LLLAYGGQEFDDR;LLLAYGGQEFDDRR;LNAIFVK;NVFPDLLEK;RIPQTEWPAFKPK;YADFVK | 0.3480 | 24.2389 | 8 |
| Translationally-controlled tumor protein homolog | APDQVDVFK;IEGFNPSAEEADEGTDAAVESGVDIVLNHR;LEEKAPDQVDVFK;LVDEVIYEVTGK;LVETYAFGDK;LVETYAFGDKK | 0.3314 | 26.3317 | 8 |
| Mitochondrial NADH dehydrogenase iron-sulfur protein 8 | AAQTLFWTELAR;EPATINYPFEK;GFAVTLAHIFK;GFAVTLAHIFKEPATINYPFEK;LLSNGDKWESEIATNIR | 0.2329 | 19.7789 | 8 |
| Trypsin-like serine protease | AASIAGANYWPGDNSAAWAAGWGTTSSGGSASEQLR;AILTAAHCTVGDAVSR;IVGGSVTTIDR;LGSTWANSGGVVHNVNANLVHGSYNSR;SASTFSFNNNVR;TMDNDIAILR | 0.4409 | 21.9431 | 7 |
| Catalase | AAELASSDPDYSIR;DAAGFIQER;DASLFPSFIHTQK;DLYNAIAK;FSTVGGESGSADTVR;FSTVGGESGSADTVRDPR;IFTQVHPDLGSK;LFSYSDTHR;NGPTLLQDVNFLDEISSFDR;QVFDDAQK;TVDNIVGHLK;VAAGLAPFKK | 0.2584 | 21.2631 | 6 |
| Cytochrome c oxidase subunit 2 | IMVTATDVIHSWTIPSLGVK;LLDVDNR;LLYLLDELNNPLITLK;NFINWINNYS | 0.2335 | 14.0407 | 5 |
| 14-3-3 zeta | DSTLIMQLLR;EVTETGVELSNEER;QAFDDAIAELDTLNEDSYK;YLAEVATGETR | 0.2186 | 16.2506 | 4 |
| Glutathione S-transferase delta 4 | AIITYLVNK;GSSLYPEEPK;LNPQHTVPTLVDDGFSIWESR;LYFDIGTLYQR | 0.2361 | 16.2506 | 4 |
| Putative acetyltransferase ACT1 | AEITPVTLTVK;DEVDNFALR;LGAQFGITR;TAFGTFGGVFR | 0.101 | 10.0784 | 4 |
| Cytochrome P450 | DADAGFATVEESHVGK;FTFWR;GLLLLHGDEWK;LVQEIR | 0.0742 | 13.4830 | 4 |
| Moderately methionine rich storage protein | EDLYFPGVK;FNNYVIAR;GEIFTLHIDR;GYWPWLK;LIDININR;LNFVELDTFLYK;VTFDILSDK;YYLER | 0.0904 | 15.7976 | 4 |
